# Persistent variations of blood DNA methylation associated with treatment exposures and risk for cardiometabolic outcomes in long-term survivors of childhood cancer in the St. Jude Lifetime Cohort

**DOI:** 10.1186/s13073-021-00875-1

**Published:** 2021-04-06

**Authors:** Nan Song, Chia-Wei Hsu, Haitao Pan, Yinan Zheng, Lifang Hou, Jin-ah Sim, Zhenghong Li, Heather Mulder, John Easton, Emily Walker, Geoffrey Neale, Carmen L. Wilson, Kirsten K. Ness, Kevin R. Krull, Deo Kumar Srivastava, Yutaka Yasui, Jinghui Zhang, Melissa M. Hudson, Leslie L. Robison, I-Chan Huang, Zhaoming Wang

**Affiliations:** 1 Department of Epidemiology and Cancer Control, St. Jude Children’s Research Hospital, 262 Danny Thomas Place, MS 735, Memphis, TN 38105 USA; 2 Department of Pharmacy, Chungbuk National University, Cheongju, Korea; 3 Department of Biostatistics, St. Jude Children’s Research Hospital, Memphis, TN USA; 4 Department of Preventive Medicine, Northwestern University, Chicago, IL USA; 5 Department of Computational Biology, St. Jude Children’s Research Hospital, Memphis, TN USA; 6 Hartwell Center, St. Jude Children’s Research Hospital, Memphis, TN USA; 7 Department of Oncology, St. Jude Children’s Research Hospital, Memphis, TN USA

**Keywords:** Blood DNA methylation, Cancer treatment, Childhood cancer survivorship, Cardiometabolic conditions, Epigenome-wide association study, Mediation analysis

## Abstract

**Background:**

It is well-established that cancer treatment substantially increases the risk of long-term adverse health outcomes among childhood cancer survivors. However, there is limited research on the underlying mechanisms. To elucidate the pathophysiology and a possible causal pathway from treatment exposures to cardiometabolic conditions, we conducted epigenome-wide association studies (EWAS) to identify the DNA methylation (DNAm) sites associated with cancer treatment exposures and examined whether treatment-associated DNAm sites mediate associations between specific treatments and cardiometabolic conditions.

**Methods:**

We included 2052 survivors (median age 33.7 years) of European ancestry from the St. Jude Lifetime Cohort Study, a retrospective hospital-based study with prospective clinical follow-up. Cumulative doses of chemotherapy and region-specific radiation were abstracted from medical records. Seven cardiometabolic conditions were clinically assessed. DNAm profile was measured using MethylationEPIC BeadChip with blood-derived DNA.

**Results:**

By performing multiple treatment-specific EWAS, we identified 935 5′-cytosine-phosphate-guanine-3′ (CpG) sites mapped to 538 genes/regions associated with one or more cancer treatments at the epigenome-wide significance level (*p* < 9 × 10^−8^). Among the treatment-associated CpGs, 8 were associated with obesity, 63 with hypercholesterolemia, and 17 with hypertriglyceridemia (false discovery rate-adjusted *p* < 0.05). We observed substantial mediation by methylation at four independent CpGs (cg06963130, cg21922478, cg22976567, cg07403981) for the association between abdominal field radiotherapy (abdominal-RT) and risk of hypercholesterolemia (70.3%) and by methylation at three CpGs (cg19634849, cg13552692, cg09853238) for the association between abdominal-RT and hypertriglyceridemia (54.6%). In addition, three CpGs (cg26572901, cg12715065, cg21163477) partially mediated the association between brain-RT and obesity with a 32.9% mediation effect, and two CpGs mediated the association between corticosteroids and obesity (cg22351187, 14.2%) and between brain-RT and hypertriglyceridemia (cg13360224, 10.5%). Notably, several mediator CpGs reside in the proximity of well-established dyslipidemia genes: cg21922478 (*ITGA1*) and cg22976567 (*LMNA*).

**Conclusions:**

In childhood cancer survivors, cancer treatment exposures are associated with DNAm patterns present decades following the exposure. Treatment-associated DNAm sites may mediate the causal pathway from specific treatment exposures to certain cardiometabolic conditions, suggesting the utility of DNAm sites as risk predictors and potential mechanistic targets for future intervention studies.

## Background

Progress in cancer treatment has dramatically improved the 5-year survival following a childhood cancer diagnosis to more than 85% between 2010 and 2016 [1]. Thus, the population of childhood cancer survivors has grown rapidly and is estimated to exceed 500,000 persons in the USA [2]. Unfortunately, the treatment of childhood malignancies is associated with long-term morbidity and mortality [3–7]. Mounting evidence suggests that reduced physical activity, muscular weakness, metabolic derangements, and cognitive declines are common problems among adults treated for childhood malignancies [7–11]. Furthermore, premature cellular senescence, sterile inflammation, and mitochondrial dysfunction resulting from a primary cancer diagnosis or treatment-related toxicity may contribute to adverse health outcomes [12]. Accordingly, survivors of childhood cancer often develop treatment-related late effects with 60% to more than 90% of survivors experiencing one or more long-term chronic health conditions (CHCs) [13], an approximately 2-fold greater burden of CHCs than community controls [4]. Treatment-related adverse health outcomes encompass a broad range of CHCs [4, 14, 15], hospitalizations [16], premature frailty [17], and early mortality [14]. Some of the most commonly observed CHCs among survivors include obesity [18, 19], diabetes mellitus [20], cardiovascular diseases [21, 22], hypertension [9, 22, 23], and subsequent neoplasms [24, 25].

While the substantially increased risk and total burden of adverse health outcomes among childhood cancer survivors have been extensively described, there is a need to unravel the complex interplay between therapeutic exposures and genetic susceptibility in order to elucidate the pathogenesis of specific health conditions [6]. Pathogenic germline mutations in DNA repair genes contribute to the subsequent neoplasm risk in childhood cancer survivors, especially among those who received high cumulative doses of specific agents and modalities [26]. Unlike germline genetics (DNA sequence), which is largely static throughout the life course, epigenetic patterns are plastic and can be modified in response to internal and external insults including medical treatments [27]. Population-based studies among breast cancer [28] and gastric cancer [29] patients have provided evidence supporting that chemotherapy, radiotherapy, or a combination of anticancer treatments have a profound impact on epigenetic alterations, primarily in the form of CpG methylation. The processes leading to aberrant DNA methylation (DNAm) are poorly understood. How epigenetic alterations resulting from cancer therapy interact with downstream gene regulation machineries and ultimately lead to the development of CHCs in individual survivors is still largely unknown. Possible biological mechanisms have been suggested; for instance, cellular oxidative stress and DNA damage can induce aberrant DNAm by recruiting DNA methyltransferase complex [30]. Emerging evidence suggests that alterations in DNAm in the blood can at least influence immune regulation [31] or blood lipids and metabolites [32]. Several population-based studies of adult-onset cancers have identified treatment-induced blood DNAm changes and associated these changes with health outcomes, specifically cognitive decline in breast cancer patients [28] and poor survival for colorectal cancer [33], lung cancer [34, 35], and ovarian cancer patients [36, 37]. Thus, epigenetic alterations due to cancer therapeutic agents may mediate or modify gene regulation potentially resulting in systemic changes contributing to the development of CHCs.

It is biologically plausible that treatments used during active childhood cancer could leave an epigenetic mark. Hence, we hypothesized that cancer treatment modalities cause aberrant hypo- or hyper-DNAm, which may affect the long-term risk of CHC among childhood cancer survivors. In this study, epigenome-wide association studies (EWAS) were conducted among adult survivors of childhood cancer participating in the St. Jude Lifetime Cohort Study (SJLIFE) to identify differentially methylated DNA CpG sites between survivors exposed or unexposed to a certain treatment and their associations with CHCs. We specifically focused on seven common cardiometabolic conditions including obesity, hypertension, hypercholesterolemia, hypertriglyceridemia, abnormal glucose metabolism, cardiomyopathy, and myocardial infarction, given that blood DNAm plays a role in the regulation of blood lipids and other metabolites [32].

## Methods

### Study population

SJLIFE is a retrospective cohort study with prospective follow-up of survivors diagnosed with childhood cancer and treated at St. Jude Children’s Research Hospital, described elsewhere [38, 39]. Participants complete questionnaires assessing demographic and epidemiological factors and receive comprehensive medical and laboratory assessments at each follow-up to characterize their health conditions. Genome-wide EPIC methylation profiling (Illumina, San Diego, CA, USA) was performed using blood-derived DNA from 2689 SJLIFE survivors. Subsequent sample exclusion criteria included the following: (1) low total intensity of DNAm (*n* = 3), (2) no whole-genome sequencing data (*n* = 46), (3) age at blood draw under 18 years old (*n* = 218), and (4) population admixture coefficient for CEU population < 80% (*n* = 370) based on the STRUCTURE analysis [40] with three continental references (JPT+CHB, CEU, YRI) from 1000 Genomes Project. Accordingly, we included 2052 childhood cancer survivors of European ancestry in statistical analyses (Additional file 1: Fig. S1).

### Treatment exposures

Treatment exposure information was extracted from medical records using a structured protocol [38]. Briefly, using radiation oncology treatment records, region-specific radiotherapy (RT) dosimetry, including brain-RT, chest-RT, abdominal-RT, and pelvic-RT, was estimated [41]. Cumulative doses and exposure status of individual chemotherapeutic agents were abstracted from medical records. The number of exposed survivors provided sufficient statistical power to analyze alkylating agents, anthracyclines, antimetabolites, asparaginase enzymes, epipodophyllotoxins, corticosteroids, and vinca alkaloids. Equivalency approaches were applied for cumulative alkylating agent exposure [42] and anthracycline exposure [43]. Region-specific radiation doses are described elsewhere [44] and in Supplementary Methods in Additional file 1.

### Chronic health conditions

A modification of the Common Terminology Criteria for Adverse Events (version 4.03, National Cancer Institute) [45] was applied to clinically ascertain medical outcomes and score for severity [39]. Clinical outcomes were severity-graded as 0 (no problem), 1 (mild), 2 (moderate), 3 (severe or disabling), and 4 (life-threatening) [39]. All CHCs with grades ≥1 were grouped together as cases. Considering DNAm is known to play an essential role in the regulation of blood lipids or metabolites [46], we included in this study seven common cardiometabolic CHCs: abnormal glucose metabolism, cardiomyopathy, hypercholesterolemia, hypertriglyceridemia, hypertension, myocardial infarction, and obesity. Only incident CHCs that occurred after the blood draw for methylation profiling as part of their long-term follow-up clinical assessment (median = 29.4 years and interquartile range [IQR] = 22.6–36.8 years from primary cancer diagnosis) were considered in the current study.

### DNA methylation profiling

Genome-wide methylation data were generated using Infinium MethylationEPIC BeadChip array (Illumina, San Diego, CA, USA). Genomic DNA (250 ng) was extracted from blood samples according to the standard procedures as described previously [47]. Further, bisulfite treatment, array hybridization, and scanning are provided in Supplementary Methods in Additional file 1. The raw intensity data were exported from Illumina Genome Studio and analyzed in R (version 3.6.3) using the minfi package [48] including a cross-array quantile normalization. Methylation is described as a *β* value, which is a continuous variable ranging between 0 (no methylation) and 1 (full methylation). In any sample, a probe with a detection *p*-value of more than 0.01 was assigned missing status. Any sample or probe with more than 5% missing values was excluded from the downstream analysis. Non-specific or cross-reactive probes, probes with SNPs nearby the CpG site, or probes on sex chromosomes (X, Y) were also excluded. A total number of 686,880 probes remained for further analyses. Marker intensities were normalized by quantile normalization. *M*-value (i.e., logit transformation of *β* value) was subsequently calculated and used in regression analyses [49]. Six leukocyte subtype proportions (neutrophils, monocytes, CD8+ T cells, CD4+ T cells, natural killer cells, and B cells) were estimated based on methylation signatures using Houseman’s method [50, 51]. A principal components analysis of methylation levels of all CpG sites that passed QC was performed to quantify latent structures or batch effects in the data. The array annotations provided by Illumina were used to map probes to their corresponding genes.

### Statistical analysis

To identify DNAm level at each CpG site influenced by specific treatment for childhood cancer, EWAS analysis was performed using multiple linear regression of methylation level at each CpG site (dependent variable, continuous) on each treatment exposure status (independent variable, binary: exposed vs non-exposed) or the cumulative dose (independent variable, categorical, by tertiles or different dose ranges) with covariate adjustments including sex, age, other cancer treatment exposures (see below), leukocyte subtype proportions, top three genetic principal components, and top four methylation principal components determined by the change rate of eigenvalues. The correlation between every pair of binary treatment exposures was described by the phi coefficient, and the statistical significance (*p*-value) of its departure from 0 was assessed. Adjustment for other cancer treatment exposures in the EWAS was made if the phi coefficient with the treatment exposure of interest is less than 0.4 and with a *p*-value > 0.05. R package CpGassoc [52] was used for the EWAS multiple linear regression analysis, and we used *p*-value < 9 × 10^−8^ corresponding to 5% family-wise error as the threshold for genome-wide significance [53]. Quantile-quantile plots showing the observed and expected *p*-values were generated using the CpGassoc R package. The genomic inflation factors were in the range of 1.12–1.63 (Additional file 1: Fig. S2). We used genetic control-adjusted *p*-values instead of raw *p*-values for the assessment of epigenome-wide significance (*P*_gc-adjusted_ < 9 × 10^−8^). Manhattan plots were generated for visualization of EWAS results using the CMplot R package [54], and Venn diagrams for visualizing unique and overlapping CpGs associated with different cancer treatments were generated using the VennDiagram R package [55]. The distribution of treatment-associated CpGs and overall array content was compared according to the CpG island regions and genomic functional annotations. After mapping significant treatment-associated CpG hits to genes, we conducted a statistical overrepresentation test of the mapped genes with the PANTHER classification system [56]. For this pathway analysis, both GO biological process complete [57] and PANTHER pathway were considered.

**Fig. 1:**
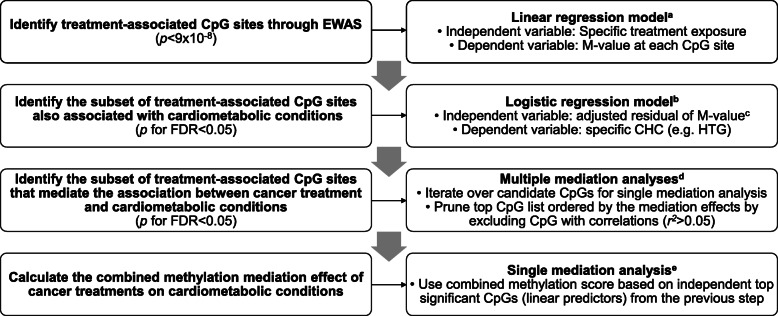
Statistical analysis workflow. Abbreviations: CHC, chronic health condition; EWAS, epigenome-wide association study; HTG, hypertriglyceridemia. ^a^Linear regression model was adjusted for covariates including sex, age, other cancer treatment exposures, leukocyte subtype proportions, top three genetic principal components, and top four methylation principal components. ^b^Logistic regression model was adjusted for covariates including sex, age, and CHC-specific polygenic risk score. ^c^Residual of *M*-value was calculated based on linear regression adjusted for covariates including sex, age, leukocyte subtype proportions, significant genetic principal components, and methylation principal components. ^d^Mediation analysis included two regression modes: a logistic regression model with CHC status as an outcome, specific treatment as treatment variable (term used for the exposure in the Mediation R package), residual *M*-value for a CpG site as a mediator variable and adjusted for age, sex, CHC-specific polygenic risk score, and other cancer treatment exposures; a linear regression model with residual *M*-value for a CpG site as an outcome, specific treatment as treatment variable and other significant treatments as covariates. ^e^Mediation analysis as above except for replacing residual *M*-value for a CpG site with a combined methylation score by summing up the residual *M*-values for multiple CpG sites that were found to be significant mediators individually

We systematically identified SNPs that were previously reported to be associated with each specific CHC using the GWAS catalog, pruned the SNPs to satisfy pairwise *r*^2^ < 0.3, and constructed a polygenic risk score by summing up all risk alleles carried by each survivor. The polygenic risk score was added as a covariate in the regression model for CHC risk. In addition, we extracted the genetic variants for three DNA methyltransferases (DNMTs) including *DNMT1*, *DNMT3A*, and *DNMT3B*, and three ten-eleven translocation (TET) enzymes including *TET1*, *TET2*, and *TET3* for the whole-genome sequencing data. We identified two survivors carrying germline truncation mutations in the *TET2* gene. Both *TET2* mutation carriers were excluded from the treatment EWAS and CHC association analyses. We further investigated the associations between the residual methylation level of each treatment-associated methylation site (independent variable, residuals derived from the multiple linear regression of the EWAS model above but without adjusting for treatments) and a specific incident CHC (dependent variable, binary) using a logistic regression model with adjustments for age, sex, and CHC-specific polygenic risk score. False discovery rate (FDR)-adjusted *p*-values (*P*_FDR_) were obtained to control for multiple testing [58]. Survivors with specific CHC occurring prior to collection of blood for the methylation profiling were excluded from this analysis. Mediation analysis was performed by treating the CpG sites as hypothesized causative mediators for the association of treatment exposures with risk of incident CHCs using the Mediation R package [59]. Statistical analysis workflow was summarized in Fig. 1. All statistical analyses were performed by using R.3.6.3 [2] or SAS 9.4 (SAS Institute Inc., Cary, NC, USA), and all statistical tests were two-sided.

## Results

### Characteristics of the study population

Table 1 shows the characteristics of the 2052 childhood cancer survivors of European ancestry. Survivors were previously diagnosed with leukemia (34.1%), lymphoma (21.8%), sarcoma (13.4%), central nervous system (CNS) tumors (11.3%), embryonal tumors (13.5%), and others (6.0%). Treatment exposures comprised classic alkylating agents (58.2%), anthracyclines (58.0%), antimetabolites (49.9%), asparaginase enzymes (58.0%), epipodophyllotoxins (34.6%), corticosteroids (47.0%), vinca alkaloids (72.2%), brain-RT (30.7%), chest-RT (28.1%), abdominal-RT (20.1%), and pelvic-RT (17.2%). The incidence of cardiometabolic conditions in the study population was abnormal glucose metabolism (18.0%, 95% CI = 16.2–19.9%), cardiomyopathy (9.6%, 95% CI = 8.4–11.1%), hypercholesterolemia (32.8%, 95% CI = 30.5–35.3%), hypertriglyceridemia (25.9%, 95% CI = 23.8–28.2%), hypertension (53.3%, 95% CI = 50.7–56.0%), myocardial infarction (2.5%, 95% CI = 1.9–3.3%), and obesity (62.0%, 95% CI = 59.6–64.5%). The median age at diagnosis was 8.5 (range = 0.0–23.6) years, and the median age at DNA sampling was 33.7 (range = 18.0–66.4) years.

**Table 1:** Characteristics of the study population

Characteristics	Number	Percent
Total	2052	100.0
Sex		
Male	1084	52.8
Female	968	47.2
Diagnosis		
Leukemia	699	34.1
Acute lymphoblastic leukemia	644	31.4
Acute myeloid leukemia	53	2.6
Other leukemia	2	0.1
Lymphoma	448	21.8
Hodgkin lymphoma	288	14.0
Non-Hodgkin lymphoma	160	7.8
Sarcoma	274	13.4
Ewing sarcoma	74	3.6
Osteosarcoma	74	3.6
Rhabdomyosarcoma	71	3.5
Soft tissue sarcoma	55	2.7
CNS tumors	231	11.3
Astrocytoma or glioma	93	4.5
Medulloblastoma or PNET	56	2.7
Ependymoma	26	1.3
Other CNS tumors	56	2.7
Embryonal	276	13.5
Wilms tumor	134	6.5
Neuroblastoma	107	5.2
Germ cell tumor	35	1.7
Others	124	6.0
Retinoblastoma	45	2.2
Hepatoblastoma	13	0.6
Melanoma	12	0.6
Carcinomas	24	1.2
Others	30	1.5
Chemotherapy		
Alkylating agent, classic	1194	58.2
Alkylating agent, heavy metal	239	11.6
Alkylating agent, non-classic	67	3.3
Anthracyclines	1190	58.0
Anti-metabolites	1024	49.9
Asparaginase enzymes	1190	58.0
Epipodophyllotoxins	709	34.6
Corticosteroids	965	47.0
Vinca alkaloids	1482	72.2
Radiation therapy (RT)		
Brain RT	629	30.7
Chest RT	577	28.1
Abdominal RT	412	20.1
Pelvic RT	352	17.2
CHCs	Incident *N*/*N* at risk	(%, 95% CI)
Abnormal glucose metabolism	302/1680	(18.0, 16.2–19.9)
Cardiomyopathy	168/1742	(9.6, 8.4–11.1)
Hypercholesterolemia	479/1461	(32.8, 30.5–35.3)
Hypertriglyceridemia	399/1539	(25.9, 23.8–28.2)
Hypertension	727/1365	(53.3, 50.7–56.0)
Myocardial infarction	47/1892	(2.5, 1.9–3.3)
Obesity	942/1519	(62.0, 59.6–64.5)
Median age at diagnosis, years (range)	8.5	(0.0–23.6)
Median age at DNA sampling, years (range)	33.7	(18.0–66.4)
Median follow-up from primary diagnosis, years (range)	29.4	(7.5–55.6)

*Abbreviations*: *CNS* central nervous system, *PNET* primitive neuroectodermal tumor, *RT* radiation therapy, *CHC* chronic health condition, and *CI* confidence interval

### Treatment-specific associations of DNA methylation

**Fig. 2:**
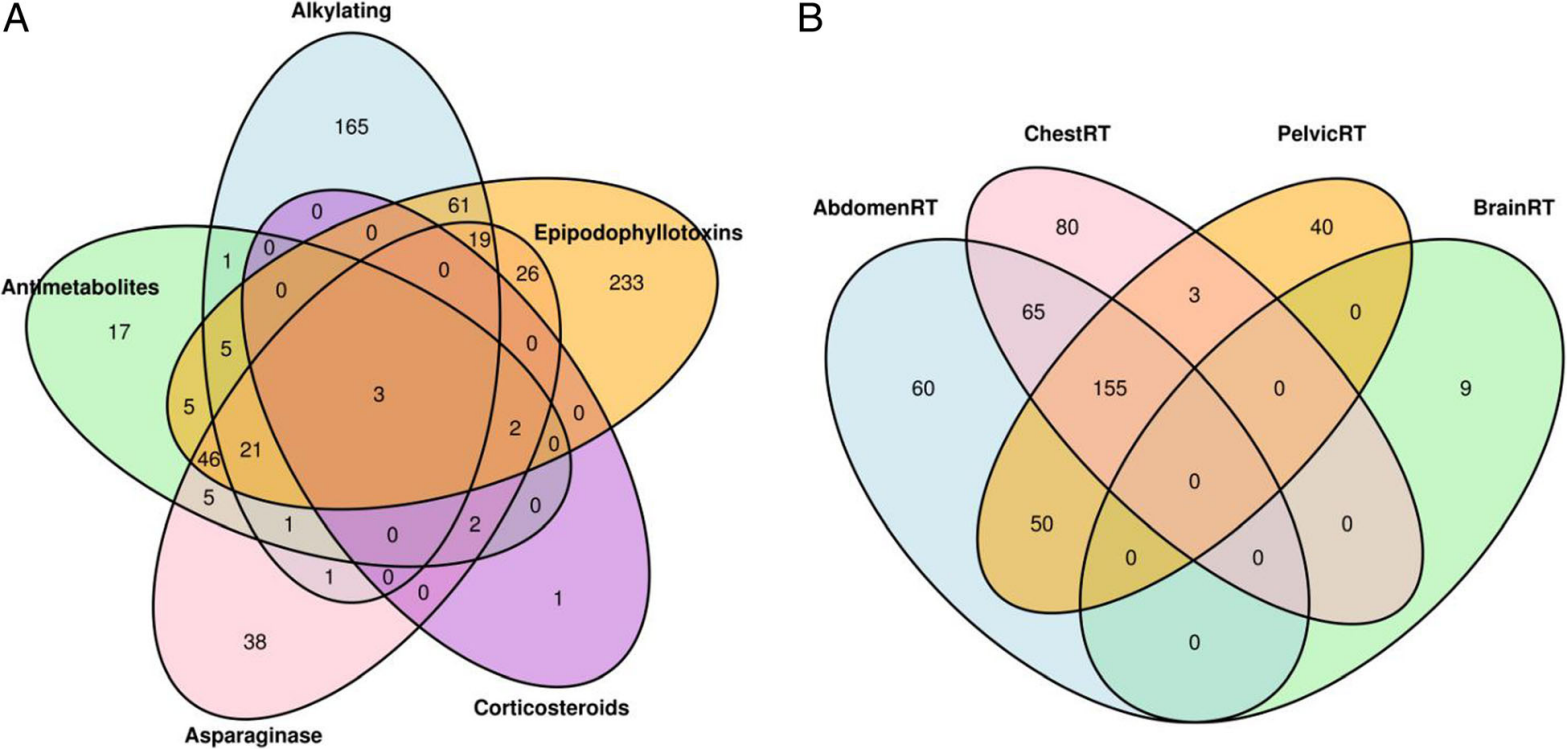
Venn diagram showing the overlap of DNA methylation sites associated with specific cancer treatments. **a** Chemotherapy. **b** Radiation therapy

Examination of all the pairwise correlations among the 11 different treatments (Additional file 1: Table S1) showed 9 pairs of treatments with moderate to high correlations (phi coefficient > 0.4 and *p* < 0.05), including antimetabolites and asparaginase enzymes, antimetabolites and corticosteroids, antimetabolites and vinca alkaloids, asparaginase enzymes and corticosteroids, asparaginase enzymes and epipodophyllotoxins, corticosteroids and vinca alkaloids, chest-RT and abdomen-RT, chest-RT and pelvis-RT, and abdomen-RT and pelvis-RT. By performing multiple treatment-specific EWAS analyses after excluding the strongly correlated treatments from covariate adjustments, a total of 935 CpG sites mapped to 538 genes/regions were associated with one or more cancer treatments at the epigenome-wide significant level (*p* < 9 × 10^−8^). These epigenome-wide results showed 277 DNAm hits for alkylating agent, 108 hits for antimetabolites, 164 hits for asparaginase enzymes, 421 hits for epipodophyllotoxin, 8 hits for corticosteroids, 9 hits for brain-RT, 303 hits for chest-RT, 330 hits for abdominal-RT, and 248 hits for pelvic-RT, but no hit for anthracyclines and vinca alkaloids (Additional file **1**: Fig. S3, Table S2). Figure 2 shows the overlap of DNAm sites associated with a specific chemotherapeutic agent or RT field. Among 652 CpG sites identified to be associated with chemotherapy exposure, there were 198 CpG sites associated with two or more chemotherapy agents, and the remaining were specifically associated with alkylating agents (*n* = 165), antimetabolites (*n* = 17), asparaginase enzymes (*n* = 38), epipodophyllotoxins (*n* = 233), and corticosteroids (*n* = 1) (Fig. 2a). Among 462 CpG sites associated with RT exposure, there were 273 CpG sites associated with two or more region-specific RT treatments, and the remaining are specifically associated with brain-RT (*n* = 9), chest-RT (*n* = 80), abdominal-RT (*n* = 60), and pelvic-RT (*n* = 40) (Fig. 2b).

Among significant CpGs for each treatment status, there were also statistically significant linear dose-response relationship between the continuous cumulative dose of the specific treatment and DNAm level showing 264/277 (95.3%) hits for alkylating agents, 59/108 (54.6%) hits for antimetabolites, 129/164 (78.7%) hits for asparaginase enzymes, 5/8 (62.5%) hits for corticosteroids, 92/421 (21.8%) hits for epipodophyllotoxins, 7/9 (77.8%) hits for brain-RT, 271/303 (86.6%) hits for chest-RT, 295/330 (89.4%) hits for abdominal-RT, and 240/248 (96.8%) hits for pelvic-RT. By analyzing the association between 9 paired combinations of treatments and DNAm at CpGs (i.e., comparing DNAm between the group of survivors who received both treatments with the group of survivors who received neither of the two treatments), we identified 276 additional epigenome-wide significant CpGs which were not found by analyzing each of the two treatments separately (Additional file 1: Table S3). In the pathway analysis, we found 20 GO biological processes for chest-RT-associated hits and 12 processes for epipodophyllotoxins-associated hits with *P*_FDR_ < 0.05 (Additional file 1: Table S4). When distributions of treatment-associated CpG sites and overall array content were compared by CpG islands or genomic functional annotations, the treatment-associated CpGs were significantly overrepresented in the categories of open sea and intergenic, and significantly unrepresented in the categories of CpG islands, TSS1500, TSS200, 5′-UTR, and 1st exon (Additional file 1: Fig. S4).

### Association of treatment-associated methylation sites with CHC

Evaluation of the association between each treatment-related CpG and each CHC using logistic regression models showed that the highest number of abdominal-RT-related CpGs (*n* = 63) were significantly (*P*_FDR_ < 0.05) associated with hypercholesterolemia, followed by brain-RT-related CpGs (*n* = 7) with obesity, abdominal-RT-related CpGs (*n* = 16) with hypertriglyceridemia, brain-RT-related CpGs (*n* = 1) with hypertriglyceridemia, and corticosteroids-related CpG (*n* = 1) with obesity (Additional file 1: Table S5). There was no single treatment-associated CpG site associated with abnormal glucose metabolism, cardiomyopathy, myocardial infarction, or hypertension.

### Treatment-associated methylation sites mediate the effect of treatment on CHC

The multivariable associations between treatment exposures and CHCs are presented in Additional file 1: Table S6. Associations were observed between anthracyclines and cardiomyopathy (*p* = 0.01), epipodophyllotoxins and hypercholesterolemia (*p* = 0.02), brain-RT and hypercholesterolemia (*p* < 0.001) and hypertriglyceridemia (*p* < 0.001), abdominal-RT and abnormal glucose metabolism (*p* = 0.001), hypercholesterolemia (*p* = 0.03) and hypertriglyceridemia (*p* = 0.001), chest-RT and hypertension (*p* = 0.03), and pelvis-RT and myocardial infarction (*p* = 0.04).

**Fig. 3:**
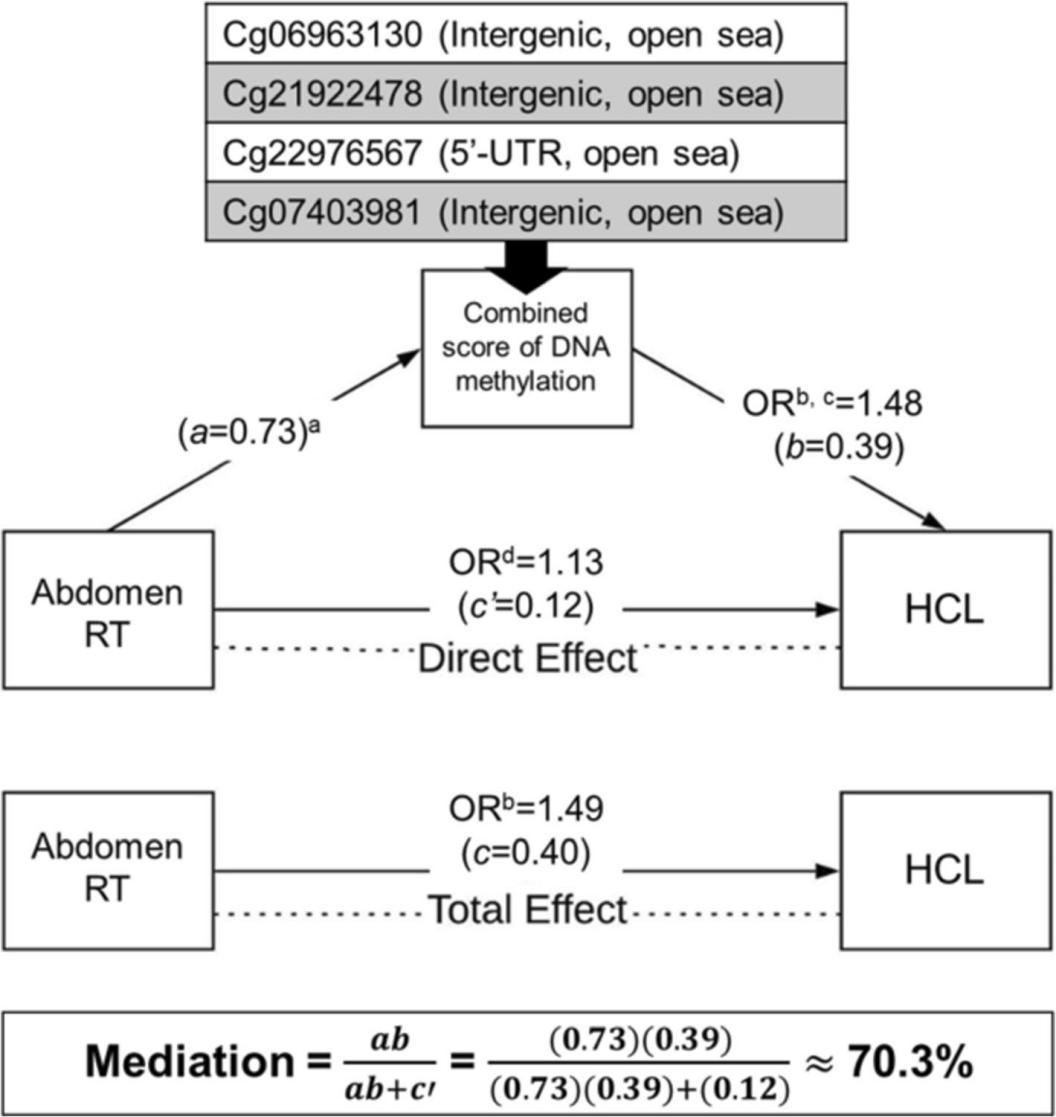
DNA methylation mediates the associations between treatment exposures and CHCs. The diagram illustrates the combined mediation effect for the association between abdominal RT and HCL. Abbreviations: HCL, hypercholesterolemia; RT, radiation therapy. ^a^Linear regression model with residual *M*-value for a CpG site as an outcome and was adjusted for covariates including sex, age, other cancer treatment exposures, leukocyte subtype proportions, top three genetic principal components, and top four methylation principal components. ^b^Logistic regression model with CHC status as an outcome, a specific treatment as an independent variable and was adjusted for covariates including sex, age, and CHC-specific polygenic risk score, and other significant treatments. ^c^Residual of *M*-value was calculated based on linear regression adjusted for covariates including sex, age, leukocyte subtype proportions, significant genetic principal components, and methylation principal components. ^d^Mediation analysis included two regression modes: a logistic regression model with CHC status as an outcome, specific treatment as treatment variable (term used for the exposure in the Mediation R package), residual *M*-value for a CpG site as a mediator variable and adjusted for age, sex, CHC-specific polygenic risk score, and other cancer treatment exposures; a linear regression model with residual *M*-value for a CpG site as an outcome, specific treatment as treatment variable and other significant treatments as covariates. The final mediation analysis used a combined methylation score by summing up the residual *M*-values for multiple CpG sites that were found to be significant mediators individually

There were 63 CpG sites whose methylation levels were associated with abdominal-RT at the genome-wide significance level and also with hypercholesterolemia after adjusting for multiple comparison. In the mediation analysis, each of these CpGs was considered as a hypothesized mediator variable, abdominal-RT as the exposure, and status of hypercholesterolemia (binary) as the outcome while adjusting for sex, age, polygenic risk score, and brain-RT (another exposure significantly associated with hypertriglyceridemia). Eighteen CpGs were identified with significant average causal mediation effects (ACME) (*P*_FDR_ < 0.05). Using pairwise Pearson correlation coefficient *r*^2^ threshold of 0.05, four independent CpGs were obtained by top-down pruning the 18 CpGs sorted by estimated ACME in decreasing order. For the final mediation analysis, using a combined score (i.e., summation of the methylation levels of four CpGs) as a mediator variable, substantial mediation (70.3%) was achieved for the effect of abdominal-RT on hypercholesterolemia (OR = 1.49) (Fig. 3 and Table 2). Using the same strategy, we found a combined score encompassing 3 CpGs accounted for 54.6% mediated effects of abdominal-RT on the risk of hypertriglyceridemia (OR = 1.50), and another set of three CpGs partially mediated (32.9%) the effect of brain-RT on the risk of obesity (OR = 1.56). One CpG (cg13360224) partially mediated the effect of brain-RT on the risk of hypertriglyceridemia (10.5%, OR = 1.75), and another CpG (cg22351187) mediated the effect of corticosteroids on the risk of obesity (14.2%, OR = 1.56). Among 12 mediator CpGs (Table 2), five were positively associated and seven were negatively associated with a specific cardiometabolic condition (Additional file 1: Fig. S5).

**Table 2:** DNA methylation mediation effect of treatment exposures and CHCs

Treatment	CHC	Total effect, OR	CpG	Chromosome	Gene	Functional genomic annotation	CpG island region	% mediation
Abdominal RT	HCL	1.49	cg06963130	chr2	*NA*	Intergenic	Open sea	35.5
			cg21922478	chr5	*ITGA1*; *CTD-2175A23.1*	Intergenic	Open sea	23.8
			cg22976567	chr1	*LMNA*	5′-UTR	Open sea	22.1
			cg07403981	chr1	*NA*	Intergenic	Open sea	14.6
			Combined score^a^					70.3
Abdominal-RT	HTG	1.5	cg19634849	chr5	*CYSTM1*; *PFDN1*	Body	Open sea	28.6
			cg13552692	chr18	*CCDC102B*	5′-UTR	Open sea	24.8
			cg09853238	chr6	*NA*	Intergenic	Open sea	17.2
			Combined score^a^					54.6
Brain-RT	HTG	1.75	cg13360224	chr6	*RP11-359 N11.1*	Intergenic	Open sea	10.5
Brain-RT	Obesity	1.57	cg26572901	chr7	*NA*	Intergenic	Open sea	16.3
			cg12715065	chr6	*NA*	Intergenic	Open sea	14.3
			cg21163477	chr16	*XYLT1*	Body	Open sea	11.7
			Combined score^a^					32.9
Corticosteroids	Obesity	1.56	cg22351187	chr12	*KRT80*	TSS1500	Open sea	14.2

*Abbreviations*: *CHC* chronic health condition, *OR* odds ratio, *RT* radiation therapy, *HTG* hypertriglyceridemia, and *HCL* hypercholesterolemia

^a^Calculated by summing the DNA methylation values of CpG sites with the significant mediation effect of treatment exposures and CHCs

## Discussion

The biological basis underlying treatment-related risks for adverse health outcomes among survivors of childhood cancer is largely unknown. We speculated that one plausible casual pathway is the acquisition and persistent soma-wide alterations in DNAm. In this study, the first large-scale association analyses between cancer treatments and DNA methylation in survivors of childhood cancer, our mediation analyses provide compelling evidence in substantiating this hypothesis. Moreover, we identify unique and overlapping methylation signatures across different cancer treatments that may serve as mechanistic targets for future intervention studies.

Many of the genome-wide significant treatment-associated CpGs have established associations with aging, smoking, diet, and other lifestyle factors [60], suggesting there is a common set of CpGs serving as “sensors” that are sensitive to both internal and external environments. Among chemotherapy exposures, epipodophyllotoxins had the highest number of CpG hits, followed by alkylating agents, asparaginase enzymes, antimetabolites, and corticosteroids, and there was no single hit for anthracyclines or vinca alkaloids. Interestingly, the majority of alkylating agent-associated CpGs (68%) were hypomethylated (i.e., negative correlations between DNAm level and alkylating exposure status). Alkylating agents primarily cause alkylated DNA adducts which can be repaired by base excision [61]. It is known that base excision repair plays a role in epigenetic regulation and may erase epigenetic marks (i.e., 5-methylcytosines) by converting them back to cytosine [62]. Also, it is important to note that DNA methylation is the most common type of alkylation, and a methyl group (CH_3_) is a special form of the alkyl group (C_*n*_H_2*n* + 1_). Therefore, we speculated that it is possible that alkylating agents have a more direct impact on DNA methylation. Among radiation exposures, there were a comparable number of hits among chest-RT, abdominal-RT, and pelvic-RT exposures, but far fewer CpG hits were associated with brain-RT exposure. This is likely due to limited exposure to the bone marrow tissue among patients who received RT to the brain, considering we measured methylation on blood-derived DNA. In this regard, the observation of a high percentage of CpGs with a dose response for RT exposures, but not for chemotherapy agents, was intriguing.

Some of the sensor CpGs were also associated with human traits such as aging, dyslipidemia, BMI, and alcohol consumption based on the annotation using EWAS catalog (Additional file 1: Table S7) [60] as well as one or more of the seven CHCs in our study, which make them eligible as potential mediators in the mediation analysis for the pathway from treatment exposures to health outcomes. Indeed, we found a range of mediation effects by multiple CpGs for the associations between treatments and CHCs. It is highly notable that we found 100% mediation for the association between abdominal-RT and hypertriglyceridemia or hypercholesterolemia. The existing literature provides strong support for the plausibility of our findings. DNAm methylation has an established role in the regulation of blood lipids and the etiology of dyslipidemia [46]. The mediator CpGs that showed statistically significant mediation effects in this study have been associated with blood lipids and related diseases in other studies and/or are in the proximity of well-established genes regulating dyslipidemia. (e.g., cg21922478 (*ITGA1*) [63], cg22976567 (*LMNA*)) [64]. The identification of key genes previously implicated in abnormal lipid metabolism in an agnostic EWAS attests to the strength of our findings.

For the partially mediated effects we discovered, other causal pathways from specific treatment to CHC are possible, including a process that indicates that DNA damage is associated with anticancer therapies and specific mechanisms for DNA repair [61, 65–68]. Our previous study show pathogenic mutations in DNA repair pathways increase the risk of developing subsequent neoplasms, especially among survivors who received high doses of radiation or specific types of chemotherapeutic agents [26].

Our study has some limitations. First, due to frequent use of multimodality therapy, delineation of independent associations was not always feasible. To identify independent hits for each treatment, EWAS for each treatment was adjusted for other treatment exposures except for specific treatments that were highly correlated. Furthermore, due to the fact that cancer treatment is determined by cancer type together with age at diagnosis and era of diagnosis, our EWAS findings of treatment-specific effects may be driven by an underlying specific cancer diagnosis. Moreover, the lack of detailed stage information for all study participants precluded adjustment for childhood cancer stage in the analysis. Second, we did not consider other factors that affect the methylation landscape such as social economic status, health behaviors, and environmental exposures which could confound the findings. Third, even though we considered temporality among treatment exposures, DNAm (measured at a single time point), and incidence of CHCs, we could not definitively infer causality among three entities. Fourth, our study focused on European ancestry survivors of childhood cancer. Further replication with larger and more diverse survivor populations and validation to confirm generalizability to other ethnicities are needed to confirm the role of DNAm in associations between cancer treatments and adverse health outcomes. Lastly, the other limitation of the study is that we did not consider co-morbidity in the analysis of the association between DNAm and cardiometabolic conditions; therefore, when each specific condition was analyzed, survivors also had a range of other CHCs that could impact methylation.

## Conclusions

In summary, we identified thousands of CpG sites associated with specific cancer treatments at genome-wide significant levels, suggesting that DNAm is an important biological embedding mechanism for prior cancer treatment exposures. We observed hundreds of these treatment-associated CpG sites significantly associated with one or more of the seven cardiometabolic CHC risks after adjusting for multiple testing. Moreover, dozens of these sensor CpG sites showed full or partial mediation effects for the association between specific treatment exposure and cardiometabolic CHC, suggesting DNAm, as a biomarker, can be used as a risk predictor and potential mechanistic target for future intervention studies among survivors of childhood cancer. Our study has limitations that require cautious interpretation of the results presented. Future studies are warranted to further validate and replicate these findings.

## Supplementary Information

**Additional file 1: Supplementary Methods.** Bisulfite treatment, array hybridization, and scanning for DNA Methylation Profiling. **Fig. S1.** A flow diagram of the study population. **Fig. S2.** Q-Q plot showing the distribution of the observed versus expected association *p*-values. **Fig. S3.** Manhattan plot showing the treatment-specific association of DNA methylation. **Fig. S4.** The distributions of whole-array and significant treatment-associated CpG sites. **Fig. S5.** The distribution of beta-values for CpG mediators by groups of CHCs. **Table S1.** Pairwise correlations among eleven different treatments. **Table S2**. CpG hits associated with the specific treatment exposure (*P*<9x10^-8^). **Table S3.** 276 additional CpG hits associated with 9 paired combinations of treatments (*P*<9x10^-8^). **Table S4.** GO biological process annotations of specific treatment-associated CpG sites. **Table S5.** Association of treatment-associated methylation sites with CHCs (*P*_*FDR*_<0.05). **Table S6.** Multivariable associations of treatment exposures with CHCs (*P*<0.05). **Table S7.** Previously published associations of blood-based DNA methylation on CpG sites with health conditions.)

## Data Availability

The DNA methylation data, demographics, and cancer treatment information generated and analyzed in this study are accessible at NCBI Gene Expression Omnibus database under the accession number GSE169156 https://www.ncbi.nlm.nih.gov/geo/query/acc.cgi?acc=GSE169156 [69]. Additional clinical data about the St. Jude Lifetime Cohort Study can be accessed via the survivorship portal hosted on the St. Jude cloud (http://survivorship.stjude.cloud/).

## Abbreviations

ACME: Average causal mediation effects
AGM: Abnormal glucose metabolism
CHC: Chronic health conditions
CI: Confidence interval
CNS: Central nervous system
CpG: 5′-Cytosine-phosphate-guanine-3′
EWAS: Epigenome-wide association study
FDR: False discovery rate
HCL: Hypercholesterolemia
HTG: Hypertriglyceridemia
IQR: Interquartile range
OR: Odds ratio
PNET: Primitive neuroectodermal tumor
RT: Radiotherapy
SJLIFE: St. Jude Lifetime Cohort Study
