# Supplementary material for: Persistent variations of blood DNA methylation associated with treatment exposures and risk for cardiometabolic outcomes in long-term survivors of childhood cancer in the St. Jude Lifetime Cohort
Source: Genome Med. 2021 Apr 6;13:53. doi: 10.1186/s13073-021-00875-1 (PMC8025387; doi:10.1186/s13073-021-00875-1)
Supplement: Supplementary file 1 — Additional file 1: Supplementary Methods. Bisulfite treatment, array hybridization, and scanning for DNA Methylation Profiling. Fig. S1. A flow diagram of the study population. Fig. S2. Q-Q plot showing the distribution of the observed versus expected association p-values. Fig. S3. Manhattan plot showing the treatment-specific association of DNA methylation. Fig. S4. The distributions of whole-array and significant treatment-associated CpG sites. Fig. S5. The distribution of beta-values for CpG mediators by groups of CHCs. Table S1. Pairwise correlations among eleven different treatments. Table S2. CpG hits associated with the specific treatment exposure (P<9x10^-8^). Table S3. 276 additional CpG hits associated with 9 paired combinations of treatments (P<9x10^-8^). Table S4. GO biological process annotations of specific treatment-associated CpG sites. Table S5. Association of treatment-associated methylation sites with CHCs (PFDR<0.05). Table S6. Multivariable associations of treatment exposures with CHCs (P<0.05). Table S7. Previously published associations of blood-based DNA methylation on CpG sites with health conditions.) [file 13073_2021_875_MOESM1_ESM.docx]

**Supplementary Methods**

***Bisulfite treatment, array hybridization, and scanning for DNA Methylation Profiling***

Genomic DNA (250 ng per sample) were treated with bisulfite using the Zymo EZ DNA Methylation Kit under the following thermos-cycling conditions: 16 cycles: 95 degrees Celsius for 30 sec, 50 degrees Celsius for 1 hour. Following bisulfite treatment, DNA samples were desulphonated, column purified, then eluted using 12 µl of elution buffer (Zymo Research Corp.). Treating DNA with sodium bisulfite selectively converts cytosine to uracil but preserves 5-methylcytosine, which is immune from deamination. Bisulfite-converted DNA (4 µl) was then processed by following the Illumina Infinium Methylation Assay protocol, which included hybridization to MethylationEPIC BeadChips, single base extension assay, and staining and scanning using the Illumina HiScan system.

***Radiation doses***

The radiation doses represent the maximum treatment dose (cGy) to any portion of a body region (~4 to 10 cm depth). For other-head, neck, chest, abdomen, pelvis or limbs, if >10% to 100% of the region is in a direct beam then the region is considered in the treatment field. For brain treatment at least 50% of the segment must be in the primary beam to be considered in a treatment field. Regions that received only stray (scatter and leakage) radiation are designated as stray high (SH) if the region was adjacent to a region receiving direct radiation or as stray low (SL) if the region is not adjacent to any region receiving direct radiation treatment. Laterality (left, right, or central) of treatment is not provided.

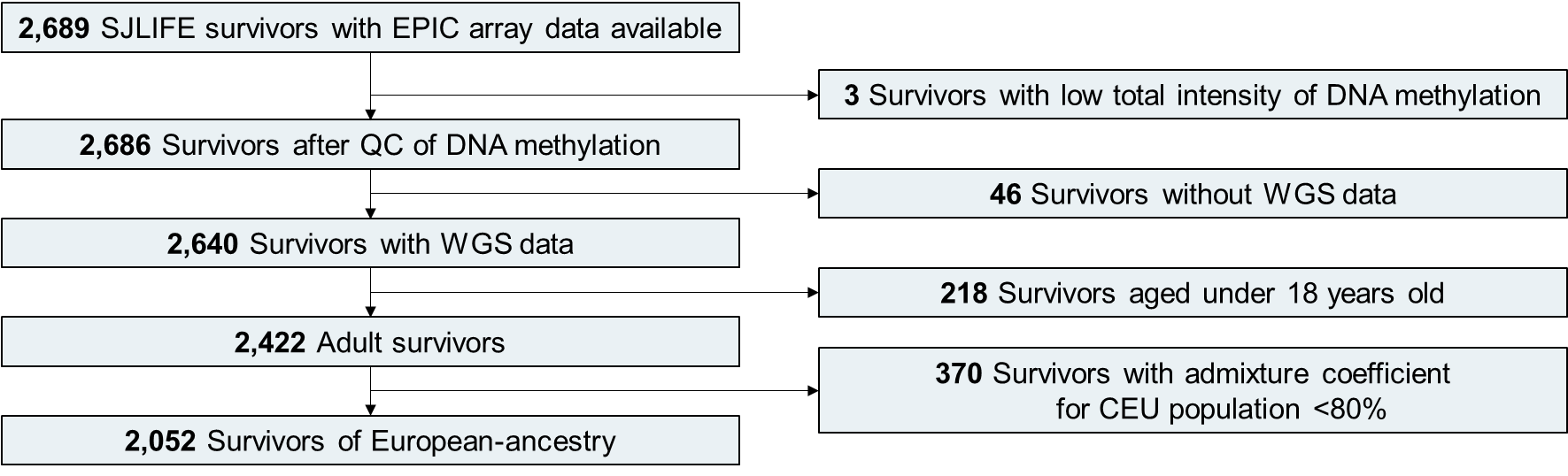


**Fig. S1. A flow diagram of the study population**

Abbreviations: SJLIFE (St. Jude Lifetime Cohort Study), QC (quality control), and WGS (whole-genome sequencing)

**
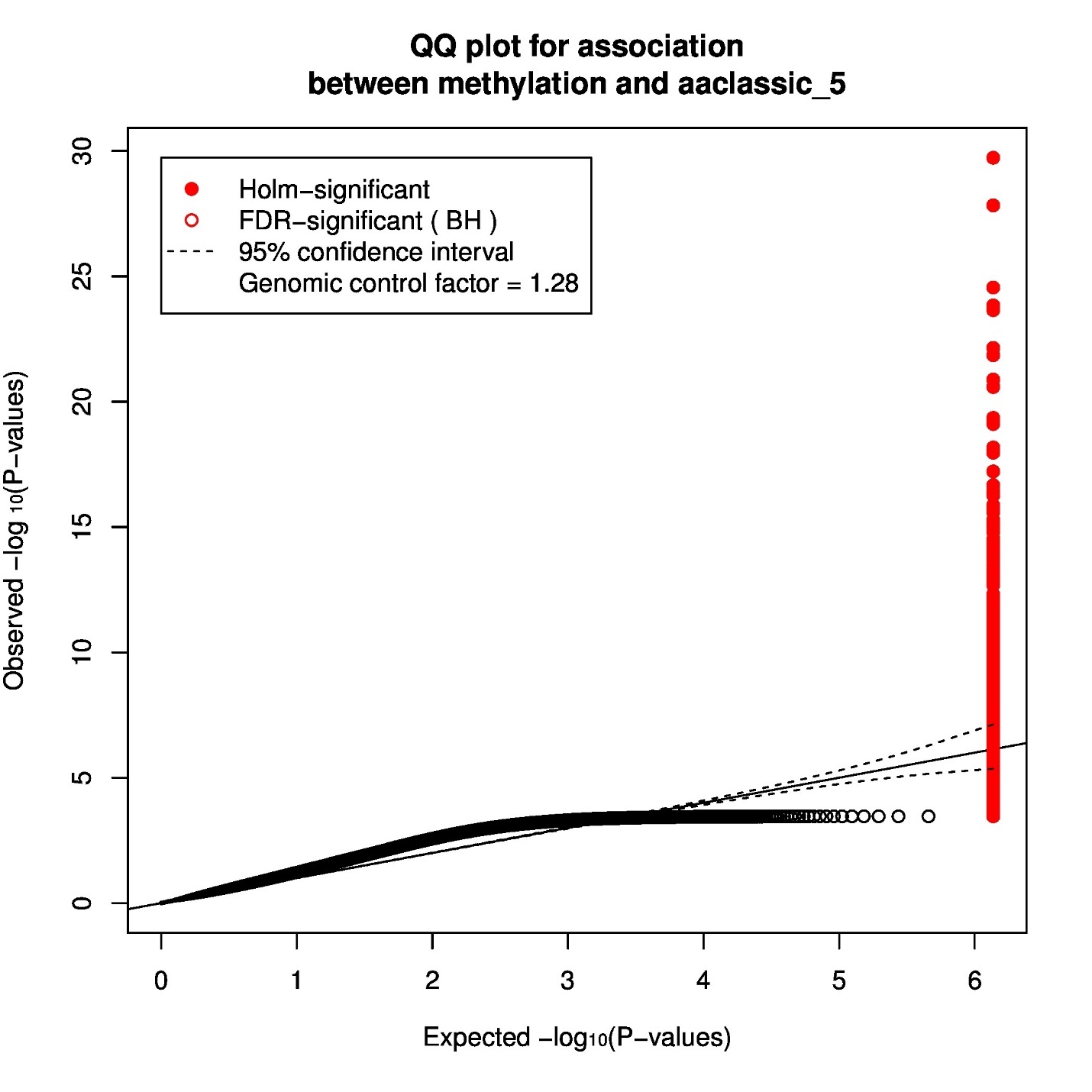
**

**(A) Alkylating agents, classic**

**
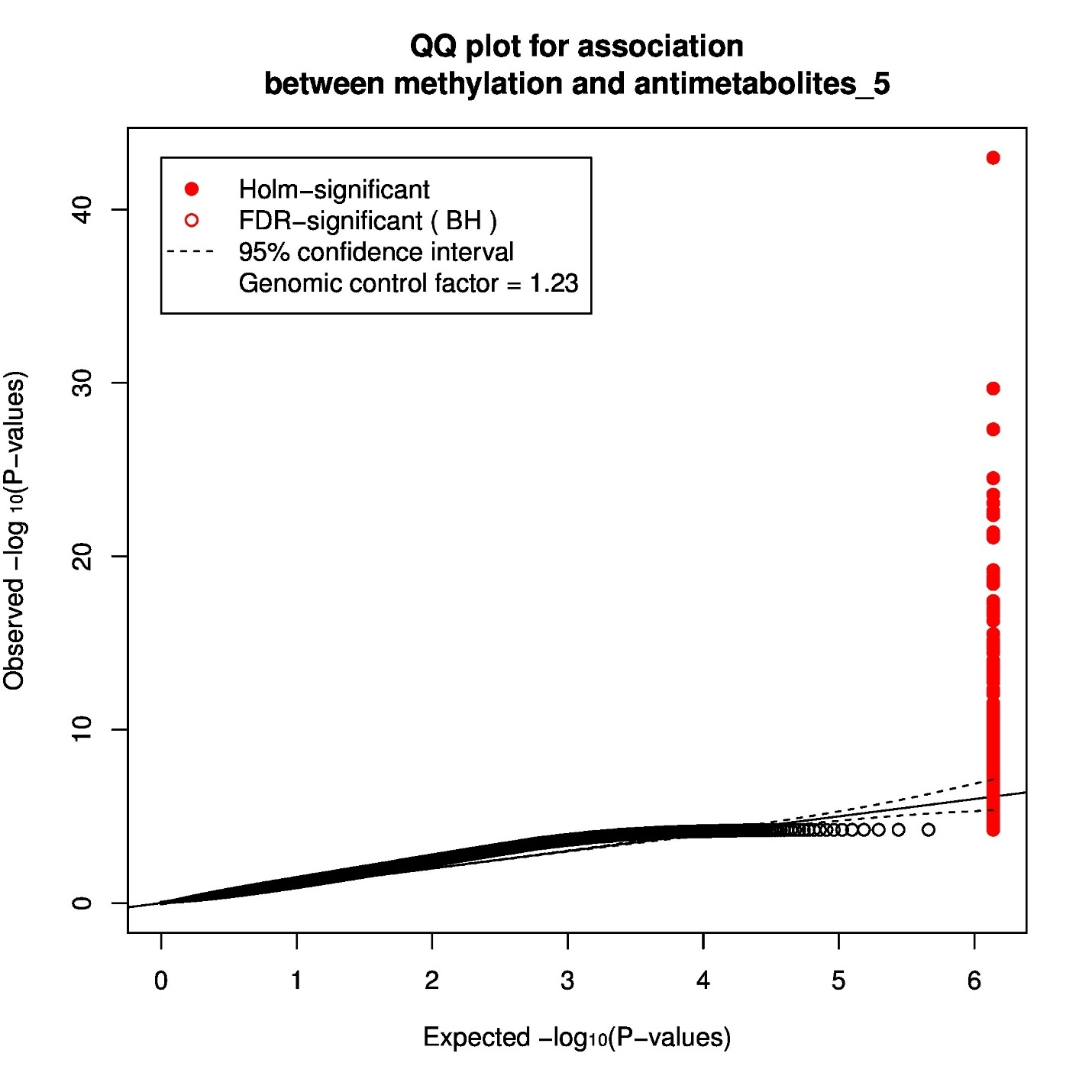
**

**(B) Anti-metabolites**

**
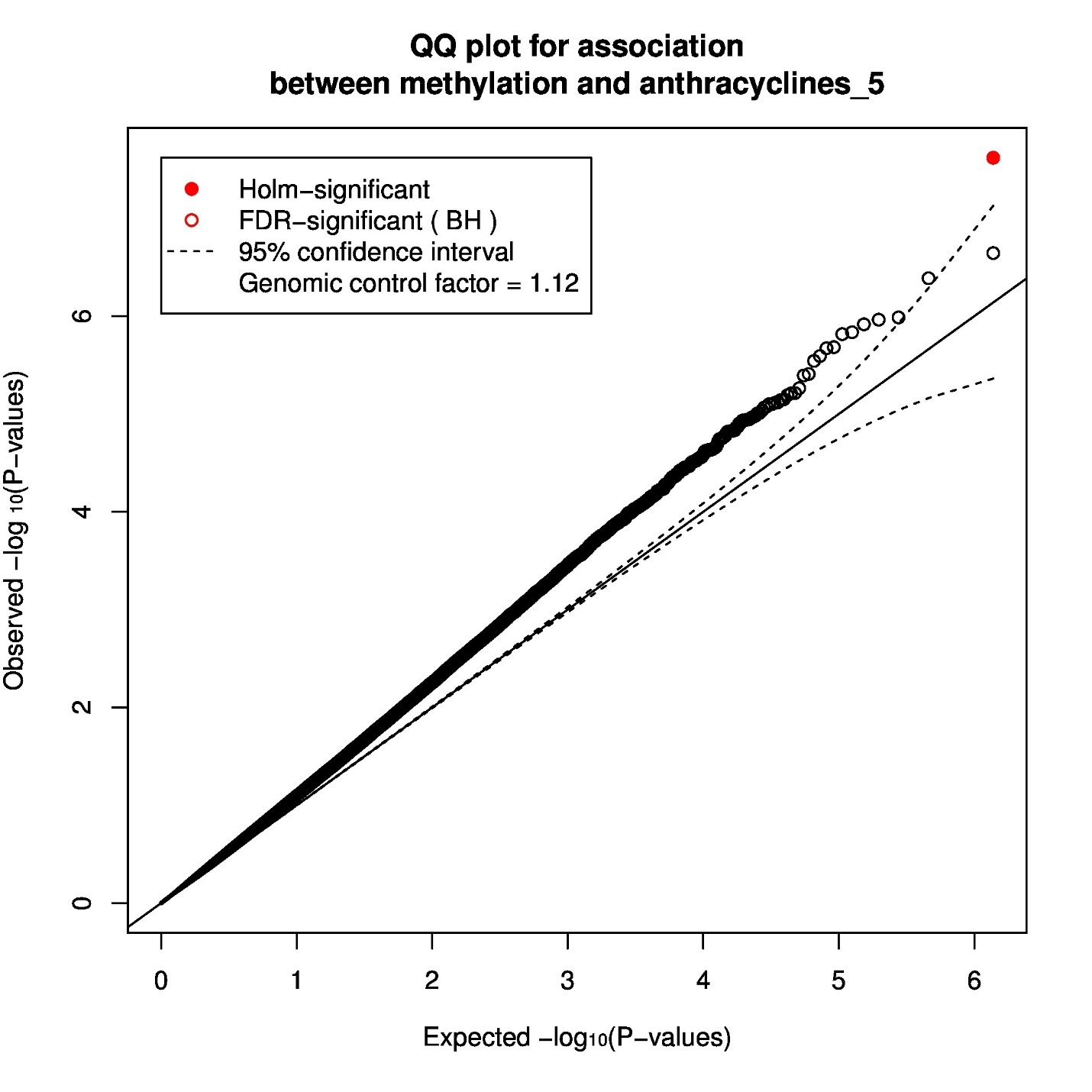
**

**(C) Anthracyclines**

**
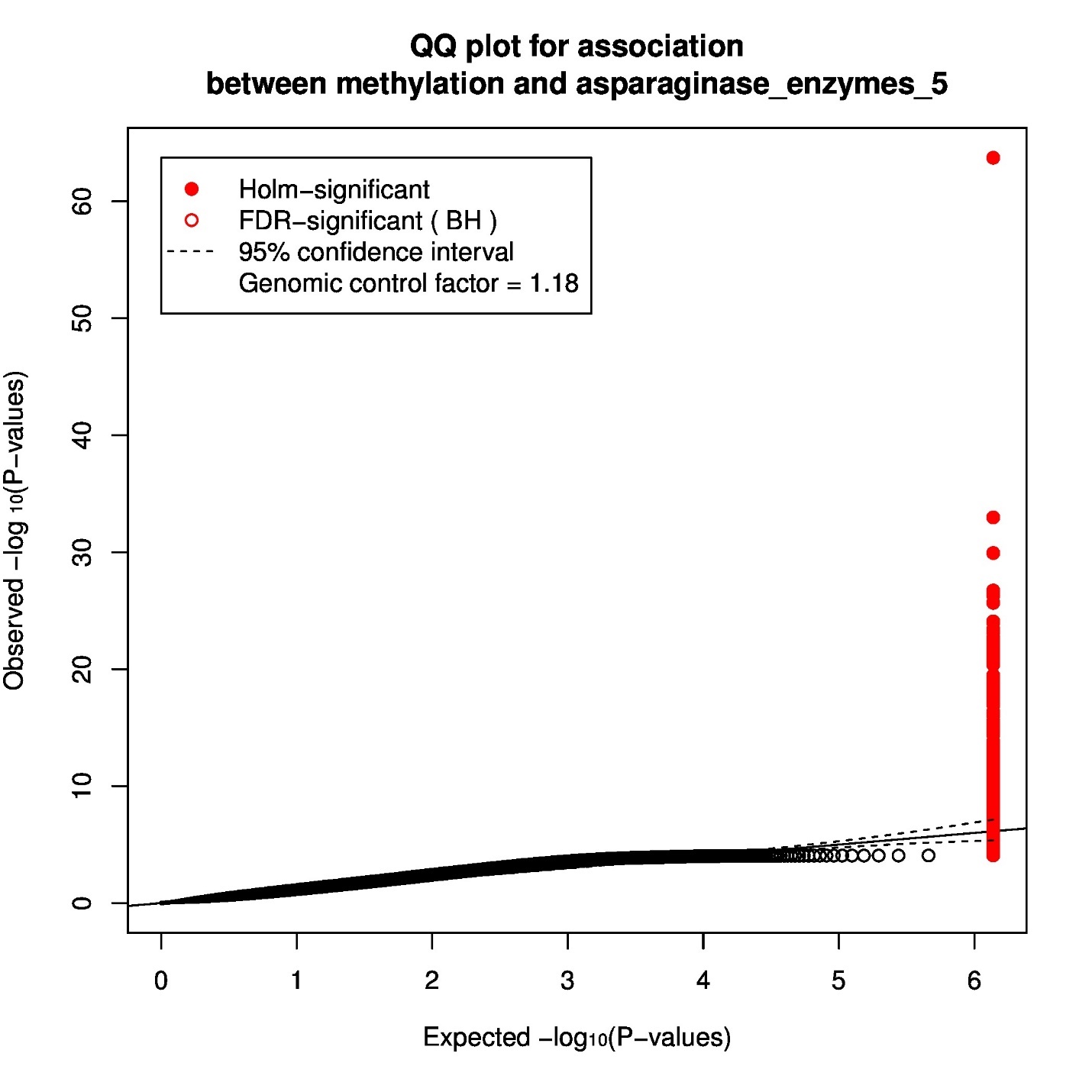
**

**(D) Asparaginase enzymes**

**
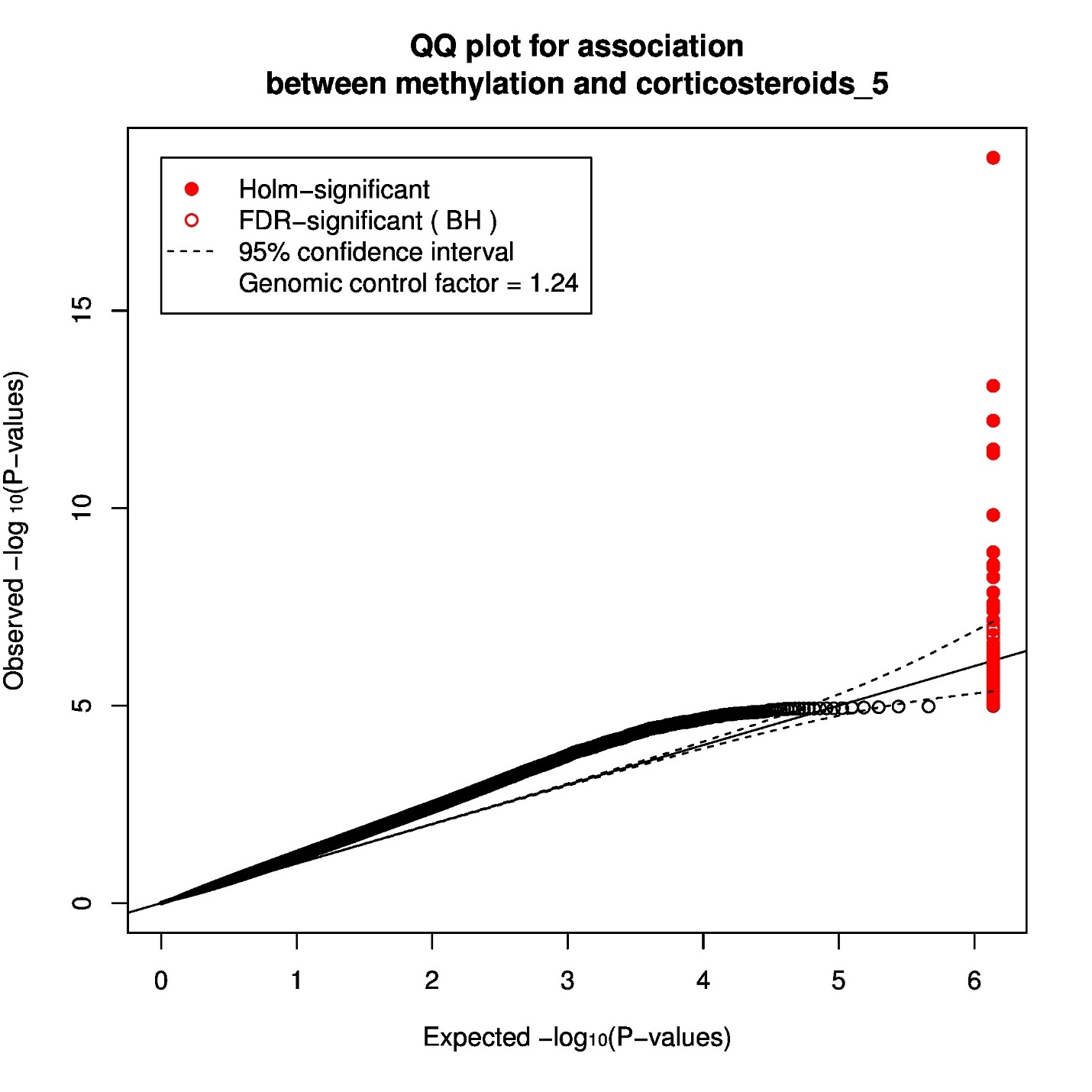
**

**(E) Corticosteroids**

**
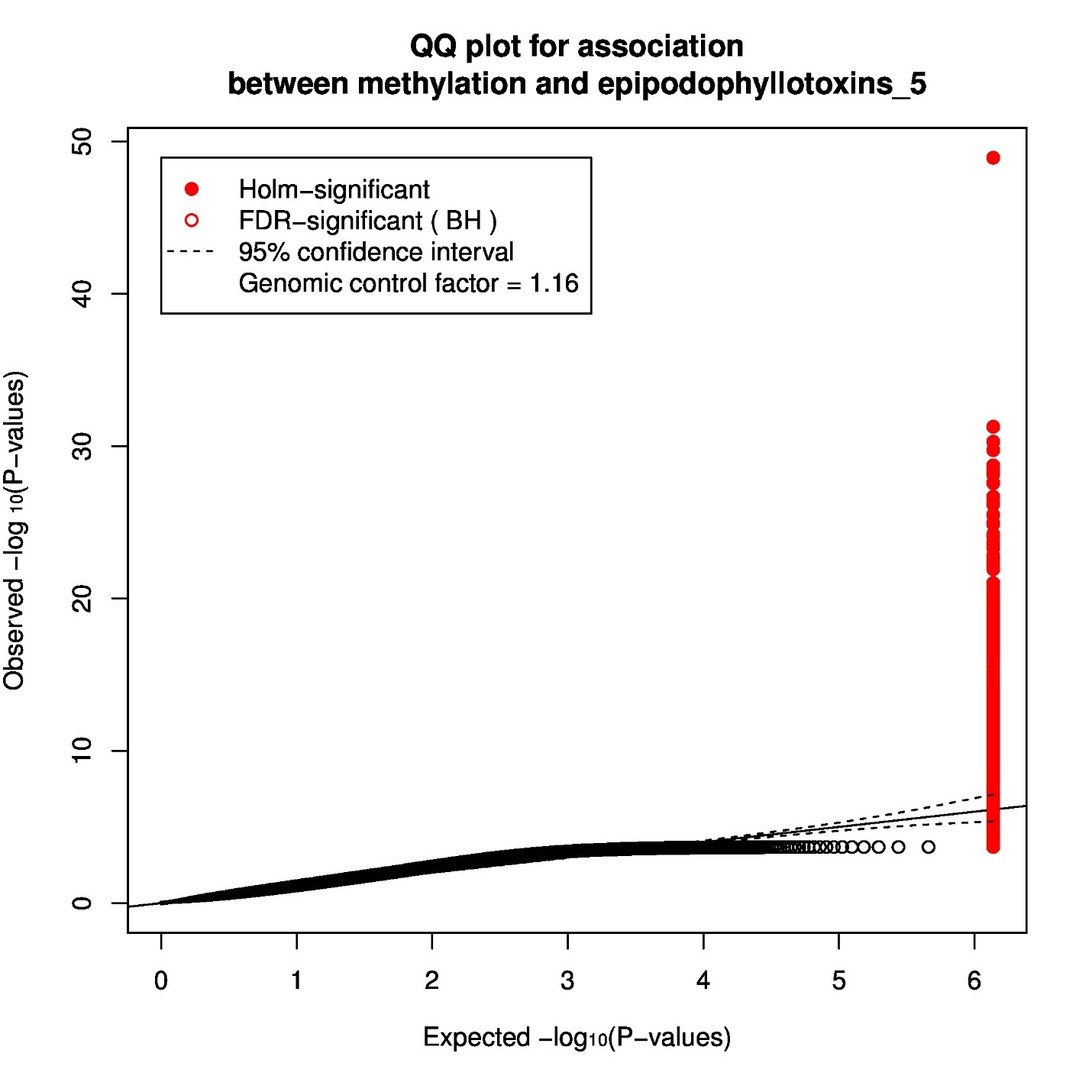
**

**(F) Epipodophyllotoxins**

**
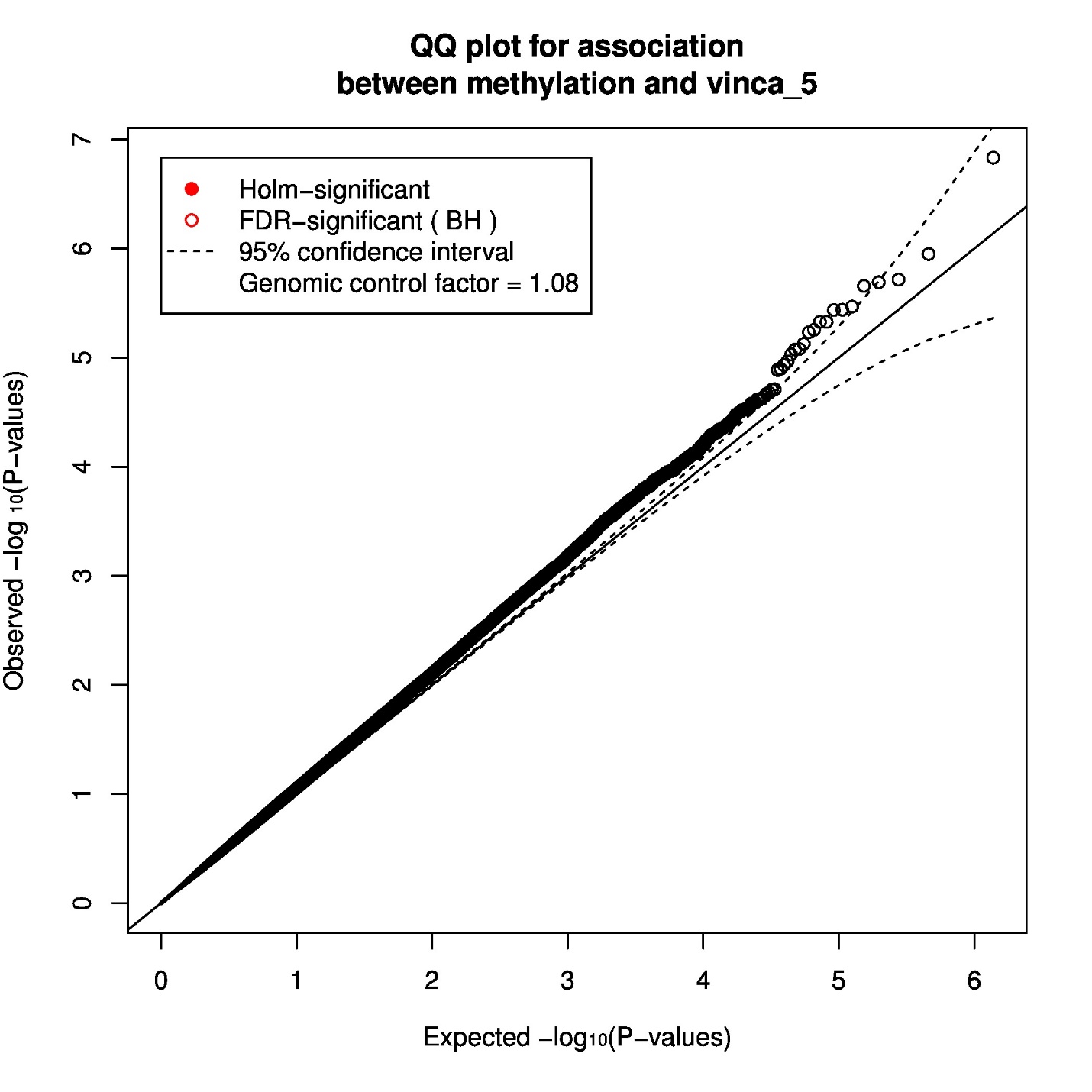
**

**(G) Vinca alkaloids**

**
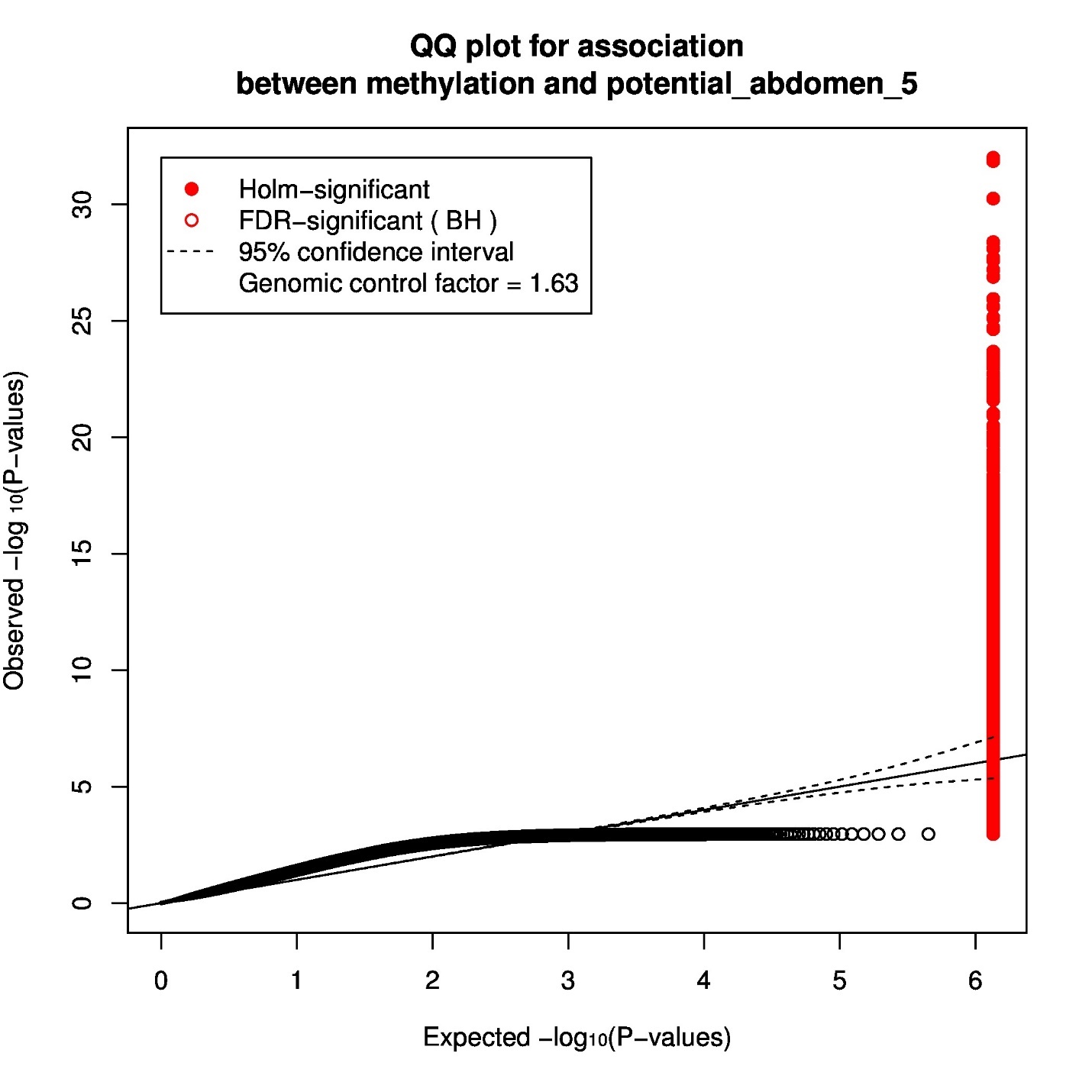
**

**(H) Abdomen-RT**

**
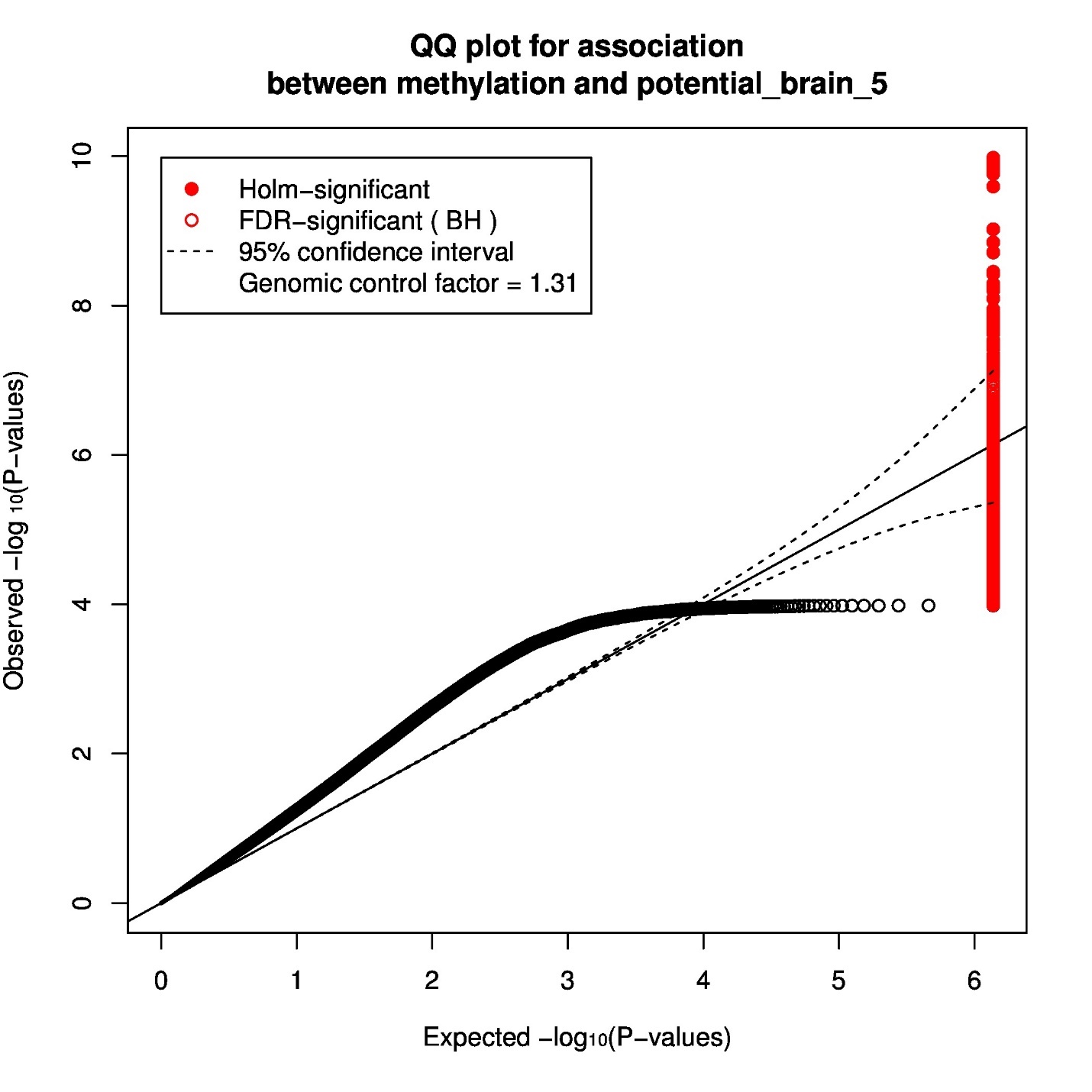
**

**(I) Brain-RT**

**
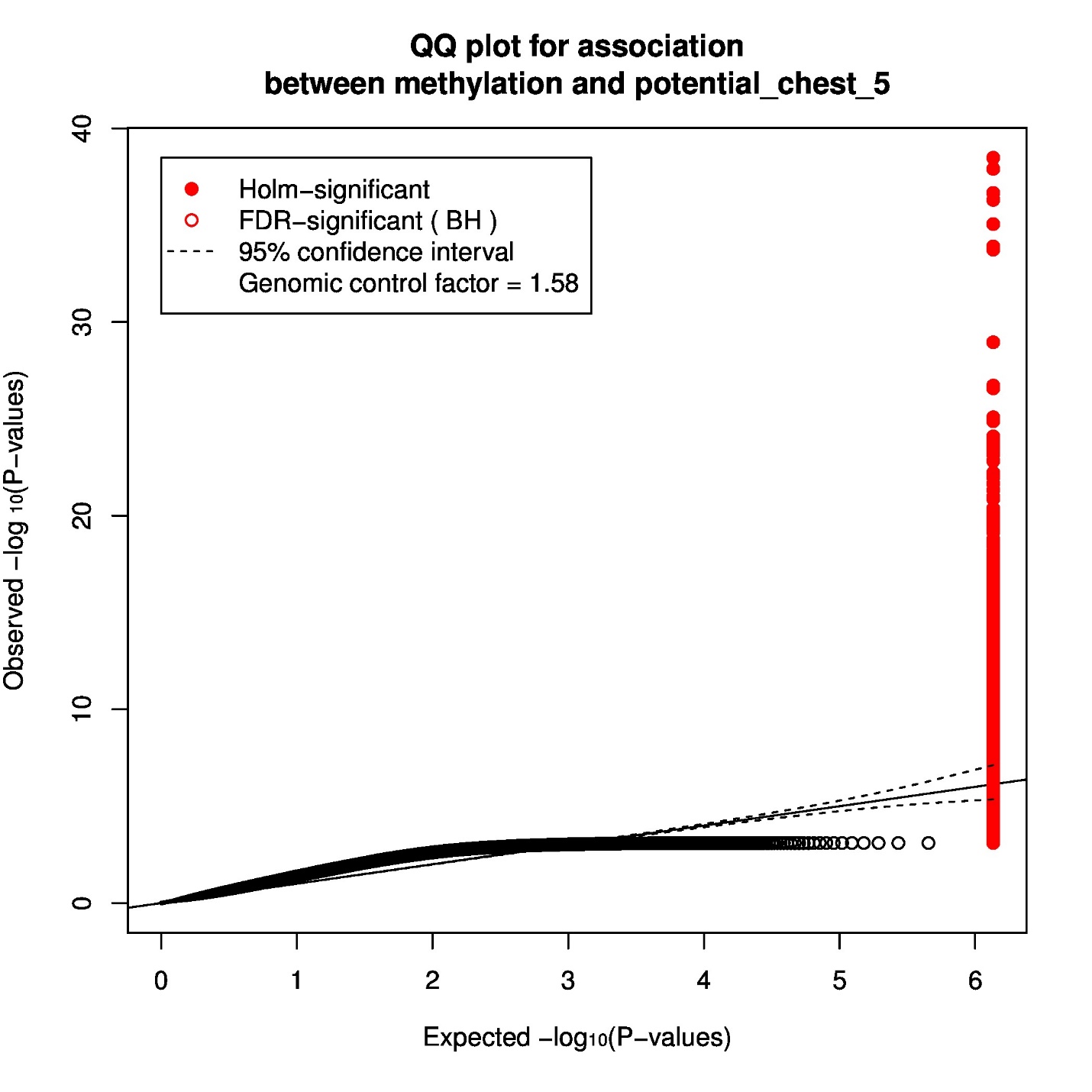
**

**(J) Chest-RT**

**
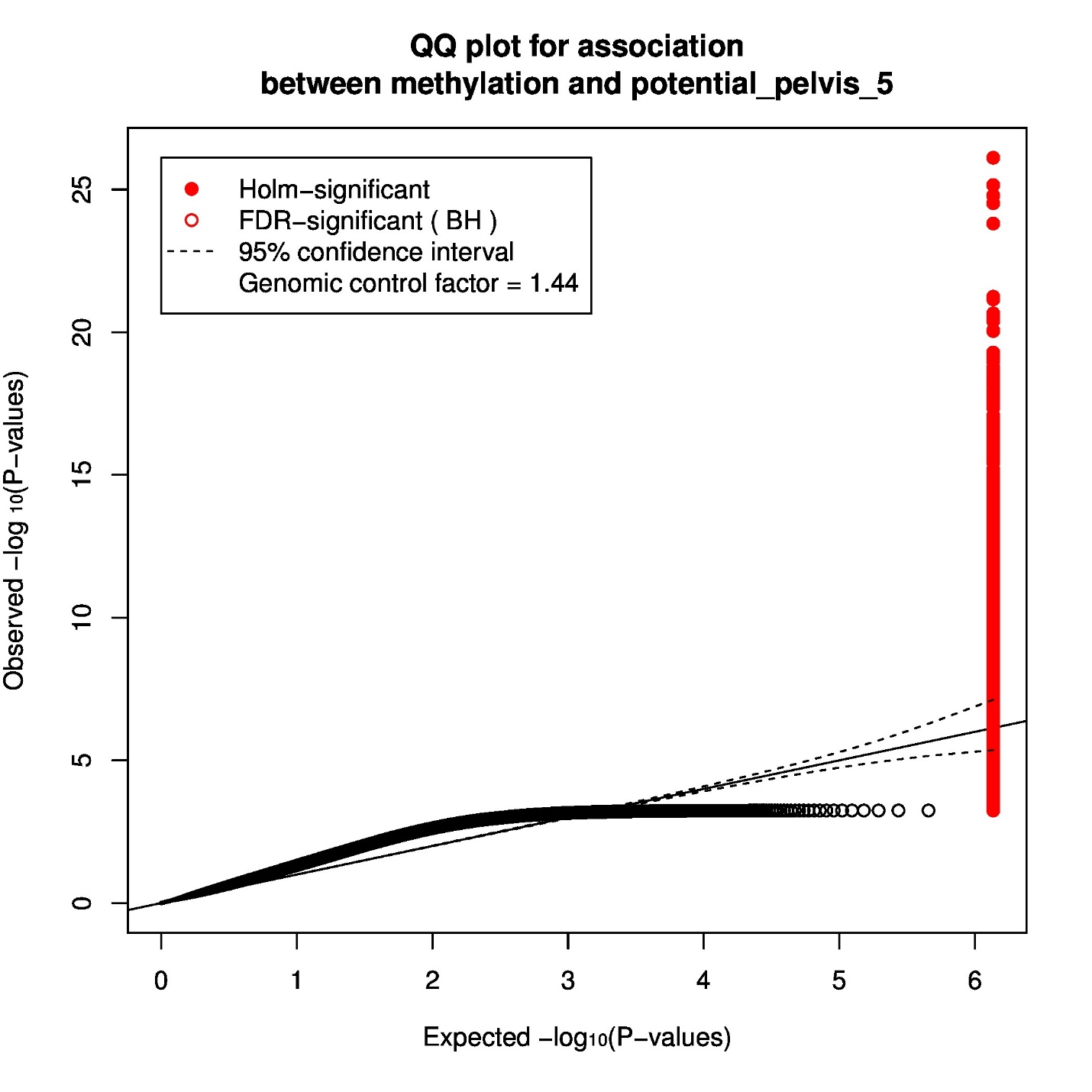
**

**(K) Pelvis-RT**

**Fig. S2. Q-Q plot showing the distribution of the observed versus expected association *p*-values.**

**
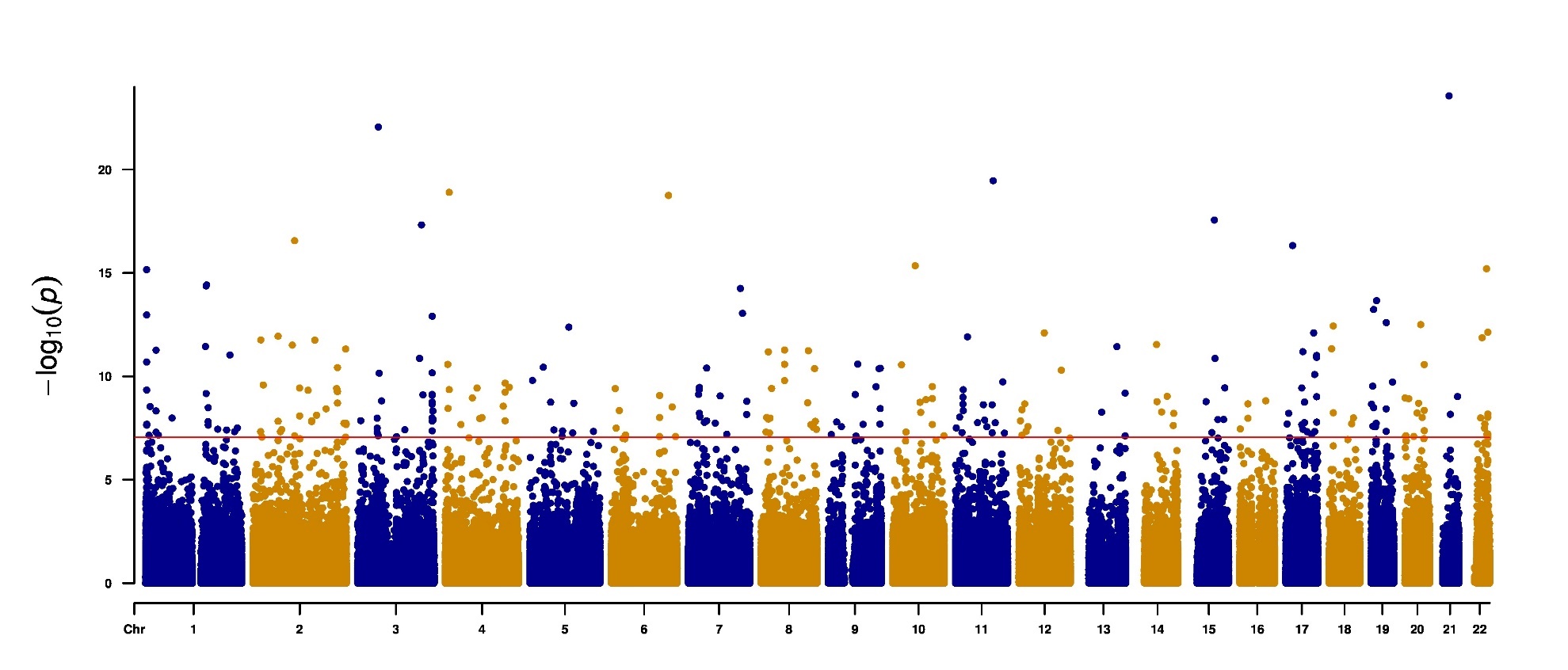
**

**(A) Alkylating agents, classic**

**
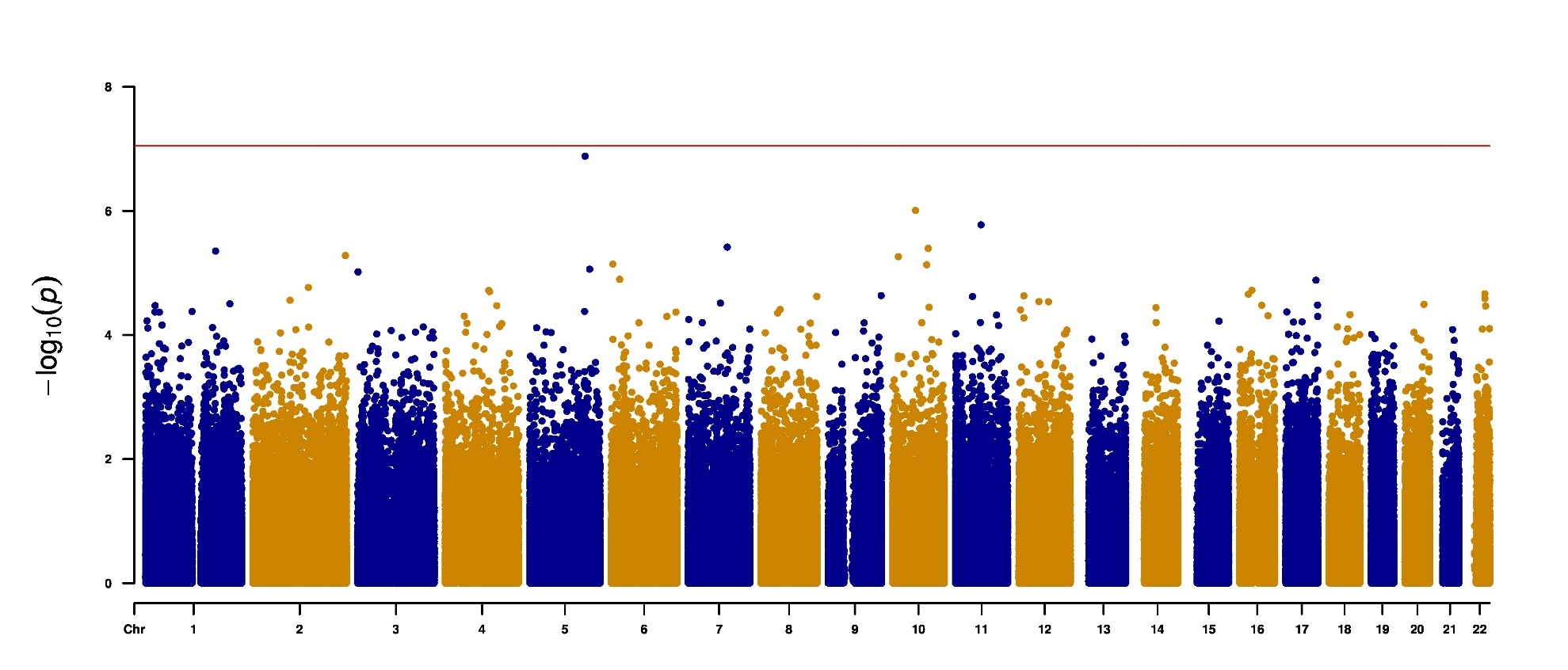
**

**(B) Anthracyclines**

**
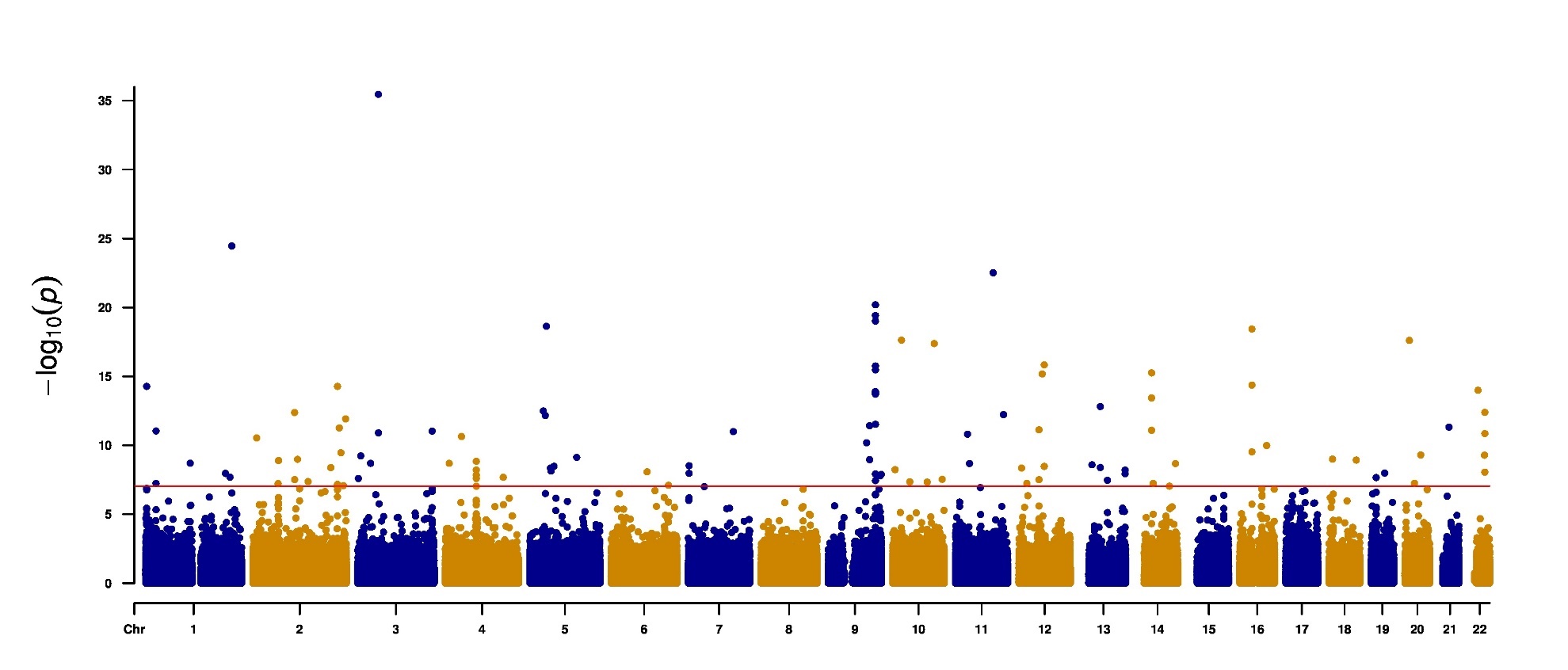
**

**(C) Anti-metabolites**

**
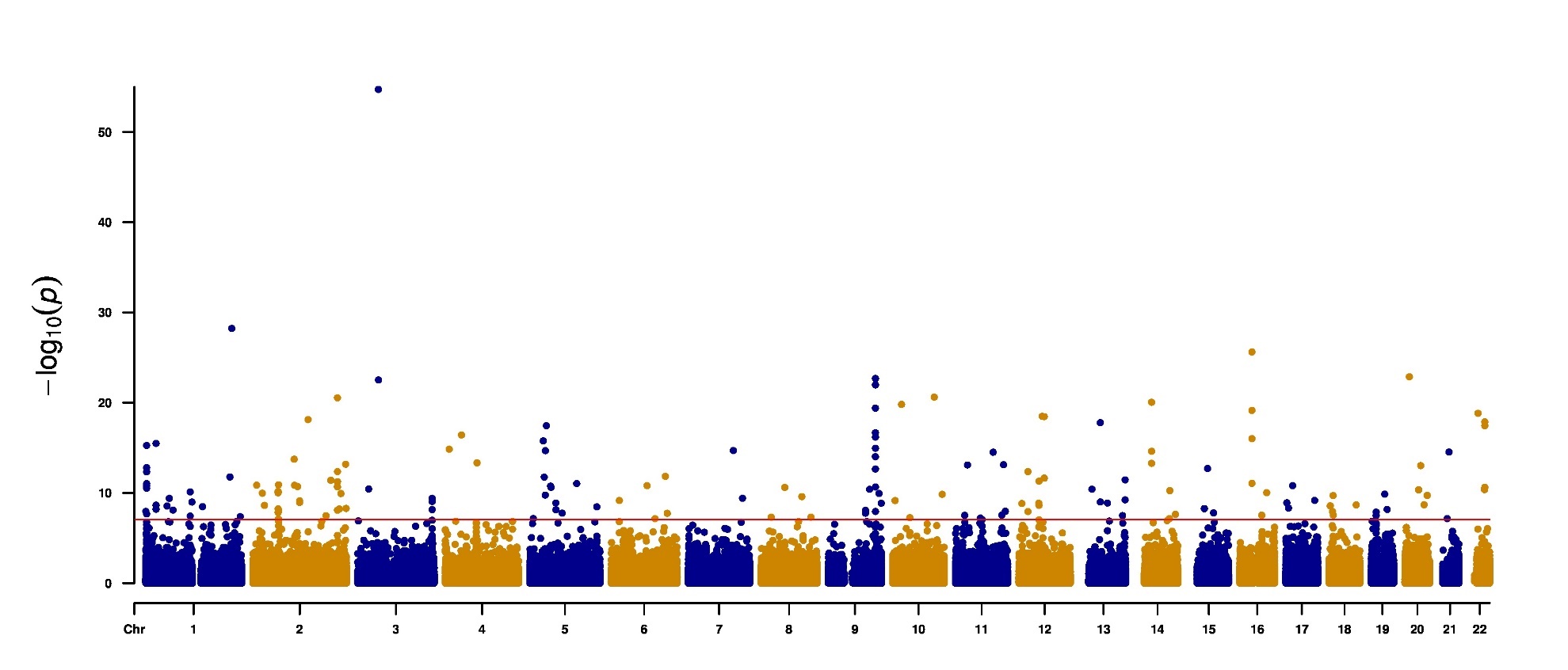
**

**(D) Asparaginase enzymes**

**
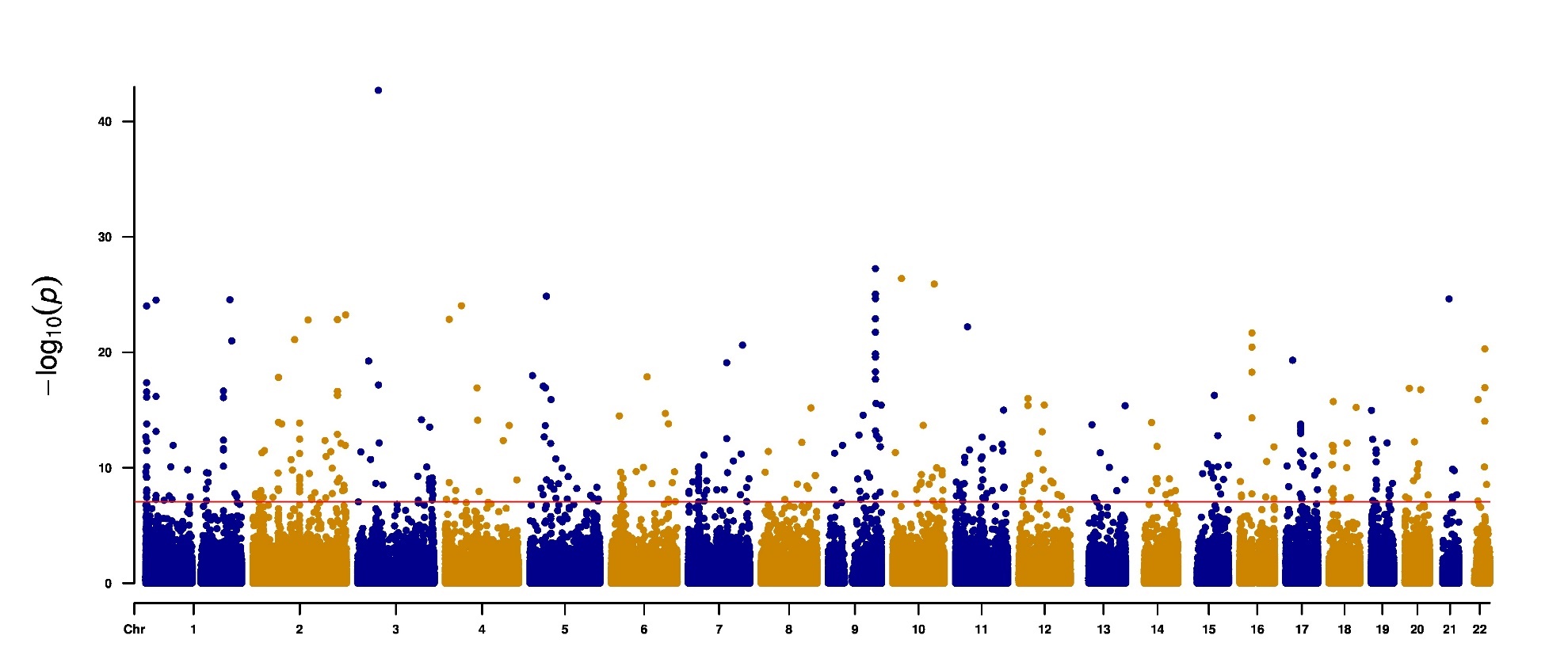
**

**(E) Epipodophyllotoxins**

**
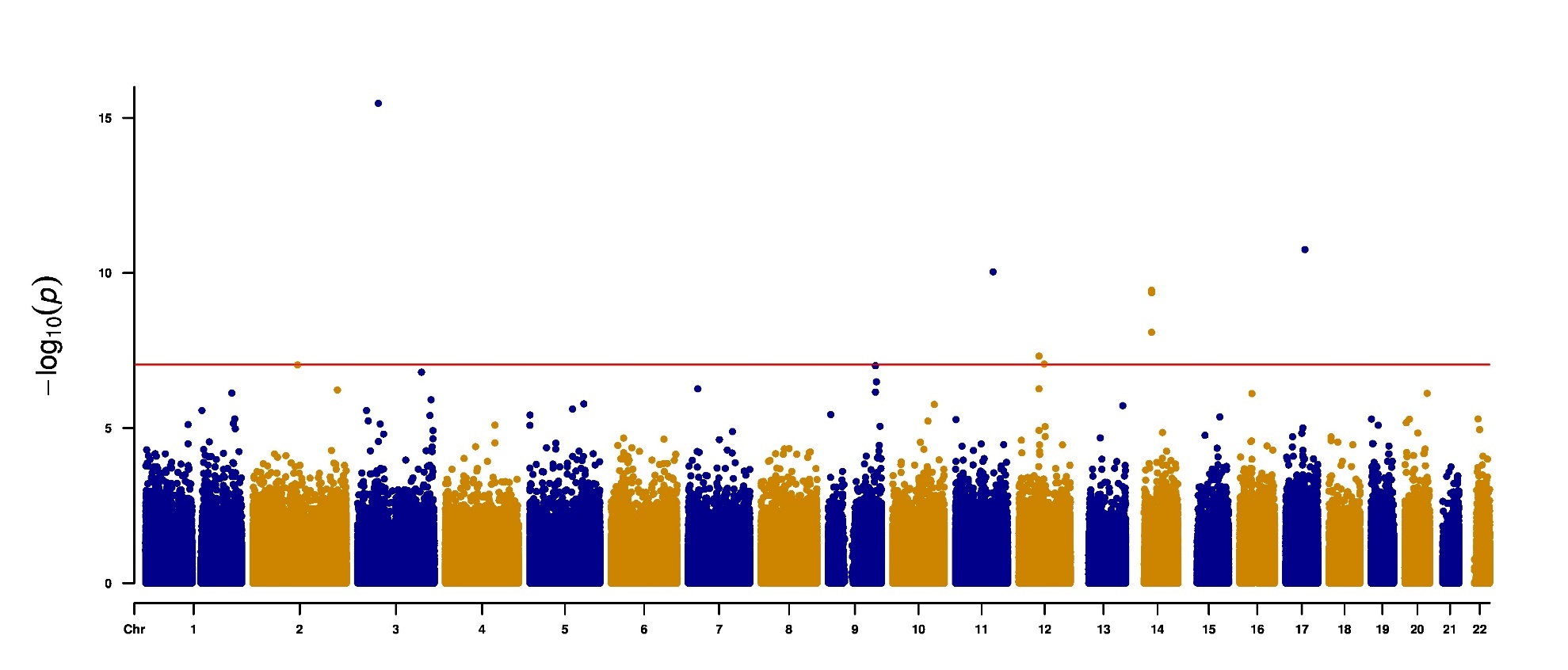
**

**(F) Corticosteroids**

**
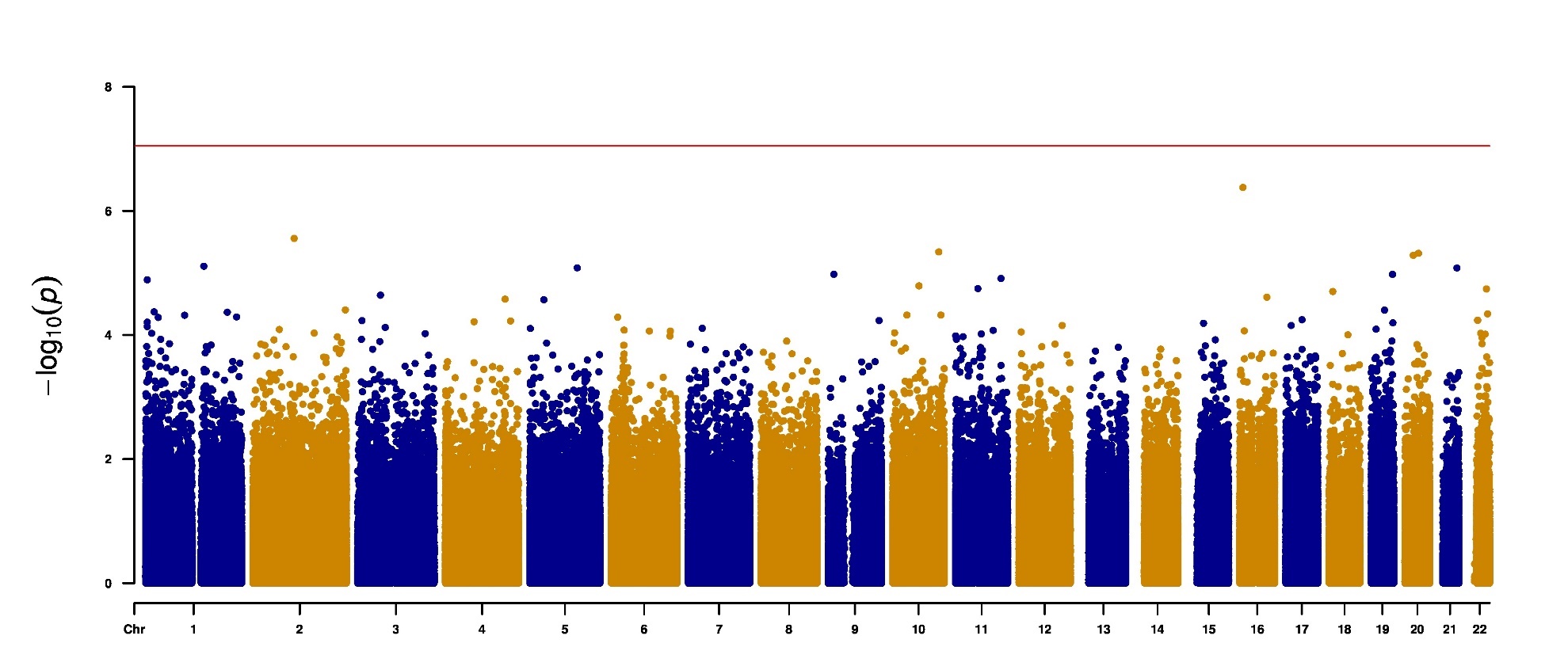
**

**(G) Vinca alkaloids**

**
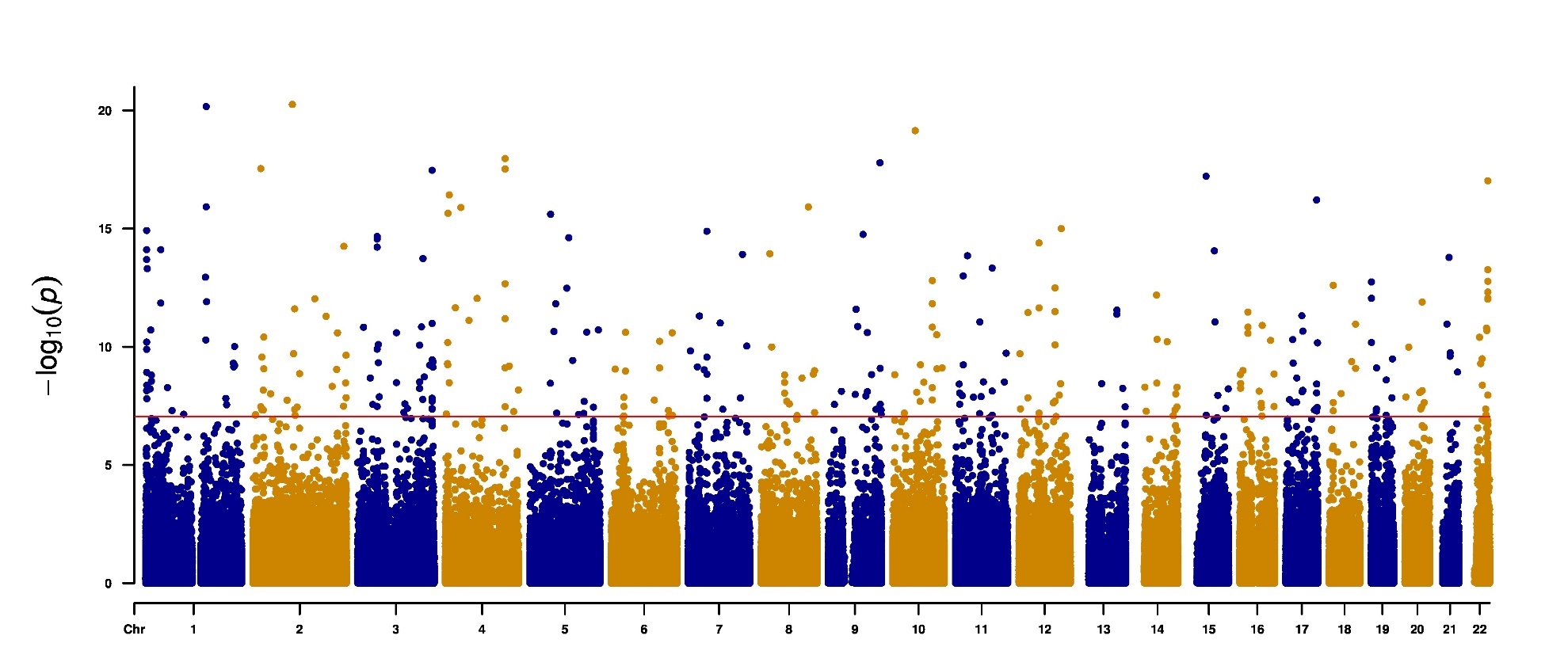
**

**(H) Abdomen-RT**

**
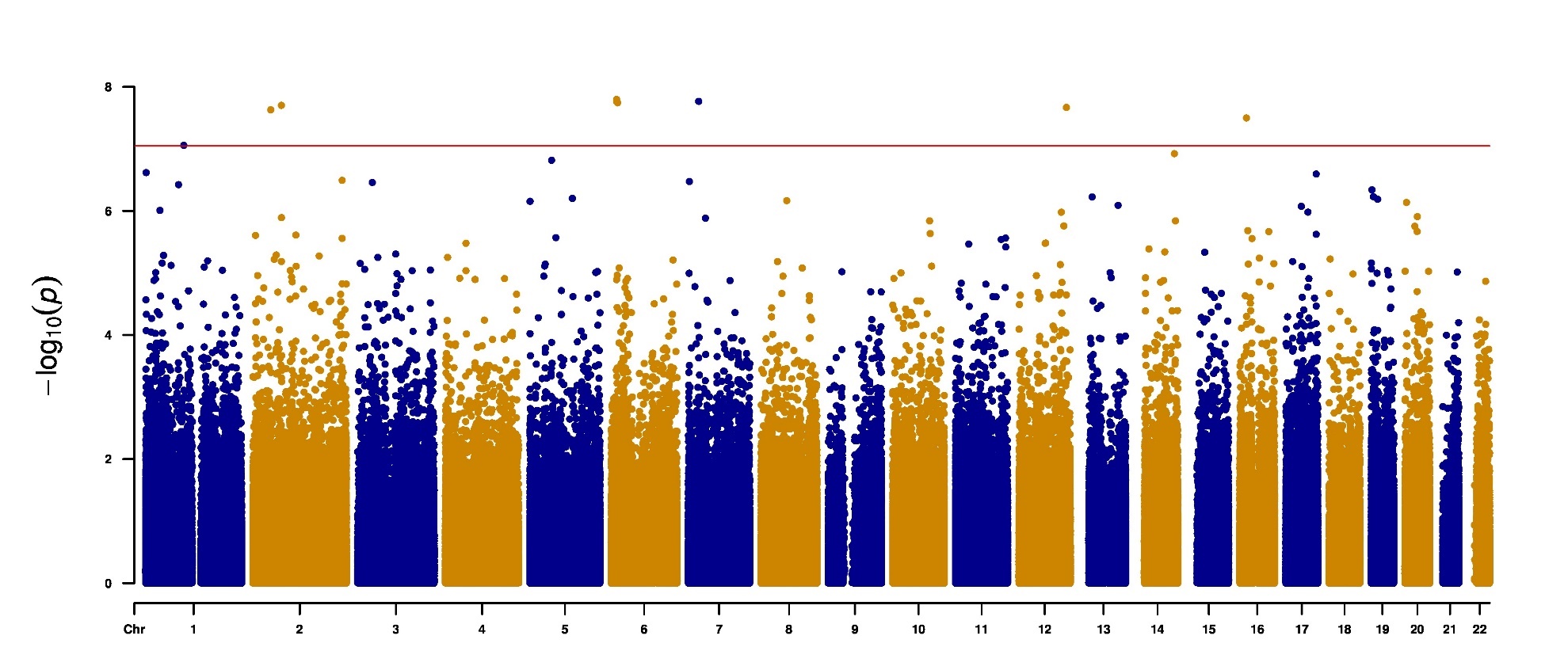
**

**(I) Brain-RT**

**
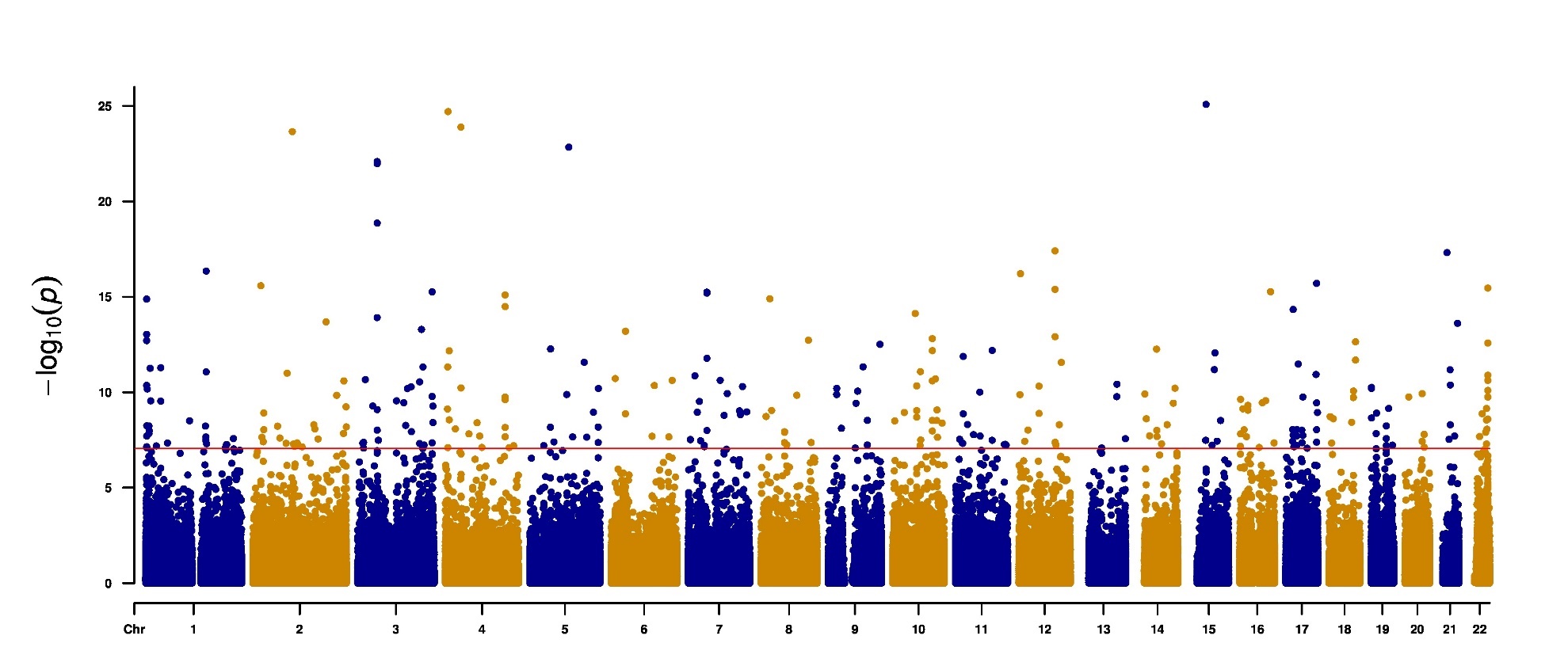
**

**(J) Chest-RT**

**
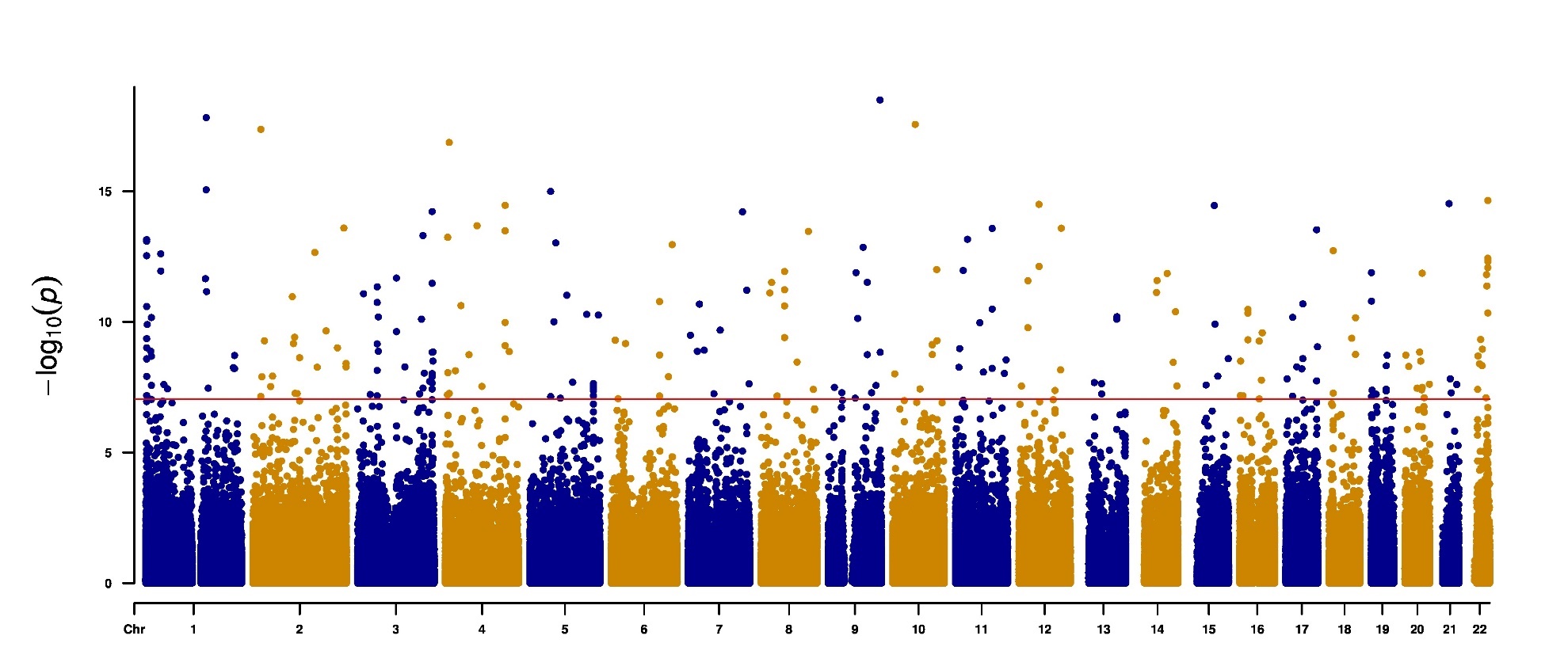
**

**(K) Pelvis-RT**

**Fig. S3. Manhattan plot showing the treatment-specific association of DNA methylation.**

**
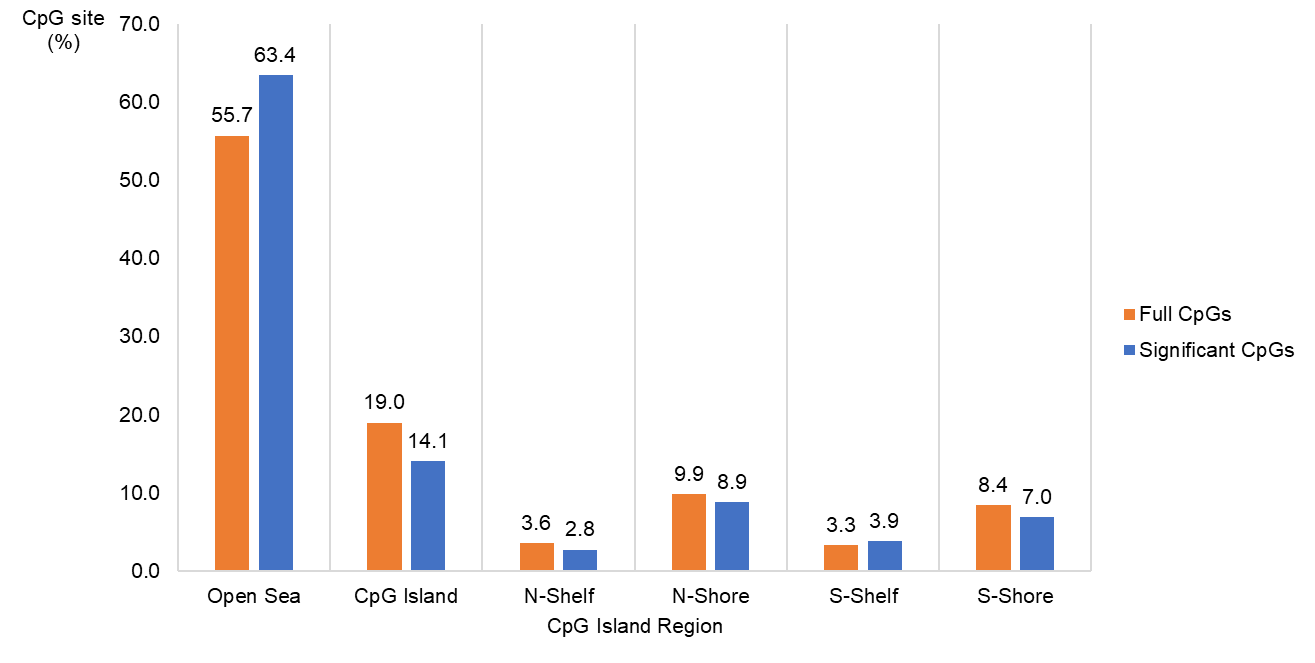
**

**(A) CpG distributions across CpG island regions.** Chi-square tests suggest significant differences for open sea (*p*<0.00001) and CpG island (*p*=0.001), and nonsignificant differences for N-shelf (*p*=0.18), N-shore (*p*=0.30), S-Shelf (*p*=0.38) and S-Shore (*p*=0.10).

**
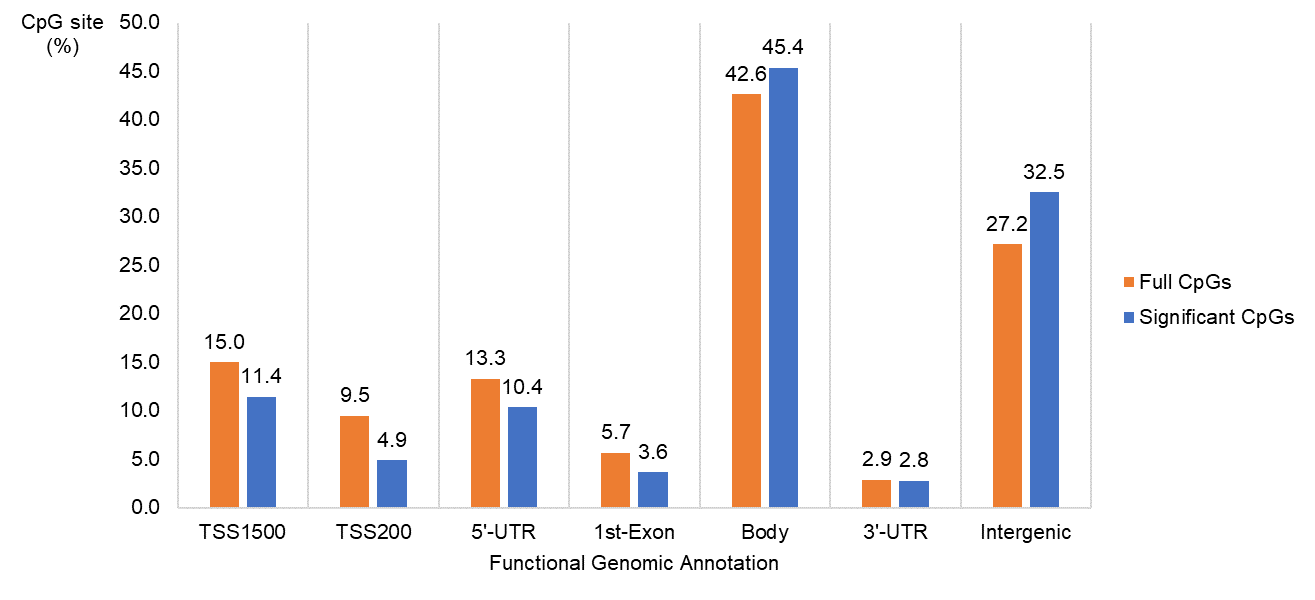
**

**(B) CpG distributions across different functional genomic annotations.** Chi-square tests suggest significant differences for TSS1500 (*p*=0.002), TSS200 (*p*<0.00001), 5’-UTR (*p*=0.009), 1^st^ exon (*p*=0.008) and intergenic (*p*=0.0003), and nonsignificant differences for gene body (*p*=0.09) and 3’-UTR (*p*=0.86).

**Fig. S4. The distributions of whole-array and significant treatment-associated CpG sites**

**
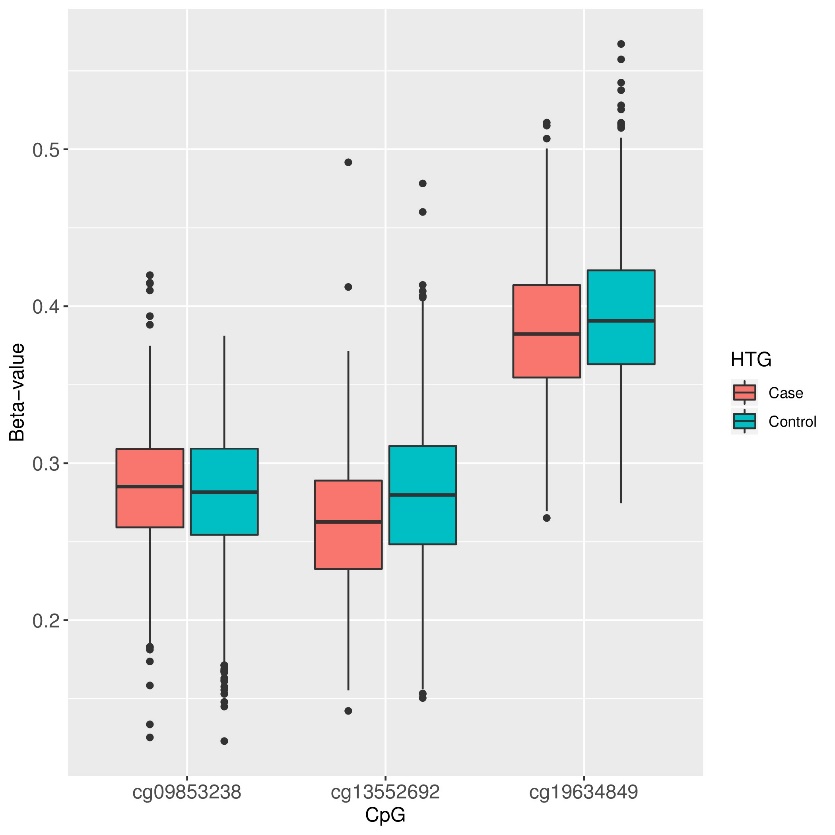
**

**(A) CpG mediators of abdomen-RT and HTG.** P values from T-tests were 0.24, <0.0001 and 0.0014 for cg09853238, cg13552692 and cg19634849, respectively.

**
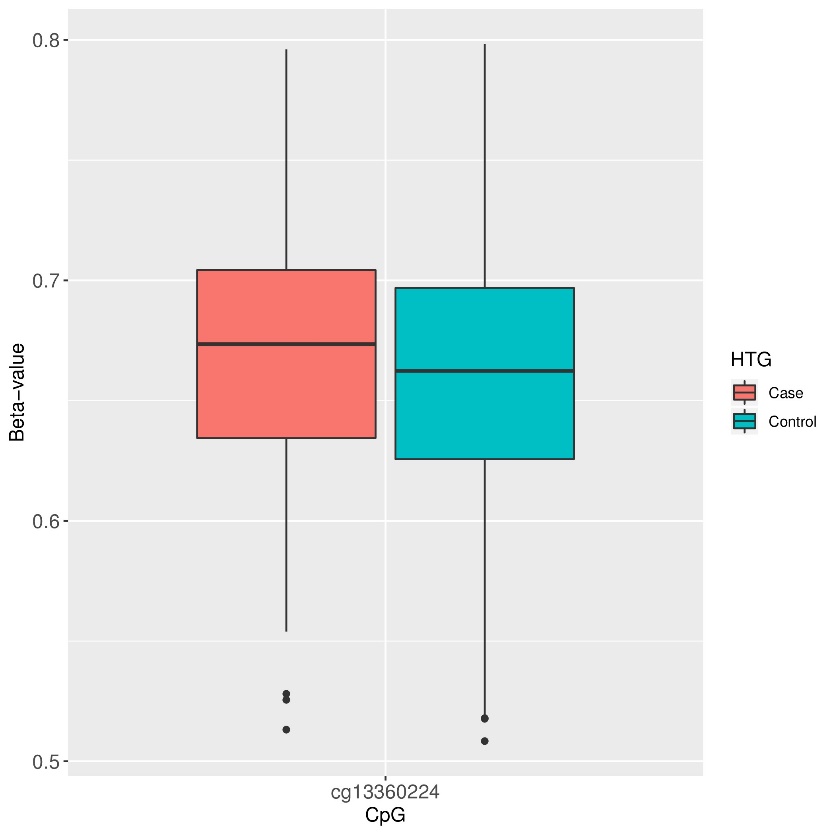
**

**(B) CpG mediators of brain-RT and HTG.** P value from T-test was 0.0018

**
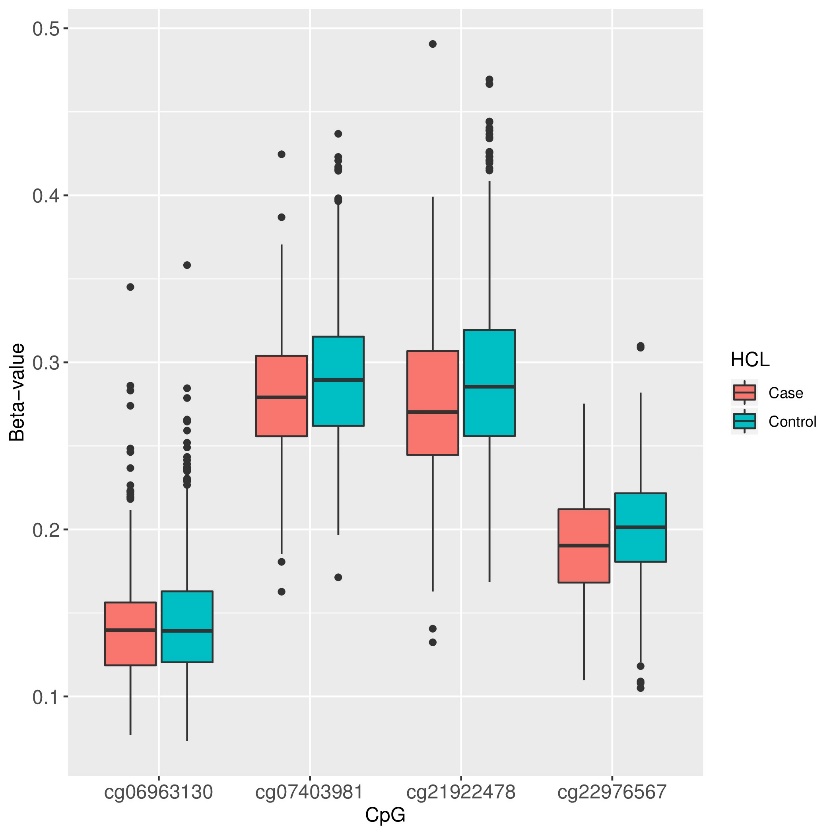
**

**(C) CpG mediators of abdomen-RT and HCL.** P values from T-tests were 0.16, <0.001, <0.0001 and <0.0001 for cg06963130, cg21922478, cg22976567 and cg07403981, respectively.

**
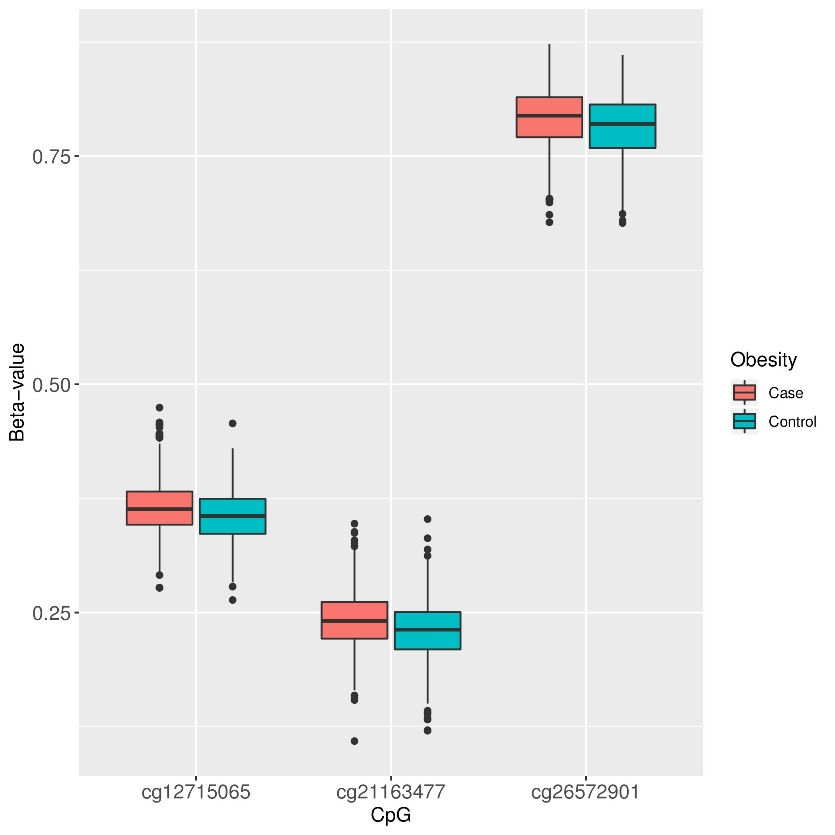
**

**(D) CpG mediators of brain-RT and obesity.** P values from T-tests were < 0.0001 for all three CpGs

**
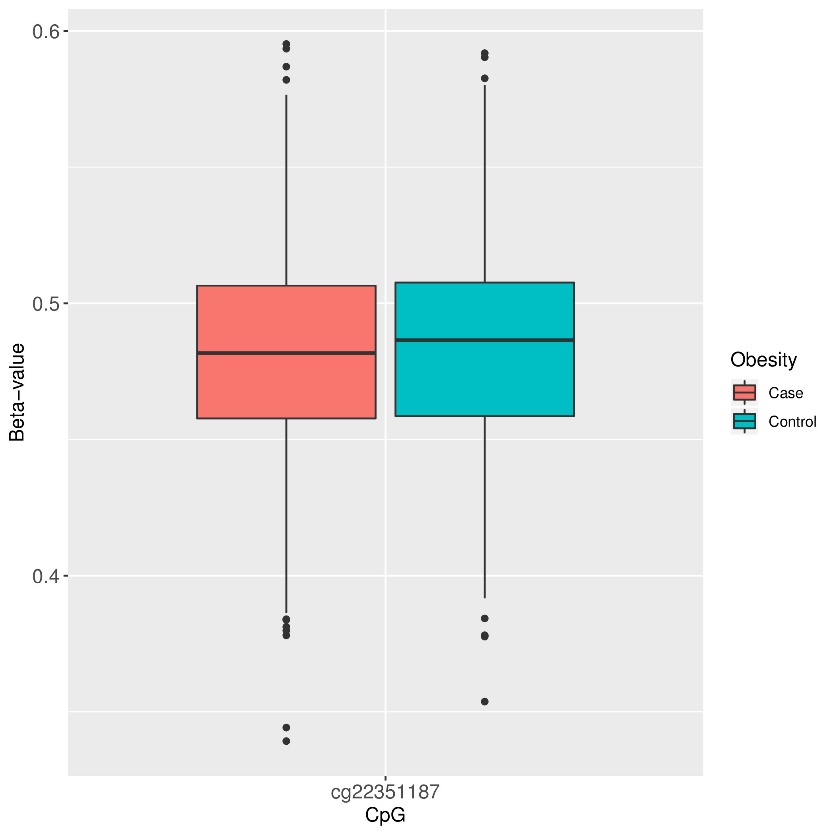
**

**(E) CpG mediators of corticosteroids and obesity.** P value from T-test was 0.17

**Fig. S5. The distribution of beta-values for CpG mediators by groups of CHCs.**

| **Table S1. Pairwise correlations among eleven different treatments** | | | | | | | | | | | | | | | | | | | | | | |
| --- | --- | --- | --- | --- | --- | --- | --- | --- | --- | --- | --- | --- | --- | --- | --- | --- | --- | --- | --- | --- | --- | --- |
| CT/RT | Pearson's correlation coefficient (r) and *p*-value | | | | | | | | | | | | | | | | | | | | | |
|  | Alkylating agent | | Anthracyclines | | Antimetabolites | | Asparaginase enzymes | | Corticosteroids | | Epipodophyllotoxins | | Vinca alkaloids | | Brain RT | | Chest RT | | Abdomen RT | | Pelvis RT | |
| Chemotherapy (CT) |  |  |  |  |  |  |  |  |  |  |  |  |  |  |  |  |  |  |  |  |  |  |
| Alkylating agent | 1.00 | - |  |  |  |  |  |  |  |  |  |  |  |  |  |  |  |  |  |  |  |  |
| Anthracyclines | 0.36 | <0.01 | 1.00 | - |  |  |  |  |  |  |  |  |  |  |  |  |  |  |  |  |  |  |
| Antimetabolites | 0.22 | <0.01 | 0.36 | <0.01 | 1.00 | - |  |  |  |  |  |  |  |  |  |  |  |  |  |  |  |  |
| Asparaginase enzymes | 0.04 | 0.05 | 0.21 | <0.01 | 0.67 | <0.01 | 1.00 | - |  |  |  |  |  |  |  |  |  |  |  |  |  |  |
| Corticosteroids | 0.18 | <0.01 | 0.30 | <0.01 | 0.77 | <0.01 | 0.69 | <0.01 | 1.00 | - |  |  |  |  |  |  |  |  |  |  |  |  |
| Epipodophyllotoxins | 0.19 | <0.01 | 0.35 | <0.01 | 0.34 | <0.01 | 0.55 | <0.01 | 0.37 | <0.01 | 1.00 | - |  |  |  |  |  |  |  |  |  |  |
| Vinca alkaloids | 0.35 | <0.01 | 0.32 | <0.01 | 0.40 | <0.01 | 0.39 | <0.01 | 0.57 | <0.01 | 0.22 | <0.01 | 1.00 | - |  |  |  |  |  |  |  |  |
| Radiation therapy (RT) |  |  |  |  |  |  |  |  |  |  |  |  |  |  |  |  |  |  |  |  |  |  |
| Brain-RT | 0.06 | 0.01 | -0.17 | <0.01 | 0.21 | <0.01 | 0.26 | <0.01 | 0.22 | <0.01 | 0.12 | <0.01 | 0.11 | <0.01 | 1.00 | - |  |  |  |  |  |  |
| Chest-RT | 0.06 | 0.00 | -0.01 | 0.51 | -0.25 | <0.01 | -0.34 | <0.01 | -0.09 | <0.01 | -0.15 | <0.01 | 0.13 | <0.01 | -0.06 | 0.01 | 1.00 | - |  |  |  |  |
| Abdomen-RT | 0.05 | 0.01 | -0.07 | <0.01 | -0.26 | <0.01 | -0.26 | <0.01 | -0.15 | <0.01 | -0.12 | <0.01 | 0.11 | <0.01 | 0.04 | 0.10 | 0.79 | <0.01 | 1.00 | - |  |  |
| Pelvis-RT | 0.09 | <0.01 | -0.03 | 0.19 | -0.21 | <0.01 | -0.22 | <0.01 | -0.15 | <0.01 | -0.08 | <0.01 | 0.14 | <0.01 | 0.09 | <0.01 | 0.62 | <0.01 | 0.79 | <0.01 | 1.00 | - |

| **Table S2. CpG hits associated with the specific treatment exposure (*P*<9x10^-8^)** | | | | | | | | | | |
| --- | --- | --- | --- | --- | --- | --- | --- | --- | --- | --- |
| Treatment | CpG | Chromosome | Start position | End position | HGNC gene | Coefficient | (SE) | *P* | Coefficient_Dose-response_ | *P_Dose-response_* |
| ***Chemotherapy*** |  |  |  |  |  |  |  |  |  |  |
| Alkylating agents | cg03877706 | chr21 | 21,200,312 | 21,200,314 | *NCAM2* | -0.243 | (0.021) | 2.7E-24 | -0.121 | 1.3E-32 |
| Alkylating agents | cg26408927 | chr3 | 53,725,695 | 53,725,697 | *CACNA1D* | -0.189 | (0.017) | 8.7E-23 | -0.098 | 6.2E-33 |
| Alkylating agents | cg08057120 | chr11 | 97,364,170 | 97,364,172 | *NA* | -0.233 | (0.022) | 3.4E-20 | -0.114 | 5.6E-26 |
| Alkylating agents | cg26342454 | chr4 | 10,033,637 | 10,033,639 | *SLC2A9* | -0.228 | (0.022) | 1.3E-19 | -0.118 | 3.4E-28 |
| Alkylating agents | cg12009405 | chr6 | 148,330,194 | 148,330,196 | *SASH1* | -0.185 | (0.018) | 1.8E-19 | -0.089 | 8.4E-24 |
| Alkylating agents | cg27192248 | chr15 | 64,993,330 | 64,993,332 | *NA* | -0.242 | (0.024) | 2.8E-18 | -0.118 | 3.4E-23 |
| Alkylating agents | cg17407859 | chr3 | 165,259,995 | 165,259,997 | *LINC01322* | -0.179 | (0.018) | 4.7E-18 | -0.080 | 1.8E-19 |
| Alkylating agents | cg24833462 | chr2 | 108,053,726 | 108,053,728 | *AC023672.2* | -0.201 | (0.021) | 2.8E-17 | -0.100 | 1.4E-22 |
| Alkylating agents | cg00303773 | chr17 | 17,849,260 | 17,849,262 | *TOM1L2* | -0.182 | (0.019) | 4.7E-17 | -0.087 | 1.2E-20 |
| Alkylating agents | cg21945120 | chr10 | 58,157,562 | 58,157,564 | *NA* | -0.108 | (0.012) | 4.4E-16 | -0.049 | 4.5E-18 |
| Alkylating agents | cg04188877 | chr22 | 42,721,943 | 42,721,945 | *A4GALT* | 0.082 | (0.009) | 6.2E-16 | 0.040 | 1.1E-19 |
| Alkylating agents | cg06991974 | chr1 | 3,067,653 | 3,067,655 | *LINC00982* | 0.094 | (0.010) | 6.9E-16 | 0.047 | 1.0E-20 |
| Alkylating agents | cg10570484 | chr1 | 158,038,015 | 158,038,017 | *KIRREL1* | -0.065 | (0.007) | 3.8E-15 | -0.030 | 3.4E-17 |
| Alkylating agents | cg13224583 | chr1 | 156,913,952 | 156,913,954 | *PEAR1* | 0.089 | (0.010) | 4.2E-15 | 0.047 | 1.0E-22 |
| Alkylating agents | cg04368942 | chr7 | 134,886,568 | 134,886,570 | *CALD1* | -0.088 | (0.010) | 5.6E-15 | -0.041 | 1.9E-17 |
| Alkylating agents | cg08173263 | chr19 | 14,166,098 | 14,166,100 | *ADGRL1;CTB-55O6.12* | 0.147 | (0.017) | 2.2E-14 | 0.068 | 2.8E-16 |
| Alkylating agents | cg06581459 | chr19 | 6,577,656 | 6,577,658 | *NA* | -0.081 | (0.009) | 5.9E-14 | -0.041 | 1.5E-18 |
| Alkylating agents | cg07086679 | chr7 | 140,316,321 | 140,316,323 | *SLC37A3* | -0.092 | (0.011) | 8.9E-14 | -0.048 | 2.3E-19 |
| Alkylating agents | cg07946633 | chr1 | 3,067,680 | 3,067,682 | *LINC00982;PRDM16* | 0.073 | (0.009) | 1.1E-13 | 0.036 | 1.1E-17 |
| Alkylating agents | cg13474360 | chr3 | 192,885,619 | 192,885,621 | *MB21D2* | 0.074 | (0.009) | 1.2E-13 | 0.035 | 5.2E-16 |
| Alkylating agents | cg13486805 | chr19 | 39,248,023 | 39,248,025 | *IFNL4* | 0.093 | (0.011) | 2.5E-13 | 0.049 | 5.5E-19 |
| Alkylating agents | cg27478635 | chr20 | 41,003,796 | 41,003,798 | *NA* | -0.144 | (0.017) | 3.1E-13 | -0.074 | 2.9E-18 |
| Alkylating agents | cg05331731 | chr18 | 11,147,146 | 11,147,148 | *PIEZO2* | 0.155 | (0.019) | 3.7E-13 | 0.073 | 1.4E-15 |
| Alkylating agents | cg11662039 | chr5 | 100,648,041 | 100,648,043 | *NA* | -0.122 | (0.015) | 4.1E-13 | -0.060 | 5.4E-17 |
| Alkylating agents | cg03834031 | chr22 | 46,069,836 | 46,069,838 | *MIRLET7BHG;RP6-109B7.4* | -0.063 | (0.008) | 7.4E-13 | -0.032 | 1.8E-17 |
| Alkylating agents | cg11031221 | chr17 | 72,421,375 | 72,421,377 | *LINC00511* | -0.073 | (0.009) | 7.9E-13 | -0.034 | 1.3E-14 |
| Alkylating agents | cg07677157 | chr12 | 65,657,147 | 65,657,149 | *RP11-221N13.4* | -0.131 | (0.016) | 7.9E-13 | -0.057 | 7.9E-13 |
| Alkylating agents | cg26376241 | chr2 | 65,366,886 | 65,366,888 | *SPRED2* | -0.134 | (0.017) | 1.1E-12 | -0.067 | 1.0E-16 |
| Alkylating agents | cg17200702 | chr11 | 30,992,376 | 30,992,378 | *DCDC1* | -0.120 | (0.015) | 1.2E-12 | -0.067 | 7.2E-21 |
| Alkylating agents | cg17783244 | chr22 | 31,342,562 | 31,342,564 | *PATZ1* | 0.086 | (0.011) | 1.3E-12 | 0.042 | 1.3E-15 |
| Alkylating agents | cg22432387 | chr2 | 20,634,604 | 20,634,606 | *HS1BP3* | -0.142 | (0.018) | 1.7E-12 | -0.073 | 4.7E-17 |
| Alkylating agents | cg05135521 | chr2 | 160,331,823 | 160,331,825 | *RBMS1* | -0.094 | (0.012) | 1.8E-12 | -0.044 | 2.3E-14 |
| Alkylating agents | cg23839200 | chr14 | 51,072,092 | 51,072,094 | *TRIM9* | -0.068 | (0.009) | 2.9E-12 | -0.031 | 4.4E-13 |
| Alkylating agents | cg13294852 | chr2 | 102,055,667 | 102,055,669 | *NA* | -0.098 | (0.012) | 3.1E-12 | -0.047 | 1.2E-14 |
| Alkylating agents | cg26189283 | chr1 | 155,136,901 | 155,136,903 | *SLC50A1* | -0.088 | (0.011) | 3.6E-12 | -0.038 | 2.9E-12 |
| Alkylating agents | cg07861288 | chr13 | 91,719,216 | 91,719,218 | *GPC5* | -0.069 | (0.009) | 3.7E-12 | -0.031 | 9.4E-13 |
| Alkylating agents | cg27326027 | chr18 | 6,885,253 | 6,885,255 | *ARHGAP28* | -0.119 | (0.015) | 4.6E-12 | -0.057 | 1.6E-14 |
| Alkylating agents | cg20598190 | chr2 | 239,927,506 | 239,927,508 | *NDUFA10* | -0.175 | (0.022) | 4.8E-12 | -0.095 | 2.1E-18 |
| Alkylating agents | cg04882213 | chr8 | 60,967,832 | 60,967,834 | *AC022182.3* | -0.115 | (0.015) | 5.3E-12 | -0.051 | 1.9E-12 |
| Alkylating agents | cg06507987 | chr1 | 27,360,276 | 27,360,278 | *MAP3K6* | 0.096 | (0.012) | 5.4E-12 | 0.049 | 4.3E-16 |
| Alkylating agents | cg26621020 | chr8 | 122,647,819 | 122,647,821 | *NA* | -0.117 | (0.015) | 5.7E-12 | -0.058 | 1.4E-15 |
| Alkylating agents | cg00062245 | chr17 | 44,384,891 | 44,384,893 | *ITGA2B* | 0.082 | (0.010) | 6.4E-12 | 0.044 | 1.3E-17 |
| Alkylating agents | cg17763553 | chr8 | 19,095,972 | 19,095,974 | *RP11-1080G15.1;RP11-1080G15.2* | -0.125 | (0.016) | 6.5E-12 | -0.060 | 2.5E-14 |
| Alkylating agents | cg22688137 | chr1 | 218,460,155 | 218,460,157 | *NA* | -0.091 | (0.012) | 9.3E-12 | -0.041 | 9.4E-13 |
| Alkylating agents | cg00973876 | chr17 | 79,925,408 | 79,925,410 | *RP11-353N14.4;RP11-353N14.5* | -0.076 | (0.010) | 9.7E-12 | -0.031 | 1.0E-10 |
| Alkylating agents | cg05483571 | chr17 | 79,927,424 | 79,927,426 | *RP11-353N14.5* | -0.101 | (0.013) | 1.2E-11 | -0.047 | 2.8E-13 |
| Alkylating agents | cg26663696 | chr3 | 160,403,486 | 160,403,488 | *MIR15B;MIR16-2;RP11-432B6.3;SMC4* | -0.122 | (0.016) | 1.3E-11 | -0.052 | 1.7E-11 |
| Alkylating agents | cg17232357 | chr15 | 66,720,493 | 66,720,495 | *SMAD6* | -0.088 | (0.011) | 1.3E-11 | -0.039 | 7.9E-12 |
| Alkylating agents | cg12140144 | chr1 | 3,067,710 | 3,067,712 | *LINC00982;PRDM16* | 0.239 | (0.031) | 2.0E-11 | 0.126 | 1.8E-16 |
| Alkylating agents | cg03864568 | chr9 | 76,400,794 | 76,400,796 | *NA* | -0.105 | (0.014) | 2.6E-11 | -0.045 | 2.7E-11 |
| Alkylating agents | cg01869007 | chr8 | 60,967,853 | 60,967,855 | *AC022182.3* | -0.137 | (0.018) | 2.6E-11 | -0.064 | 7.0E-13 |
| Alkylating agents | cg16198075 | chr4 | 6,417,112 | 6,417,114 | *PPP2R2C* | 0.102 | (0.013) | 2.6E-11 | 0.047 | 1.0E-12 |
| Alkylating agents | cg12800962 | chr20 | 50,009,899 | 50,009,901 | *NA* | 0.090 | (0.012) | 2.7E-11 | 0.042 | 1.2E-12 |
| Alkylating agents | cg14634473 | chr10 | 22,816,507 | 22,816,509 | *NA* | -0.075 | (0.010) | 2.8E-11 | -0.037 | 2.2E-14 |
| Alkylating agents | cg26550214 | chr5 | 34,609,387 | 34,609,389 | *NA* | -0.099 | (0.013) | 3.6E-11 | -0.048 | 1.0E-13 |
| Alkylating agents | cg13903421 | chr2 | 218,873,991 | 218,873,993 | *WNT6* | 0.167 | (0.022) | 3.8E-11 | 0.098 | 8.6E-20 |
| Alkylating agents | cg17858880 | chr7 | 47,537,261 | 47,537,263 | *TNS3* | 0.077 | (0.010) | 3.9E-11 | 0.041 | 2.7E-16 |
| Alkylating agents | cg05468212 | chr9 | 134,796,289 | 134,796,291 | *COL5A1* | 0.047 | (0.006) | 4.0E-11 | 0.024 | 6.0E-15 |
| Alkylating agents | cg22948745 | chr8 | 138,688,051 | 138,688,053 | *COL22A1* | -0.102 | (0.014) | 4.2E-11 | -0.044 | 3.7E-11 |
| Alkylating agents | cg11686251 | chr9 | 131,598,498 | 131,598,500 | *RAPGEF1* | 0.085 | (0.011) | 4.2E-11 | 0.043 | 4.8E-15 |
| Alkylating agents | cg17183905 | chr12 | 109,816,036 | 109,816,038 | *TRPV4* | -0.064 | (0.009) | 5.0E-11 | -0.031 | 1.6E-13 |
| Alkylating agents | cg08866213 | chr3 | 192,812,987 | 192,812,989 | *MB21D2* | 0.115 | (0.016) | 6.8E-11 | 0.062 | 4.3E-16 |
| Alkylating agents | cg03984209 | chr3 | 55,885,989 | 55,885,991 | *ERC2* | -0.088 | (0.012) | 7.0E-11 | -0.044 | 6.1E-14 |
| Alkylating agents | cg06822114 | chr17 | 74,935,513 | 74,935,515 | *AC087651.1;OTOP3* | 0.063 | (0.009) | 8.1E-11 | 0.028 | 2.8E-11 |
| Alkylating agents | cg19664938 | chr5 | 6,493,144 | 6,493,146 | *UBE2QL1* | -0.067 | (0.009) | 1.6E-10 | -0.030 | 4.7E-11 |
| Alkylating agents | cg07265924 | chr8 | 60,967,910 | 60,967,912 | *AC022182.3* | -0.151 | (0.021) | 1.6E-10 | -0.069 | 9.5E-12 |
| Alkylating agents | cg17389813 | chr11 | 122,189,556 | 122,189,558 | *MIR100HG* | -0.134 | (0.019) | 1.9E-10 | -0.065 | 6.0E-13 |
| Alkylating agents | cg07097098 | chr19 | 55,210,269 | 55,210,271 | *PTPRH* | -0.071 | (0.010) | 1.9E-10 | -0.034 | 3.5E-12 |
| Alkylating agents | cg03065175 | chr4 | 154,740,605 | 154,740,607 | *LRAT* | 0.091 | (0.013) | 2.1E-10 | 0.042 | 1.6E-11 |
| Alkylating agents | cg15170715 | chr2 | 27,086,008 | 27,086,010 | *EMILIN1;KHK* | 0.102 | (0.014) | 2.6E-10 | 0.057 | 1.3E-16 |
| Alkylating agents | cg17996394 | chr19 | 4,075,485 | 4,075,487 | *NA* | -0.052 | (0.007) | 3.0E-10 | -0.023 | 1.7E-10 |
| Alkylating agents | cg13442016 | chr10 | 102,436,581 | 102,436,583 | *MIR146B* | -0.087 | (0.012) | 3.1E-10 | -0.042 | 3.3E-12 |
| Alkylating agents | cg13511623 | chr9 | 123,716,678 | 123,716,680 | *DENND1A* | -0.118 | (0.016) | 3.2E-10 | -0.057 | 1.3E-12 |
| Alkylating agents | cg03479114 | chr4 | 165,359,572 | 165,359,574 | *NA* | -0.091 | (0.013) | 3.4E-10 | -0.045 | 4.9E-13 |
| Alkylating agents | cg15251748 | chr7 | 28,953,847 | 28,953,849 | *TRIL* | -0.105 | (0.015) | 3.4E-10 | -0.056 | 1.2E-14 |
| Alkylating agents | cg08919084 | chr15 | 92,012,157 | 92,012,159 | *SLCO3A1* | -0.082 | (0.011) | 3.6E-10 | -0.038 | 2.2E-11 |
| Alkylating agents | cg21513437 | chr17 | 41,521,854 | 41,521,856 | *KRT15* | -0.046 | (0.006) | 3.6E-10 | -0.023 | 1.2E-13 |
| Alkylating agents | cg12960124 | chr4 | 82,100,021 | 82,100,023 | *NA* | -0.092 | (0.013) | 3.7E-10 | -0.052 | 1.0E-16 |
| Alkylating agents | cg21150701 | chr2 | 120,899,241 | 120,899,243 | *GLI2* | 0.067 | (0.009) | 3.7E-10 | 0.034 | 9.6E-14 |
| Alkylating agents | cg05944249 | chr8 | 27,636,723 | 27,636,725 | *SCARA3* | 0.112 | (0.016) | 3.8E-10 | 0.054 | 1.9E-12 |
| Alkylating agents | cg21443659 | chr2 | 216,880,997 | 216,880,999 | *AC007563.5* | -0.094 | (0.013) | 3.9E-10 | -0.042 | 1.2E-10 |
| Alkylating agents | cg13935634 | chr6 | 10,583,535 | 10,583,537 | *GCNT2* | -0.082 | (0.011) | 3.9E-10 | -0.042 | 4.0E-14 |
| Alkylating agents | cg21933008 | chr7 | 28,542,336 | 28,542,338 | *CREB5* | 0.070 | (0.010) | 4.4E-10 | 0.032 | 2.8E-11 |
| Alkylating agents | cg25788793 | chr4 | 10,032,955 | 10,032,957 | *SLC2A9* | -0.065 | (0.009) | 4.4E-10 | -0.033 | 4.7E-13 |
| Alkylating agents | cg03026982 | chr11 | 19,932,152 | 19,932,154 | *NAV2* | -0.079 | (0.011) | 4.4E-10 | -0.039 | 5.9E-13 |
| Alkylating agents | cg14200569 | chr1 | 3,135,360 | 3,135,362 | *PRDM16* | 0.116 | (0.016) | 4.6E-10 | 0.060 | 7.5E-14 |
| Alkylating agents | cg17279142 | chr2 | 142,834,321 | 142,834,323 | *NA* | -0.116 | (0.016) | 4.7E-10 | -0.056 | 4.1E-12 |
| Alkylating agents | cg25242471 | chr2 | 218,874,009 | 218,874,011 | *WNT6* | 0.162 | (0.023) | 5.5E-10 | 0.091 | 5.0E-16 |
| Alkylating agents | cg08271909 | chr4 | 154,740,538 | 154,740,540 | *LRAT* | 0.093 | (0.013) | 5.8E-10 | 0.046 | 1.6E-12 |
| Alkylating agents | cg12882503 | chr13 | 112,992,378 | 112,992,380 | *MCF2L* | 0.084 | (0.012) | 6.5E-10 | 0.043 | 1.5E-13 |
| Alkylating agents | cg16112727 | chr1 | 156,913,429 | 156,913,431 | *PEAR1* | 0.059 | (0.008) | 6.8E-10 | 0.027 | 3.5E-11 |
| Alkylating agents | cg13081213 | chr7 | 26,675,137 | 26,675,139 | *SKAP2* | -0.089 | (0.013) | 7.5E-10 | -0.043 | 5.7E-12 |
| Alkylating agents | cg01543150 | chr3 | 192,916,271 | 192,916,273 | *MB21D2* | 0.080 | (0.011) | 7.5E-10 | 0.038 | 1.1E-11 |
| Alkylating agents | cg09762316 | chr9 | 70,043,437 | 70,043,439 | *MAMDC2;MAMDC2-AS1* | 0.069 | (0.010) | 7.5E-10 | 0.034 | 7.4E-13 |
| Alkylating agents | cg15143202 | chr3 | 169,148,736 | 169,148,738 | *MECOM* | -0.098 | (0.014) | 7.8E-10 | -0.043 | 4.0E-10 |
| Alkylating agents | cg11228724 | chr3 | 192,826,101 | 192,826,103 | *MB21D2* | 0.064 | (0.009) | 8.2E-10 | 0.030 | 1.8E-11 |
| Alkylating agents | cg17109563 | chr6 | 125,363,279 | 125,363,281 | *NA* | -0.091 | (0.013) | 8.4E-10 | -0.045 | 1.1E-12 |
| Alkylating agents | cg19805775 | chr7 | 82,534,800 | 82,534,802 | *NA* | -0.084 | (0.012) | 8.8E-10 | -0.038 | 2.0E-10 |
| Alkylating agents | cg08119153 | chr14 | 78,446,957 | 78,446,959 | *NRXN3* | 0.102 | (0.015) | 9.2E-10 | 0.057 | 2.1E-15 |
| Alkylating agents | cg03664096 | chr21 | 43,197,746 | 43,197,748 | *NA* | -0.073 | (0.010) | 9.4E-10 | -0.031 | 1.8E-09 |
| Alkylating agents | cg11339839 | chr17 | 79,927,329 | 79,927,331 | *RP11-353N14.5* | -0.052 | (0.008) | 9.6E-10 | -0.024 | 3.9E-11 |
| Alkylating agents | cg22239727 | chr11 | 19,242,886 | 19,242,888 | *E2F8;RP11-428C19.4* | 0.051 | (0.007) | 1.0E-09 | 0.023 | 2.6E-10 |
| Alkylating agents | cg20543681 | chr20 | 662,028 | 662,030 | *RP5-850E9.3;SCRT2* | 0.062 | (0.009) | 1.1E-09 | 0.031 | 7.5E-13 |
| Alkylating agents | cg04895971 | chr4 | 69,858,820 | 69,858,822 | *SULT1E1* | -0.084 | (0.012) | 1.1E-09 | -0.031 | 2.0E-07 |
| Alkylating agents | cg08101174 | chr10 | 102,436,783 | 102,436,785 | *NA* | -0.067 | (0.010) | 1.2E-09 | -0.030 | 1.9E-10 |
| Alkylating agents | cg09835575 | chr20 | 9,985,215 | 9,985,217 | *ANKEF1;RP5-839B4.8* | -0.092 | (0.013) | 1.2E-09 | -0.047 | 9.5E-13 |
| Alkylating agents | cg01102638 | chr10 | 85,919,524 | 85,919,526 | *GRID1* | -0.116 | (0.017) | 1.3E-09 | -0.047 | 1.2E-08 |
| Alkylating agents | cg19616242 | chr16 | 67,169,318 | 67,169,320 | *HSF4;NOL3* | 0.067 | (0.010) | 1.5E-09 | 0.034 | 9.8E-13 |
| Alkylating agents | cg16128859 | chr3 | 62,145,418 | 62,145,420 | *PTPRG* | -0.046 | (0.007) | 1.5E-09 | -0.022 | 7.5E-12 |
| Alkylating agents | cg19080839 | chr7 | 151,242,505 | 151,242,507 | *SMARCD3* | 0.068 | (0.010) | 1.6E-09 | 0.035 | 3.4E-13 |
| Alkylating agents | cg16985233 | chr15 | 43,514,050 | 43,514,052 | *MAP1A* | -0.091 | (0.013) | 1.7E-09 | -0.044 | 1.0E-11 |
| Alkylating agents | cg05712639 | chr14 | 52,352,667 | 52,352,669 | *NA* | -0.136 | (0.020) | 1.7E-09 | -0.070 | 4.3E-13 |
| Alkylating agents | cg17144149 | chr17 | 48,579,209 | 48,579,211 | *HOXB-AS3;HOXB3;HOXB4* | -0.099 | (0.014) | 1.7E-09 | -0.043 | 1.1E-09 |
| Alkylating agents | cg01012330 | chr3 | 192,916,269 | 192,916,271 | *MB21D2* | 0.068 | (0.010) | 1.8E-09 | 0.035 | 8.5E-13 |
| Alkylating agents | cg11988169 | chr5 | 53,543,411 | 53,543,413 | *NA* | -0.198 | (0.029) | 1.8E-09 | -0.104 | 2.0E-13 |
| Alkylating agents | cg26491604 | chr17 | 48,579,501 | 48,579,503 | *HOXB-AS3;HOXB3;HOXB4* | -0.052 | (0.008) | 1.8E-09 | -0.024 | 1.2E-10 |
| Alkylating agents | cg01435315 | chr10 | 70,588,663 | 70,588,665 | *NA* | -0.094 | (0.014) | 1.8E-09 | -0.047 | 2.6E-12 |
| Alkylating agents | cg01198738 | chr8 | 120,585,378 | 120,585,380 | *SNTB1* | -0.078 | (0.011) | 1.9E-09 | -0.038 | 7.8E-12 |
| Alkylating agents | cg14639163 | chr2 | 218,873,806 | 218,873,808 | *WNT6* | 0.103 | (0.015) | 1.9E-09 | 0.066 | 2.5E-19 |
| Alkylating agents | cg21774980 | chr20 | 35,502,719 | 35,502,721 | *CEP250* | 0.042 | (0.006) | 2.0E-09 | 0.022 | 1.6E-13 |
| Alkylating agents | cg24539599 | chr5 | 113,489,222 | 113,489,224 | *MCC* | -0.064 | (0.009) | 2.0E-09 | -0.031 | 1.2E-11 |
| Alkylating agents | cg20517202 | chr16 | 21,150,520 | 21,150,522 | *DNAH3* | -0.097 | (0.014) | 2.1E-09 | -0.042 | 1.1E-09 |
| Alkylating agents | cg16449837 | chr12 | 15,279,392 | 15,279,394 | *RERG* | -0.057 | (0.008) | 2.1E-09 | -0.027 | 5.0E-11 |
| Alkylating agents | cg02023167 | chr11 | 19,242,643 | 19,242,645 | *E2F8;RP11-428C19.4* | 0.045 | (0.007) | 2.2E-09 | 0.023 | 7.4E-13 |
| Alkylating agents | cg07573872 | chr19 | 1,126,342 | 1,126,344 | *SBNO2* | -0.070 | (0.010) | 2.2E-09 | -0.038 | 2.9E-14 |
| Alkylating agents | cg10755723 | chr3 | 192,859,174 | 192,859,176 | *MB21D2* | 0.046 | (0.007) | 2.2E-09 | 0.024 | 1.3E-12 |
| Alkylating agents | cg08909363 | chr11 | 72,181,494 | 72,181,496 | *RP11-807H22.7* | 0.051 | (0.007) | 2.4E-09 | 0.025 | 1.6E-11 |
| Alkylating agents | cg11994851 | chr11 | 94,767,901 | 94,767,903 | *AMOTL1* | -0.051 | (0.008) | 2.4E-09 | -0.022 | 3.6E-09 |
| Alkylating agents | cg26811385 | chr4 | 149,910,850 | 149,910,852 | *NA* | -0.091 | (0.013) | 2.7E-09 | -0.047 | 1.2E-12 |
| Alkylating agents | cg21393163 | chr1 | 12,157,571 | 12,157,573 | *NA* | -0.093 | (0.014) | 2.9E-09 | -0.042 | 3.5E-10 |
| Alkylating agents | cg18419358 | chr6 | 157,962,976 | 157,962,978 | *NA* | -0.106 | (0.016) | 3.0E-09 | -0.055 | 1.1E-12 |
| Alkylating agents | cg08903740 | chr1 | 162,317,927 | 162,317,929 | *NOS1AP;RP11-565P22.2* | 0.064 | (0.010) | 3.3E-09 | 0.035 | 8.3E-14 |
| Alkylating agents | cg03868982 | chr19 | 6,578,062 | 6,578,064 | *NA* | 0.070 | (0.010) | 3.3E-09 | 0.028 | 4.9E-08 |
| Alkylating agents | cg09921385 | chr4 | 6,999,154 | 6,999,156 | *TBC1D14* | -0.088 | (0.013) | 3.6E-09 | -0.045 | 1.3E-12 |
| Alkylating agents | cg08662753 | chr9 | 134,669,653 | 134,669,655 | *COL5A1* | 0.072 | (0.011) | 3.6E-09 | 0.040 | 3.7E-14 |
| Alkylating agents | cg25896883 | chr2 | 189,407,943 | 189,407,945 | *NA* | -0.092 | (0.014) | 3.7E-09 | -0.049 | 2.7E-13 |
| Alkylating agents | cg06180200 | chr19 | 38,912,295 | 38,912,297 | *CCER2* | 0.074 | (0.011) | 3.7E-09 | 0.038 | 1.7E-12 |
| Alkylating agents | cg27423177 | chr12 | 8,066,761 | 8,066,763 | *C3AR1* | -0.089 | (0.013) | 4.1E-09 | -0.043 | 6.3E-11 |
| Alkylating agents | cg06260709 | chr20 | 50,010,083 | 50,010,085 | *NA* | 0.081 | (0.012) | 4.4E-09 | 0.038 | 1.0E-10 |
| Alkylating agents | cg27024924 | chr6 | 21,186,285 | 21,186,287 | *CDKAL1* | -0.108 | (0.016) | 4.5E-09 | -0.051 | 1.3E-10 |
| Alkylating agents | cg10473623 | chr11 | 20,097,629 | 20,097,631 | *NAV2* | -0.081 | (0.012) | 4.5E-09 | -0.036 | 6.6E-10 |
| Alkylating agents | cg20996351 | chr1 | 27,360,740 | 27,360,742 | *MAP3K6* | 0.245 | (0.037) | 4.6E-09 | 0.124 | 6.6E-12 |
| Alkylating agents | cg00806461 | chr3 | 194,687,341 | 194,687,343 | *FAM43A* | 0.060 | (0.009) | 4.7E-09 | 0.024 | 3.8E-08 |
| Alkylating agents | cg09764150 | chr14 | 64,280,395 | 64,280,397 | *ESR2* | -0.120 | (0.018) | 5.3E-09 | -0.054 | 9.8E-10 |
| Alkylating agents | cg02541477 | chr13 | 52,061,901 | 52,061,903 | *NEK5* | -0.125 | (0.019) | 5.4E-09 | -0.052 | 1.6E-08 |
| Alkylating agents | cg01674036 | chr10 | 72,298,243 | 72,298,245 | *NA* | -0.082 | (0.012) | 5.5E-09 | -0.039 | 1.5E-10 |
| Alkylating agents | cg06268875 | chr18 | 11,147,385 | 11,147,387 | *PIEZO2* | 0.094 | (0.014) | 5.8E-09 | 0.048 | 5.3E-12 |
| Alkylating agents | cg00494337 | chr20 | 31,606,220 | 31,606,222 | *ID1;MIR3193* | -0.064 | (0.010) | 5.9E-09 | -0.030 | 2.8E-10 |
| Alkylating agents | cg20977229 | chr7 | 28,749,516 | 28,749,518 | *CREB5* | -0.124 | (0.019) | 6.1E-09 | -0.060 | 9.1E-11 |
| Alkylating agents | cg22573230 | chr17 | 7,745,952 | 7,745,954 | *DNAH2* | -0.061 | (0.009) | 6.1E-09 | -0.031 | 2.4E-12 |
| Alkylating agents | cg04742397 | chr14 | 95,520,216 | 95,520,218 | *NA* | -0.038 | (0.006) | 6.1E-09 | -0.017 | 1.5E-09 |
| Alkylating agents | cg01234420 | chr22 | 46,057,927 | 46,057,929 | *MIRLET7BHG;RP6-109B7.2* | -0.064 | (0.010) | 6.7E-09 | -0.030 | 1.7E-10 |
| Alkylating agents | cg13329407 | chr21 | 24,429,059 | 24,429,061 | *AP000476.1* | -0.141 | (0.021) | 6.8E-09 | -0.074 | 1.5E-12 |
| Alkylating agents | cg06808571 | chr7 | 150,945,167 | 150,945,169 | *KCNH2* | 0.066 | (0.010) | 6.9E-09 | 0.032 | 3.0E-11 |
| Alkylating agents | cg15965233 | chr2 | 164,795,047 | 164,795,049 | *COBLL1* | -0.060 | (0.009) | 7.5E-09 | -0.028 | 7.7E-10 |
| Alkylating agents | cg01004017 | chr2 | 120,899,770 | 120,899,772 | *GLI2* | 0.090 | (0.014) | 8.1E-09 | 0.048 | 1.3E-12 |
| Alkylating agents | cg02203380 | chr7 | 30,134,192 | 30,134,194 | *MTURN* | -0.066 | (0.010) | 9.0E-09 | -0.030 | 6.7E-10 |
| Alkylating agents | cg11077681 | chr11 | 10,693,640 | 10,693,642 | *MRVI1* | 0.067 | (0.010) | 9.3E-09 | 0.032 | 1.8E-10 |
| Alkylating agents | cg00218103 | chr22 | 46,070,096 | 46,070,098 | *MIRLET7BHG;RP6-109B7.4* | -0.072 | (0.011) | 9.5E-09 | -0.037 | 3.8E-12 |
| Alkylating agents | cg01838523 | chr20 | 44,714,648 | 44,714,650 | *KCNK15-AS1;WISP2* | -0.054 | (0.008) | 9.6E-09 | -0.027 | 4.7E-11 |
| Alkylating agents | cg03748458 | chr8 | 14,853,738 | 14,853,740 | *MIR383;SGCZ* | -0.087 | (0.013) | 9.7E-09 | -0.044 | 2.2E-11 |
| Alkylating agents | cg08573679 | chr6 | 125,363,363 | 125,363,365 | *NA* | -0.089 | (0.014) | 9.7E-09 | -0.043 | 1.1E-10 |
| Alkylating agents | cg12769615 | chr4 | 94,732,004 | 94,732,006 | *NA* | -0.069 | (0.011) | 9.9E-09 | -0.031 | 4.8E-09 |
| Alkylating agents | cg12526942 | chr18 | 63,208,322 | 63,208,324 | *BCL2* | -0.065 | (0.010) | 1.0E-08 | -0.031 | 3.7E-10 |
| Alkylating agents | cg01530498 | chr22 | 27,475,264 | 27,475,266 | *NA* | -0.065 | (0.010) | 1.0E-08 | -0.032 | 5.6E-11 |
| Alkylating agents | cg05848650 | chr3 | 192,893,050 | 192,893,052 | *MB21D2* | -0.110 | (0.017) | 1.0E-08 | -0.057 | 5.5E-12 |
| Alkylating agents | cg22108243 | chr1 | 68,725,462 | 68,725,464 | *NA* | -0.105 | (0.016) | 1.0E-08 | -0.056 | 1.4E-12 |
| Alkylating agents | cg26931213 | chr8 | 23,668,552 | 23,668,554 | *NA* | 0.125 | (0.019) | 1.0E-08 | 0.058 | 6.7E-10 |
| Alkylating agents | cg21585138 | chr3 | 50,607,674 | 50,607,676 | *CISH* | -0.075 | (0.011) | 1.1E-08 | -0.035 | 6.4E-10 |
| Alkylating agents | cg14143795 | chr16 | 21,150,833 | 21,150,835 | *DNAH3* | -0.099 | (0.015) | 1.1E-08 | -0.044 | 4.2E-09 |
| Alkylating agents | cg15396830 | chr4 | 89,303,327 | 89,303,329 | *GPRIN3* | -0.111 | (0.017) | 1.1E-08 | -0.046 | 5.6E-08 |
| Alkylating agents | cg00166216 | chr3 | 194,687,130 | 194,687,132 | *FAM43A* | 0.056 | (0.009) | 1.2E-08 | 0.025 | 6.1E-09 |
| Alkylating agents | cg17102948 | chr8 | 19,096,237 | 19,096,239 | *RP11-1080G15.1;RP11-1080G15.2* | -0.055 | (0.008) | 1.2E-08 | -0.028 | 1.3E-11 |
| Alkylating agents | cg07625513 | chr15 | 89,034,554 | 89,034,556 | *NA* | -0.073 | (0.011) | 1.2E-08 | -0.027 | 1.5E-06 |
| Alkylating agents | cg01693697 | chr15 | 73,920,554 | 73,920,556 | *LOXL1-AS1* | -0.090 | (0.014) | 1.2E-08 | -0.050 | 1.8E-13 |
| Alkylating agents | cg09331409 | chr11 | 75,604,070 | 75,604,072 | *MAP6* | -0.068 | (0.010) | 1.3E-08 | -0.030 | 3.5E-09 |
| Alkylating agents | cg10762064 | chr3 | 194,967,685 | 194,967,687 | *NA* | -0.070 | (0.011) | 1.3E-08 | -0.035 | 9.1E-11 |
| Alkylating agents | cg10587886 | chr3 | 8,320,146 | 8,320,148 | *LMCD1-AS1* | -0.108 | (0.017) | 1.4E-08 | -0.048 | 5.3E-09 |
| Alkylating agents | cg27330053 | chr2 | 152,200,783 | 152,200,785 | *NA* | -0.042 | (0.006) | 1.4E-08 | -0.018 | 1.0E-08 |
| Alkylating agents | cg15105359 | chr7 | 47,537,358 | 47,537,360 | *TNS3* | 0.165 | (0.026) | 1.4E-08 | 0.094 | 3.9E-14 |
| Alkylating agents | cg05293861 | chr12 | 4,153,541 | 4,153,543 | *NA* | -0.105 | (0.016) | 1.4E-08 | -0.048 | 2.1E-09 |
| Alkylating agents | cg01511232 | chr4 | 154,740,776 | 154,740,778 | *LRAT* | 0.102 | (0.016) | 1.4E-08 | 0.053 | 1.4E-11 |
| Alkylating agents | cg20970886 | chr3 | 192,850,924 | 192,850,926 | *MB21D2* | -0.066 | (0.010) | 1.4E-08 | -0.031 | 3.7E-10 |
| Alkylating agents | cg01751245 | chr2 | 65,366,626 | 65,366,628 | *SPRED2* | -0.092 | (0.014) | 1.5E-08 | -0.045 | 1.5E-10 |
| Alkylating agents | cg05107036 | chr8 | 140,653,660 | 140,653,662 | *NA* | -0.071 | (0.011) | 1.5E-08 | -0.032 | 3.6E-09 |
| Alkylating agents | cg18158149 | chr1 | 162,168,424 | 162,168,426 | *NOS1AP* | -0.055 | (0.009) | 1.5E-08 | -0.032 | 2.0E-14 |
| Alkylating agents | cg11419575 | chr2 | 152,200,863 | 152,200,865 | *NA* | -0.045 | (0.007) | 1.5E-08 | -0.018 | 1.4E-07 |
| Alkylating agents | cg00264650 | chr9 | 22,030,259 | 22,030,261 | *CDKN2B-AS1;RP11-145E5.5* | 0.051 | (0.008) | 1.6E-08 | 0.021 | 1.2E-07 |
| Alkylating agents | cg09130658 | chr17 | 79,927,230 | 79,927,232 | *RP11-353N14.5* | -0.072 | (0.011) | 1.6E-08 | -0.031 | 1.7E-08 |
| Alkylating agents | cg18676273 | chr7 | 41,693,234 | 41,693,236 | *AC005027.3;INHBA;INHBA-AS1* | -0.060 | (0.009) | 1.7E-08 | -0.027 | 2.7E-09 |
| Alkylating agents | cg13670378 | chr17 | 48,579,414 | 48,579,416 | *HOXB-AS3;HOXB3;HOXB4* | -0.077 | (0.012) | 1.7E-08 | -0.036 | 9.3E-10 |
| Alkylating agents | cg21141726 | chr11 | 57,251,946 | 57,251,948 | *NA* | -0.067 | (0.010) | 1.7E-08 | -0.025 | 1.1E-06 |
| Alkylating agents | cg01278701 | chr11 | 102,760,655 | 102,760,657 | *WTAPP1* | -0.081 | (0.013) | 1.7E-08 | -0.038 | 1.3E-09 |
| Alkylating agents | cg25410220 | chr7 | 69,801,822 | 69,801,824 | *AUTS2* | -0.060 | (0.009) | 1.8E-08 | -0.029 | 3.3E-10 |
| Alkylating agents | cg18480977 | chr19 | 13,072,035 | 13,072,037 | *AC007787.2;NFIX* | 0.053 | (0.008) | 1.8E-08 | 0.028 | 8.7E-12 |
| Alkylating agents | cg06963130 | chr2 | 235,434,127 | 235,434,129 | *NA* | -0.079 | (0.012) | 1.9E-08 | -0.035 | 5.4E-09 |
| Alkylating agents | cg25241559 | chr2 | 241,049,961 | 241,049,963 | *AC005237.4;SNED1* | 0.050 | (0.008) | 1.9E-08 | 0.021 | 4.5E-08 |
| Alkylating agents | cg15871086 | chr18 | 58,859,362 | 58,859,364 | *NA* | -0.065 | (0.010) | 2.0E-08 | -0.029 | 5.0E-09 |
| Alkylating agents | cg09146183 | chr22 | 38,214,368 | 38,214,370 | *MAFF* | 0.077 | (0.012) | 2.0E-08 | 0.041 | 3.5E-12 |
| Alkylating agents | cg07872945 | chr9 | 134,010,913 | 134,010,915 | *NA* | -0.067 | (0.011) | 2.0E-08 | -0.028 | 9.4E-08 |
| Alkylating agents | cg05449815 | chr17 | 3,640,570 | 3,640,572 | *CTNS* | -0.065 | (0.010) | 2.0E-08 | -0.033 | 2.6E-11 |
| Alkylating agents | cg15491247 | chr1 | 3,313,859 | 3,313,861 | *PRDM16* | -0.065 | (0.010) | 2.0E-08 | -0.031 | 4.2E-10 |
| Alkylating agents | cg24765521 | chr9 | 90,296,803 | 90,296,805 | *NA* | -0.103 | (0.016) | 2.1E-08 | -0.055 | 3.6E-12 |
| Alkylating agents | cg09701700 | chr10 | 102,435,085 | 102,435,087 | *MIR146B* | -0.059 | (0.009) | 2.1E-08 | -0.025 | 1.9E-08 |
| Alkylating agents | cg17593512 | chr4 | 40,439,495 | 40,439,497 | *RBM47* | -0.091 | (0.014) | 2.1E-08 | -0.041 | 5.8E-09 |
| Alkylating agents | cg19075225 | chr2 | 241,070,097 | 241,070,099 | *SNED1* | 0.093 | (0.015) | 2.1E-08 | 0.042 | 5.2E-09 |
| Alkylating agents | cg07710094 | chr8 | 129,267,705 | 129,267,707 | *NA* | -0.082 | (0.013) | 2.2E-08 | -0.045 | 1.2E-12 |
| Alkylating agents | cg22156456 | chr17 | 41,687,986 | 41,687,988 | *EIF1* | -0.054 | (0.008) | 2.2E-08 | -0.027 | 6.3E-11 |
| Alkylating agents | cg00753112 | chr8 | 135,581,057 | 135,581,059 | *KHDRBS3* | -0.041 | (0.006) | 2.3E-08 | -0.018 | 2.5E-08 |
| Alkylating agents | cg03078557 | chr1 | 162,285,248 | 162,285,250 | *NOS1AP* | -0.134 | (0.021) | 2.3E-08 | -0.068 | 3.2E-11 |
| Alkylating agents | cg17445936 | chr1 | 3,173,780 | 3,173,782 | *PRDM16* | 0.122 | (0.019) | 2.3E-08 | 0.065 | 4.7E-12 |
| Alkylating agents | cg21302696 | chr14 | 94,390,773 | 94,390,775 | *SERPINA1* | -0.054 | (0.008) | 2.4E-08 | -0.025 | 1.8E-09 |
| Alkylating agents | cg02849956 | chr19 | 4,634,814 | 4,634,816 | *NA* | -0.042 | (0.007) | 2.6E-08 | -0.018 | 2.6E-08 |
| Alkylating agents | cg10541181 | chr12 | 23,559,658 | 23,559,660 | *SOX5* | -0.110 | (0.017) | 2.7E-08 | -0.060 | 1.2E-12 |
| Alkylating agents | cg11093223 | chr9 | 34,363,683 | 34,363,685 | *NA* | 0.041 | (0.007) | 2.7E-08 | 0.021 | 8.3E-11 |
| Alkylating agents | cg01246599 | chr19 | 11,256,411 | 11,256,413 | *DOCK6* | 0.069 | (0.011) | 2.7E-08 | 0.036 | 1.2E-11 |
| Alkylating agents | cg26237233 | chr11 | 81,071,020 | 81,071,022 | *NA* | -0.048 | (0.008) | 2.7E-08 | -0.023 | 8.3E-10 |
| Alkylating agents | cg26936966 | chr8 | 135,599,881 | 135,599,883 | *KHDRBS3* | 0.054 | (0.009) | 2.9E-08 | 0.025 | 4.2E-09 |
| Alkylating agents | cg26612799 | chr1 | 237,672,941 | 237,672,943 | *RYR2* | -0.068 | (0.011) | 3.1E-08 | -0.033 | 8.6E-10 |
| Alkylating agents | cg07518532 | chr11 | 1,968,276 | 1,968,278 | *MRPL23* | 0.045 | (0.007) | 3.1E-08 | 0.023 | 1.8E-10 |
| Alkylating agents | cg27589809 | chr3 | 50,612,978 | 50,612,980 | *CISH;MAPKAPK3* | -0.051 | (0.008) | 3.2E-08 | -0.025 | 3.3E-10 |
| Alkylating agents | cg27115863 | chr22 | 37,525,632 | 37,525,634 | *NA* | -0.045 | (0.007) | 3.2E-08 | -0.019 | 4.3E-08 |
| Alkylating agents | cg18742528 | chr6 | 13,053,287 | 13,053,289 | *PHACTR1* | -0.054 | (0.009) | 3.2E-08 | -0.024 | 2.0E-08 |
| Alkylating agents | cg06855546 | chr16 | 1,488,824 | 1,488,826 | *PTX4* | 0.053 | (0.008) | 3.5E-08 | 0.028 | 6.8E-12 |
| Alkylating agents | cg17438696 | chr1 | 186,806,703 | 186,806,705 | *NA* | -0.079 | (0.013) | 3.6E-08 | -0.037 | 1.9E-09 |
| Alkylating agents | cg06269255 | chr17 | 44,269,426 | 44,269,428 | *SLC4A1* | -0.047 | (0.008) | 3.6E-08 | -0.022 | 5.3E-09 |
| Alkylating agents | cg14203118 | chr8 | 143,598,722 | 143,598,724 | *EEF1D;TIGD5* | 0.128 | (0.020) | 3.6E-08 | 0.055 | 3.8E-08 |
| Alkylating agents | cg18763712 | chr2 | 74,602,770 | 74,602,772 | *M1AP* | -0.075 | (0.012) | 3.8E-08 | -0.037 | 3.3E-10 |
| Alkylating agents | cg14436379 | chr3 | 122,245,755 | 122,245,757 | *CASR* | -0.095 | (0.015) | 3.8E-08 | -0.042 | 2.2E-08 |
| Alkylating agents | cg08774778 | chr5 | 62,082,559 | 62,082,561 | *NA* | -0.075 | (0.012) | 3.9E-08 | -0.034 | 7.5E-09 |
| Alkylating agents | cg20700458 | chr1 | 209,884,839 | 209,884,841 | *NA* | -0.056 | (0.009) | 3.9E-08 | -0.020 | 4.3E-06 |
| Alkylating agents | cg11796961 | chr12 | 100,959,608 | 100,959,610 | *ANO4* | -0.050 | (0.008) | 4.1E-08 | -0.022 | 2.3E-08 |
| Alkylating agents | cg11840258 | chr20 | 50,226,580 | 50,226,582 | *NA* | -0.039 | (0.006) | 4.2E-08 | -0.017 | 1.3E-08 |
| Alkylating agents | cg09895388 | chr3 | 192,902,276 | 192,902,278 | *MB21D2* | 0.048 | (0.008) | 4.3E-08 | 0.021 | 1.3E-08 |
| Alkylating agents | cg07761095 | chr5 | 84,394,170 | 84,394,172 | *CTD-2269F5.1* | -0.071 | (0.011) | 4.4E-08 | -0.033 | 5.5E-09 |
| Alkylating agents | cg22013747 | chr12 | 18,999,131 | 18,999,133 | *NA* | -0.050 | (0.008) | 4.5E-08 | -0.025 | 2.4E-10 |
| Alkylating agents | cg26763855 | chr19 | 38,912,281 | 38,912,283 | *CCER2* | 0.069 | (0.011) | 4.6E-08 | 0.036 | 4.5E-11 |
| Alkylating agents | cg25360591 | chr5 | 164,353,338 | 164,353,340 | *CTC-340A15.2* | -0.106 | (0.017) | 4.6E-08 | -0.053 | 1.9E-10 |
| Alkylating agents | cg26296337 | chr17 | 74,935,696 | 74,935,698 | *AC087651.1;OTOP3* | 0.047 | (0.008) | 4.6E-08 | 0.020 | 4.4E-08 |
| Alkylating agents | cg07210082 | chr2 | 20,185,762 | 20,185,764 | *NA* | 0.058 | (0.009) | 4.7E-08 | 0.029 | 2.2E-10 |
| Alkylating agents | cg00589617 | chr1 | 230,279,596 | 230,279,598 | *GALNT2* | 0.101 | (0.016) | 4.7E-08 | 0.050 | 5.4E-10 |
| Alkylating agents | cg14129477 | chr17 | 55,344,858 | 55,344,860 | *NA* | -0.064 | (0.010) | 4.8E-08 | -0.032 | 1.9E-10 |
| Alkylating agents | cg25502475 | chr2 | 71,212,450 | 71,212,452 | *PAIP2B* | -0.059 | (0.009) | 4.8E-08 | -0.029 | 4.2E-10 |
| Alkylating agents | cg10941185 | chr8 | 13,131,006 | 13,131,008 | *DLC1* | -0.060 | (0.010) | 4.9E-08 | -0.026 | 4.7E-08 |
| Alkylating agents | cg05983525 | chr10 | 34,492,301 | 34,492,303 | *PARD3* | 0.060 | (0.010) | 4.9E-08 | 0.026 | 2.5E-08 |
| Alkylating agents | cg01730970 | chr1 | 27,360,582 | 27,360,584 | *MAP3K6* | 0.161 | (0.026) | 5.0E-08 | 0.090 | 1.2E-12 |
| Alkylating agents | cg01641136 | chr3 | 50,607,810 | 50,607,812 | *CISH* | -0.073 | (0.012) | 5.2E-08 | -0.032 | 4.6E-08 |
| Alkylating agents | cg14449059 | chr15 | 58,520,380 | 58,520,382 | *LIPC;RP11-50C13.2* | -0.054 | (0.009) | 5.2E-08 | -0.027 | 2.0E-10 |
| Alkylating agents | cg11242444 | chr11 | 16,883,207 | 16,883,209 | *PLEKHA7* | -0.077 | (0.013) | 5.2E-08 | -0.033 | 1.0E-07 |
| Alkylating agents | cg18603250 | chr11 | 94,767,885 | 94,767,887 | *AMOTL1* | -0.051 | (0.008) | 5.3E-08 | -0.025 | 3.8E-10 |
| Alkylating agents | cg19152802 | chr5 | 110,514,185 | 110,514,187 | *MIR548F3;TMEM232* | -0.084 | (0.014) | 5.4E-08 | -0.043 | 1.6E-10 |
| Alkylating agents | cg23300529 | chr8 | 22,131,498 | 22,131,500 | *HR* | 0.052 | (0.008) | 5.5E-08 | 0.021 | 5.4E-07 |
| Alkylating agents | cg12569593 | chr11 | 126,543,820 | 126,543,822 | *KIRREL3;KIRREL3-AS1* | -0.046 | (0.007) | 5.5E-08 | -0.021 | 6.5E-09 |
| Alkylating agents | cg13251842 | chr22 | 46,112,588 | 46,112,590 | *MIR4763;MIRLET7A3;MIRLET7B;MIRLET7BHG* | 0.076 | (0.012) | 6.1E-08 | 0.030 | 8.1E-07 |
| Alkylating agents | cg10119082 | chr7 | 99,393,002 | 99,393,004 | *ARPC1B;PDAP1* | 0.189 | (0.031) | 6.3E-08 | 0.109 | 4.6E-13 |
| Alkylating agents | cg13910395 | chr22 | 43,577,140 | 43,577,142 | *EFCAB6* | -0.059 | (0.010) | 6.4E-08 | -0.031 | 3.7E-11 |
| Alkylating agents | cg05794482 | chr17 | 67,270,738 | 67,270,740 | *SNORA8* | -0.060 | (0.010) | 6.4E-08 | -0.031 | 9.6E-11 |
| Alkylating agents | cg20534846 | chr9 | 8,188,953 | 8,188,955 | *NA* | -0.064 | (0.010) | 6.5E-08 | -0.022 | 1.7E-05 |
| Alkylating agents | cg25325512 | chr6 | 37,174,443 | 37,174,445 | *PIM1* | -0.078 | (0.013) | 6.7E-08 | -0.038 | 1.4E-09 |
| Alkylating agents | cg09322375 | chr1 | 33,145,797 | 33,145,799 | *TRIM62* | -0.044 | (0.007) | 6.8E-08 | -0.020 | 1.1E-08 |
| Alkylating agents | cg10971669 | chr12 | 8,066,821 | 8,066,823 | *C3AR1* | -0.070 | (0.011) | 6.9E-08 | -0.035 | 2.7E-10 |
| Alkylating agents | cg12385729 | chr1 | 9,183,619 | 9,183,621 | *RP3-510D11.1;RP3-510D11.2* | 0.079 | (0.013) | 7.0E-08 | 0.045 | 5.7E-13 |
| Alkylating agents | cg04537723 | chr20 | 2,283,919 | 2,283,921 | *NA* | -0.062 | (0.010) | 7.3E-08 | -0.027 | 3.3E-08 |
| Alkylating agents | cg16943126 | chr10 | 132,497,609 | 132,497,611 | *NA* | 0.037 | (0.006) | 7.4E-08 | 0.019 | 3.3E-11 |
| Alkylating agents | cg07501635 | chr3 | 53,680,140 | 53,680,142 | *CACNA1D* | 0.137 | (0.022) | 7.4E-08 | 0.065 | 4.1E-09 |
| Alkylating agents | cg22982242 | chr2 | 108,069,673 | 108,069,675 | *NA* | 0.057 | (0.009) | 7.5E-08 | 0.027 | 4.6E-09 |
| Alkylating agents | cg22619104 | chr20 | 1,871,207 | 1,871,209 | *NA* | 0.071 | (0.012) | 7.5E-08 | 0.039 | 1.2E-11 |
| Alkylating agents | cg09294084 | chr13 | 112,992,417 | 112,992,419 | *MCF2L* | 0.073 | (0.012) | 7.5E-08 | 0.042 | 7.9E-13 |
| Alkylating agents | cg09978533 | chr22 | 46,069,279 | 46,069,281 | *MIRLET7BHG;RP6-109B7.4* | -0.056 | (0.009) | 7.6E-08 | -0.032 | 1.5E-12 |
| Alkylating agents | cg24769355 | chr9 | 72,041,875 | 72,041,877 | *NA* | -0.088 | (0.014) | 7.6E-08 | -0.036 | 4.9E-07 |
| Alkylating agents | cg06148273 | chr5 | 83,306,780 | 83,306,782 | *XRCC4* | -0.073 | (0.012) | 7.8E-08 | -0.036 | 6.6E-10 |
| Alkylating agents | cg15272254 | chr20 | 23,051,217 | 23,051,219 | *THBD* | 0.067 | (0.011) | 8.0E-08 | 0.035 | 7.4E-11 |
| Alkylating agents | cg11387340 | chr6 | 166,557,238 | 166,557,240 | *RPS6KA2* | -0.078 | (0.013) | 8.1E-08 | -0.034 | 4.6E-08 |
| Alkylating agents | cg04989440 | chr6 | 125,363,433 | 125,363,435 | *NA* | -0.087 | (0.014) | 8.1E-08 | -0.042 | 2.2E-09 |
| Alkylating agents | cg05657694 | chr3 | 100,635,045 | 100,635,047 | *ADGRG7* | -0.097 | (0.016) | 8.2E-08 | -0.037 | 2.2E-06 |
| Alkylating agents | cg21385305 | chr2 | 23,490,082 | 23,490,084 | *KLHL29* | -0.072 | (0.012) | 8.6E-08 | -0.037 | 8.9E-11 |
| Alkylating agents | cg00248242 | chr2 | 239,927,641 | 239,927,643 | *NDUFA10* | -0.066 | (0.011) | 8.7E-08 | -0.033 | 5.3E-10 |
| Antimetabolites | cg26408927 | chr3 | 53,725,695 | 53,725,697 | *CACNA1D* | -0.248 | (0.017) | 3.5E-36 | -0.124 | 2.5E-40 |
| Antimetabolites | cg24771104 | chr1 | 223,233,619 | 223,233,621 | *SUSD4* | -0.121 | (0.010) | 3.5E-25 | -0.056 | 2.0E-24 |
| Antimetabolites | cg08057120 | chr11 | 97,364,170 | 97,364,172 | *NA* | -0.253 | (0.023) | 3.0E-23 | -0.111 | 8.5E-20 |
| Antimetabolites | cg06347782 | chr9 | 122,228,902 | 122,228,904 | *LHX6* | 0.451 | (0.043) | 6.4E-21 | 0.196 | 5.7E-18 |
| Antimetabolites | cg04201727 | chr9 | 122,228,929 | 122,228,931 | *LHX6* | 0.381 | (0.037) | 3.8E-20 | 0.163 | 8.3E-17 |
| Antimetabolites | cg13386974 | chr9 | 122,229,076 | 122,229,078 | *LHX6* | 0.388 | (0.038) | 9.7E-20 | 0.157 | 8.3E-15 |
| Antimetabolites | cg01351601 | chr5 | 42,547,299 | 42,547,301 | *GHR* | -0.119 | (0.012) | 2.3E-19 | -0.046 | 3.2E-13 |
| Antimetabolites | cg05625471 | chr16 | 31,331,426 | 31,331,428 | *ITGAM* | 0.341 | (0.034) | 3.6E-19 | 0.166 | 3.2E-20 |
| Antimetabolites | cg14634473 | chr10 | 22,816,507 | 22,816,509 | *NA* | -0.101 | (0.010) | 2.4E-18 | -0.039 | 1.4E-12 |
| Antimetabolites | cg14903826 | chr20 | 11,581,468 | 11,581,470 | *NA* | -0.111 | (0.011) | 2.5E-18 | -0.055 | 6.3E-20 |
| Antimetabolites | cg17554494 | chr10 | 107,375,293 | 107,375,295 | *NA* | -0.184 | (0.019) | 4.1E-18 | -0.096 | 8.5E-22 |
| Antimetabolites | cg07677157 | chr12 | 65,657,147 | 65,657,149 | *RP11-221N13.4* | -0.152 | (0.016) | 1.5E-16 | -0.064 | 2.7E-13 |
| Antimetabolites | cg21237939 | chr9 | 122,228,767 | 122,228,769 | *LHX6* | 0.258 | (0.028) | 1.7E-16 | 0.119 | 1.1E-15 |
| Antimetabolites | cg02539128 | chr9 | 122,229,155 | 122,229,157 | *LHX6* | 0.257 | (0.028) | 3.4E-16 | 0.102 | 8.8E-12 |
| Antimetabolites | cg02129236 | chr14 | 38,290,732 | 38,290,734 | *RP11-96D24.1* | -0.145 | (0.016) | 5.6E-16 | -0.083 | 9.6E-23 |
| Antimetabolites | cg09277673 | chr12 | 60,395,015 | 60,395,017 | *NA* | -0.162 | (0.018) | 6.6E-16 | -0.072 | 3.0E-14 |
| Antimetabolites | cg02256631 | chr16 | 31,331,630 | 31,331,632 | *ITGAM* | 0.206 | (0.023) | 4.3E-15 | 0.098 | 3.4E-15 |
| Antimetabolites | cg14639163 | chr2 | 218,873,806 | 218,873,808 | *WNT6* | 0.135 | (0.015) | 5.3E-15 | 0.057 | 3.3E-12 |
| Antimetabolites | cg14200569 | chr1 | 3,135,360 | 3,135,362 | *PRDM16* | 0.147 | (0.017) | 5.3E-15 | 0.053 | 3.7E-09 |
| Antimetabolites | cg23205858 | chr22 | 20,734,517 | 20,734,519 | *NA* | 0.109 | (0.013) | 1.0E-14 | 0.050 | 4.7E-14 |
| Antimetabolites | cg00485681 | chr9 | 122,228,481 | 122,228,483 | *LHX6* | 0.157 | (0.018) | 1.3E-14 | 0.065 | 1.3E-11 |
| Antimetabolites | cg00774728 | chr9 | 122,228,352 | 122,228,354 | *LHX6* | 0.408 | (0.048) | 1.6E-14 | 0.177 | 2.0E-12 |
| Antimetabolites | cg22254104 | chr9 | 122,229,152 | 122,229,154 | *LHX6* | 0.258 | (0.030) | 1.9E-14 | 0.109 | 6.7E-12 |
| Antimetabolites | cg23567872 | chr14 | 38,290,705 | 38,290,707 | *RP11-96D24.1* | -0.167 | (0.020) | 3.6E-14 | -0.092 | 6.8E-19 |
| Antimetabolites | cg20676788 | chr13 | 48,805,654 | 48,805,656 | *NA* | -0.109 | (0.013) | 1.5E-13 | -0.045 | 1.5E-10 |
| Antimetabolites | cg26550214 | chr5 | 34,609,387 | 34,609,389 | *NA* | -0.111 | (0.014) | 3.2E-13 | -0.050 | 3.0E-12 |
| Antimetabolites | cg09146183 | chr22 | 38,214,368 | 38,214,370 | *MAFF* | 0.101 | (0.012) | 4.1E-13 | 0.048 | 2.5E-13 |
| Antimetabolites | cg24833462 | chr2 | 108,053,726 | 108,053,728 | *AC023672.2* | -0.174 | (0.021) | 4.2E-13 | -0.059 | 2.7E-07 |
| Antimetabolites | cg11495719 | chr11 | 124,315,216 | 124,315,218 | *NA* | -0.123 | (0.015) | 6.0E-13 | -0.059 | 2.2E-13 |
| Antimetabolites | cg12943885 | chr5 | 39,650,826 | 39,650,828 | *NA* | -0.087 | (0.011) | 6.9E-13 | -0.038 | 7.1E-11 |
| Antimetabolites | cg20598190 | chr2 | 239,927,506 | 239,927,508 | *NDUFA10* | -0.182 | (0.023) | 1.2E-12 | -0.074 | 1.2E-09 |
| Antimetabolites | cg17434149 | chr9 | 122,228,483 | 122,228,485 | *LHX6* | 0.133 | (0.017) | 2.9E-12 | 0.054 | 1.7E-09 |
| Antimetabolites | cg06225648 | chr9 | 107,213,793 | 107,213,795 | *NA* | -0.077 | (0.010) | 3.8E-12 | -0.026 | 7.2E-07 |
| Antimetabolites | cg03877706 | chr21 | 21,200,312 | 21,200,314 | *NCAM2* | -0.165 | (0.021) | 4.8E-12 | -0.057 | 5.6E-07 |
| Antimetabolites | cg09410512 | chr2 | 223,699,682 | 223,699,684 | *NA* | -0.138 | (0.018) | 5.5E-12 | -0.050 | 1.9E-07 |
| Antimetabolites | cg22351187 | chr12 | 52,192,304 | 52,192,306 | *KRT80* | -0.069 | (0.009) | 7.5E-12 | -0.029 | 1.0E-09 |
| Antimetabolites | cg07386640 | chr14 | 38,290,884 | 38,290,886 | *RP11-96D24.1* | -0.161 | (0.021) | 8.1E-12 | -0.097 | 1.9E-18 |
| Antimetabolites | cg06507987 | chr1 | 27,360,276 | 27,360,278 | *MAP3K6* | 0.095 | (0.012) | 9.1E-12 | 0.033 | 5.2E-07 |
| Antimetabolites | cg08866213 | chr3 | 192,812,987 | 192,812,989 | *MB21D2* | 0.121 | (0.016) | 9.4E-12 | 0.058 | 3.8E-12 |
| Antimetabolites | cg11447971 | chr7 | 116,577,259 | 116,577,261 | *LINC01510* | -0.073 | (0.010) | 1.0E-11 | -0.033 | 5.3E-11 |
| Antimetabolites | cg12769607 | chr3 | 54,035,150 | 54,035,152 | *NA* | -0.084 | (0.011) | 1.3E-11 | -0.043 | 1.1E-13 |
| Antimetabolites | cg15548613 | chr22 | 38,214,787 | 38,214,789 | *MAFF* | 0.111 | (0.015) | 1.4E-11 | 0.055 | 1.9E-12 |
| Antimetabolites | cg17200702 | chr11 | 30,992,376 | 30,992,378 | *DCDC1* | -0.115 | (0.015) | 1.6E-11 | -0.045 | 1.8E-08 |
| Antimetabolites | cg12622958 | chr4 | 41,757,979 | 41,757,981 | *RP11-227F19.1* | -0.081 | (0.011) | 2.3E-11 | -0.043 | 1.2E-13 |
| Antimetabolites | cg14792781 | chr2 | 9,976,114 | 9,976,116 | *GRHL1* | -0.068 | (0.009) | 2.9E-11 | -0.023 | 1.3E-06 |
| Antimetabolites | cg13787850 | chr9 | 99,433,668 | 99,433,670 | *NA* | -0.072 | (0.010) | 6.6E-11 | -0.025 | 1.6E-06 |
| Antimetabolites | cg27093918 | chr16 | 69,530,721 | 69,530,723 | *NA* | -0.110 | (0.015) | 1.0E-10 | -0.029 | 3.9E-04 |
| Antimetabolites | cg09326409 | chr16 | 31,331,131 | 31,331,133 | *ITGAM* | 0.192 | (0.027) | 3.0E-10 | 0.108 | 6.2E-14 |
| Antimetabolites | cg15063366 | chr2 | 227,818,315 | 227,818,317 | *NA* | -0.079 | (0.011) | 3.5E-10 | -0.027 | 6.1E-06 |
| Antimetabolites | cg27478635 | chr20 | 41,003,796 | 41,003,798 | *NA* | -0.123 | (0.018) | 5.0E-10 | -0.047 | 5.6E-07 |
| Antimetabolites | cg27115863 | chr22 | 37,525,632 | 37,525,634 | *NA* | -0.051 | (0.007) | 5.2E-10 | -0.020 | 2.4E-07 |
| Antimetabolites | cg10587886 | chr3 | 8,320,146 | 8,320,148 | *LMCD1-AS1* | -0.119 | (0.017) | 5.9E-10 | -0.028 | 2.0E-03 |
| Antimetabolites | cg24630035 | chr5 | 121,053,366 | 121,053,368 | *NA* | -0.254 | (0.037) | 7.4E-10 | -0.123 | 2.8E-10 |
| Antimetabolites | cg06991118 | chr18 | 9,117,349 | 9,117,351 | *NDUFV2;RP11-143J12.3;RP11-21J18.1* | -0.102 | (0.015) | 1.0E-09 | -0.033 | 2.8E-05 |
| Antimetabolites | cg01504762 | chr2 | 115,647,916 | 115,647,918 | *DPP10* | -0.090 | (0.013) | 1.0E-09 | -0.058 | 6.1E-17 |
| Antimetabolites | cg04839576 | chr9 | 107,213,641 | 107,213,643 | *NA* | -0.096 | (0.014) | 1.1E-09 | -0.034 | 5.7E-06 |
| Antimetabolites | cg14130607 | chr18 | 70,422,334 | 70,422,336 | *NA* | -0.070 | (0.010) | 1.2E-09 | -0.024 | 1.3E-05 |
| Antimetabolites | cg11807280 | chr2 | 66,427,511 | 66,427,513 | *MEIS1-AS3* | -0.144 | (0.021) | 1.3E-09 | -0.054 | 1.4E-06 |
| Antimetabolites | cg04235768 | chr4 | 80,197,188 | 80,197,190 | *PRDM8* | 0.193 | (0.029) | 1.4E-09 | 0.087 | 8.3E-09 |
| Antimetabolites | cg00709171 | chr1 | 115,670,689 | 115,670,691 | *VANGL1* | 0.066 | (0.010) | 2.0E-09 | 0.031 | 1.8E-09 |
| Antimetabolites | cg26342454 | chr4 | 10,033,637 | 10,033,639 | *SLC2A9* | -0.151 | (0.023) | 2.0E-09 | -0.064 | 9.2E-08 |
| Antimetabolites | cg10724969 | chr3 | 33,664,475 | 33,664,477 | *CLASP2* | -0.087 | (0.013) | 2.0E-09 | -0.024 | 5.1E-04 |
| Antimetabolites | cg17302948 | chr11 | 36,149,980 | 36,149,982 | *LDLRAD3* | -0.063 | (0.009) | 2.1E-09 | -0.021 | 3.3E-05 |
| Antimetabolites | cg26225814 | chr14 | 100,019,357 | 100,019,359 | *EVL* | 0.090 | (0.014) | 2.1E-09 | 0.032 | 8.1E-06 |
| Antimetabolites | cg19291696 | chr13 | 27,195,299 | 27,195,301 | *RP11-428O18.6* | -0.074 | (0.011) | 2.6E-09 | -0.031 | 1.4E-07 |
| Antimetabolites | cg07065756 | chr7 | 2,079,704 | 2,079,706 | *MAD1L1* | 0.078 | (0.012) | 3.0E-09 | 0.034 | 3.9E-08 |
| Antimetabolites | cg08774778 | chr5 | 62,082,559 | 62,082,561 | *NA* | -0.081 | (0.012) | 3.3E-09 | -0.025 | 1.7E-04 |
| Antimetabolites | cg03116409 | chr12 | 65,648,773 | 65,648,775 | *RP11-221N13.4* | -0.094 | (0.014) | 3.4E-09 | -0.046 | 8.6E-10 |
| Antimetabolites | cg14856755 | chr13 | 48,805,662 | 48,805,664 | *NA* | -0.072 | (0.011) | 4.1E-09 | -0.025 | 1.9E-05 |
| Antimetabolites | cg19634902 | chr2 | 201,652,689 | 201,652,691 | *MPP4* | -0.067 | (0.010) | 4.1E-09 | -0.031 | 8.6E-09 |
| Antimetabolites | cg13120986 | chr12 | 7,060,707 | 7,060,709 | *C1S* | 0.062 | (0.010) | 4.5E-09 | 0.014 | 4.3E-03 |
| Antimetabolites | cg01329519 | chr5 | 53,303,201 | 53,303,203 | *NA* | -0.106 | (0.016) | 4.5E-09 | -0.056 | 4.4E-11 |
| Antimetabolites | cg26316423 | chr10 | 6,062,173 | 6,062,175 | *IL2RA* | -0.065 | (0.010) | 5.9E-09 | -0.027 | 5.1E-07 |
| Antimetabolites | cg05059566 | chr4 | 80,197,492 | 80,197,494 | *PRDM8* | 0.191 | (0.030) | 6.1E-09 | 0.090 | 7.1E-09 |
| Antimetabolites | cg09294084 | chr13 | 112,992,417 | 112,992,419 | *MCF2L* | 0.080 | (0.012) | 6.3E-09 | 0.032 | 9.1E-07 |
| Antimetabolites | cg15748734 | chr5 | 54,670,217 | 54,670,219 | *NA* | -0.088 | (0.014) | 7.5E-09 | -0.045 | 3.7E-10 |
| Antimetabolites | cg10633815 | chr6 | 92,978,610 | 92,978,612 | *NA* | -0.118 | (0.018) | 8.4E-09 | -0.061 | 2.6E-10 |
| Antimetabolites | cg26038465 | chr22 | 38,214,506 | 38,214,508 | *MAFF* | 0.073 | (0.011) | 8.9E-09 | 0.034 | 1.9E-08 |
| Antimetabolites | cg08006730 | chr19 | 35,109,835 | 35,109,837 | *NA* | 0.064 | (0.010) | 1.0E-08 | 0.031 | 2.5E-09 |
| Antimetabolites | cg01119831 | chr7 | 2,079,972 | 2,079,974 | *MAD1L1* | 0.053 | (0.008) | 1.1E-08 | 0.019 | 1.5E-05 |
| Antimetabolites | cg23264395 | chr1 | 206,922,893 | 206,922,895 | *FCMR* | -0.040 | (0.006) | 1.1E-08 | -0.014 | 1.7E-05 |
| Antimetabolites | cg12882503 | chr13 | 112,992,378 | 112,992,380 | *MCF2L* | 0.077 | (0.012) | 1.2E-08 | 0.026 | 5.7E-05 |
| Antimetabolites | cg11328695 | chr9 | 122,229,068 | 122,229,070 | *LHX6* | 0.170 | (0.027) | 1.2E-08 | 0.074 | 1.8E-07 |
| Antimetabolites | cg13274149 | chr9 | 137,280,940 | 137,280,942 | *TOR4A* | 0.054 | (0.009) | 1.3E-08 | 0.023 | 4.9E-07 |
| Antimetabolites | cg27018912 | chr4 | 80,197,447 | 80,197,449 | *PRDM8* | 0.177 | (0.028) | 1.5E-08 | 0.084 | 1.0E-08 |
| Antimetabolites | cg08662753 | chr9 | 134,669,653 | 134,669,655 | *COL5A1* | 0.069 | (0.011) | 1.6E-08 | 0.024 | 3.0E-05 |
| Antimetabolites | cg22688137 | chr1 | 218,460,155 | 218,460,157 | *NA* | -0.075 | (0.012) | 2.1E-08 | -0.033 | 1.4E-07 |
| Antimetabolites | cg26811385 | chr4 | 149,910,850 | 149,910,852 | *NA* | -0.086 | (0.014) | 2.1E-08 | -0.019 | 8.9E-03 |
| Antimetabolites | cg24277817 | chr19 | 13,024,847 | 13,024,849 | *NFIX* | 0.093 | (0.015) | 2.2E-08 | 0.027 | 6.5E-04 |
| Antimetabolites | cg10694470 | chr4 | 80,197,445 | 80,197,447 | *PRDM8* | 0.149 | (0.024) | 2.5E-08 | 0.071 | 1.6E-08 |
| Antimetabolites | cg03029958 | chr3 | 2,090,634 | 2,090,636 | *RN7SKP144* | -0.093 | (0.015) | 2.6E-08 | -0.046 | 7.6E-09 |
| Antimetabolites | cg14731462 | chr10 | 127,991,164 | 127,991,166 | *PTPRE* | -0.079 | (0.013) | 2.9E-08 | -0.026 | 1.1E-04 |
| Antimetabolites | cg22982242 | chr2 | 108,069,673 | 108,069,675 | *NA* | 0.059 | (0.010) | 3.1E-08 | 0.017 | 6.3E-04 |
| Antimetabolites | cg00280812 | chr12 | 52,192,059 | 52,192,061 | *KRT80* | -0.047 | (0.008) | 3.1E-08 | -0.018 | 4.8E-06 |
| Antimetabolites | cg05308107 | chr13 | 67,228,627 | 67,228,629 | *PCDH9* | 0.096 | (0.016) | 3.4E-08 | 0.049 | 1.9E-09 |
| Antimetabolites | cg13648318 | chr9 | 122,229,128 | 122,229,130 | *LHX6* | 0.152 | (0.025) | 3.6E-08 | 0.060 | 4.0E-06 |
| Antimetabolites | cg17279142 | chr2 | 142,834,321 | 142,834,323 | *NA* | -0.103 | (0.017) | 4.3E-08 | -0.043 | 1.0E-06 |
| Antimetabolites | cg01090659 | chr10 | 44,261,921 | 44,261,923 | *RP11-20J15.2* | -0.043 | (0.007) | 4.4E-08 | -0.022 | 2.0E-09 |
| Antimetabolites | cg20341758 | chr10 | 89,206,463 | 89,206,465 | *CH25H* | -0.078 | (0.013) | 4.6E-08 | -0.034 | 3.5E-07 |
| Antimetabolites | cg01663665 | chr20 | 24,816,582 | 24,816,584 | *NA* | -0.043 | (0.007) | 5.7E-08 | -0.009 | 1.4E-02 |
| Antimetabolites | cg09568818 | chr12 | 20,681,118 | 20,681,120 | *PDE3A* | -0.065 | (0.011) | 5.8E-08 | -0.024 | 3.7E-05 |
| Antimetabolites | cg01730970 | chr1 | 27,360,582 | 27,360,584 | *MAP3K6* | 0.160 | (0.027) | 5.8E-08 | 0.057 | 4.2E-05 |
| Antimetabolites | cg26376241 | chr2 | 65,366,886 | 65,366,888 | *SPRED2* | -0.102 | (0.017) | 5.9E-08 | -0.036 | 7.1E-05 |
| Antimetabolites | cg01440687 | chr14 | 42,541,813 | 42,541,815 | *CTD-2307P3.1* | -0.106 | (0.018) | 5.9E-08 | -0.049 | 9.7E-08 |
| Antimetabolites | cg01751245 | chr2 | 65,366,626 | 65,366,628 | *SPRED2* | -0.089 | (0.015) | 6.1E-08 | -0.029 | 1.8E-04 |
| Antimetabolites | cg13903421 | chr2 | 218,873,991 | 218,873,993 | *WNT6* | 0.136 | (0.023) | 6.8E-08 | 0.045 | 1.4E-04 |
| Antimetabolites | cg12009405 | chr6 | 148,330,194 | 148,330,196 | *SASH1* | -0.110 | (0.018) | 7.9E-08 | -0.039 | 7.4E-05 |
| Antimetabolites | cg19147608 | chr2 | 234,116,192 | 234,116,194 | *NA* | 0.069 | (0.012) | 8.5E-08 | 0.036 | 1.6E-09 |
| Asparaginase enzymes | cg26408927 | chr3 | 53,725,695 | 53,725,697 | *CACNA1D* | -0.357 | (0.020) | 1.9E-55 | -0.143 | 1.1E-49 |
| Asparaginase enzymes | cg24771104 | chr1 | 223,233,619 | 223,233,621 | *SUSD4* | -0.149 | (0.012) | 5.9E-29 | -0.062 | 1.7E-28 |
| Asparaginase enzymes | cg05625471 | chr16 | 31,331,426 | 31,331,428 | *ITGAM* | 0.463 | (0.040) | 2.4E-26 | 0.180 | 1.4E-22 |
| Asparaginase enzymes | cg14903826 | chr20 | 11,581,468 | 11,581,470 | *NA* | -0.145 | (0.013) | 1.3E-23 | -0.053 | 2.4E-18 |
| Asparaginase enzymes | cg06347782 | chr9 | 122,228,902 | 122,228,904 | *LHX6* | 0.552 | (0.050) | 2.0E-23 | 0.220 | 4.1E-21 |
| Asparaginase enzymes | cg12769607 | chr3 | 54,035,150 | 54,035,152 | *NA* | -0.139 | (0.013) | 3.0E-23 | -0.064 | 1.7E-27 |
| Asparaginase enzymes | cg04201727 | chr9 | 122,228,929 | 122,228,931 | *LHX6* | 0.466 | (0.043) | 1.1E-22 | 0.186 | 2.0E-20 |
| Asparaginase enzymes | cg17554494 | chr10 | 107,375,293 | 107,375,295 | *NA* | -0.232 | (0.022) | 2.4E-21 | -0.102 | 5.9E-23 |
| Asparaginase enzymes | cg14639163 | chr2 | 218,873,806 | 218,873,808 | *WNT6* | 0.187 | (0.018) | 2.9E-21 | 0.072 | 1.0E-17 |
| Asparaginase enzymes | cg02129236 | chr14 | 38,290,732 | 38,290,734 | *RP11-96D24.1* | -0.189 | (0.018) | 9.0E-21 | -0.085 | 2.2E-23 |
| Asparaginase enzymes | cg14634473 | chr10 | 22,816,507 | 22,816,509 | *NA* | -0.123 | (0.012) | 1.5E-20 | -0.049 | 1.8E-18 |
| Asparaginase enzymes | cg21237939 | chr9 | 122,228,767 | 122,228,769 | *LHX6* | 0.332 | (0.033) | 4.0E-20 | 0.139 | 4.7E-20 |
| Asparaginase enzymes | cg02256631 | chr16 | 31,331,630 | 31,331,632 | *ITGAM* | 0.273 | (0.027) | 7.2E-20 | 0.103 | 3.5E-16 |
| Asparaginase enzymes | cg23205858 | chr22 | 20,734,517 | 20,734,519 | *NA* | 0.145 | (0.015) | 1.5E-19 | 0.057 | 1.9E-17 |
| Asparaginase enzymes | cg09277673 | chr12 | 60,395,015 | 60,395,017 | *NA* | -0.203 | (0.021) | 3.3E-19 | -0.074 | 1.3E-14 |
| Asparaginase enzymes | cg07677157 | chr12 | 65,657,147 | 65,657,149 | *RP11-221N13.4* | -0.187 | (0.019) | 3.5E-19 | -0.067 | 4.1E-14 |
| Asparaginase enzymes | cg17279142 | chr2 | 142,834,321 | 142,834,323 | *NA* | -0.190 | (0.020) | 7.5E-19 | -0.077 | 1.0E-17 |
| Asparaginase enzymes | cg15548613 | chr22 | 38,214,787 | 38,214,789 | *MAFF* | 0.165 | (0.017) | 1.3E-18 | 0.063 | 2.3E-15 |
| Asparaginase enzymes | cg20676788 | chr13 | 48,805,654 | 48,805,656 | *NA* | -0.146 | (0.015) | 1.6E-18 | -0.062 | 1.6E-18 |
| Asparaginase enzymes | cg01351601 | chr5 | 42,547,299 | 42,547,301 | *GHR* | -0.132 | (0.014) | 3.5E-18 | -0.058 | 9.4E-20 |
| Asparaginase enzymes | cg09146183 | chr22 | 38,214,368 | 38,214,370 | *MAFF* | 0.137 | (0.014) | 3.5E-18 | 0.052 | 6.0E-15 |
| Asparaginase enzymes | cg13386974 | chr9 | 122,229,076 | 122,229,078 | *LHX6* | 0.414 | (0.045) | 2.1E-17 | 0.168 | 2.8E-16 |
| Asparaginase enzymes | cg12622958 | chr4 | 41,757,979 | 41,757,981 | *RP11-227F19.1* | -0.117 | (0.013) | 3.8E-17 | -0.050 | 1.9E-17 |
| Asparaginase enzymes | cg00774728 | chr9 | 122,228,352 | 122,228,354 | *LHX6* | 0.510 | (0.056) | 6.4E-17 | 0.222 | 5.8E-18 |
| Asparaginase enzymes | cg09326409 | chr16 | 31,331,131 | 31,331,133 | *ITGAM* | 0.289 | (0.032) | 9.7E-17 | 0.116 | 1.9E-15 |
| Asparaginase enzymes | cg26550214 | chr5 | 34,609,387 | 34,609,389 | *NA* | -0.142 | (0.016) | 1.7E-16 | -0.060 | 1.5E-16 |
| Asparaginase enzymes | cg06507987 | chr1 | 27,360,276 | 27,360,278 | *MAP3K6* | 0.131 | (0.015) | 3.3E-16 | 0.056 | 6.0E-17 |
| Asparaginase enzymes | cg12140144 | chr1 | 3,067,710 | 3,067,712 | *LINC00982;PRDM16* | 0.330 | (0.037) | 5.5E-16 | 0.140 | 3.8E-16 |
| Asparaginase enzymes | cg00485681 | chr9 | 122,228,481 | 122,228,483 | *LHX6* | 0.188 | (0.021) | 1.1E-15 | 0.080 | 6.8E-16 |
| Asparaginase enzymes | cg26342454 | chr4 | 10,033,637 | 10,033,639 | *SLC2A9* | -0.230 | (0.026) | 1.4E-15 | -0.083 | 8.2E-12 |
| Asparaginase enzymes | cg11447971 | chr7 | 116,577,259 | 116,577,261 | *LINC01510* | -0.095 | (0.011) | 2.0E-15 | -0.042 | 4.3E-17 |
| Asparaginase enzymes | cg18730308 | chr5 | 40,053,020 | 40,053,022 | *LINC00603* | -0.173 | (0.020) | 2.1E-15 | -0.076 | 1.3E-16 |
| Asparaginase enzymes | cg23567872 | chr14 | 38,290,705 | 38,290,707 | *RP11-96D24.1* | -0.195 | (0.023) | 2.4E-15 | -0.087 | 5.8E-17 |
| Asparaginase enzymes | cg03877706 | chr21 | 21,200,312 | 21,200,314 | *NCAM2* | -0.216 | (0.025) | 2.9E-15 | -0.075 | 9.7E-11 |
| Asparaginase enzymes | cg08057120 | chr11 | 97,364,170 | 97,364,172 | *NA* | -0.227 | (0.026) | 3.1E-15 | -0.065 | 8.3E-08 |
| Asparaginase enzymes | cg22254104 | chr9 | 122,229,152 | 122,229,154 | *LHX6* | 0.299 | (0.035) | 9.6E-15 | 0.120 | 1.5E-13 |
| Asparaginase enzymes | cg19825856 | chr2 | 106,885,276 | 106,885,278 | *ST6GAL2* | 0.116 | (0.014) | 1.8E-14 | 0.045 | 1.2E-12 |
| Asparaginase enzymes | cg12960124 | chr4 | 82,100,021 | 82,100,023 | *NA* | -0.125 | (0.015) | 4.6E-14 | -0.055 | 2.0E-15 |
| Asparaginase enzymes | cg07386640 | chr14 | 38,290,884 | 38,290,886 | *RP11-96D24.1* | -0.198 | (0.024) | 5.3E-14 | -0.098 | 7.1E-19 |
| Asparaginase enzymes | cg20598190 | chr2 | 239,927,506 | 239,927,508 | *NDUFA10* | -0.220 | (0.027) | 6.7E-14 | -0.091 | 1.4E-13 |
| Asparaginase enzymes | cg11495719 | chr11 | 124,315,216 | 124,315,218 | *NA* | -0.144 | (0.018) | 7.6E-14 | -0.053 | 1.1E-10 |
| Asparaginase enzymes | cg17200702 | chr11 | 30,992,376 | 30,992,378 | *DCDC1* | -0.146 | (0.018) | 8.0E-14 | -0.054 | 5.9E-11 |
| Asparaginase enzymes | cg27478635 | chr20 | 41,003,796 | 41,003,798 | *NA* | -0.168 | (0.021) | 9.4E-14 | -0.063 | 3.6E-11 |
| Asparaginase enzymes | cg14200569 | chr1 | 3,135,360 | 3,135,362 | *PRDM16* | 0.158 | (0.020) | 1.6E-13 | 0.055 | 1.5E-09 |
| Asparaginase enzymes | cg08618219 | chr15 | 47,412,312 | 47,412,314 | *SEMA6D* | -0.140 | (0.017) | 2.0E-13 | -0.063 | 4.1E-15 |
| Asparaginase enzymes | cg02539128 | chr9 | 122,229,155 | 122,229,157 | *LHX6* | 0.264 | (0.033) | 2.2E-13 | 0.109 | 6.4E-13 |
| Asparaginase enzymes | cg22587479 | chr2 | 218,873,503 | 218,873,505 | *WNT6* | 0.324 | (0.041) | 4.3E-13 | 0.118 | 3.4E-10 |
| Asparaginase enzymes | cg07784975 | chr12 | 23,360,735 | 23,360,737 | *NA* | -0.185 | (0.023) | 4.4E-13 | -0.073 | 1.4E-11 |
| Asparaginase enzymes | cg25618424 | chr1 | 3,072,742 | 3,072,744 | *PRDM16* | 0.112 | (0.014) | 4.5E-13 | 0.052 | 1.3E-15 |
| Asparaginase enzymes | cg03719252 | chr6 | 140,208,293 | 140,208,295 | *NA* | -0.150 | (0.019) | 1.5E-12 | -0.060 | 2.8E-11 |
| Asparaginase enzymes | cg22688137 | chr1 | 218,460,155 | 218,460,157 | *NA* | -0.109 | (0.014) | 1.7E-12 | -0.037 | 7.7E-09 |
| Asparaginase enzymes | cg12438803 | chr5 | 37,071,735 | 37,071,737 | *NA* | -0.096 | (0.012) | 1.8E-12 | -0.046 | 5.6E-16 |
| Asparaginase enzymes | cg03116409 | chr12 | 65,648,773 | 65,648,775 | *RP11-221N13.4* | -0.125 | (0.016) | 2.3E-12 | -0.053 | 2.1E-12 |
| Asparaginase enzymes | cg12882503 | chr13 | 112,992,378 | 112,992,380 | *MCF2L* | 0.107 | (0.014) | 3.6E-12 | 0.039 | 2.8E-09 |
| Asparaginase enzymes | cg19634902 | chr2 | 201,652,689 | 201,652,691 | *MPP4* | -0.089 | (0.012) | 3.9E-12 | -0.037 | 1.1E-11 |
| Asparaginase enzymes | cg22351187 | chr12 | 52,192,304 | 52,192,306 | *KRT80* | -0.078 | (0.010) | 5.0E-12 | -0.028 | 6.2E-09 |
| Asparaginase enzymes | cg00011225 | chr2 | 218,873,591 | 218,873,593 | *WNT6* | 0.373 | (0.050) | 5.8E-12 | 0.147 | 9.9E-11 |
| Asparaginase enzymes | cg15817542 | chr16 | 31,331,734 | 31,331,736 | *ITGAM* | 0.183 | (0.025) | 8.6E-12 | 0.070 | 4.3E-10 |
| Asparaginase enzymes | cg24630035 | chr5 | 121,053,366 | 121,053,368 | *NA* | -0.316 | (0.042) | 9.2E-12 | -0.135 | 4.9E-12 |
| Asparaginase enzymes | cg18509466 | chr1 | 3,071,396 | 3,071,398 | *PRDM16* | 0.078 | (0.011) | 9.3E-12 | 0.035 | 8.2E-13 |
| Asparaginase enzymes | cg11807280 | chr2 | 66,427,511 | 66,427,513 | *MEIS1-AS3* | -0.183 | (0.025) | 1.2E-11 | -0.069 | 1.5E-09 |
| Asparaginase enzymes | cg14792781 | chr2 | 9,976,114 | 9,976,116 | *GRHL1* | -0.078 | (0.010) | 1.4E-11 | -0.031 | 1.7E-10 |
| Asparaginase enzymes | cg24833462 | chr2 | 108,053,726 | 108,053,728 | *AC023672.2* | -0.186 | (0.025) | 1.4E-11 | -0.062 | 8.1E-08 |
| Asparaginase enzymes | cg00303773 | chr17 | 17,849,260 | 17,849,262 | *TOM1L2* | -0.167 | (0.023) | 1.5E-11 | -0.071 | 1.1E-11 |
| Asparaginase enzymes | cg10633815 | chr6 | 92,978,610 | 92,978,612 | *NA* | -0.157 | (0.021) | 1.6E-11 | -0.072 | 1.6E-13 |
| Asparaginase enzymes | cg01431482 | chr1 | 3,072,520 | 3,072,522 | *PRDM16* | 0.079 | (0.011) | 1.6E-11 | 0.036 | 3.2E-13 |
| Asparaginase enzymes | cg01329519 | chr5 | 53,303,201 | 53,303,203 | *NA* | -0.136 | (0.019) | 1.8E-11 | -0.053 | 6.8E-10 |
| Asparaginase enzymes | cg01504762 | chr2 | 115,647,916 | 115,647,918 | *DPP10* | -0.110 | (0.015) | 2.0E-11 | -0.044 | 1.2E-10 |
| Asparaginase enzymes | cg25242471 | chr2 | 218,874,009 | 218,874,011 | *WNT6* | 0.199 | (0.027) | 2.1E-11 | 0.076 | 1.3E-09 |
| Asparaginase enzymes | cg17434149 | chr9 | 122,228,483 | 122,228,485 | *LHX6* | 0.147 | (0.020) | 2.2E-11 | 0.062 | 1.4E-11 |
| Asparaginase enzymes | cg15748734 | chr5 | 54,670,217 | 54,670,219 | *NA* | -0.116 | (0.016) | 2.4E-11 | -0.041 | 1.9E-08 |
| Asparaginase enzymes | cg26038465 | chr22 | 38,214,506 | 38,214,508 | *MAFF* | 0.096 | (0.013) | 2.4E-11 | 0.036 | 3.0E-09 |
| Asparaginase enzymes | cg22266107 | chr8 | 61,719,969 | 61,719,971 | *NA* | 0.098 | (0.013) | 2.4E-11 | 0.039 | 2.1E-10 |
| Asparaginase enzymes | cg06991974 | chr1 | 3,067,653 | 3,067,655 | *LINC00982* | 0.088 | (0.012) | 3.0E-11 | 0.035 | 1.8E-10 |
| Asparaginase enzymes | cg13384284 | chr3 | 28,579,120 | 28,579,122 | *LINC00693* | -0.183 | (0.025) | 3.6E-11 | -0.069 | 3.6E-09 |
| Asparaginase enzymes | cg19291696 | chr13 | 27,195,299 | 27,195,301 | *RP11-428O18.6* | -0.094 | (0.013) | 3.8E-11 | -0.038 | 2.4E-10 |
| Asparaginase enzymes | cg06225648 | chr9 | 107,213,793 | 107,213,795 | *NA* | -0.083 | (0.012) | 4.0E-11 | -0.031 | 4.0E-09 |
| Asparaginase enzymes | cg27115863 | chr22 | 37,525,632 | 37,525,634 | *NA* | -0.061 | (0.009) | 4.3E-11 | -0.025 | 9.0E-11 |
| Asparaginase enzymes | cg21774980 | chr20 | 35,502,719 | 35,502,721 | *CEP250* | 0.052 | (0.007) | 4.6E-11 | 0.022 | 5.9E-11 |
| Asparaginase enzymes | cg08497690 | chr14 | 85,475,266 | 85,475,268 | *CTD-2128A3.2* | -0.124 | (0.017) | 5.7E-11 | -0.053 | 2.1E-11 |
| Asparaginase enzymes | cg01751245 | chr2 | 65,366,626 | 65,366,628 | *SPRED2* | -0.120 | (0.017) | 6.6E-11 | -0.046 | 3.4E-09 |
| Asparaginase enzymes | cg00709171 | chr1 | 115,670,689 | 115,670,691 | *VANGL1* | 0.080 | (0.011) | 7.7E-11 | 0.031 | 4.4E-09 |
| Asparaginase enzymes | cg27093918 | chr16 | 69,530,721 | 69,530,723 | *NA* | -0.125 | (0.018) | 9.4E-11 | -0.045 | 2.4E-08 |
| Asparaginase enzymes | cg26376241 | chr2 | 65,366,886 | 65,366,888 | *SPRED2* | -0.137 | (0.019) | 9.8E-11 | -0.054 | 2.0E-09 |
| Asparaginase enzymes | cg11532433 | chr2 | 24,512,428 | 24,512,430 | *NCOA1* | 0.091 | (0.013) | 1.1E-10 | 0.039 | 6.8E-11 |
| Asparaginase enzymes | cg11686251 | chr9 | 131,598,498 | 131,598,500 | *RAPGEF1* | 0.094 | (0.013) | 1.1E-10 | 0.035 | 8.9E-09 |
| Asparaginase enzymes | cg15063366 | chr2 | 227,818,315 | 227,818,317 | *NA* | -0.092 | (0.013) | 1.2E-10 | -0.038 | 2.7E-10 |
| Asparaginase enzymes | cg08006730 | chr19 | 35,109,835 | 35,109,837 | *NA* | 0.079 | (0.011) | 1.4E-10 | 0.034 | 8.5E-11 |
| Asparaginase enzymes | cg14731462 | chr10 | 127,991,164 | 127,991,166 | *PTPRE* | -0.103 | (0.015) | 1.4E-10 | -0.034 | 6.3E-07 |
| Asparaginase enzymes | cg12943885 | chr5 | 39,650,826 | 39,650,828 | *NA* | -0.088 | (0.013) | 1.7E-10 | -0.036 | 5.1E-10 |
| Asparaginase enzymes | cg03684356 | chr20 | 57,678,706 | 57,678,708 | *PMEPA1* | 0.113 | (0.016) | 1.9E-10 | 0.041 | 3.7E-08 |
| Asparaginase enzymes | cg21050234 | chr18 | 10,726,519 | 10,726,521 | *PIEZO2;RP11-21G15.1* | 0.141 | (0.020) | 1.9E-10 | 0.059 | 2.6E-10 |
| Asparaginase enzymes | cg10353174 | chr8 | 105,788,523 | 105,788,525 | *ZFPM2;ZFPM2-AS1* | -0.122 | (0.018) | 2.5E-10 | -0.051 | 2.4E-10 |
| Asparaginase enzymes | cg07086679 | chr7 | 140,316,321 | 140,316,323 | *SLC37A3* | -0.088 | (0.013) | 3.9E-10 | -0.039 | 2.6E-11 |
| Asparaginase enzymes | cg07914614 | chr1 | 61,606,795 | 61,606,797 | *NA* | 0.096 | (0.014) | 4.0E-10 | 0.039 | 1.4E-09 |
| Asparaginase enzymes | cg08866213 | chr3 | 192,812,987 | 192,812,989 | *MB21D2* | 0.124 | (0.018) | 4.0E-10 | 0.045 | 7.0E-08 |
| Asparaginase enzymes | cg09294084 | chr13 | 112,992,417 | 112,992,419 | *MCF2L* | 0.095 | (0.014) | 5.8E-10 | 0.035 | 6.0E-08 |
| Asparaginase enzymes | cg09978353 | chr17 | 74,763,142 | 74,763,144 | *SLC9A3R1* | 0.076 | (0.011) | 6.7E-10 | 0.031 | 4.1E-09 |
| Asparaginase enzymes | cg27024924 | chr6 | 21,186,285 | 21,186,287 | *CDKAL1* | -0.130 | (0.019) | 6.9E-10 | -0.053 | 1.6E-09 |
| Asparaginase enzymes | cg26316423 | chr10 | 6,062,173 | 6,062,175 | *IL2RA* | -0.078 | (0.012) | 7.1E-10 | -0.028 | 1.3E-07 |
| Asparaginase enzymes | cg01004017 | chr2 | 120,899,770 | 120,899,772 | *GLI2* | 0.110 | (0.016) | 7.6E-10 | 0.034 | 6.5E-06 |
| Asparaginase enzymes | cg20970886 | chr3 | 192,850,924 | 192,850,926 | *MB21D2* | -0.080 | (0.012) | 9.0E-10 | -0.025 | 4.0E-06 |
| Asparaginase enzymes | cg14856755 | chr13 | 48,805,662 | 48,805,664 | *NA* | -0.084 | (0.013) | 1.0E-09 | -0.030 | 1.5E-07 |
| Asparaginase enzymes | cg23575571 | chr1 | 120,262,998 | 120,263,000 | *NA* | 0.079 | (0.012) | 1.0E-09 | 0.035 | 7.8E-11 |
| Asparaginase enzymes | cg21150701 | chr2 | 120,899,241 | 120,899,243 | *GLI2* | 0.073 | (0.011) | 1.1E-09 | 0.025 | 5.5E-07 |
| Asparaginase enzymes | cg21915904 | chr17 | 2,926,475 | 2,926,477 | *RAP1GAP2* | 0.070 | (0.011) | 1.2E-09 | 0.028 | 1.4E-08 |
| Asparaginase enzymes | cg13286857 | chr5 | 66,822,120 | 66,822,122 | *MAST4* | -0.067 | (0.010) | 1.3E-09 | -0.023 | 8.6E-07 |
| Asparaginase enzymes | cg05308107 | chr13 | 67,228,627 | 67,228,629 | *PCDH9* | 0.118 | (0.018) | 1.3E-09 | 0.043 | 1.4E-07 |
| Asparaginase enzymes | cg04507642 | chr12 | 51,419,879 | 51,419,881 | *SLC4A8* | 0.073 | (0.011) | 1.4E-09 | 0.029 | 1.1E-08 |
| Asparaginase enzymes | cg13274149 | chr9 | 137,280,940 | 137,280,942 | *TOR4A* | 0.065 | (0.010) | 1.4E-09 | 0.027 | 2.3E-09 |
| Asparaginase enzymes | cg14739080 | chr12 | 7,772,932 | 7,772,934 | *NANOGNB* | 0.057 | (0.009) | 1.5E-09 | 0.022 | 4.9E-08 |
| Asparaginase enzymes | cg13129556 | chr20 | 49,823,682 | 49,823,684 | *SLC9A8* | -0.089 | (0.014) | 2.0E-09 | -0.032 | 1.9E-07 |
| Asparaginase enzymes | cg14130607 | chr18 | 70,422,334 | 70,422,336 | *NA* | -0.078 | (0.012) | 2.1E-09 | -0.031 | 9.9E-09 |
| Asparaginase enzymes | cg01730970 | chr1 | 27,360,582 | 27,360,584 | *MAP3K6* | 0.201 | (0.031) | 2.2E-09 | 0.093 | 3.3E-11 |
| Asparaginase enzymes | cg03747456 | chr12 | 52,192,342 | 52,192,344 | *KRT80* | -0.046 | (0.007) | 2.4E-09 | -0.019 | 3.8E-09 |
| Asparaginase enzymes | cg19743666 | chr2 | 30,148,617 | 30,148,619 | *YPEL5* | -0.118 | (0.018) | 2.4E-09 | -0.044 | 9.5E-08 |
| Asparaginase enzymes | cg19876649 | chr18 | 3,220,274 | 3,220,276 | *MYOM1;RP13-270P17.2* | -0.086 | (0.013) | 2.7E-09 | -0.033 | 6.2E-08 |
| Asparaginase enzymes | cg12628061 | chr1 | 55,988,056 | 55,988,058 | *NA* | 0.114 | (0.018) | 2.8E-09 | 0.046 | 1.1E-08 |
| Asparaginase enzymes | cg14694639 | chr1 | 147,567,897 | 147,567,899 | *BCL9* | 0.057 | (0.009) | 3.3E-09 | 0.021 | 3.4E-07 |
| Asparaginase enzymes | cg17038235 | chr5 | 172,943,520 | 172,943,522 | *ERGIC1* | 0.069 | (0.011) | 3.5E-09 | 0.028 | 2.0E-08 |
| Asparaginase enzymes | cg10137837 | chr17 | 7,023,422 | 7,023,424 | *BCL6B* | -0.036 | (0.006) | 4.8E-09 | -0.014 | 5.0E-08 |
| Asparaginase enzymes | cg11356990 | chr2 | 241,030,698 | 241,030,700 | *AC005237.4;SNED1* | 0.046 | (0.007) | 5.2E-09 | 0.017 | 3.6E-07 |
| Asparaginase enzymes | cg02560069 | chr15 | 39,133,413 | 39,133,415 | *RP11-624L4.1* | 0.079 | (0.012) | 5.4E-09 | 0.034 | 3.3E-09 |
| Asparaginase enzymes | cg25644567 | chr2 | 65,366,786 | 65,366,788 | *SPRED2* | -0.080 | (0.013) | 5.8E-09 | -0.028 | 9.7E-07 |
| Asparaginase enzymes | cg09410512 | chr2 | 223,699,682 | 223,699,684 | *NA* | -0.132 | (0.021) | 6.0E-09 | -0.050 | 1.2E-07 |
| Asparaginase enzymes | cg20996351 | chr1 | 27,360,740 | 27,360,742 | *MAP3K6* | 0.274 | (0.043) | 6.3E-09 | 0.114 | 1.0E-08 |
| Asparaginase enzymes | cg09992085 | chr19 | 41,456,428 | 41,456,430 | *PCAT19* | -0.085 | (0.013) | 6.7E-09 | -0.035 | 1.1E-08 |
| Asparaginase enzymes | cg01012330 | chr3 | 192,916,269 | 192,916,271 | *MB21D2* | 0.073 | (0.012) | 7.0E-09 | 0.023 | 1.8E-05 |
| Asparaginase enzymes | cg20308138 | chr5 | 67,301,693 | 67,301,695 | *NA* | -0.050 | (0.008) | 7.3E-09 | -0.018 | 5.0E-07 |
| Asparaginase enzymes | cg26547821 | chr1 | 71,366,080 | 71,366,082 | *ZRANB2-AS2* | -0.144 | (0.023) | 7.9E-09 | -0.064 | 1.3E-09 |
| Asparaginase enzymes | cg13903421 | chr2 | 218,873,991 | 218,873,993 | *WNT6* | 0.164 | (0.026) | 8.3E-09 | 0.058 | 1.7E-06 |
| Asparaginase enzymes | cg20404850 | chr9 | 96,612,377 | 96,612,379 | *CDC14B* | 0.134 | (0.021) | 8.5E-09 | 0.055 | 1.5E-08 |
| Asparaginase enzymes | cg22943590 | chr2 | 66,421,664 | 66,421,666 | *NA* | -0.153 | (0.025) | 9.7E-09 | -0.050 | 8.0E-06 |
| Asparaginase enzymes | cg19546057 | chr11 | 128,906,320 | 128,906,322 | *C11orf45;KCNJ5* | 0.053 | (0.008) | 1.1E-08 | 0.022 | 2.5E-08 |
| Asparaginase enzymes | cg23207077 | chr1 | 970,607 | 970,609 | *PLEKHN1* | 0.064 | (0.010) | 1.1E-08 | 0.021 | 9.1E-06 |
| Asparaginase enzymes | cg11328695 | chr9 | 122,229,068 | 122,229,070 | *LHX6* | 0.193 | (0.031) | 1.1E-08 | 0.077 | 5.4E-08 |
| Asparaginase enzymes | cg06991118 | chr18 | 9,117,349 | 9,117,351 | *NDUFV2;RP11-143J12.3;RP11-21J18.1* | -0.107 | (0.017) | 1.1E-08 | -0.040 | 4.9E-07 |
| Asparaginase enzymes | cg10541181 | chr12 | 23,559,658 | 23,559,660 | *SOX5* | -0.128 | (0.021) | 1.2E-08 | -0.055 | 4.6E-09 |
| Asparaginase enzymes | cg06744585 | chr19 | 13,024,993 | 13,024,995 | *NFIX* | 0.201 | (0.032) | 1.3E-08 | 0.058 | 1.1E-04 |
| Asparaginase enzymes | cg09884146 | chr2 | 65,366,773 | 65,366,775 | *SPRED2* | -0.066 | (0.011) | 1.5E-08 | -0.025 | 6.5E-07 |
| Asparaginase enzymes | cg02205744 | chr15 | 62,957,201 | 62,957,203 | *NA* | -0.060 | (0.010) | 1.6E-08 | -0.029 | 1.0E-10 |
| Asparaginase enzymes | cg06148273 | chr5 | 83,306,780 | 83,306,782 | *XRCC4* | -0.086 | (0.014) | 1.7E-08 | -0.038 | 3.3E-09 |
| Asparaginase enzymes | cg01792230 | chr9 | 96,612,375 | 96,612,377 | *CDC14B* | 0.120 | (0.020) | 1.8E-08 | 0.052 | 7.5E-09 |
| Asparaginase enzymes | cg08191886 | chr6 | 145,148,612 | 145,148,614 | *NA* | 0.079 | (0.013) | 1.8E-08 | 0.033 | 2.7E-08 |
| Asparaginase enzymes | cg07946633 | chr1 | 3,067,680 | 3,067,682 | *LINC00982;PRDM16* | 0.062 | (0.010) | 2.1E-08 | 0.026 | 4.3E-08 |
| Asparaginase enzymes | cg26225814 | chr14 | 100,019,357 | 100,019,359 | *EVL* | 0.095 | (0.016) | 2.3E-08 | 0.035 | 1.3E-06 |
| Asparaginase enzymes | cg01282174 | chr11 | 119,759,434 | 119,759,436 | *NA* | 0.110 | (0.018) | 2.8E-08 | 0.044 | 1.2E-07 |
| Asparaginase enzymes | cg05331731 | chr18 | 11,147,146 | 11,147,148 | *PIEZO2* | 0.134 | (0.022) | 2.8E-08 | 0.040 | 7.1E-05 |
| Asparaginase enzymes | cg18398267 | chr16 | 57,045,270 | 57,045,272 | *NLRC5* | 0.058 | (0.010) | 3.0E-08 | 0.022 | 6.6E-07 |
| Asparaginase enzymes | cg03505273 | chr11 | 23,805,076 | 23,805,078 | *RP11-945A11.2* | -0.118 | (0.020) | 3.0E-08 | -0.048 | 9.4E-08 |
| Asparaginase enzymes | cg26581012 | chr19 | 13,024,518 | 13,024,520 | *NFIX* | 0.104 | (0.017) | 3.2E-08 | 0.033 | 4.2E-05 |
| Asparaginase enzymes | cg19969083 | chr13 | 106,100,837 | 106,100,839 | *NA* | 0.070 | (0.012) | 3.2E-08 | 0.024 | 6.5E-06 |
| Asparaginase enzymes | cg25896883 | chr2 | 189,407,943 | 189,407,945 | *NA* | -0.098 | (0.016) | 3.2E-08 | -0.047 | 3.1E-10 |
| Asparaginase enzymes | cg21890448 | chr1 | 115,671,054 | 115,671,056 | *VANGL1* | 0.063 | (0.011) | 3.7E-08 | 0.026 | 4.4E-08 |
| Asparaginase enzymes | cg19716264 | chr1 | 245,018,847 | 245,018,849 | *EFCAB2* | 0.082 | (0.014) | 4.2E-08 | 0.033 | 1.4E-07 |
| Asparaginase enzymes | cg07710094 | chr8 | 129,267,705 | 129,267,707 | *NA* | -0.091 | (0.015) | 4.8E-08 | -0.046 | 3.6E-11 |
| Asparaginase enzymes | cg07944953 | chr8 | 26,458,151 | 26,458,153 | *BNIP3L* | 0.105 | (0.018) | 4.9E-08 | 0.038 | 2.6E-06 |
| Asparaginase enzymes | cg25586488 | chr10 | 44,261,885 | 44,261,887 | *RP11-20J15.2* | -0.056 | (0.009) | 5.5E-08 | -0.021 | 1.2E-06 |
| Asparaginase enzymes | cg25925177 | chr11 | 64,342,705 | 64,342,707 | *CCDC88B;MIR7155* | 0.072 | (0.012) | 6.1E-08 | 0.028 | 5.2E-07 |
| Asparaginase enzymes | cg03231199 | chr5 | 8,634,220 | 8,634,222 | *NA* | 0.135 | (0.023) | 6.9E-08 | 0.064 | 1.1E-09 |
| Asparaginase enzymes | cg17723693 | chr14 | 84,442,754 | 84,442,756 | *NA* | -0.101 | (0.017) | 7.0E-08 | -0.045 | 1.3E-08 |
| Asparaginase enzymes | cg05713399 | chr21 | 16,302,893 | 16,302,895 | *AP001172.2;MIR99AHG* | 0.100 | (0.017) | 7.0E-08 | 0.037 | 3.2E-06 |
| Asparaginase enzymes | cg11070989 | chr6 | 113,447,300 | 113,447,302 | *NA* | -0.118 | (0.020) | 7.2E-08 | -0.041 | 8.1E-06 |
| Asparaginase enzymes | cg06867863 | chr2 | 65,932,195 | 65,932,197 | *AC074391.1* | 0.068 | (0.012) | 7.8E-08 | 0.030 | 1.3E-08 |
| Corticosteroids | cg26408927 | chr3 | 53,725,695 | 53,725,697 | *CACNA1D* | -0.157 | (0.017) | 3.4E-16 | -0.056 | 8.3E-11 |
| Corticosteroids | cg09932507 | chr17 | 49,566,047 | 49,566,049 | *RP5-1029K10.2;RP5-1029K10.4* | -0.073 | (0.010) | 1.8E-11 | -0.039 | 8.2E-16 |
| Corticosteroids | cg08057120 | chr11 | 97,364,170 | 97,364,172 | *NA* | -0.161 | (0.022) | 9.2E-11 | -0.039 | 4.5E-04 |
| Corticosteroids | cg07386640 | chr14 | 38,290,884 | 38,290,886 | *RP11-96D24.1* | -0.142 | (0.020) | 3.6E-10 | -0.078 | 4.5E-15 |
| Corticosteroids | cg02129236 | chr14 | 38,290,732 | 38,290,734 | *RP11-96D24.1* | -0.108 | (0.015) | 4.3E-10 | -0.055 | 6.3E-13 |
| Corticosteroids | cg23567872 | chr14 | 38,290,705 | 38,290,707 | *RP11-96D24.1* | -0.122 | (0.019) | 8.1E-09 | -0.063 | 2.2E-11 |
| Corticosteroids | cg22351187 | chr12 | 52,192,304 | 52,192,306 | *KRT80* | -0.054 | (0.009) | 4.8E-08 | -0.023 | 2.3E-07 |
| Corticosteroids | cg07677157 | chr12 | 65,657,147 | 65,657,149 | *RP11-221N13.4* | -0.095 | (0.016) | 8.6E-08 | -0.027 | 8.5E-04 |
| Epipodophyllotoxins | cg26408927 | chr3 | 53,725,695 | 53,725,697 | *CACNA1D* | -0.270 | (0.018) | 2.0E-43 | -0.095 | 1.6E-28 |
| Epipodophyllotoxins | cg21237939 | chr9 | 122,228,767 | 122,228,769 | *LHX6* | 0.342 | (0.029) | 5.6E-28 | 0.106 | 7.8E-15 |
| Epipodophyllotoxins | cg14634473 | chr10 | 22,816,507 | 22,816,509 | *NA* | -0.123 | (0.010) | 4.0E-27 | -0.040 | 2.1E-15 |
| Epipodophyllotoxins | cg17554494 | chr10 | 107,375,293 | 107,375,295 | *NA* | -0.226 | (0.019) | 1.2E-26 | -0.084 | 1.3E-19 |
| Epipodophyllotoxins | cg00485681 | chr9 | 122,228,481 | 122,228,483 | *LHX6* | 0.212 | (0.018) | 9.0E-26 | 0.067 | 3.5E-14 |
| Epipodophyllotoxins | cg01351601 | chr5 | 42,547,299 | 42,547,301 | *GHR* | -0.136 | (0.012) | 1.4E-25 | -0.045 | 1.6E-15 |
| Epipodophyllotoxins | cg06347782 | chr9 | 122,228,902 | 122,228,904 | *LHX6* | 0.498 | (0.044) | 2.3E-25 | 0.161 | 1.5E-14 |
| Epipodophyllotoxins | cg03877706 | chr21 | 21,200,312 | 21,200,314 | *NCAM2* | -0.248 | (0.022) | 2.3E-25 | -0.074 | 9.4E-13 |
| Epipodophyllotoxins | cg22688137 | chr1 | 218,460,155 | 218,460,157 | *NA* | -0.139 | (0.012) | 2.8E-25 | -0.042 | 6.6E-13 |
| Epipodophyllotoxins | cg06507987 | chr1 | 27,360,276 | 27,360,278 | *MAP3K6* | 0.145 | (0.013) | 3.0E-25 | 0.051 | 1.1E-16 |
| Epipodophyllotoxins | cg12622958 | chr4 | 41,757,979 | 41,757,981 | *RP11-227F19.1* | -0.124 | (0.011) | 9.0E-25 | -0.045 | 3.6E-17 |
| Epipodophyllotoxins | cg12140144 | chr1 | 3,067,710 | 3,067,712 | *LINC00982;PRDM16* | 0.367 | (0.033) | 9.6E-25 | 0.132 | 2.7E-17 |
| Epipodophyllotoxins | cg20598190 | chr2 | 239,927,506 | 239,927,508 | *NDUFA10* | -0.256 | (0.023) | 5.7E-24 | -0.084 | 3.3E-14 |
| Epipodophyllotoxins | cg00774728 | chr9 | 122,228,352 | 122,228,354 | *LHX6* | 0.529 | (0.048) | 1.2E-23 | 0.165 | 9.5E-13 |
| Epipodophyllotoxins | cg26342454 | chr4 | 10,033,637 | 10,033,639 | *SLC2A9* | -0.252 | (0.023) | 1.4E-23 | -0.075 | 7.0E-12 |
| Epipodophyllotoxins | cg14639163 | chr2 | 218,873,806 | 218,873,808 | *WNT6* | 0.173 | (0.016) | 1.4E-23 | 0.052 | 8.6E-12 |
| Epipodophyllotoxins | cg17279142 | chr2 | 142,834,321 | 142,834,323 | *NA* | -0.188 | (0.017) | 1.6E-23 | -0.069 | 3.3E-17 |
| Epipodophyllotoxins | cg17200702 | chr11 | 30,992,376 | 30,992,378 | *DCDC1* | -0.167 | (0.016) | 6.1E-23 | -0.048 | 6.1E-11 |
| Epipodophyllotoxins | cg04201727 | chr9 | 122,228,929 | 122,228,931 | *LHX6* | 0.402 | (0.038) | 1.8E-22 | 0.130 | 5.5E-13 |
| Epipodophyllotoxins | cg05625471 | chr16 | 31,331,426 | 31,331,428 | *ITGAM* | 0.369 | (0.035) | 2.1E-22 | 0.112 | 1.5E-11 |
| Epipodophyllotoxins | cg24833462 | chr2 | 108,053,726 | 108,053,728 | *AC023672.2* | -0.227 | (0.022) | 8.0E-22 | -0.061 | 5.6E-09 |
| Epipodophyllotoxins | cg24771104 | chr1 | 223,233,619 | 223,233,621 | *SUSD4* | -0.111 | (0.011) | 1.0E-21 | -0.045 | 3.6E-19 |
| Epipodophyllotoxins | cg07086679 | chr7 | 140,316,321 | 140,316,323 | *SLC37A3* | -0.117 | (0.011) | 2.4E-21 | -0.041 | 4.6E-14 |
| Epipodophyllotoxins | cg09326409 | chr16 | 31,331,131 | 31,331,133 | *ITGAM* | 0.287 | (0.028) | 3.6E-21 | 0.080 | 1.7E-09 |
| Epipodophyllotoxins | cg15548613 | chr22 | 38,214,787 | 38,214,789 | *MAFF* | 0.155 | (0.015) | 5.0E-21 | 0.050 | 3.6E-12 |
| Epipodophyllotoxins | cg17434149 | chr9 | 122,228,483 | 122,228,485 | *LHX6* | 0.176 | (0.017) | 1.4E-20 | 0.053 | 1.2E-10 |
| Epipodophyllotoxins | cg22254104 | chr9 | 122,229,152 | 122,229,154 | *LHX6* | 0.309 | (0.031) | 2.6E-20 | 0.099 | 1.5E-11 |
| Epipodophyllotoxins | cg00303773 | chr17 | 17,849,260 | 17,849,262 | *TOM1L2* | -0.199 | (0.020) | 4.9E-20 | -0.070 | 9.5E-14 |
| Epipodophyllotoxins | cg13384284 | chr3 | 28,579,120 | 28,579,122 | *LINC00693* | -0.221 | (0.022) | 5.6E-20 | -0.061 | 6.0E-09 |
| Epipodophyllotoxins | cg10119082 | chr7 | 99,393,002 | 99,393,004 | *ARPC1B;PDAP1* | 0.319 | (0.032) | 7.9E-20 | 0.092 | 1.8E-09 |
| Epipodophyllotoxins | cg13386974 | chr9 | 122,229,076 | 122,229,078 | *LHX6* | 0.377 | (0.039) | 4.9E-19 | 0.124 | 1.9E-11 |
| Epipodophyllotoxins | cg02256631 | chr16 | 31,331,630 | 31,331,632 | *ITGAM* | 0.233 | (0.024) | 5.3E-19 | 0.068 | 2.7E-09 |
| Epipodophyllotoxins | cg19664938 | chr5 | 6,493,144 | 6,493,146 | *UBE2QL1* | -0.093 | (0.010) | 1.0E-18 | -0.026 | 2.2E-08 |
| Epipodophyllotoxins | cg10633815 | chr6 | 92,978,610 | 92,978,612 | *NA* | -0.178 | (0.019) | 1.3E-18 | -0.058 | 4.6E-11 |
| Epipodophyllotoxins | cg11807280 | chr2 | 66,427,511 | 66,427,513 | *MEIS1-AS3* | -0.208 | (0.022) | 1.5E-18 | -0.075 | 2.8E-13 |
| Epipodophyllotoxins | cg02539128 | chr9 | 122,229,155 | 122,229,157 | *LHX6* | 0.273 | (0.029) | 2.1E-18 | 0.087 | 1.4E-10 |
| Epipodophyllotoxins | cg07946633 | chr1 | 3,067,680 | 3,067,682 | *LINC00982;PRDM16* | 0.085 | (0.009) | 4.3E-18 | 0.028 | 6.8E-11 |
| Epipodophyllotoxins | cg12769607 | chr3 | 54,035,150 | 54,035,152 | *NA* | -0.106 | (0.011) | 6.7E-18 | -0.038 | 2.6E-12 |
| Epipodophyllotoxins | cg26550214 | chr5 | 34,609,387 | 34,609,389 | *NA* | -0.129 | (0.014) | 8.4E-18 | -0.042 | 2.5E-10 |
| Epipodophyllotoxins | cg09146183 | chr22 | 38,214,368 | 38,214,370 | *MAFF* | 0.118 | (0.013) | 1.1E-17 | 0.040 | 5.3E-11 |
| Epipodophyllotoxins | cg18730308 | chr5 | 40,053,020 | 40,053,022 | *LINC00603* | -0.165 | (0.018) | 1.2E-17 | -0.064 | 3.2E-14 |
| Epipodophyllotoxins | cg12960124 | chr4 | 82,100,021 | 82,100,023 | *NA* | -0.125 | (0.014) | 1.2E-17 | -0.042 | 6.1E-11 |
| Epipodophyllotoxins | cg14903826 | chr20 | 11,581,468 | 11,581,470 | *NA* | -0.108 | (0.012) | 1.3E-17 | -0.038 | 5.1E-12 |
| Epipodophyllotoxins | cg27478635 | chr20 | 41,003,796 | 41,003,798 | *NA* | -0.168 | (0.018) | 1.7E-17 | -0.058 | 2.2E-11 |
| Epipodophyllotoxins | cg09358973 | chr1 | 201,648,901 | 201,648,903 | *NAV1* | 0.208 | (0.023) | 2.2E-17 | 0.058 | 5.7E-08 |
| Epipodophyllotoxins | cg22587479 | chr2 | 218,873,503 | 218,873,505 | *WNT6* | 0.333 | (0.036) | 2.4E-17 | 0.096 | 2.5E-08 |
| Epipodophyllotoxins | cg00011225 | chr2 | 218,873,591 | 218,873,593 | *WNT6* | 0.403 | (0.044) | 2.5E-17 | 0.120 | 8.6E-09 |
| Epipodophyllotoxins | cg06991974 | chr1 | 3,067,653 | 3,067,655 | *LINC00982* | 0.098 | (0.011) | 2.7E-17 | 0.033 | 5.4E-11 |
| Epipodophyllotoxins | cg27192248 | chr15 | 64,993,330 | 64,993,332 | *NA* | -0.231 | (0.025) | 5.3E-17 | -0.067 | 2.9E-08 |
| Epipodophyllotoxins | cg25242471 | chr2 | 218,874,009 | 218,874,011 | *WNT6* | 0.219 | (0.024) | 5.4E-17 | 0.064 | 1.6E-08 |
| Epipodophyllotoxins | cg01730970 | chr1 | 27,360,582 | 27,360,584 | *MAP3K6* | 0.246 | (0.027) | 6.6E-17 | 0.089 | 5.2E-12 |
| Epipodophyllotoxins | cg14200569 | chr1 | 3,135,360 | 3,135,362 | *PRDM16* | 0.155 | (0.017) | 7.7E-17 | 0.039 | 1.4E-06 |
| Epipodophyllotoxins | cg08883485 | chr1 | 201,650,658 | 201,650,660 | *NAV1* | 0.197 | (0.022) | 8.3E-17 | 0.059 | 1.3E-08 |
| Epipodophyllotoxins | cg10541181 | chr12 | 23,559,658 | 23,559,660 | *SOX5* | -0.164 | (0.018) | 9.8E-17 | -0.048 | 2.5E-08 |
| Epipodophyllotoxins | cg15748734 | chr5 | 54,670,217 | 54,670,219 | *NA* | -0.126 | (0.014) | 1.2E-16 | -0.033 | 8.6E-07 |
| Epipodophyllotoxins | cg23205858 | chr22 | 20,734,517 | 20,734,519 | *NA* | 0.116 | (0.013) | 1.3E-16 | 0.037 | 9.5E-10 |
| Epipodophyllotoxins | cg06268875 | chr18 | 11,147,385 | 11,147,387 | *PIEZO2* | 0.133 | (0.015) | 1.9E-16 | 0.038 | 9.4E-08 |
| Epipodophyllotoxins | cg13511623 | chr9 | 123,716,678 | 123,716,680 | *DENND1A* | -0.153 | (0.017) | 2.7E-16 | -0.053 | 7.1E-11 |
| Epipodophyllotoxins | cg07677157 | chr12 | 65,657,147 | 65,657,149 | *RP11-221N13.4* | -0.149 | (0.017) | 3.7E-16 | -0.049 | 8.1E-10 |
| Epipodophyllotoxins | cg13274149 | chr9 | 137,280,940 | 137,280,942 | *TOR4A* | 0.077 | (0.009) | 3.8E-16 | 0.028 | 1.3E-11 |
| Epipodophyllotoxins | cg07784975 | chr12 | 23,360,735 | 23,360,737 | *NA* | -0.183 | (0.021) | 4.1E-16 | -0.060 | 1.1E-09 |
| Epipodophyllotoxins | cg12882503 | chr13 | 112,992,378 | 112,992,380 | *MCF2L* | 0.110 | (0.012) | 4.2E-16 | 0.027 | 3.6E-06 |
| Epipodophyllotoxins | cg14130607 | chr18 | 70,422,334 | 70,422,336 | *NA* | -0.092 | (0.010) | 5.8E-16 | -0.028 | 9.1E-09 |
| Epipodophyllotoxins | cg07710094 | chr8 | 129,267,705 | 129,267,707 | *NA* | -0.119 | (0.014) | 6.6E-16 | -0.045 | 1.9E-12 |
| Epipodophyllotoxins | cg11495719 | chr11 | 124,315,216 | 124,315,218 | *NA* | -0.136 | (0.016) | 1.0E-15 | -0.045 | 7.7E-10 |
| Epipodophyllotoxins | cg07573872 | chr19 | 1,126,342 | 1,126,344 | *SBNO2* | -0.094 | (0.011) | 1.1E-15 | -0.026 | 3.1E-07 |
| Epipodophyllotoxins | cg03719252 | chr6 | 140,208,293 | 140,208,295 | *NA* | -0.149 | (0.017) | 2.0E-15 | -0.046 | 2.1E-08 |
| Epipodophyllotoxins | cg24765521 | chr9 | 90,296,803 | 90,296,805 | *NA* | -0.146 | (0.017) | 2.8E-15 | -0.035 | 1.3E-05 |
| Epipodophyllotoxins | cg27024924 | chr6 | 21,186,285 | 21,186,287 | *CDKAL1* | -0.145 | (0.017) | 3.2E-15 | -0.042 | 1.4E-07 |
| Epipodophyllotoxins | cg15817542 | chr16 | 31,331,734 | 31,331,736 | *ITGAM* | 0.185 | (0.022) | 4.8E-15 | 0.051 | 8.4E-07 |
| Epipodophyllotoxins | cg17407859 | chr3 | 165,259,995 | 165,259,997 | *LINC01322* | -0.160 | (0.019) | 7.0E-15 | -0.046 | 3.3E-07 |
| Epipodophyllotoxins | cg01150955 | chr4 | 83,686,183 | 83,686,185 | *RP11-767N15.1* | -0.146 | (0.017) | 7.7E-15 | -0.038 | 3.7E-06 |
| Epipodophyllotoxins | cg26038465 | chr22 | 38,214,506 | 38,214,508 | *MAFF* | 0.098 | (0.012) | 9.3E-15 | 0.034 | 7.3E-10 |
| Epipodophyllotoxins | cg22943590 | chr2 | 66,421,664 | 66,421,666 | *NA* | -0.181 | (0.022) | 1.2E-14 | -0.065 | 2.0E-10 |
| Epipodophyllotoxins | cg02129236 | chr14 | 38,290,732 | 38,290,734 | *RP11-96D24.1* | -0.137 | (0.016) | 1.2E-14 | -0.060 | 1.1E-14 |
| Epipodophyllotoxins | cg01004017 | chr2 | 120,899,770 | 120,899,772 | *GLI2* | 0.121 | (0.014) | 1.3E-14 | 0.034 | 8.0E-07 |
| Epipodophyllotoxins | cg12009405 | chr6 | 148,330,194 | 148,330,196 | *SASH1* | -0.157 | (0.019) | 1.5E-14 | -0.036 | 4.6E-05 |
| Epipodophyllotoxins | cg18763712 | chr2 | 74,602,770 | 74,602,772 | *M1AP* | -0.104 | (0.013) | 1.6E-14 | -0.032 | 5.5E-08 |
| Epipodophyllotoxins | cg22729726 | chr1 | 3,207,289 | 3,207,291 | *PRDM16;RP11-193J6.1* | 0.075 | (0.009) | 1.6E-14 | 0.020 | 4.7E-06 |
| Epipodophyllotoxins | cg07118262 | chr17 | 38,510,225 | 38,510,227 | *ARHGAP23* | 0.128 | (0.015) | 1.7E-14 | 0.041 | 1.9E-08 |
| Epipodophyllotoxins | cg19291696 | chr13 | 27,195,299 | 27,195,301 | *RP11-428O18.6* | -0.095 | (0.011) | 1.9E-14 | -0.035 | 1.0E-10 |
| Epipodophyllotoxins | cg21667520 | chr10 | 78,862,391 | 78,862,393 | *NA* | -0.056 | (0.007) | 2.1E-14 | -0.016 | 3.3E-07 |
| Epipodophyllotoxins | cg03479114 | chr4 | 165,359,572 | 165,359,574 | *NA* | -0.111 | (0.013) | 2.2E-14 | -0.026 | 4.3E-05 |
| Epipodophyllotoxins | cg05424022 | chr17 | 38,509,589 | 38,509,591 | *ARHGAP23* | 0.182 | (0.022) | 2.2E-14 | 0.057 | 3.9E-08 |
| Epipodophyllotoxins | cg12943885 | chr5 | 39,650,826 | 39,650,828 | *NA* | -0.091 | (0.011) | 2.2E-14 | -0.022 | 2.9E-05 |
| Epipodophyllotoxins | cg16300419 | chr17 | 38,509,917 | 38,509,919 | *ARHGAP23* | 0.169 | (0.021) | 2.9E-14 | 0.055 | 1.4E-08 |
| Epipodophyllotoxins | cg12307404 | chr3 | 186,552,703 | 186,552,705 | *TBCCD1* | -0.070 | (0.008) | 3.0E-14 | -0.018 | 8.6E-06 |
| Epipodophyllotoxins | cg01564268 | chr17 | 38,510,716 | 38,510,718 | *ARHGAP23* | 0.183 | (0.022) | 6.0E-14 | 0.057 | 6.5E-08 |
| Epipodophyllotoxins | cg11328695 | chr9 | 122,229,068 | 122,229,070 | *LHX6* | 0.222 | (0.027) | 6.4E-14 | 0.068 | 1.3E-07 |
| Epipodophyllotoxins | cg20996351 | chr1 | 27,360,740 | 27,360,742 | *MAP3K6* | 0.313 | (0.039) | 7.0E-14 | 0.110 | 1.7E-09 |
| Epipodophyllotoxins | cg09277673 | chr12 | 60,395,015 | 60,395,017 | *NA* | -0.148 | (0.018) | 7.6E-14 | -0.036 | 2.6E-05 |
| Epipodophyllotoxins | cg11637712 | chr17 | 38,509,607 | 38,509,609 | *ARHGAP23* | 0.201 | (0.025) | 1.1E-13 | 0.064 | 7.0E-08 |
| Epipodophyllotoxins | cg13903421 | chr2 | 218,873,991 | 218,873,993 | *WNT6* | 0.186 | (0.023) | 1.3E-13 | 0.047 | 1.7E-05 |
| Epipodophyllotoxins | cg09720404 | chr9 | 79,801,729 | 79,801,731 | *NA* | -0.081 | (0.010) | 1.5E-13 | -0.021 | 8.1E-06 |
| Epipodophyllotoxins | cg10941826 | chr9 | 124,741,841 | 124,741,843 | *NR6A1* | -0.067 | (0.008) | 1.6E-13 | -0.021 | 1.3E-07 |
| Epipodophyllotoxins | cg01693697 | chr15 | 73,920,554 | 73,920,556 | *LOXL1-AS1* | -0.116 | (0.015) | 1.6E-13 | -0.036 | 1.8E-07 |
| Epipodophyllotoxins | cg12438803 | chr5 | 37,071,735 | 37,071,737 | *NA* | -0.089 | (0.011) | 2.1E-13 | -0.038 | 2.8E-13 |
| Epipodophyllotoxins | cg23207077 | chr1 | 970,607 | 970,609 | *PLEKHN1* | 0.072 | (0.009) | 2.1E-13 | 0.024 | 4.4E-08 |
| Epipodophyllotoxins | cg08257257 | chr11 | 68,750,335 | 68,750,337 | *TESMIN* | 0.050 | (0.006) | 2.2E-13 | 0.013 | 7.2E-06 |
| Epipodophyllotoxins | cg05715492 | chr7 | 99,393,514 | 99,393,516 | *ARPC1B;PDAP1* | 0.072 | (0.009) | 3.0E-13 | 0.021 | 8.6E-07 |
| Epipodophyllotoxins | cg11686251 | chr9 | 131,598,498 | 131,598,500 | *RAPGEF1* | 0.094 | (0.012) | 3.1E-13 | 0.030 | 1.2E-07 |
| Epipodophyllotoxins | cg21150701 | chr2 | 120,899,241 | 120,899,243 | *GLI2* | 0.077 | (0.010) | 3.3E-13 | 0.020 | 2.0E-05 |
| Epipodophyllotoxins | cg17996394 | chr19 | 4,075,485 | 4,075,487 | *NA* | -0.060 | (0.008) | 3.5E-13 | -0.020 | 3.6E-08 |
| Epipodophyllotoxins | cg04179952 | chr1 | 201,650,166 | 201,650,168 | *NAV1* | 0.295 | (0.037) | 4.1E-13 | 0.081 | 5.0E-06 |
| Epipodophyllotoxins | cg04084749 | chr2 | 186,575,639 | 186,575,641 | *NA* | -0.133 | (0.017) | 4.4E-13 | -0.036 | 8.2E-06 |
| Epipodophyllotoxins | cg26811385 | chr4 | 149,910,850 | 149,910,852 | *NA* | -0.111 | (0.014) | 4.4E-13 | -0.029 | 2.0E-05 |
| Epipodophyllotoxins | cg18509466 | chr1 | 3,071,396 | 3,071,398 | *PRDM16* | 0.073 | (0.009) | 5.2E-13 | 0.027 | 7.2E-10 |
| Epipodophyllotoxins | cg01663665 | chr20 | 24,816,582 | 24,816,584 | *NA* | -0.057 | (0.007) | 5.7E-13 | -0.014 | 6.9E-05 |
| Epipodophyllotoxins | cg10353174 | chr8 | 105,788,523 | 105,788,525 | *ZFPM2;ZFPM2-AS1* | -0.122 | (0.016) | 6.4E-13 | -0.050 | 1.9E-11 |
| Epipodophyllotoxins | cg09992085 | chr19 | 41,456,428 | 41,456,430 | *PCAT19* | -0.093 | (0.012) | 7.0E-13 | -0.034 | 1.6E-09 |
| Epipodophyllotoxins | cg03984209 | chr3 | 55,885,989 | 55,885,991 | *ERC2* | -0.097 | (0.012) | 7.1E-13 | -0.021 | 3.2E-04 |
| Epipodophyllotoxins | cg11054905 | chr18 | 46,718,997 | 46,718,999 | *ST8SIA5* | -0.085 | (0.011) | 7.2E-13 | -0.026 | 5.9E-07 |
| Epipodophyllotoxins | cg15063366 | chr2 | 227,818,315 | 227,818,317 | *NA* | -0.090 | (0.012) | 7.7E-13 | -0.030 | 3.7E-08 |
| Epipodophyllotoxins | cg11988169 | chr5 | 53,543,411 | 53,543,413 | *NA* | -0.235 | (0.030) | 7.8E-13 | -0.065 | 4.6E-06 |
| Epipodophyllotoxins | cg02610360 | chr11 | 120,326,811 | 120,326,813 | *TMEM136* | 0.124 | (0.016) | 9.1E-13 | 0.038 | 3.5E-07 |
| Epipodophyllotoxins | cg13959831 | chr9 | 36,938,343 | 36,938,345 | *PAX5* | -0.097 | (0.013) | 1.1E-12 | -0.026 | 1.0E-05 |
| Epipodophyllotoxins | cg26547821 | chr1 | 71,366,080 | 71,366,082 | *ZRANB2-AS2* | -0.156 | (0.020) | 1.1E-12 | -0.055 | 1.2E-08 |
| Epipodophyllotoxins | cg16887422 | chr2 | 239,113,410 | 239,113,412 | *HDAC4* | 0.092 | (0.012) | 1.1E-12 | 0.023 | 3.8E-05 |
| Epipodophyllotoxins | cg17125362 | chr18 | 8,526,137 | 8,526,139 | *NA* | -0.071 | (0.009) | 1.2E-12 | -0.017 | 1.0E-04 |
| Epipodophyllotoxins | cg05331731 | chr18 | 11,147,146 | 11,147,148 | *PIEZO2* | 0.151 | (0.020) | 1.3E-12 | 0.044 | 2.7E-06 |
| Epipodophyllotoxins | cg05712639 | chr14 | 52,352,667 | 52,352,669 | *NA* | -0.159 | (0.021) | 1.4E-12 | -0.050 | 4.4E-07 |
| Epipodophyllotoxins | cg05468212 | chr9 | 134,796,289 | 134,796,291 | *COL5A1* | 0.050 | (0.007) | 1.5E-12 | 0.012 | 9.3E-05 |
| Epipodophyllotoxins | cg11846580 | chr16 | 88,524,000 | 88,524,002 | *RP11-21B21.4;ZFPM1* | 0.086 | (0.011) | 1.5E-12 | 0.024 | 8.1E-06 |
| Epipodophyllotoxins | cg08057120 | chr11 | 97,364,170 | 97,364,172 | *NA* | -0.176 | (0.023) | 2.1E-12 | -0.037 | 6.8E-04 |
| Epipodophyllotoxins | cg16517702 | chr1 | 201,648,724 | 201,648,726 | *NAV1* | 0.202 | (0.027) | 2.2E-12 | 0.057 | 4.9E-06 |
| Epipodophyllotoxins | cg06744585 | chr19 | 13,024,993 | 13,024,995 | *NFIX* | 0.217 | (0.029) | 2.8E-12 | 0.052 | 1.4E-04 |
| Epipodophyllotoxins | cg17302948 | chr11 | 36,149,980 | 36,149,982 | *LDLRAD3* | -0.073 | (0.010) | 2.8E-12 | -0.019 | 3.0E-05 |
| Epipodophyllotoxins | cg14920846 | chr1 | 201,649,080 | 201,649,082 | *NAV1* | 0.180 | (0.024) | 2.9E-12 | 0.046 | 3.5E-05 |
| Epipodophyllotoxins | cg19743666 | chr2 | 30,148,617 | 30,148,619 | *YPEL5* | -0.122 | (0.016) | 3.2E-12 | -0.038 | 7.5E-07 |
| Epipodophyllotoxins | cg17445936 | chr1 | 3,173,780 | 3,173,782 | *PRDM16* | 0.152 | (0.020) | 3.2E-12 | 0.043 | 6.0E-06 |
| Epipodophyllotoxins | cg24698979 | chr17 | 38,509,856 | 38,509,858 | *ARHGAP23* | 0.214 | (0.028) | 3.5E-12 | 0.069 | 2.4E-07 |
| Epipodophyllotoxins | cg17389813 | chr11 | 122,189,556 | 122,189,558 | *MIR100HG* | -0.146 | (0.019) | 3.6E-12 | -0.044 | 2.0E-06 |
| Epipodophyllotoxins | cg12049992 | chr18 | 11,147,785 | 11,147,787 | *PIEZO2* | 0.135 | (0.018) | 3.7E-12 | 0.039 | 4.2E-06 |
| Epipodophyllotoxins | cg17763553 | chr8 | 19,095,972 | 19,095,974 | *RP11-1080G15.1;RP11-1080G15.2* | -0.126 | (0.017) | 3.9E-12 | -0.033 | 3.4E-05 |
| Epipodophyllotoxins | cg19634902 | chr2 | 201,652,689 | 201,652,691 | *MPP4* | -0.078 | (0.010) | 3.9E-12 | -0.026 | 1.9E-07 |
| Epipodophyllotoxins | cg10587886 | chr3 | 8,320,146 | 8,320,148 | *LMCD1-AS1* | -0.132 | (0.018) | 4.3E-12 | -0.036 | 1.4E-05 |
| Epipodophyllotoxins | cg02450387 | chr10 | 6,722,353 | 6,722,355 | *NA* | -0.082 | (0.011) | 4.8E-12 | -0.030 | 5.5E-09 |
| Epipodophyllotoxins | cg20676788 | chr13 | 48,805,654 | 48,805,656 | *NA* | -0.101 | (0.014) | 5.0E-12 | -0.030 | 2.4E-06 |
| Epipodophyllotoxins | cg21385305 | chr2 | 23,490,082 | 23,490,084 | *KLHL29* | -0.093 | (0.012) | 5.2E-12 | -0.028 | 1.5E-06 |
| Epipodophyllotoxins | cg23165574 | chr9 | 16,510,110 | 16,510,112 | *BNC2* | -0.067 | (0.009) | 5.4E-12 | -0.020 | 1.7E-06 |
| Epipodophyllotoxins | cg09608551 | chr12 | 49,920,554 | 49,920,556 | *RP11-70F11.8* | -0.071 | (0.009) | 5.5E-12 | -0.022 | 6.3E-07 |
| Epipodophyllotoxins | cg18480977 | chr19 | 13,072,035 | 13,072,037 | *AC007787.2;NFIX* | 0.065 | (0.009) | 5.6E-12 | 0.015 | 3.7E-04 |
| Epipodophyllotoxins | cg21791662 | chr2 | 120,867,632 | 120,867,634 | *GLI2;RP11-297J22.1* | 0.154 | (0.021) | 5.8E-12 | 0.032 | 1.0E-03 |
| Epipodophyllotoxins | cg00062245 | chr17 | 44,384,891 | 44,384,893 | *ITGA2B* | 0.082 | (0.011) | 6.0E-12 | 0.019 | 3.3E-04 |
| Epipodophyllotoxins | cg07172149 | chr7 | 136,883,124 | 136,883,126 | *AC009264.1;CHRM2* | -0.094 | (0.013) | 6.2E-12 | -0.033 | 2.9E-08 |
| Epipodophyllotoxins | cg01356139 | chr7 | 40,885,562 | 40,885,564 | *NA* | -0.049 | (0.007) | 8.0E-12 | -0.014 | 7.7E-06 |
| Epipodophyllotoxins | cg11031221 | chr17 | 72,421,375 | 72,421,377 | *LINC00511* | -0.069 | (0.009) | 9.5E-12 | -0.017 | 8.8E-05 |
| Epipodophyllotoxins | cg25896883 | chr2 | 189,407,943 | 189,407,945 | *NA* | -0.106 | (0.014) | 1.1E-11 | -0.036 | 1.0E-07 |
| Epipodophyllotoxins | cg09296604 | chr11 | 68,750,330 | 68,750,332 | *TESMIN* | 0.103 | (0.014) | 1.1E-11 | 0.032 | 1.7E-06 |
| Epipodophyllotoxins | cg03505273 | chr11 | 23,805,076 | 23,805,078 | *RP11-945A11.2* | -0.128 | (0.017) | 1.2E-11 | -0.039 | 2.7E-06 |
| Epipodophyllotoxins | cg02329226 | chr11 | 66,209,316 | 66,209,318 | *PACS1* | 0.052 | (0.007) | 1.6E-11 | 0.016 | 3.9E-06 |
| Epipodophyllotoxins | cg13286857 | chr5 | 66,822,120 | 66,822,122 | *MAST4* | -0.066 | (0.009) | 1.7E-11 | -0.017 | 6.4E-05 |
| Epipodophyllotoxins | cg10724969 | chr3 | 33,664,475 | 33,664,477 | *CLASP2* | -0.096 | (0.013) | 1.9E-11 | -0.030 | 1.6E-06 |
| Epipodophyllotoxins | cg24879954 | chr2 | 98,775,640 | 98,775,642 | *NA* | -0.099 | (0.014) | 2.0E-11 | -0.034 | 1.0E-07 |
| Epipodophyllotoxins | cg11447971 | chr7 | 116,577,259 | 116,577,261 | *LINC01510* | -0.071 | (0.010) | 2.6E-11 | -0.025 | 5.1E-08 |
| Epipodophyllotoxins | cg27093918 | chr16 | 69,530,721 | 69,530,723 | *NA* | -0.113 | (0.016) | 2.9E-11 | -0.034 | 3.9E-06 |
| Epipodophyllotoxins | cg01001422 | chr19 | 13,024,816 | 13,024,818 | *NFIX* | 0.093 | (0.013) | 3.1E-11 | 0.022 | 3.0E-04 |
| Epipodophyllotoxins | cg03216822 | chr11 | 23,805,197 | 23,805,199 | *RP11-945A11.2* | -0.085 | (0.012) | 3.5E-11 | -0.019 | 8.3E-04 |
| Epipodophyllotoxins | cg15825321 | chr17 | 38,510,405 | 38,510,407 | *ARHGAP23* | 0.101 | (0.014) | 3.6E-11 | 0.032 | 1.7E-06 |
| Epipodophyllotoxins | cg21774980 | chr20 | 35,502,719 | 35,502,721 | *CEP250* | 0.046 | (0.006) | 4.5E-11 | 0.015 | 1.6E-06 |
| Epipodophyllotoxins | cg08618219 | chr15 | 47,412,312 | 47,412,314 | *SEMA6D* | -0.111 | (0.016) | 4.6E-11 | -0.044 | 1.4E-09 |
| Epipodophyllotoxins | cg22815707 | chr18 | 9,138,607 | 9,138,609 | *ANKRD12;RP11-21J18.1* | -0.121 | (0.017) | 5.2E-11 | -0.039 | 1.4E-06 |
| Epipodophyllotoxins | cg12641545 | chr18 | 7,601,197 | 7,601,199 | *PTPRM* | -0.133 | (0.019) | 5.6E-11 | -0.030 | 6.8E-04 |
| Epipodophyllotoxins | cg08650597 | chr15 | 100,902,500 | 100,902,502 | *ALDH1A3;RP11-66B24.4* | -0.074 | (0.010) | 5.8E-11 | -0.026 | 1.3E-07 |
| Epipodophyllotoxins | cg05449815 | chr17 | 3,640,570 | 3,640,572 | *CTNS* | -0.075 | (0.011) | 6.9E-11 | -0.022 | 1.2E-05 |
| Epipodophyllotoxins | cg04287574 | chr1 | 201,650,493 | 201,650,495 | *NAV1* | 0.168 | (0.024) | 7.4E-11 | 0.044 | 8.1E-05 |
| Epipodophyllotoxins | cg25618424 | chr1 | 3,072,742 | 3,072,744 | *PRDM16* | 0.089 | (0.013) | 8.2E-11 | 0.036 | 1.4E-09 |
| Epipodophyllotoxins | cg18468796 | chr15 | 74,319,835 | 74,319,837 | *CCDC33;RP11-60L3.6* | 0.045 | (0.006) | 8.2E-11 | 0.015 | 2.9E-07 |
| Epipodophyllotoxins | cg11410718 | chr7 | 27,130,792 | 27,130,794 | *HOXA-AS2;HOXA-AS3;HOXA3;HOXA4;RP1-170O19.22* | 0.187 | (0.027) | 8.4E-11 | 0.048 | 1.4E-04 |
| Epipodophyllotoxins | cg16265717 | chr1 | 65,527,465 | 65,527,467 | *LEPR* | -0.144 | (0.020) | 8.4E-11 | -0.061 | 1.8E-10 |
| Epipodophyllotoxins | cg27115863 | chr22 | 37,525,632 | 37,525,634 | *NA* | -0.053 | (0.008) | 8.5E-11 | -0.017 | 2.4E-06 |
| Epipodophyllotoxins | cg15975895 | chr3 | 178,950,818 | 178,950,820 | *NA* | -0.099 | (0.014) | 8.6E-11 | -0.026 | 1.0E-04 |
| Epipodophyllotoxins | cg14686645 | chr17 | 44,375,057 | 44,375,059 | *ITGA2B* | 0.127 | (0.018) | 8.7E-11 | 0.032 | 1.4E-04 |
| Epipodophyllotoxins | cg07317062 | chr7 | 27,130,768 | 27,130,770 | *HOXA-AS2;HOXA-AS3;HOXA3;HOXA4;RP1-170O19.22* | 0.204 | (0.029) | 9.0E-11 | 0.052 | 1.5E-04 |
| Epipodophyllotoxins | cg01354879 | chr6 | 83,430,330 | 83,430,332 | *ME1* | -0.123 | (0.018) | 9.1E-11 | -0.032 | 9.6E-05 |
| Epipodophyllotoxins | cg03183160 | chr15 | 58,312,163 | 58,312,165 | *ALDH1A2* | -0.070 | (0.010) | 9.2E-11 | -0.021 | 1.0E-05 |
| Epipodophyllotoxins | cg07598481 | chr13 | 72,194,680 | 72,194,682 | *NA* | -0.117 | (0.017) | 9.3E-11 | -0.039 | 6.2E-07 |
| Epipodophyllotoxins | cg05513069 | chr18 | 46,028,342 | 46,028,344 | *PSTPIP2;RP11-8H2.1* | 0.238 | (0.034) | 9.8E-11 | 0.083 | 2.0E-07 |
| Epipodophyllotoxins | cg02699612 | chr10 | 113,753,520 | 113,753,522 | *PLEKHS1* | -0.133 | (0.019) | 9.9E-11 | -0.044 | 7.6E-07 |
| Epipodophyllotoxins | cg19604806 | chr10 | 113,463,930 | 113,463,932 | *NA* | -0.075 | (0.011) | 1.0E-10 | -0.026 | 2.9E-07 |
| Epipodophyllotoxins | cg06148273 | chr5 | 83,306,780 | 83,306,782 | *XRCC4* | -0.087 | (0.013) | 1.1E-10 | -0.029 | 6.3E-07 |
| Epipodophyllotoxins | cg12022558 | chr2 | 205,245,819 | 205,245,821 | *PARD3B* | -0.119 | (0.017) | 1.1E-10 | -0.027 | 6.5E-04 |
| Epipodophyllotoxins | cg04317399 | chr7 | 27,130,693 | 27,130,695 | *HOXA-AS2;HOXA-AS3;HOXA3;HOXA4;RP1-170O19.22* | 0.157 | (0.023) | 1.2E-10 | 0.039 | 2.2E-04 |
| Epipodophyllotoxins | cg12588978 | chr21 | 30,338,894 | 30,338,896 | *KRTAP27-1* | -0.063 | (0.009) | 1.3E-10 | -0.017 | 6.5E-05 |
| Epipodophyllotoxins | cg20457580 | chr20 | 32,457,522 | 32,457,524 | *NOL4L* | 0.050 | (0.007) | 1.5E-10 | 0.016 | 1.0E-06 |
| Epipodophyllotoxins | cg14307056 | chr1 | 109,167,309 | 109,167,311 | *KIAA1324* | -0.076 | (0.011) | 1.5E-10 | -0.023 | 6.8E-06 |
| Epipodophyllotoxins | cg01600165 | chr12 | 62,269,167 | 62,269,169 | *FAM19A2;USP15* | -0.098 | (0.014) | 1.5E-10 | -0.030 | 7.5E-06 |
| Epipodophyllotoxins | cg09840667 | chr11 | 68,750,315 | 68,750,317 | *TESMIN* | 0.067 | (0.010) | 1.5E-10 | 0.017 | 2.8E-04 |
| Epipodophyllotoxins | cg01376829 | chr2 | 102,187,944 | 102,187,946 | *IL1RL2* | 0.100 | (0.014) | 1.6E-10 | 0.028 | 3.3E-05 |
| Epipodophyllotoxins | cg13730618 | chr21 | 34,717,106 | 34,717,108 | *CLIC6* | -0.071 | (0.010) | 1.8E-10 | -0.024 | 9.7E-07 |
| Epipodophyllotoxins | cg19151852 | chr17 | 81,980,734 | 81,980,736 | *ASPSCR1* | 0.095 | (0.014) | 1.8E-10 | 0.029 | 8.0E-06 |
| Epipodophyllotoxins | cg07269647 | chr10 | 127,889,734 | 127,889,736 | *CLRN3* | -0.100 | (0.014) | 1.8E-10 | -0.025 | 2.1E-04 |
| Epipodophyllotoxins | cg15852318 | chr6 | 64,736,784 | 64,736,786 | *EYS* | -0.121 | (0.018) | 2.1E-10 | -0.033 | 8.1E-05 |
| Epipodophyllotoxins | cg16055613 | chr6 | 163,766,368 | 163,766,370 | *NA* | -0.083 | (0.012) | 2.3E-10 | -0.030 | 1.0E-07 |
| Epipodophyllotoxins | cg24396605 | chr1 | 970,741 | 970,743 | *PLEKHN1* | 0.090 | (0.013) | 2.3E-10 | 0.026 | 2.6E-05 |
| Epipodophyllotoxins | cg18317842 | chr8 | 11,063,991 | 11,063,993 | *AF131215.6;XKR6* | -0.064 | (0.009) | 2.4E-10 | -0.019 | 1.4E-05 |
| Epipodophyllotoxins | cg26922444 | chr6 | 24,910,975 | 24,910,977 | *RIPOR2* | 0.097 | (0.014) | 2.4E-10 | 0.034 | 3.1E-07 |
| Epipodophyllotoxins | cg17457637 | chr7 | 27,131,097 | 27,131,099 | *HOXA-AS2;HOXA-AS3;HOXA3;HOXA4;RP1-170O19.22* | 0.164 | (0.024) | 2.6E-10 | 0.041 | 3.0E-04 |
| Epipodophyllotoxins | cg10570484 | chr1 | 158,038,015 | 158,038,017 | *KIRREL1* | -0.051 | (0.008) | 2.7E-10 | -0.012 | 8.4E-04 |
| Epipodophyllotoxins | cg12573976 | chr7 | 101,551,445 | 101,551,447 | *COL26A1* | -0.047 | (0.007) | 2.7E-10 | -0.015 | 1.9E-06 |
| Epipodophyllotoxins | cg17958964 | chr9 | 101,440,755 | 101,440,757 | *NA* | -0.072 | (0.011) | 2.7E-10 | -0.017 | 5.4E-04 |
| Epipodophyllotoxins | cg26376241 | chr2 | 65,366,886 | 65,366,888 | *SPRED2* | -0.118 | (0.017) | 2.8E-10 | -0.034 | 3.2E-05 |
| Epipodophyllotoxins | cg18158149 | chr1 | 162,168,424 | 162,168,426 | *NOS1AP* | -0.061 | (0.009) | 2.9E-10 | -0.018 | 3.1E-05 |
| Epipodophyllotoxins | cg11211942 | chr15 | 56,815,423 | 56,815,425 | *ZNF280D* | -0.078 | (0.011) | 2.9E-10 | -0.028 | 2.3E-07 |
| Epipodophyllotoxins | cg24169822 | chr7 | 27,131,374 | 27,131,376 | *HOXA-AS2;HOXA-AS3;HOXA3;HOXA4;RP1-170O19.22* | 0.179 | (0.026) | 2.9E-10 | 0.043 | 5.3E-04 |
| Epipodophyllotoxins | cg02265177 | chr2 | 146,124,946 | 146,124,948 | *NA* | 0.083 | (0.012) | 3.1E-10 | 0.020 | 4.1E-04 |
| Epipodophyllotoxins | cg24629297 | chr15 | 34,701,648 | 34,701,650 | *NA* | 0.042 | (0.006) | 3.2E-10 | 0.013 | 1.5E-05 |
| Epipodophyllotoxins | cg21734651 | chr10 | 74,145,284 | 74,145,286 | *AP3M1* | -0.117 | (0.017) | 3.9E-10 | -0.041 | 6.5E-07 |
| Epipodophyllotoxins | cg14731462 | chr10 | 127,991,164 | 127,991,166 | *PTPRE* | -0.089 | (0.013) | 4.0E-10 | -0.032 | 2.4E-07 |
| Epipodophyllotoxins | cg09978353 | chr17 | 74,763,142 | 74,763,144 | *SLC9A3R1* | 0.068 | (0.010) | 4.4E-10 | 0.023 | 1.9E-06 |
| Epipodophyllotoxins | cg05107036 | chr8 | 140,653,660 | 140,653,662 | *NA* | -0.078 | (0.012) | 4.7E-10 | -0.026 | 1.1E-06 |
| Epipodophyllotoxins | cg16540258 | chr12 | 27,822,241 | 27,822,243 | *NA* | -0.075 | (0.011) | 5.2E-10 | -0.020 | 1.3E-04 |
| Epipodophyllotoxins | cg24496847 | chr20 | 32,457,310 | 32,457,312 | *NOL4L* | 0.058 | (0.009) | 5.3E-10 | 0.018 | 7.4E-06 |
| Epipodophyllotoxins | cg02933413 | chr3 | 155,702,354 | 155,702,356 | *PLCH1* | -0.104 | (0.016) | 5.4E-10 | -0.032 | 8.9E-06 |
| Epipodophyllotoxins | cg14915244 | chr1 | 2,148,773 | 2,148,775 | *PRKCZ* | -0.123 | (0.018) | 5.8E-10 | -0.029 | 9.7E-04 |
| Epipodophyllotoxins | cg01641754 | chr5 | 99,020,278 | 99,020,280 | *NA* | -0.081 | (0.012) | 5.9E-10 | -0.019 | 7.9E-04 |
| Epipodophyllotoxins | cg13713922 | chr2 | 120,868,001 | 120,868,003 | *GLI2;RP11-297J22.1* | 0.114 | (0.017) | 6.5E-10 | 0.022 | 7.0E-03 |
| Epipodophyllotoxins | cg06225648 | chr9 | 107,213,793 | 107,213,795 | *NA* | -0.068 | (0.010) | 6.6E-10 | -0.021 | 9.1E-06 |
| Epipodophyllotoxins | cg13442016 | chr10 | 102,436,581 | 102,436,583 | *MIR146B* | -0.085 | (0.013) | 6.6E-10 | -0.026 | 1.6E-05 |
| Epipodophyllotoxins | cg25440434 | chr1 | 3,350,932 | 3,350,934 | *PRDM16* | 0.078 | (0.012) | 6.9E-10 | 0.020 | 3.3E-04 |
| Epipodophyllotoxins | cg15857661 | chr10 | 102,436,485 | 102,436,487 | *MIR146B* | -0.083 | (0.013) | 7.1E-10 | -0.027 | 3.8E-06 |
| Epipodophyllotoxins | cg00770773 | chr3 | 192,853,856 | 192,853,858 | *MB21D2* | 0.106 | (0.016) | 7.3E-10 | 0.033 | 7.9E-06 |
| Epipodophyllotoxins | cg02205744 | chr15 | 62,957,201 | 62,957,203 | *NA* | -0.058 | (0.009) | 7.6E-10 | -0.022 | 1.2E-07 |
| Epipodophyllotoxins | cg04353171 | chr6 | 30,730,951 | 30,730,953 | *FLOT1* | 0.091 | (0.014) | 7.8E-10 | 0.034 | 1.4E-07 |
| Epipodophyllotoxins | cg09410512 | chr2 | 223,699,682 | 223,699,684 | *NA* | -0.122 | (0.018) | 8.2E-10 | -0.035 | 4.7E-05 |
| Epipodophyllotoxins | cg14359292 | chr7 | 27,131,272 | 27,131,274 | *HOXA-AS2;HOXA-AS3;HOXA3;HOXA4;RP1-170O19.22* | 0.146 | (0.022) | 8.4E-10 | 0.039 | 2.0E-04 |
| Epipodophyllotoxins | cg06738602 | chr14 | 52,313,915 | 52,313,917 | *PTGER2* | 0.102 | (0.015) | 8.6E-10 | 0.031 | 2.1E-05 |
| Epipodophyllotoxins | cg25151498 | chr3 | 186,098,202 | 186,098,204 | *ETV5* | 0.101 | (0.015) | 8.8E-10 | 0.023 | 1.7E-03 |
| Epipodophyllotoxins | cg05453127 | chr14 | 49,973,098 | 49,973,100 | *NA* | -0.093 | (0.014) | 8.8E-10 | -0.033 | 4.9E-07 |
| Epipodophyllotoxins | cg05238750 | chr7 | 156,903,588 | 156,903,590 | *NA* | -0.085 | (0.013) | 9.0E-10 | -0.025 | 4.7E-05 |
| Epipodophyllotoxins | cg17723693 | chr14 | 84,442,754 | 84,442,756 | *NA* | -0.101 | (0.015) | 9.2E-10 | -0.029 | 5.3E-05 |
| Epipodophyllotoxins | cg03864568 | chr9 | 76,400,794 | 76,400,796 | *NA* | -0.096 | (0.014) | 9.4E-10 | -0.023 | 6.5E-04 |
| Epipodophyllotoxins | cg21694644 | chr15 | 88,491,346 | 88,491,348 | *RP11-97O12.7* | -0.044 | (0.007) | 1.0E-09 | -0.012 | 7.3E-05 |
| Epipodophyllotoxins | cg11155924 | chr11 | 70,603,152 | 70,603,154 | *RP11-826F13.1;SHANK2* | 0.101 | (0.015) | 1.1E-09 | 0.030 | 4.1E-05 |
| Epipodophyllotoxins | cg09294084 | chr13 | 112,992,417 | 112,992,419 | *MCF2L* | 0.083 | (0.013) | 1.1E-09 | 0.024 | 5.2E-05 |
| Epipodophyllotoxins | cg10856826 | chr5 | 42,882,054 | 42,882,056 | *SELENOP* | -0.070 | (0.011) | 1.1E-09 | -0.024 | 1.1E-06 |
| Epipodophyllotoxins | cg03465652 | chr2 | 120,868,159 | 120,868,161 | *GLI2;RP11-297J22.1* | 0.092 | (0.014) | 1.1E-09 | 0.018 | 6.3E-03 |
| Epipodophyllotoxins | cg03080336 | chr4 | 185,112,223 | 185,112,225 | *NA* | -0.067 | (0.010) | 1.1E-09 | -0.021 | 9.7E-06 |
| Epipodophyllotoxins | cg24277817 | chr19 | 13,024,847 | 13,024,849 | *NFIX* | 0.100 | (0.015) | 1.2E-09 | 0.018 | 1.1E-02 |
| Epipodophyllotoxins | cg04965297 | chr19 | 13,024,514 | 13,024,516 | *NFIX* | 0.111 | (0.017) | 1.2E-09 | 0.026 | 1.1E-03 |
| Epipodophyllotoxins | cg08705753 | chr12 | 27,332,210 | 27,332,212 | *ARNTL2* | -0.049 | (0.008) | 1.3E-09 | -0.015 | 2.5E-05 |
| Epipodophyllotoxins | cg11291879 | chr20 | 22,285,800 | 22,285,802 | *NA* | -0.042 | (0.006) | 1.3E-09 | -0.013 | 1.7E-05 |
| Epipodophyllotoxins | cg15105359 | chr7 | 47,537,358 | 47,537,360 | *TNS3* | 0.175 | (0.027) | 1.3E-09 | 0.045 | 4.1E-04 |
| Epipodophyllotoxins | cg22997113 | chr7 | 27,130,621 | 27,130,623 | *HOXA-AS2;HOXA-AS3;HOXA3;HOXA4;RP1-170O19.22* | 0.180 | (0.027) | 1.4E-09 | 0.046 | 3.5E-04 |
| Epipodophyllotoxins | cg07690222 | chr12 | 83,015,895 | 83,015,897 | *TMTC2* | -0.151 | (0.023) | 1.4E-09 | -0.053 | 1.1E-06 |
| Epipodophyllotoxins | cg02256616 | chr16 | 1,579,407 | 1,579,409 | *IFT140;LA16c-313F4.1;LA16c-395F10.2;LA16c-425C2.1* | 0.062 | (0.009) | 1.6E-09 | 0.020 | 4.0E-06 |
| Epipodophyllotoxins | cg04688366 | chr7 | 11,458,999 | 11,459,001 | *AC004538.3;THSD7A* | -0.075 | (0.011) | 1.6E-09 | -0.023 | 1.5E-05 |
| Epipodophyllotoxins | cg06795233 | chr2 | 218,872,669 | 218,872,671 | *WNT6* | 0.108 | (0.017) | 1.7E-09 | 0.024 | 1.9E-03 |
| Epipodophyllotoxins | cg07518532 | chr11 | 1,968,276 | 1,968,278 | *MRPL23* | 0.049 | (0.008) | 1.7E-09 | 0.011 | 1.4E-03 |
| Epipodophyllotoxins | cg08903740 | chr1 | 162,317,927 | 162,317,929 | *NOS1AP;RP11-565P22.2* | 0.065 | (0.010) | 1.8E-09 | 0.022 | 3.7E-06 |
| Epipodophyllotoxins | cg22676671 | chr10 | 73,785,041 | 73,785,043 | *ZSWIM8* | -0.082 | (0.013) | 1.8E-09 | -0.020 | 6.2E-04 |
| Epipodophyllotoxins | cg05752891 | chr3 | 185,282,074 | 185,282,076 | *EHHADH;MAP3K13* | -0.079 | (0.012) | 1.8E-09 | -0.022 | 9.8E-05 |
| Epipodophyllotoxins | cg25788793 | chr4 | 10,032,955 | 10,032,957 | *SLC2A9* | -0.062 | (0.010) | 1.9E-09 | -0.018 | 8.0E-05 |
| Epipodophyllotoxins | cg08657492 | chr7 | 27,131,212 | 27,131,214 | *HOXA-AS2;HOXA-AS3;HOXA3;HOXA4;RP1-170O19.22* | 0.148 | (0.023) | 1.9E-09 | 0.035 | 1.1E-03 |
| Epipodophyllotoxins | cg18419358 | chr6 | 157,962,976 | 157,962,978 | *NA* | -0.106 | (0.016) | 1.9E-09 | -0.031 | 5.6E-05 |
| Epipodophyllotoxins | cg15890546 | chr3 | 194,967,758 | 194,967,760 | *NA* | -0.066 | (0.010) | 2.0E-09 | -0.016 | 6.0E-04 |
| Epipodophyllotoxins | cg12351310 | chr5 | 53,642,161 | 53,642,163 | *NDUFS4* | -0.069 | (0.011) | 2.0E-09 | -0.018 | 3.0E-04 |
| Epipodophyllotoxins | cg19975025 | chr12 | 88,309,753 | 88,309,755 | *NA* | -0.081 | (0.013) | 2.1E-09 | -0.026 | 1.1E-05 |
| Epipodophyllotoxins | cg07097098 | chr19 | 55,210,269 | 55,210,271 | *PTPRH* | -0.067 | (0.010) | 2.2E-09 | -0.019 | 9.5E-05 |
| Epipodophyllotoxins | cg16586518 | chr3 | 46,998,723 | 46,998,725 | *NBEAL2* | 0.051 | (0.008) | 2.2E-09 | 0.016 | 3.2E-05 |
| Epipodophyllotoxins | cg11449146 | chr11 | 22,728,917 | 22,728,919 | *GAS2* | -0.126 | (0.019) | 2.3E-09 | -0.044 | 1.6E-06 |
| Epipodophyllotoxins | cg14172524 | chr6 | 105,726,868 | 105,726,870 | *NA* | -0.064 | (0.010) | 2.4E-09 | -0.019 | 5.4E-05 |
| Epipodophyllotoxins | cg05614059 | chr14 | 52,313,667 | 52,313,669 | *PTGER2* | 0.094 | (0.015) | 2.4E-09 | 0.029 | 2.2E-05 |
| Epipodophyllotoxins | cg23956565 | chr5 | 73,770,813 | 73,770,815 | *ARHGEF28* | -0.069 | (0.011) | 2.5E-09 | -0.018 | 3.0E-04 |
| Epipodophyllotoxins | cg07051715 | chr12 | 14,654,652 | 14,654,654 | *GUCY2C* | -0.073 | (0.011) | 2.5E-09 | -0.021 | 6.5E-05 |
| Epipodophyllotoxins | cg17674228 | chr10 | 72,688,280 | 72,688,282 | *NA* | -0.086 | (0.013) | 2.5E-09 | -0.026 | 3.5E-05 |
| Epipodophyllotoxins | cg19550755 | chr11 | 18,773,348 | 18,773,350 | *PTPN5* | 0.052 | (0.008) | 2.5E-09 | 0.021 | 4.4E-08 |
| Epipodophyllotoxins | cg10558233 | chr8 | 93,880,384 | 93,880,386 | *PDP1* | -0.086 | (0.013) | 2.6E-09 | -0.024 | 9.7E-05 |
| Epipodophyllotoxins | cg01991968 | chr10 | 94,287,934 | 94,287,936 | *PLCE1;PLCE1-AS1* | -0.042 | (0.007) | 2.7E-09 | -0.015 | 2.0E-06 |
| Epipodophyllotoxins | cg01435315 | chr10 | 70,588,663 | 70,588,665 | *NA* | -0.092 | (0.014) | 2.8E-09 | -0.019 | 6.0E-03 |
| Epipodophyllotoxins | cg04188877 | chr22 | 42,721,943 | 42,721,945 | *A4GALT* | 0.060 | (0.009) | 2.8E-09 | 0.019 | 1.6E-05 |
| Epipodophyllotoxins | cg25952581 | chr7 | 27,131,341 | 27,131,343 | *HOXA-AS2;HOXA-AS3;HOXA3;HOXA4;RP1-170O19.22* | 0.150 | (0.023) | 2.8E-09 | 0.035 | 1.4E-03 |
| Epipodophyllotoxins | cg18761994 | chr6 | 24,911,097 | 24,911,099 | *RIPOR2* | 0.163 | (0.025) | 3.0E-09 | 0.036 | 2.6E-03 |
| Epipodophyllotoxins | cg08173263 | chr19 | 14,166,098 | 14,166,100 | *ADGRL1;CTB-55O6.12* | 0.113 | (0.018) | 3.0E-09 | 0.026 | 1.9E-03 |
| Epipodophyllotoxins | cg03287111 | chr2 | 120,868,058 | 120,868,060 | *GLI2;RP11-297J22.1* | 0.120 | (0.019) | 3.0E-09 | 0.023 | 8.8E-03 |
| Epipodophyllotoxins | cg02160714 | chr3 | 65,334,305 | 65,334,307 | *NA* | -0.053 | (0.008) | 3.0E-09 | -0.020 | 2.9E-07 |
| Epipodophyllotoxins | cg15298714 | chr2 | 235,505,551 | 235,505,553 | *AGAP1;AGAP1-IT1* | 0.094 | (0.015) | 3.3E-09 | 0.032 | 5.3E-06 |
| Epipodophyllotoxins | cg19142026 | chr7 | 27,130,774 | 27,130,776 | *HOXA-AS2;HOXA-AS3;HOXA3;HOXA4;RP1-170O19.22* | 0.236 | (0.037) | 3.7E-09 | 0.058 | 8.2E-04 |
| Epipodophyllotoxins | cg20745261 | chr8 | 118,649,968 | 118,649,970 | *SAMD12-AS1* | -0.075 | (0.012) | 3.8E-09 | -0.019 | 5.1E-04 |
| Epipodophyllotoxins | cg24990672 | chr3 | 186,359,107 | 186,359,109 | *DGKG* | -0.043 | (0.007) | 3.8E-09 | -0.015 | 2.7E-06 |
| Epipodophyllotoxins | cg01329519 | chr5 | 53,303,201 | 53,303,203 | *NA* | -0.105 | (0.017) | 4.0E-09 | -0.039 | 5.0E-07 |
| Epipodophyllotoxins | cg19567594 | chr11 | 10,693,627 | 10,693,629 | *MRVI1* | 0.074 | (0.012) | 4.0E-09 | 0.014 | 1.1E-02 |
| Epipodophyllotoxins | cg22573230 | chr17 | 7,745,952 | 7,745,954 | *DNAH2* | -0.061 | (0.010) | 4.3E-09 | -0.017 | 1.6E-04 |
| Epipodophyllotoxins | cg13491413 | chr6 | 24,910,882 | 24,910,884 | *RIPOR2* | 0.096 | (0.015) | 4.5E-09 | 0.025 | 5.0E-04 |
| Epipodophyllotoxins | cg04338129 | chr11 | 65,867,919 | 65,867,921 | *EFEMP2* | 0.080 | (0.013) | 4.5E-09 | 0.021 | 3.6E-04 |
| Epipodophyllotoxins | cg05333719 | chr15 | 74,629,117 | 74,629,119 | *CLK3* | 0.044 | (0.007) | 4.5E-09 | 0.012 | 2.6E-04 |
| Epipodophyllotoxins | cg27121267 | chr11 | 125,674,396 | 125,674,398 | *ACRV1;CHEK1* | -0.051 | (0.008) | 4.6E-09 | -0.017 | 4.0E-06 |
| Epipodophyllotoxins | cg17038235 | chr5 | 172,943,520 | 172,943,522 | *ERGIC1* | 0.060 | (0.010) | 4.9E-09 | 0.019 | 2.4E-05 |
| Epipodophyllotoxins | cg06808571 | chr7 | 150,945,167 | 150,945,169 | *KCNH2* | 0.066 | (0.010) | 5.0E-09 | 0.021 | 1.9E-05 |
| Epipodophyllotoxins | cg10938436 | chr11 | 124,746,169 | 124,746,171 | *NRGN;RP11-677M14.2* | 0.108 | (0.017) | 5.7E-09 | 0.039 | 1.1E-06 |
| Epipodophyllotoxins | cg20061837 | chr5 | 28,734,730 | 28,734,732 | *NA* | -0.090 | (0.014) | 5.9E-09 | -0.032 | 2.0E-06 |
| Epipodophyllotoxins | cg03116409 | chr12 | 65,648,773 | 65,648,775 | *RP11-221N13.4* | -0.092 | (0.015) | 6.0E-09 | -0.029 | 2.4E-05 |
| Epipodophyllotoxins | cg20251080 | chr8 | 122,489,796 | 122,489,798 | *RP11-96B2.1* | 0.069 | (0.011) | 6.1E-09 | 0.016 | 1.8E-03 |
| Epipodophyllotoxins | cg24630035 | chr5 | 121,053,366 | 121,053,368 | *NA* | -0.238 | (0.038) | 6.3E-09 | -0.096 | 6.0E-08 |
| Epipodophyllotoxins | cg21050234 | chr18 | 10,726,519 | 10,726,521 | *PIEZO2;RP11-21G15.1* | 0.114 | (0.018) | 6.4E-09 | 0.040 | 3.9E-06 |
| Epipodophyllotoxins | cg13224583 | chr1 | 156,913,952 | 156,913,954 | *PEAR1* | 0.065 | (0.010) | 6.6E-09 | 0.011 | 1.9E-02 |
| Epipodophyllotoxins | cg14398390 | chr2 | 120,780,507 | 120,780,509 | *GLI2* | 0.073 | (0.012) | 6.6E-09 | 0.020 | 3.8E-04 |
| Epipodophyllotoxins | cg01751245 | chr2 | 65,366,626 | 65,366,628 | *SPRED2* | -0.094 | (0.015) | 6.7E-09 | -0.028 | 7.2E-05 |
| Epipodophyllotoxins | cg18230175 | chr3 | 192,802,836 | 192,802,838 | *MB21D2* | 0.076 | (0.012) | 6.9E-09 | 0.025 | 1.2E-05 |
| Epipodophyllotoxins | cg19611364 | chr19 | 13,024,679 | 13,024,681 | *NFIX* | 0.102 | (0.016) | 7.1E-09 | 0.022 | 4.2E-03 |
| Epipodophyllotoxins | cg17988780 | chr6 | 30,731,002 | 30,731,004 | *FLOT1* | 0.101 | (0.016) | 7.3E-09 | 0.033 | 1.5E-05 |
| Epipodophyllotoxins | cg08774778 | chr5 | 62,082,559 | 62,082,561 | *NA* | -0.079 | (0.013) | 7.4E-09 | -0.013 | 2.6E-02 |
| Epipodophyllotoxins | cg25953130 | chr10 | 61,993,790 | 61,993,792 | *ARID5B* | -0.085 | (0.014) | 7.6E-09 | -0.028 | 1.3E-05 |
| Epipodophyllotoxins | cg05478824 | chr17 | 82,012,258 | 82,012,260 | *ASPSCR1* | 0.064 | (0.010) | 7.7E-09 | 0.016 | 7.3E-04 |
| Epipodophyllotoxins | cg11795881 | chr7 | 93,245,897 | 93,245,899 | *VPS50* | -0.091 | (0.015) | 7.8E-09 | -0.016 | 1.6E-02 |
| Epipodophyllotoxins | cg24718465 | chr19 | 46,495,860 | 46,495,862 | *CTB-158D10.3;PNMA8B;PPP5D1* | 0.076 | (0.012) | 7.9E-09 | 0.028 | 1.4E-06 |
| Epipodophyllotoxins | cg05038391 | chr7 | 73,774,121 | 73,774,123 | *NA* | -0.044 | (0.007) | 8.1E-09 | -0.011 | 9.1E-04 |
| Epipodophyllotoxins | cg01095763 | chr2 | 236,338,961 | 236,338,963 | *IQCA1* | 0.105 | (0.017) | 8.1E-09 | 0.033 | 3.6E-05 |
| Epipodophyllotoxins | cg14547509 | chr9 | 14,318,498 | 14,318,500 | *NFIB;RP11-120J1.1* | -0.066 | (0.011) | 8.1E-09 | -0.023 | 3.6E-06 |
| Epipodophyllotoxins | cg01418153 | chr1 | 3,323,564 | 3,323,566 | *PRDM16* | 0.083 | (0.013) | 8.3E-09 | 0.022 | 3.8E-04 |
| Epipodophyllotoxins | cg17858880 | chr7 | 47,537,261 | 47,537,263 | *TNS3* | 0.067 | (0.011) | 8.7E-09 | 0.014 | 6.3E-03 |
| Epipodophyllotoxins | cg23889967 | chr10 | 129,770,048 | 129,770,050 | *NA* | 0.075 | (0.012) | 8.7E-09 | 0.023 | 3.9E-05 |
| Epipodophyllotoxins | cg08540433 | chr3 | 186,098,165 | 186,098,167 | *ETV5* | 0.086 | (0.014) | 8.8E-09 | 0.016 | 1.7E-02 |
| Epipodophyllotoxins | cg08791563 | chr2 | 66,508,921 | 66,508,923 | *MEIS1* | 0.116 | (0.019) | 9.0E-09 | 0.030 | 7.7E-04 |
| Epipodophyllotoxins | cg00891669 | chr4 | 26,812,388 | 26,812,390 | *NA* | -0.075 | (0.012) | 9.1E-09 | -0.020 | 4.5E-04 |
| Epipodophyllotoxins | cg25371036 | chr11 | 94,767,582 | 94,767,584 | *AMOTL1* | -0.054 | (0.009) | 9.2E-09 | -0.015 | 3.3E-04 |
| Epipodophyllotoxins | cg23119026 | chr2 | 20,577,069 | 20,577,071 | *HS1BP3* | -0.047 | (0.008) | 9.4E-09 | -0.015 | 1.2E-05 |
| Epipodophyllotoxins | cg26225814 | chr14 | 100,019,357 | 100,019,359 | *EVL* | 0.086 | (0.014) | 9.7E-09 | 0.024 | 2.2E-04 |
| Epipodophyllotoxins | cg08349425 | chr2 | 232,293,121 | 232,293,123 | *DIS3L2* | -0.050 | (0.008) | 9.7E-09 | -0.016 | 1.8E-05 |
| Epipodophyllotoxins | cg13866747 | chr13 | 91,313,553 | 91,313,555 | *NA* | -0.109 | (0.018) | 9.8E-09 | -0.028 | 8.6E-04 |
| Epipodophyllotoxins | cg17591595 | chr7 | 27,131,431 | 27,131,433 | *HOXA-AS2;HOXA-AS3;HOXA3;HOXA4;RP1-170O19.22* | 0.119 | (0.019) | 1.0E-08 | 0.025 | 5.1E-03 |
| Epipodophyllotoxins | cg23567872 | chr14 | 38,290,705 | 38,290,707 | *RP11-96D24.1* | -0.125 | (0.020) | 1.0E-08 | -0.056 | 3.3E-09 |
| Epipodophyllotoxins | cg22706402 | chr9 | 81,875,144 | 81,875,146 | *NA* | -0.038 | (0.006) | 1.1E-08 | -0.014 | 1.6E-06 |
| Epipodophyllotoxins | cg11077681 | chr11 | 10,693,640 | 10,693,642 | *MRVI1* | 0.067 | (0.011) | 1.1E-08 | 0.011 | 2.5E-02 |
| Epipodophyllotoxins | cg27326027 | chr18 | 6,885,253 | 6,885,255 | *ARHGAP28* | -0.098 | (0.016) | 1.1E-08 | -0.026 | 5.1E-04 |
| Epipodophyllotoxins | cg25236398 | chr12 | 9,775,728 | 9,775,730 | *NA* | -0.083 | (0.013) | 1.1E-08 | -0.033 | 2.4E-07 |
| Epipodophyllotoxins | cg07065756 | chr7 | 2,079,704 | 2,079,706 | *MAD1L1* | 0.074 | (0.012) | 1.1E-08 | 0.023 | 4.3E-05 |
| Epipodophyllotoxins | cg03090301 | chr4 | 87,498,595 | 87,498,597 | *SPARCL1* | -0.069 | (0.011) | 1.1E-08 | -0.018 | 6.0E-04 |
| Epipodophyllotoxins | cg22432387 | chr2 | 20,634,604 | 20,634,606 | *HS1BP3* | -0.114 | (0.019) | 1.2E-08 | -0.031 | 4.4E-04 |
| Epipodophyllotoxins | cg09925014 | chr2 | 120,737,656 | 120,737,658 | *GLI2* | -0.050 | (0.008) | 1.2E-08 | -0.015 | 1.1E-04 |
| Epipodophyllotoxins | cg13590413 | chr6 | 30,640,665 | 30,640,667 | *ATAT1* | 0.050 | (0.008) | 1.2E-08 | 0.020 | 3.1E-07 |
| Epipodophyllotoxins | cg08662753 | chr9 | 134,669,653 | 134,669,655 | *COL5A1* | 0.069 | (0.011) | 1.3E-08 | 0.022 | 3.1E-05 |
| Epipodophyllotoxins | cg07363855 | chr1 | 3,067,905 | 3,067,907 | *LINC00982;PRDM16* | 0.054 | (0.009) | 1.4E-08 | 0.020 | 1.8E-06 |
| Epipodophyllotoxins | cg16288101 | chr14 | 88,155,193 | 88,155,195 | *RP11-753D20.1* | -0.116 | (0.019) | 1.4E-08 | -0.054 | 1.2E-09 |
| Epipodophyllotoxins | cg05135521 | chr2 | 160,331,823 | 160,331,825 | *RBMS1* | -0.075 | (0.012) | 1.5E-08 | -0.022 | 1.2E-04 |
| Epipodophyllotoxins | cg26237233 | chr11 | 81,071,020 | 81,071,022 | *NA* | -0.048 | (0.008) | 1.7E-08 | -0.014 | 2.3E-04 |
| Epipodophyllotoxins | cg21707607 | chr7 | 2,115,842 | 2,115,844 | *MAD1L1* | 0.081 | (0.013) | 1.7E-08 | 0.031 | 7.0E-07 |
| Epipodophyllotoxins | cg00889363 | chr2 | 205,391,116 | 205,391,118 | *PARD3B* | -0.043 | (0.007) | 1.7E-08 | -0.009 | 8.2E-03 |
| Epipodophyllotoxins | cg07349633 | chr1 | 231,493,745 | 231,493,747 | *NA* | -0.090 | (0.015) | 1.7E-08 | -0.023 | 1.1E-03 |
| Epipodophyllotoxins | cg01754713 | chr16 | 31,215,012 | 31,215,014 | *TRIM72* | 0.043 | (0.007) | 1.8E-08 | 0.013 | 1.5E-04 |
| Epipodophyllotoxins | cg26316423 | chr10 | 6,062,173 | 6,062,175 | *IL2RA* | -0.062 | (0.010) | 1.8E-08 | -0.014 | 4.0E-03 |
| Epipodophyllotoxins | cg00931181 | chr2 | 6,770,499 | 6,770,501 | *LINC00487* | -0.070 | (0.011) | 1.8E-08 | -0.016 | 4.0E-03 |
| Epipodophyllotoxins | cg13891045 | chr17 | 37,793,028 | 37,793,030 | *NA* | -0.047 | (0.008) | 1.8E-08 | -0.012 | 1.1E-03 |
| Epipodophyllotoxins | cg14792781 | chr2 | 9,976,114 | 9,976,116 | *GRHL1* | -0.057 | (0.009) | 1.8E-08 | -0.019 | 9.8E-06 |
| Epipodophyllotoxins | cg12233463 | chr15 | 81,387,225 | 81,387,227 | *NA* | -0.073 | (0.012) | 1.9E-08 | -0.018 | 1.1E-03 |
| Epipodophyllotoxins | cg00432659 | chr18 | 11,151,283 | 11,151,285 | *NA* | 0.074 | (0.012) | 1.9E-08 | 0.019 | 7.8E-04 |
| Epipodophyllotoxins | cg05302489 | chr6 | 31,792,648 | 31,792,650 | *VARS* | 0.070 | (0.012) | 2.0E-08 | 0.022 | 3.6E-05 |
| Epipodophyllotoxins | cg12594228 | chr12 | 100,576,972 | 100,576,974 | *GAS2L3* | -0.076 | (0.013) | 2.1E-08 | -0.018 | 2.3E-03 |
| Epipodophyllotoxins | cg16983486 | chr11 | 8,832,696 | 8,832,698 | *ST5* | -0.042 | (0.007) | 2.1E-08 | -0.010 | 1.5E-03 |
| Epipodophyllotoxins | cg16423910 | chr19 | 49,340,369 | 49,340,371 | *CD37;CTC-301O7.4* | 0.079 | (0.013) | 2.1E-08 | 0.020 | 1.1E-03 |
| Epipodophyllotoxins | cg01013171 | chr14 | 75,249,665 | 75,249,667 | *RP11-293M10.1* | 0.061 | (0.010) | 2.1E-08 | 0.024 | 3.0E-07 |
| Epipodophyllotoxins | cg15833353 | chr5 | 33,807,172 | 33,807,174 | *ADAMTS12* | -0.046 | (0.008) | 2.2E-08 | -0.010 | 3.3E-03 |
| Epipodophyllotoxins | cg03868978 | chr21 | 41,165,366 | 41,165,368 | *NA* | -0.057 | (0.009) | 2.2E-08 | -0.012 | 7.9E-03 |
| Epipodophyllotoxins | cg04368942 | chr7 | 134,886,568 | 134,886,570 | *CALD1* | -0.062 | (0.010) | 2.2E-08 | -0.022 | 4.6E-06 |
| Epipodophyllotoxins | cg10473623 | chr11 | 20,097,629 | 20,097,631 | *NAV2* | -0.077 | (0.013) | 2.2E-08 | -0.014 | 2.0E-02 |
| Epipodophyllotoxins | cg00713549 | chr7 | 41,630,893 | 41,630,895 | *NA* | -0.085 | (0.014) | 2.2E-08 | -0.021 | 1.8E-03 |
| Epipodophyllotoxins | cg10762064 | chr3 | 194,967,685 | 194,967,687 | *NA* | -0.069 | (0.011) | 2.2E-08 | -0.018 | 8.3E-04 |
| Epipodophyllotoxins | cg09092525 | chr20 | 59,995,077 | 59,995,079 | *CDH26* | -0.063 | (0.010) | 2.3E-08 | -0.023 | 3.2E-06 |
| Epipodophyllotoxins | cg09276451 | chr16 | 4,371,601 | 4,371,603 | *CORO7;CORO7-PAM16;VASN* | 0.064 | (0.011) | 2.3E-08 | 0.021 | 3.2E-05 |
| Epipodophyllotoxins | cg13449606 | chr1 | 27,359,836 | 27,359,838 | *MAP3K6* | 0.074 | (0.012) | 2.5E-08 | 0.021 | 3.3E-04 |
| Epipodophyllotoxins | cg27353963 | chr5 | 157,025,426 | 157,025,428 | *NA* | -0.045 | (0.007) | 2.5E-08 | -0.020 | 1.6E-08 |
| Epipodophyllotoxins | cg04968127 | chr11 | 10,693,665 | 10,693,667 | *MRVI1* | 0.060 | (0.010) | 2.6E-08 | 0.013 | 7.8E-03 |
| Epipodophyllotoxins | cg21647621 | chr1 | 61,285,632 | 61,285,634 | *NFIA* | 0.060 | (0.010) | 2.7E-08 | 0.016 | 9.1E-04 |
| Epipodophyllotoxins | cg12301696 | chr11 | 2,213,779 | 2,213,781 | *NA* | 0.073 | (0.012) | 2.7E-08 | 0.019 | 7.0E-04 |
| Epipodophyllotoxins | cg03969996 | chr19 | 46,495,797 | 46,495,799 | *CTB-158D10.3;PNMA8B;PPP5D1* | 0.111 | (0.019) | 2.8E-08 | 0.039 | 9.9E-06 |
| Epipodophyllotoxins | cg25425537 | chr2 | 6,770,386 | 6,770,388 | *LINC00487* | -0.079 | (0.013) | 2.8E-08 | -0.020 | 9.0E-04 |
| Epipodophyllotoxins | cg17183905 | chr12 | 109,816,036 | 109,816,038 | *TRPV4* | -0.054 | (0.009) | 3.0E-08 | -0.012 | 5.0E-03 |
| Epipodophyllotoxins | cg13817952 | chr9 | 122,228,176 | 122,228,178 | *LHX6* | 0.260 | (0.043) | 3.0E-08 | 0.077 | 1.8E-04 |
| Epipodophyllotoxins | cg04823169 | chr9 | 98,088,051 | 98,088,053 | *TRIM14* | 0.121 | (0.020) | 3.1E-08 | 0.035 | 2.1E-04 |
| Epipodophyllotoxins | cg09022082 | chr1 | 236,107,284 | 236,107,286 | *NA* | -0.053 | (0.009) | 3.1E-08 | -0.017 | 3.9E-05 |
| Epipodophyllotoxins | cg24007583 | chr16 | 4,371,576 | 4,371,578 | *CORO7;CORO7-PAM16;VASN* | 0.049 | (0.008) | 3.2E-08 | 0.015 | 5.2E-05 |
| Epipodophyllotoxins | cg17954090 | chr1 | 115,728,650 | 115,728,652 | *CASQ2* | -0.039 | (0.007) | 3.3E-08 | -0.009 | 2.6E-03 |
| Epipodophyllotoxins | cg22619104 | chr20 | 1,871,207 | 1,871,209 | *NA* | 0.073 | (0.012) | 3.4E-08 | 0.016 | 5.3E-03 |
| Epipodophyllotoxins | cg01379386 | chr21 | 29,145,392 | 29,145,394 | *MAP3K7CL* | -0.082 | (0.014) | 3.4E-08 | -0.025 | 1.4E-04 |
| Epipodophyllotoxins | cg21513437 | chr17 | 41,521,854 | 41,521,856 | *KRT15* | -0.040 | (0.007) | 3.4E-08 | -0.008 | 8.6E-03 |
| Epipodophyllotoxins | cg02606058 | chr16 | 67,161,754 | 67,161,756 | *FBXL8;TRADD* | 0.040 | (0.007) | 3.5E-08 | 0.012 | 8.8E-05 |
| Epipodophyllotoxins | cg04206986 | chr2 | 51,997,113 | 51,997,115 | *AC007682.1* | -0.046 | (0.008) | 3.6E-08 | -0.015 | 5.3E-05 |
| Epipodophyllotoxins | cg15170715 | chr2 | 27,086,008 | 27,086,010 | *EMILIN1;KHK* | 0.088 | (0.015) | 3.6E-08 | 0.016 | 2.2E-02 |
| Epipodophyllotoxins | cg06562165 | chr18 | 55,511,189 | 55,511,191 | *TCF4* | -0.062 | (0.010) | 3.7E-08 | -0.021 | 2.2E-05 |
| Epipodophyllotoxins | cg12753787 | chr1 | 3,323,147 | 3,323,149 | *PRDM16* | 0.040 | (0.007) | 3.7E-08 | 0.011 | 6.5E-04 |
| Epipodophyllotoxins | cg03764753 | chr2 | 151,306,640 | 151,306,642 | *NA* | -0.092 | (0.016) | 3.8E-08 | -0.030 | 5.1E-05 |
| Epipodophyllotoxins | cg04005059 | chr2 | 23,496,967 | 23,496,969 | *KLHL29* | -0.062 | (0.010) | 3.9E-08 | -0.013 | 7.8E-03 |
| Epipodophyllotoxins | cg08336473 | chr13 | 33,353,344 | 33,353,346 | *NA* | -0.074 | (0.012) | 3.9E-08 | -0.021 | 2.7E-04 |
| Epipodophyllotoxins | cg17696044 | chr11 | 70,603,210 | 70,603,212 | *RP11-826F13.1;SHANK2* | 0.100 | (0.017) | 4.0E-08 | 0.027 | 6.6E-04 |
| Epipodophyllotoxins | cg00601810 | chr2 | 188,496,176 | 188,496,178 | *GULP1* | -0.105 | (0.018) | 4.0E-08 | -0.032 | 1.6E-04 |
| Epipodophyllotoxins | cg19936032 | chr6 | 24,911,185 | 24,911,187 | *RIPOR2* | 0.066 | (0.011) | 4.0E-08 | 0.011 | 3.0E-02 |
| Epipodophyllotoxins | cg20308138 | chr5 | 67,301,693 | 67,301,695 | *NA* | -0.042 | (0.007) | 4.3E-08 | -0.011 | 1.4E-03 |
| Epipodophyllotoxins | cg25360591 | chr5 | 164,353,338 | 164,353,340 | *CTC-340A15.2* | -0.106 | (0.018) | 4.4E-08 | -0.036 | 2.2E-05 |
| Epipodophyllotoxins | cg05723839 | chr11 | 76,885,902 | 76,885,904 | *ACER3* | -0.046 | (0.008) | 4.7E-08 | -0.018 | 1.1E-06 |
| Epipodophyllotoxins | cg09835575 | chr20 | 9,985,215 | 9,985,217 | *ANKEF1;RP5-839B4.8* | -0.082 | (0.014) | 4.8E-08 | -0.028 | 2.4E-05 |
| Epipodophyllotoxins | cg06647693 | chr16 | 88,523,931 | 88,523,933 | *RP11-21B21.4;ZFPM1* | 0.028 | (0.005) | 4.8E-08 | 0.009 | 1.0E-04 |
| Epipodophyllotoxins | cg20689675 | chr18 | 47,284,782 | 47,284,784 | *CTD-2130O13.1* | -0.048 | (0.008) | 4.9E-08 | -0.012 | 1.5E-03 |
| Epipodophyllotoxins | cg01622416 | chr21 | 29,938,189 | 29,938,191 | *GRIK1* | -0.083 | (0.014) | 5.1E-08 | -0.031 | 3.7E-06 |
| Epipodophyllotoxins | cg22156456 | chr17 | 41,687,986 | 41,687,988 | *EIF1* | -0.052 | (0.009) | 5.1E-08 | -0.012 | 3.9E-03 |
| Epipodophyllotoxins | cg05506147 | chr5 | 96,614,413 | 96,614,415 | *CAST;CTD-2337A12.1* | -0.066 | (0.011) | 5.2E-08 | -0.023 | 2.0E-05 |
| Epipodophyllotoxins | cg11414254 | chr5 | 177,022,010 | 177,022,012 | *UIMC1;ZNF346* | -0.075 | (0.013) | 5.3E-08 | -0.022 | 3.0E-04 |
| Epipodophyllotoxins | cg13474360 | chr3 | 192,885,619 | 192,885,621 | *MB21D2* | 0.054 | (0.009) | 5.3E-08 | 0.016 | 2.4E-04 |
| Epipodophyllotoxins | cg20492091 | chr4 | 2,906,740 | 2,906,742 | *ADD1* | 0.063 | (0.011) | 5.4E-08 | 0.022 | 1.9E-05 |
| Epipodophyllotoxins | cg20778199 | chr6 | 147,699,744 | 147,699,746 | *RP11-307P5.1;SAMD5* | -0.077 | (0.013) | 5.5E-08 | -0.024 | 1.0E-04 |
| Epipodophyllotoxins | cg22108243 | chr1 | 68,725,462 | 68,725,464 | *NA* | -0.100 | (0.017) | 5.5E-08 | -0.031 | 9.0E-05 |
| Epipodophyllotoxins | cg07093259 | chr9 | 84,152,315 | 84,152,317 | *NA* | -0.057 | (0.010) | 5.6E-08 | -0.020 | 1.0E-05 |
| Epipodophyllotoxins | cg27330053 | chr2 | 152,200,783 | 152,200,785 | *NA* | -0.040 | (0.007) | 5.6E-08 | -0.009 | 3.0E-03 |
| Epipodophyllotoxins | cg09153462 | chr8 | 61,690,083 | 61,690,085 | *ASPH* | -0.069 | (0.012) | 5.6E-08 | -0.026 | 2.5E-06 |
| Epipodophyllotoxins | cg25744287 | chr1 | 26,961,627 | 26,961,629 | *KDF1* | -0.053 | (0.009) | 5.7E-08 | -0.017 | 6.9E-05 |
| Epipodophyllotoxins | cg10971669 | chr12 | 8,066,821 | 8,066,823 | *C3AR1* | -0.070 | (0.012) | 6.0E-08 | -0.022 | 7.2E-05 |
| Epipodophyllotoxins | cg20970886 | chr3 | 192,850,924 | 192,850,926 | *MB21D2* | -0.062 | (0.011) | 6.2E-08 | -0.016 | 1.6E-03 |
| Epipodophyllotoxins | cg02385703 | chr1 | 48,082,228 | 48,082,230 | *RP11-193P11.3* | -0.043 | (0.007) | 6.5E-08 | -0.013 | 1.5E-04 |
| Epipodophyllotoxins | cg17153045 | chr3 | 157,120,438 | 157,120,440 | *LINC00880* | 0.065 | (0.011) | 6.6E-08 | 0.019 | 3.5E-04 |
| Epipodophyllotoxins | cg00525823 | chr18 | 11,148,510 | 11,148,512 | *PIEZO2* | -0.045 | (0.008) | 6.7E-08 | -0.016 | 6.6E-06 |
| Epipodophyllotoxins | cg11613118 | chr19 | 4,586,765 | 4,586,767 | *NA* | -0.057 | (0.010) | 6.9E-08 | -0.014 | 2.7E-03 |
| Epipodophyllotoxins | cg23117180 | chr1 | 158,003,607 | 158,003,609 | *KIRREL1* | 0.060 | (0.010) | 7.1E-08 | 0.014 | 2.9E-03 |
| Epipodophyllotoxins | cg13553455 | chr10 | 104,086,243 | 104,086,245 | *COL17A1* | -0.046 | (0.008) | 7.1E-08 | -0.010 | 8.4E-03 |
| Epipodophyllotoxins | cg06192447 | chr11 | 18,793,697 | 18,793,699 | *PTPN5* | 0.041 | (0.007) | 7.5E-08 | 0.012 | 2.6E-04 |
| Epipodophyllotoxins | cg22279159 | chr4 | 26,197,646 | 26,197,648 | *RBPJ* | -0.082 | (0.014) | 7.5E-08 | -0.022 | 9.9E-04 |
| Epipodophyllotoxins | cg02601188 | chr5 | 175,876,869 | 175,876,871 | *CPLX2* | -0.088 | (0.015) | 7.5E-08 | -0.021 | 4.0E-03 |
| Epipodophyllotoxins | cg08927146 | chr22 | 20,395,754 | 20,395,756 | *ZNF74* | 0.052 | (0.009) | 7.6E-08 | 0.018 | 1.5E-05 |
| Epipodophyllotoxins | cg25695450 | chr7 | 41,219,438 | 41,219,440 | *NA* | -0.069 | (0.012) | 7.7E-08 | -0.020 | 2.7E-04 |
| Epipodophyllotoxins | cg06991118 | chr18 | 9,117,349 | 9,117,351 | *NDUFV2;RP11-143J12.3;RP11-21J18.1* | -0.089 | (0.015) | 7.9E-08 | -0.029 | 4.2E-05 |
| Epipodophyllotoxins | cg16750801 | chr10 | 127,908,048 | 127,908,050 | *PTPRE* | -0.067 | (0.012) | 7.9E-08 | -0.025 | 4.9E-06 |
| Epipodophyllotoxins | cg21192063 | chr7 | 151,251,599 | 151,251,601 | *RP5-1070G24.2;SMARCD3* | 0.105 | (0.018) | 8.2E-08 | 0.029 | 7.9E-04 |
| Epipodophyllotoxins | cg00951302 | chr6 | 165,718,496 | 165,718,498 | *PDE10A;RNU6-730P* | -0.062 | (0.011) | 8.4E-08 | -0.019 | 1.6E-04 |
| ***Radiation therapy*** | |  |  |  |  |  |  |  |  |  |
| Abdomen-RT | cg13294852 | chr2 | 102,055,667 | 102,055,669 | *NA* | -0.178 | (0.015) | 5.5E-21 | -0.118 | 8.1E-21 |
| Abdomen-RT | cg13224583 | chr1 | 156,913,952 | 156,913,954 | *PEAR1* | 0.142 | (0.012) | 6.8E-21 | 0.092 | 6.3E-20 |
| Abdomen-RT | cg21945120 | chr10 | 58,157,562 | 58,157,564 | *NA* | -0.161 | (0.014) | 7.1E-20 | -0.104 | 6.5E-19 |
| Abdomen-RT | cg08271909 | chr4 | 154,740,538 | 154,740,540 | *LRAT* | 0.177 | (0.016) | 1.1E-18 | 0.114 | 1.5E-17 |
| Abdomen-RT | cg07872945 | chr9 | 134,010,913 | 134,010,915 | *NA* | -0.142 | (0.013) | 1.6E-18 | -0.092 | 1.4E-17 |
| Abdomen-RT | cg22432387 | chr2 | 20,634,604 | 20,634,606 | *HS1BP3* | -0.236 | (0.021) | 2.9E-18 | -0.166 | 2.3E-20 |
| Abdomen-RT | cg01511232 | chr4 | 154,740,776 | 154,740,778 | *LRAT* | 0.211 | (0.019) | 3.0E-18 | 0.138 | 1.3E-17 |
| Abdomen-RT | cg08866213 | chr3 | 192,812,987 | 192,812,989 | *MB21D2* | 0.206 | (0.018) | 3.4E-18 | 0.135 | 9.5E-18 |
| Abdomen-RT | cg16985233 | chr15 | 43,514,050 | 43,514,052 | *MAP1A* | -0.177 | (0.016) | 6.0E-18 | -0.120 | 1.4E-18 |
| Abdomen-RT | cg03834031 | chr22 | 46,069,836 | 46,069,838 | *MIRLET7BHG;RP6-109B7.4* | -0.101 | (0.009) | 9.4E-18 | -0.067 | 2.0E-17 |
| Abdomen-RT | cg26342454 | chr4 | 10,033,637 | 10,033,639 | *SLC2A9* | -0.282 | (0.026) | 3.7E-17 | -0.189 | 1.7E-17 |
| Abdomen-RT | cg00973876 | chr17 | 79,925,408 | 79,925,410 | *RP11-353N14.4;RP11-353N14.5* | -0.125 | (0.012) | 6.1E-17 | -0.086 | 5.8E-18 |
| Abdomen-RT | cg16112727 | chr1 | 156,913,429 | 156,913,431 | *PEAR1* | 0.106 | (0.010) | 1.2E-16 | 0.071 | 5.5E-17 |
| Abdomen-RT | cg26621020 | chr8 | 122,647,819 | 122,647,821 | *NA* | -0.188 | (0.018) | 1.2E-16 | -0.124 | 2.4E-16 |
| Abdomen-RT | cg17593512 | chr4 | 40,439,495 | 40,439,497 | *RBM47* | -0.181 | (0.017) | 1.3E-16 | -0.123 | 2.8E-17 |
| Abdomen-RT | cg09921385 | chr4 | 6,999,154 | 6,999,156 | *TBC1D14* | -0.165 | (0.016) | 2.2E-16 | -0.115 | 5.8E-18 |
| Abdomen-RT | cg11988169 | chr5 | 53,543,411 | 53,543,413 | *NA* | -0.360 | (0.034) | 2.5E-16 | -0.235 | 8.8E-16 |
| Abdomen-RT | cg17183905 | chr12 | 109,816,036 | 109,816,038 | *TRPV4* | -0.105 | (0.010) | 9.9E-16 | -0.071 | 4.5E-16 |
| Abdomen-RT | cg06991974 | chr1 | 3,067,653 | 3,067,655 | *LINC00982* | 0.125 | (0.012) | 1.2E-15 | 0.076 | 1.7E-13 |
| Abdomen-RT | cg11504495 | chr7 | 48,088,550 | 48,088,552 | *UPP1* | -0.190 | (0.018) | 1.3E-15 | -0.131 | 7.2E-17 |
| Abdomen-RT | cg24765521 | chr9 | 90,296,803 | 90,296,805 | *NA* | -0.196 | (0.019) | 1.8E-15 | -0.128 | 5.1E-15 |
| Abdomen-RT | cg21585138 | chr3 | 50,607,674 | 50,607,676 | *CISH* | -0.140 | (0.014) | 2.1E-15 | -0.086 | 3.2E-13 |
| Abdomen-RT | cg11662039 | chr5 | 100,648,041 | 100,648,043 | *NA* | -0.179 | (0.018) | 2.4E-15 | -0.124 | 1.4E-16 |
| Abdomen-RT | cg27589809 | chr3 | 50,612,978 | 50,612,980 | *CISH;MAPKAPK3* | -0.098 | (0.010) | 2.7E-15 | -0.063 | 1.8E-14 |
| Abdomen-RT | cg21242123 | chr12 | 52,244,349 | 52,244,351 | *KRT7* | -0.118 | (0.012) | 4.0E-15 | -0.081 | 4.9E-16 |
| Abdomen-RT | cg06963130 | chr2 | 235,434,127 | 235,434,129 | *NA* | -0.147 | (0.015) | 5.6E-15 | -0.099 | 2.4E-15 |
| Abdomen-RT | cg01641136 | chr3 | 50,607,810 | 50,607,812 | *CISH* | -0.142 | (0.014) | 6.0E-15 | -0.090 | 1.3E-13 |
| Abdomen-RT | cg23776628 | chr1 | 39,633,108 | 39,633,110 | *HEYL;RP1-144F13.3* | 0.152 | (0.015) | 7.7E-15 | 0.097 | 9.7E-14 |
| Abdomen-RT | cg12140144 | chr1 | 3,067,710 | 3,067,712 | *LINC00982;PRDM16* | 0.369 | (0.037) | 7.7E-15 | 0.238 | 4.6E-14 |
| Abdomen-RT | cg27192248 | chr15 | 64,993,330 | 64,993,332 | *NA* | -0.287 | (0.029) | 8.7E-15 | -0.193 | 3.2E-15 |
| Abdomen-RT | cg09126320 | chr8 | 22,920,986 | 22,920,988 | *PEBP4* | -0.095 | (0.010) | 1.1E-14 | -0.064 | 6.3E-15 |
| Abdomen-RT | cg07086679 | chr7 | 140,316,321 | 140,316,323 | *SLC37A3* | -0.127 | (0.013) | 1.2E-14 | -0.086 | 5.2E-15 |
| Abdomen-RT | cg17200702 | chr11 | 30,992,376 | 30,992,378 | *DCDC1* | -0.173 | (0.017) | 1.4E-14 | -0.110 | 1.8E-13 |
| Abdomen-RT | cg03877706 | chr21 | 21,200,312 | 21,200,314 | *NCAM2* | -0.243 | (0.025) | 1.7E-14 | -0.154 | 2.7E-13 |
| Abdomen-RT | cg15143202 | chr3 | 169,148,736 | 169,148,738 | *MECOM* | -0.164 | (0.017) | 1.8E-14 | -0.106 | 9.5E-14 |
| Abdomen-RT | cg07946633 | chr1 | 3,067,680 | 3,067,682 | *LINC00982;PRDM16* | 0.100 | (0.010) | 2.0E-14 | 0.065 | 9.6E-14 |
| Abdomen-RT | cg11994851 | chr11 | 94,767,901 | 94,767,903 | *AMOTL1* | -0.087 | (0.009) | 4.6E-14 | -0.058 | 3.1E-14 |
| Abdomen-RT | cg02188223 | chr1 | 4,603,220 | 4,603,222 | *NA* | -0.131 | (0.014) | 4.9E-14 | -0.085 | 2.4E-13 |
| Abdomen-RT | cg09158878 | chr22 | 46,070,027 | 46,070,029 | *MIRLET7BHG;RP6-109B7.4* | -0.138 | (0.014) | 5.4E-14 | -0.089 | 3.2E-13 |
| Abdomen-RT | cg03026982 | chr11 | 19,932,152 | 19,932,154 | *NAV2* | -0.125 | (0.013) | 1.0E-13 | -0.087 | 7.0E-15 |
| Abdomen-RT | cg26189283 | chr1 | 155,136,901 | 155,136,903 | *SLC50A1* | -0.125 | (0.013) | 1.1E-13 | -0.082 | 2.3E-13 |
| Abdomen-RT | cg13442016 | chr10 | 102,436,581 | 102,436,583 | *MIR146B* | -0.137 | (0.014) | 1.6E-13 | -0.095 | 1.0E-14 |
| Abdomen-RT | cg08178756 | chr22 | 46,070,024 | 46,070,026 | *MIRLET7BHG;RP6-109B7.4* | -0.155 | (0.016) | 1.7E-13 | -0.102 | 3.6E-13 |
| Abdomen-RT | cg18598117 | chr19 | 941,125 | 941,127 | *ARID3A* | -0.141 | (0.015) | 1.8E-13 | -0.092 | 5.8E-13 |
| Abdomen-RT | cg03065175 | chr4 | 154,740,605 | 154,740,607 | *LRAT* | 0.140 | (0.015) | 2.2E-13 | 0.087 | 6.1E-12 |
| Abdomen-RT | cg05331731 | chr18 | 11,147,146 | 11,147,148 | *PIEZO2* | 0.208 | (0.022) | 2.5E-13 | 0.138 | 3.5E-13 |
| Abdomen-RT | cg06728103 | chr12 | 93,536,922 | 93,536,924 | *NA* | -0.106 | (0.011) | 3.2E-13 | -0.069 | 1.2E-12 |
| Abdomen-RT | cg24803517 | chr5 | 95,285,812 | 95,285,814 | *MCTP1* | 0.128 | (0.014) | 3.3E-13 | 0.078 | 3.4E-11 |
| Abdomen-RT | cg00218103 | chr22 | 46,070,096 | 46,070,098 | *MIRLET7BHG;RP6-109B7.4* | -0.121 | (0.013) | 4.8E-13 | -0.079 | 9.6E-13 |
| Abdomen-RT | cg23839200 | chr14 | 51,072,092 | 51,072,094 | *TRIM9* | -0.094 | (0.010) | 6.4E-13 | -0.062 | 1.2E-12 |
| Abdomen-RT | cg06576867 | chr22 | 46,070,030 | 46,070,032 | *MIRLET7BHG;RP6-109B7.4* | -0.128 | (0.014) | 8.7E-13 | -0.083 | 2.5E-12 |
| Abdomen-RT | cg02001279 | chr19 | 940,966 | 940,968 | *ARID3A* | -0.097 | (0.011) | 8.9E-13 | -0.064 | 9.2E-13 |
| Abdomen-RT | cg12960124 | chr4 | 82,100,021 | 82,100,023 | *NA* | -0.140 | (0.015) | 8.9E-13 | -0.097 | 7.8E-14 |
| Abdomen-RT | cg05135521 | chr2 | 160,331,823 | 160,331,825 | *RBMS1* | -0.127 | (0.014) | 9.2E-13 | -0.091 | 1.5E-14 |
| Abdomen-RT | cg09978533 | chr22 | 46,069,279 | 46,069,281 | *MIRLET7BHG;RP6-109B7.4* | -0.099 | (0.011) | 9.6E-13 | -0.069 | 7.5E-14 |
| Abdomen-RT | cg10570484 | chr1 | 158,038,015 | 158,038,017 | *KIRREL1* | -0.077 | (0.008) | 1.2E-12 | -0.050 | 4.2E-12 |
| Abdomen-RT | cg14122922 | chr20 | 44,714,873 | 44,714,875 | *KCNK15-AS1;WISP2* | -0.118 | (0.013) | 1.3E-12 | -0.084 | 2.0E-14 |
| Abdomen-RT | cg12634306 | chr1 | 39,633,138 | 39,633,140 | *HEYL;RP1-144F13.3* | 0.143 | (0.016) | 1.4E-12 | 0.089 | 3.3E-11 |
| Abdomen-RT | cg08101174 | chr10 | 102,436,783 | 102,436,785 | *NA* | -0.104 | (0.011) | 1.5E-12 | -0.075 | 1.4E-14 |
| Abdomen-RT | cg13286857 | chr5 | 66,822,120 | 66,822,122 | *MAST4* | -0.092 | (0.010) | 1.5E-12 | -0.058 | 1.5E-11 |
| Abdomen-RT | cg06535121 | chr4 | 26,088,488 | 26,088,490 | *NA* | -0.091 | (0.010) | 2.2E-12 | -0.060 | 5.9E-12 |
| Abdomen-RT | cg18147181 | chr12 | 52,244,373 | 52,244,375 | *KRT7* | -0.103 | (0.011) | 2.3E-12 | -0.074 | 4.1E-14 |
| Abdomen-RT | cg24833462 | chr2 | 108,053,726 | 108,053,728 | *AC023672.2* | -0.220 | (0.025) | 2.5E-12 | -0.142 | 9.7E-12 |
| Abdomen-RT | cg24769355 | chr9 | 72,041,875 | 72,041,877 | *NA* | -0.154 | (0.017) | 2.6E-12 | -0.100 | 8.3E-12 |
| Abdomen-RT | cg07861288 | chr13 | 91,719,216 | 91,719,218 | *GPC5* | -0.092 | (0.010) | 2.8E-12 | -0.061 | 3.3E-12 |
| Abdomen-RT | cg10334733 | chr12 | 93,541,835 | 93,541,837 | *NA* | -0.113 | (0.013) | 3.2E-12 | -0.079 | 2.7E-13 |
| Abdomen-RT | cg14263063 | chr16 | 21,150,859 | 21,150,861 | *DNAH3* | -0.148 | (0.017) | 3.4E-12 | -0.099 | 2.9E-12 |
| Abdomen-RT | cg10541181 | chr12 | 23,559,658 | 23,559,660 | *SOX5* | -0.183 | (0.021) | 3.5E-12 | -0.121 | 6.0E-12 |
| Abdomen-RT | cg13866747 | chr13 | 91,313,553 | 91,313,555 | *NA* | -0.176 | (0.020) | 4.2E-12 | -0.111 | 4.9E-11 |
| Abdomen-RT | cg22156456 | chr17 | 41,687,986 | 41,687,988 | *EIF1* | -0.088 | (0.010) | 4.8E-12 | -0.056 | 6.3E-11 |
| Abdomen-RT | cg15251748 | chr7 | 28,953,847 | 28,953,849 | *TRIL* | -0.154 | (0.017) | 5.0E-12 | -0.095 | 1.6E-10 |
| Abdomen-RT | cg25896883 | chr2 | 189,407,943 | 189,407,945 | *NA* | -0.145 | (0.016) | 5.1E-12 | -0.097 | 3.0E-12 |
| Abdomen-RT | cg16902294 | chr4 | 154,740,779 | 154,740,781 | *LRAT* | 0.120 | (0.014) | 6.4E-12 | 0.078 | 1.9E-11 |
| Abdomen-RT | cg06586734 | chr4 | 61,143,607 | 61,143,609 | *RP11-16N2.1* | -0.139 | (0.016) | 7.6E-12 | -0.094 | 2.4E-12 |
| Abdomen-RT | cg17232357 | chr15 | 66,720,493 | 66,720,495 | *SMAD6* | -0.119 | (0.014) | 8.8E-12 | -0.075 | 8.8E-11 |
| Abdomen-RT | cg16146033 | chr11 | 62,999,850 | 62,999,852 | *SLC22A8* | -0.114 | (0.013) | 8.8E-12 | -0.069 | 4.5E-10 |
| Abdomen-RT | cg19805775 | chr7 | 82,534,800 | 82,534,802 | *NA* | -0.124 | (0.014) | 9.8E-12 | -0.085 | 2.6E-12 |
| Abdomen-RT | cg10755723 | chr3 | 192,859,174 | 192,859,176 | *MB21D2* | 0.070 | (0.008) | 1.0E-11 | 0.047 | 9.0E-12 |
| Abdomen-RT | cg02453046 | chr21 | 15,837,853 | 15,837,855 | *USP25* | -0.094 | (0.011) | 1.1E-11 | -0.062 | 2.0E-11 |
| Abdomen-RT | cg13552692 | chr18 | 68,722,209 | 68,722,211 | *CCDC102B* | -0.125 | (0.014) | 1.1E-11 | -0.086 | 1.3E-12 |
| Abdomen-RT | cg25790232 | chr16 | 58,215,272 | 58,215,274 | *RP11-459F6.1* | -0.132 | (0.015) | 1.2E-11 | -0.083 | 1.2E-10 |
| Abdomen-RT | cg03864568 | chr9 | 76,400,794 | 76,400,796 | *NA* | -0.142 | (0.016) | 1.4E-11 | -0.098 | 2.0E-12 |
| Abdomen-RT | cg17407859 | chr3 | 165,259,995 | 165,259,997 | *LINC01322* | -0.186 | (0.021) | 1.4E-11 | -0.127 | 3.3E-12 |
| Abdomen-RT | cg14143795 | chr16 | 21,150,833 | 21,150,835 | *DNAH3* | -0.156 | (0.018) | 1.5E-11 | -0.102 | 2.7E-11 |
| Abdomen-RT | cg15857661 | chr10 | 102,436,485 | 102,436,487 | *MIR146B* | -0.122 | (0.014) | 1.5E-11 | -0.087 | 4.0E-13 |
| Abdomen-RT | cg12202498 | chr3 | 15,441,097 | 15,441,099 | *EAF1;EAF1-AS1;METTL6* | -0.077 | (0.009) | 1.5E-11 | -0.051 | 1.5E-11 |
| Abdomen-RT | cg04188877 | chr22 | 42,721,943 | 42,721,945 | *A4GALT* | 0.091 | (0.010) | 1.6E-11 | 0.061 | 8.6E-12 |
| Abdomen-RT | cg11414254 | chr5 | 177,022,010 | 177,022,012 | *UIMC1;ZNF346* | -0.124 | (0.014) | 1.9E-11 | -0.085 | 3.8E-12 |
| Abdomen-RT | cg01446044 | chr1 | 13,624,516 | 13,624,518 | *AL359771.1;RNA5SP41* | -0.067 | (0.008) | 1.9E-11 | -0.042 | 1.5E-10 |
| Abdomen-RT | cg13910395 | chr22 | 43,577,140 | 43,577,142 | *EFCAB6* | -0.098 | (0.011) | 2.0E-11 | -0.068 | 2.4E-12 |
| Abdomen-RT | cg00062245 | chr17 | 44,384,891 | 44,384,893 | *ITGA2B* | 0.106 | (0.012) | 2.2E-11 | 0.069 | 6.1E-11 |
| Abdomen-RT | cg08774778 | chr5 | 62,082,559 | 62,082,561 | *NA* | -0.122 | (0.014) | 2.2E-11 | -0.089 | 1.9E-13 |
| Abdomen-RT | cg24914621 | chr5 | 146,825,185 | 146,825,187 | *PPP2R2B* | 0.125 | (0.015) | 2.4E-11 | 0.080 | 8.3E-11 |
| Abdomen-RT | cg25325512 | chr6 | 37,174,443 | 37,174,445 | *PIM1* | -0.129 | (0.015) | 2.4E-11 | -0.082 | 2.2E-10 |
| Abdomen-RT | cg16060790 | chr9 | 101,372,935 | 101,372,937 | *BAAT* | -0.154 | (0.018) | 2.5E-11 | -0.106 | 3.7E-12 |
| Abdomen-RT | cg05657694 | chr3 | 100,635,045 | 100,635,047 | *ADGRG7* | -0.162 | (0.019) | 2.5E-11 | -0.107 | 2.7E-11 |
| Abdomen-RT | cg18419358 | chr6 | 157,962,976 | 157,962,978 | *NA* | -0.159 | (0.019) | 2.5E-11 | -0.103 | 7.8E-11 |
| Abdomen-RT | cg13903421 | chr2 | 218,873,991 | 218,873,993 | *WNT6* | 0.223 | (0.026) | 2.6E-11 | 0.152 | 8.6E-12 |
| Abdomen-RT | cg20517202 | chr16 | 21,150,520 | 21,150,522 | *DNAH3* | -0.143 | (0.017) | 2.7E-11 | -0.090 | 2.9E-10 |
| Abdomen-RT | cg02699612 | chr10 | 113,753,520 | 113,753,522 | *PLEKHS1* | -0.183 | (0.021) | 3.1E-11 | -0.128 | 2.4E-12 |
| Abdomen-RT | cg11963452 | chr2 | 28,394,684 | 28,394,686 | *FLJ31356;FOSL2* | -0.112 | (0.013) | 3.9E-11 | -0.080 | 1.3E-12 |
| Abdomen-RT | cg09539785 | chr22 | 24,729,643 | 24,729,645 | *PIWIL3* | -0.106 | (0.013) | 3.9E-11 | -0.072 | 1.9E-11 |
| Abdomen-RT | cg05712639 | chr14 | 52,352,667 | 52,352,669 | *NA* | -0.198 | (0.023) | 4.8E-11 | -0.129 | 1.1E-10 |
| Abdomen-RT | cg13806740 | chr17 | 17,971,714 | 17,971,716 | *DRC3;TOM1L2* | -0.069 | (0.008) | 5.0E-11 | -0.047 | 1.5E-11 |
| Abdomen-RT | cg22976567 | chr1 | 156,104,390 | 156,104,392 | *LMNA* | -0.092 | (0.011) | 5.1E-11 | -0.063 | 1.5E-11 |
| Abdomen-RT | cg21752134 | chr16 | 79,640,594 | 79,640,596 | *NA* | -0.084 | (0.010) | 5.4E-11 | -0.058 | 8.7E-12 |
| Abdomen-RT | cg17109563 | chr6 | 125,363,279 | 125,363,281 | *NA* | -0.129 | (0.015) | 5.8E-11 | -0.080 | 9.0E-10 |
| Abdomen-RT | cg08119153 | chr14 | 78,446,957 | 78,446,959 | *NRXN3* | 0.146 | (0.017) | 6.1E-11 | 0.094 | 2.3E-10 |
| Abdomen-RT | cg17445936 | chr1 | 3,173,780 | 3,173,782 | *PRDM16* | 0.191 | (0.023) | 6.3E-11 | 0.115 | 3.9E-09 |
| Abdomen-RT | cg12713583 | chr19 | 940,723 | 940,725 | *ARID3A* | -0.164 | (0.020) | 6.5E-11 | -0.103 | 6.6E-10 |
| Abdomen-RT | cg20112376 | chr4 | 6,116,715 | 6,116,717 | *JAKMIP1* | -0.103 | (0.012) | 6.6E-11 | -0.070 | 2.5E-11 |
| Abdomen-RT | cg05478824 | chr17 | 82,012,258 | 82,012,260 | *ASPSCR1* | 0.097 | (0.012) | 6.7E-11 | 0.062 | 2.9E-10 |
| Abdomen-RT | cg07501635 | chr3 | 53,680,140 | 53,680,142 | *CACNA1D* | 0.221 | (0.027) | 8.0E-11 | 0.142 | 3.0E-10 |
| Abdomen-RT | cg21359950 | chr12 | 93,689,693 | 93,689,695 | *CRADD* | -0.155 | (0.019) | 8.2E-11 | -0.102 | 1.2E-10 |
| Abdomen-RT | cg26663696 | chr3 | 160,403,486 | 160,403,488 | *MIR15B;MIR16-2;RP11-432B6.3;SMC4* | -0.156 | (0.019) | 8.5E-11 | -0.103 | 8.9E-11 |
| Abdomen-RT | cg19080839 | chr7 | 151,242,505 | 151,242,507 | *SMARCD3* | 0.097 | (0.012) | 9.3E-11 | 0.064 | 1.3E-10 |
| Abdomen-RT | cg00589617 | chr1 | 230,279,596 | 230,279,598 | *GALNT2* | 0.160 | (0.019) | 9.6E-11 | 0.100 | 1.2E-09 |
| Abdomen-RT | cg05944249 | chr8 | 27,636,723 | 27,636,725 | *SCARA3* | 0.154 | (0.019) | 1.0E-10 | 0.106 | 1.9E-11 |
| Abdomen-RT | cg09835575 | chr20 | 9,985,215 | 9,985,217 | *ANKEF1;RP5-839B4.8* | -0.131 | (0.016) | 1.0E-10 | -0.087 | 7.8E-11 |
| Abdomen-RT | cg05186879 | chr3 | 50,612,890 | 50,612,892 | *CISH;MAPKAPK3* | -0.076 | (0.009) | 1.2E-10 | -0.049 | 4.8E-10 |
| Abdomen-RT | cg14200569 | chr1 | 3,135,360 | 3,135,362 | *PRDM16* | 0.159 | (0.019) | 1.3E-10 | 0.099 | 1.8E-09 |
| Abdomen-RT | cg15246238 | chr7 | 5,595,502 | 5,595,504 | *FSCN1* | -0.106 | (0.013) | 1.5E-10 | -0.066 | 1.7E-09 |
| Abdomen-RT | cg13329407 | chr21 | 24,429,059 | 24,429,061 | *AP000476.1* | -0.207 | (0.025) | 1.8E-10 | -0.130 | 2.0E-09 |
| Abdomen-RT | cg04697265 | chr11 | 130,813,804 | 130,813,806 | *NA* | -0.091 | (0.011) | 1.9E-10 | -0.060 | 2.7E-10 |
| Abdomen-RT | cg12051710 | chr12 | 2,879,903 | 2,879,905 | *RHNO1;TULP3* | -0.108 | (0.013) | 2.0E-10 | -0.072 | 2.0E-10 |
| Abdomen-RT | cg22234488 | chr2 | 105,179,803 | 105,179,805 | *NA* | -0.111 | (0.014) | 2.0E-10 | -0.076 | 6.1E-11 |
| Abdomen-RT | cg25241559 | chr2 | 241,049,961 | 241,049,963 | *AC005237.4;SNED1* | 0.075 | (0.009) | 2.3E-10 | 0.051 | 7.1E-11 |
| Abdomen-RT | cg19434960 | chr21 | 23,922,356 | 23,922,358 | *NA* | -0.104 | (0.013) | 2.5E-10 | -0.068 | 6.3E-10 |
| Abdomen-RT | cg21385305 | chr2 | 23,490,082 | 23,490,084 | *KLHL29* | -0.113 | (0.014) | 2.7E-10 | -0.070 | 3.4E-09 |
| Abdomen-RT | cg00117536 | chr7 | 48,088,526 | 48,088,528 | *UPP1* | -0.122 | (0.015) | 2.7E-10 | -0.085 | 3.4E-11 |
| Abdomen-RT | cg17783244 | chr22 | 31,342,562 | 31,342,564 | *PATZ1* | 0.101 | (0.013) | 3.2E-10 | 0.066 | 7.7E-10 |
| Abdomen-RT | cg07097098 | chr19 | 55,210,269 | 55,210,271 | *PTPRH* | -0.094 | (0.012) | 3.2E-10 | -0.062 | 3.8E-10 |
| Abdomen-RT | cg05848650 | chr3 | 192,893,050 | 192,893,052 | *MB21D2* | -0.160 | (0.020) | 3.5E-10 | -0.113 | 2.3E-11 |
| Abdomen-RT | cg19152802 | chr5 | 110,514,185 | 110,514,187 | *MIR548F3;TMEM232* | -0.129 | (0.016) | 3.8E-10 | -0.090 | 5.1E-11 |
| Abdomen-RT | cg15871086 | chr18 | 58,859,362 | 58,859,364 | *NA* | -0.096 | (0.012) | 4.2E-10 | -0.064 | 5.0E-10 |
| Abdomen-RT | cg15890546 | chr3 | 194,967,758 | 194,967,760 | *NA* | -0.091 | (0.011) | 4.3E-10 | -0.059 | 1.0E-09 |
| Abdomen-RT | cg26408927 | chr3 | 53,725,695 | 53,725,697 | *CACNA1D* | -0.159 | (0.020) | 4.7E-10 | -0.103 | 1.1E-09 |
| Abdomen-RT | cg22639195 | chr17 | 19,370,112 | 19,370,114 | *B9D1* | -0.086 | (0.011) | 4.9E-10 | -0.063 | 3.8E-12 |
| Abdomen-RT | cg21781784 | chr1 | 227,437,757 | 227,437,759 | *NA* | -0.140 | (0.018) | 4.9E-10 | -0.096 | 1.2E-10 |
| Abdomen-RT | cg14879089 | chr4 | 5,827,008 | 5,827,010 | *CRMP1* | -0.122 | (0.015) | 5.1E-10 | -0.081 | 5.1E-10 |
| Abdomen-RT | cg01530498 | chr22 | 27,475,264 | 27,475,266 | *NA* | -0.093 | (0.012) | 5.1E-10 | -0.065 | 9.4E-11 |
| Abdomen-RT | cg18661060 | chr10 | 71,758,477 | 71,758,479 | *CDH23;VSIR* | -0.078 | (0.010) | 5.7E-10 | -0.051 | 8.2E-10 |
| Abdomen-RT | cg10473623 | chr11 | 20,097,629 | 20,097,631 | *NAV2* | -0.113 | (0.014) | 5.7E-10 | -0.075 | 5.1E-10 |
| Abdomen-RT | cg16198075 | chr4 | 6,417,112 | 6,417,114 | *PPP2R2C* | 0.126 | (0.016) | 5.8E-10 | 0.081 | 2.3E-09 |
| Abdomen-RT | cg12307404 | chr3 | 186,552,703 | 186,552,705 | *TBCCD1* | -0.076 | (0.010) | 5.9E-10 | -0.046 | 1.1E-08 |
| Abdomen-RT | cg05697274 | chr1 | 230,279,630 | 230,279,632 | *GALNT2* | 0.128 | (0.016) | 6.2E-10 | 0.081 | 3.4E-09 |
| Abdomen-RT | cg03479114 | chr4 | 165,359,572 | 165,359,574 | *NA* | -0.119 | (0.015) | 6.5E-10 | -0.084 | 6.9E-11 |
| Abdomen-RT | cg21025494 | chr7 | 23,463,124 | 23,463,126 | *IGF2BP3* | -0.083 | (0.010) | 7.1E-10 | -0.057 | 1.6E-10 |
| Abdomen-RT | cg03054277 | chr1 | 228,212,515 | 228,212,517 | *OBSCN;OBSCN-AS1* | 0.085 | (0.011) | 7.1E-10 | 0.054 | 3.3E-09 |
| Abdomen-RT | cg10762064 | chr3 | 194,967,685 | 194,967,687 | *NA* | -0.102 | (0.013) | 7.2E-10 | -0.068 | 6.9E-10 |
| Abdomen-RT | cg00135497 | chr4 | 154,740,696 | 154,740,698 | *LRAT* | 0.083 | (0.010) | 7.6E-10 | 0.050 | 2.8E-08 |
| Abdomen-RT | cg08573679 | chr6 | 125,363,363 | 125,363,365 | *NA* | -0.127 | (0.016) | 7.7E-10 | -0.081 | 3.3E-09 |
| Abdomen-RT | cg18462381 | chr10 | 127,738,453 | 127,738,455 | *FOXI2;RP11-288A5.2* | 0.126 | (0.016) | 7.8E-10 | 0.083 | 1.1E-09 |
| Abdomen-RT | cg08173263 | chr19 | 14,166,098 | 14,166,100 | *ADGRL1;CTB-55O6.12* | 0.157 | (0.020) | 7.8E-10 | 0.098 | 8.0E-09 |
| Abdomen-RT | cg08662753 | chr9 | 134,669,653 | 134,669,655 | *COL5A1* | 0.099 | (0.013) | 8.1E-10 | 0.058 | 6.0E-08 |
| Abdomen-RT | cg19283806 | chr18 | 68,722,182 | 68,722,184 | *CCDC102B* | -0.088 | (0.011) | 8.1E-10 | -0.058 | 8.7E-10 |
| Abdomen-RT | cg23522611 | chr2 | 28,394,744 | 28,394,746 | *FLJ31356;FOSL2* | -0.067 | (0.009) | 8.5E-10 | -0.048 | 2.7E-11 |
| Abdomen-RT | cg08587685 | chr10 | 114,632,446 | 114,632,448 | *ABLIM1* | -0.060 | (0.008) | 8.5E-10 | -0.041 | 3.7E-10 |
| Abdomen-RT | cg13935634 | chr6 | 10,583,535 | 10,583,537 | *GCNT2* | -0.107 | (0.014) | 8.7E-10 | -0.068 | 3.5E-09 |
| Abdomen-RT | cg21443659 | chr2 | 216,880,997 | 216,880,999 | *AC007563.5* | -0.122 | (0.016) | 9.0E-10 | -0.081 | 8.4E-10 |
| Abdomen-RT | cg25695450 | chr7 | 41,219,438 | 41,219,440 | *NA* | -0.105 | (0.013) | 9.3E-10 | -0.075 | 4.5E-11 |
| Abdomen-RT | cg12597694 | chr16 | 8,183,208 | 8,183,210 | *NA* | -0.157 | (0.020) | 1.0E-09 | -0.096 | 2.5E-08 |
| Abdomen-RT | cg22948745 | chr8 | 138,688,051 | 138,688,053 | *COL22A1* | -0.126 | (0.016) | 1.0E-09 | -0.079 | 9.0E-09 |
| Abdomen-RT | cg03957124 | chr6 | 37,049,092 | 37,049,094 | *NA* | -0.075 | (0.010) | 1.1E-09 | -0.048 | 5.4E-09 |
| Abdomen-RT | cg03664096 | chr21 | 43,197,746 | 43,197,748 | *NA* | -0.097 | (0.012) | 1.2E-09 | -0.063 | 3.6E-09 |
| Abdomen-RT | cg15491247 | chr1 | 3,313,859 | 3,313,861 | *PRDM16* | -0.093 | (0.012) | 1.2E-09 | -0.059 | 5.8E-09 |
| Abdomen-RT | cg01004017 | chr2 | 120,899,770 | 120,899,772 | *GLI2* | 0.126 | (0.016) | 1.4E-09 | 0.082 | 3.2E-09 |
| Abdomen-RT | cg06007201 | chr16 | 88,783,809 | 88,783,811 | *PIEZO1* | -0.111 | (0.014) | 1.4E-09 | -0.076 | 6.0E-10 |
| Abdomen-RT | cg11494773 | chr7 | 48,088,644 | 48,088,646 | *UPP1* | -0.125 | (0.016) | 1.4E-09 | -0.089 | 7.0E-11 |
| Abdomen-RT | cg26936966 | chr8 | 135,599,881 | 135,599,883 | *KHDRBS3* | 0.078 | (0.010) | 1.4E-09 | 0.050 | 5.8E-09 |
| Abdomen-RT | cg06855546 | chr16 | 1,488,824 | 1,488,826 | *PTX4* | 0.077 | (0.010) | 1.5E-09 | 0.050 | 3.0E-09 |
| Abdomen-RT | cg00008629 | chr9 | 112,331,380 | 112,331,382 | *PTBP3* | -0.159 | (0.021) | 1.5E-09 | -0.103 | 3.7E-09 |
| Abdomen-RT | cg12530994 | chr10 | 5,094,589 | 5,094,591 | *AKR1C1;AKR1C2;AKR1C3;SNORD118* | -0.124 | (0.016) | 1.5E-09 | -0.077 | 1.3E-08 |
| Abdomen-RT | cg07265924 | chr8 | 60,967,910 | 60,967,912 | *AC022182.3* | -0.191 | (0.025) | 1.5E-09 | -0.129 | 7.9E-10 |
| Abdomen-RT | cg15179400 | chr1 | 15,343,955 | 15,343,957 | *FHAD1;RP3-467K16.2* | -0.095 | (0.012) | 1.6E-09 | -0.068 | 7.5E-11 |
| Abdomen-RT | cg02757819 | chr3 | 172,581,715 | 172,581,717 | *RP11-408H1.3* | -0.081 | (0.010) | 1.9E-09 | -0.053 | 2.8E-09 |
| Abdomen-RT | cg09519959 | chr17 | 28,643,897 | 28,643,899 | *KIAA0100* | -0.090 | (0.012) | 2.1E-09 | -0.061 | 1.4E-09 |
| Abdomen-RT | cg08196217 | chr8 | 106,083,464 | 106,083,466 | *NA* | -0.131 | (0.017) | 2.1E-09 | -0.083 | 9.4E-09 |
| Abdomen-RT | cg02045948 | chr3 | 32,815,824 | 32,815,826 | *NA* | -0.093 | (0.012) | 2.1E-09 | -0.059 | 1.2E-08 |
| Abdomen-RT | cg26910511 | chr19 | 39,308,396 | 39,308,398 | *LRFN1* | 0.088 | (0.012) | 2.5E-09 | 0.052 | 1.1E-07 |
| Abdomen-RT | cg21242613 | chr1 | 15,344,049 | 15,344,051 | *FHAD1;RP3-467K16.2* | -0.077 | (0.010) | 2.8E-09 | -0.049 | 1.4E-08 |
| Abdomen-RT | cg08909363 | chr11 | 72,181,494 | 72,181,496 | *RP11-807H22.7* | 0.067 | (0.009) | 3.0E-09 | 0.042 | 1.9E-08 |
| Abdomen-RT | cg01464849 | chr3 | 160,402,692 | 160,402,694 | *RP11-432B6.3;SMC4* | -0.147 | (0.019) | 3.1E-09 | -0.099 | 1.7E-09 |
| Abdomen-RT | cg12569593 | chr11 | 126,543,820 | 126,543,822 | *KIRREL3;KIRREL3-AS1* | -0.066 | (0.009) | 3.1E-09 | -0.044 | 2.6E-09 |
| Abdomen-RT | cg07545728 | chr10 | 80,501,100 | 80,501,102 | *TSPAN14* | -0.109 | (0.014) | 3.1E-09 | -0.070 | 1.2E-08 |
| Abdomen-RT | cg04882213 | chr8 | 60,967,832 | 60,967,834 | *AC022182.3* | -0.132 | (0.017) | 3.2E-09 | -0.091 | 7.5E-10 |
| Abdomen-RT | cg25719685 | chr3 | 100,635,047 | 100,635,049 | *ADGRG7* | -0.135 | (0.018) | 3.3E-09 | -0.092 | 1.4E-09 |
| Abdomen-RT | cg25788793 | chr4 | 10,032,955 | 10,032,957 | *SLC2A9* | -0.082 | (0.011) | 3.3E-09 | -0.058 | 2.7E-10 |
| Abdomen-RT | cg20598190 | chr2 | 239,927,506 | 239,927,508 | *NDUFA10* | -0.199 | (0.026) | 3.4E-09 | -0.132 | 3.3E-09 |
| Abdomen-RT | cg08066673 | chr14 | 51,859,028 | 51,859,030 | *GNG2* | -0.077 | (0.010) | 3.4E-09 | -0.051 | 3.7E-09 |
| Abdomen-RT | cg21922478 | chr5 | 52,945,259 | 52,945,261 | *CTD-2175A23.1;ITGA1* | -0.142 | (0.019) | 3.5E-09 | -0.095 | 2.4E-09 |
| Abdomen-RT | cg08404702 | chr16 | 2,083,386 | 2,083,388 | *TSC2* | 0.074 | (0.010) | 3.6E-09 | 0.049 | 5.5E-09 |
| Abdomen-RT | cg02541477 | chr13 | 52,061,901 | 52,061,903 | *NEK5* | -0.168 | (0.022) | 3.6E-09 | -0.107 | 1.5E-08 |
| Abdomen-RT | cg03670162 | chr12 | 107,903,561 | 107,903,563 | *RP11-554D14.2* | -0.107 | (0.014) | 3.7E-09 | -0.070 | 4.6E-09 |
| Abdomen-RT | cg05483571 | chr17 | 79,927,424 | 79,927,426 | *RP11-353N14.5* | -0.117 | (0.015) | 3.8E-09 | -0.078 | 3.9E-09 |
| Abdomen-RT | cg17061862 | chr11 | 9,568,883 | 9,568,885 | *NA* | -0.101 | (0.013) | 3.8E-09 | -0.062 | 4.2E-08 |
| Abdomen-RT | cg16394551 | chr1 | 3,313,792 | 3,313,794 | *PRDM16* | -0.098 | (0.013) | 4.2E-09 | -0.061 | 3.2E-08 |
| Abdomen-RT | cg14395060 | chr22 | 32,045,182 | 32,045,184 | *SLC5A1* | -0.112 | (0.015) | 4.2E-09 | -0.082 | 7.5E-11 |
| Abdomen-RT | cg12022558 | chr2 | 205,245,819 | 205,245,821 | *PARD3B* | -0.144 | (0.019) | 4.7E-09 | -0.093 | 1.2E-08 |
| Abdomen-RT | cg02148547 | chr14 | 20,989,342 | 20,989,344 | *METTL17* | -0.082 | (0.011) | 5.1E-09 | -0.052 | 3.5E-08 |
| Abdomen-RT | cg26216433 | chr14 | 103,880,253 | 103,880,255 | *CTD-2134A5.4* | -0.107 | (0.014) | 5.1E-09 | -0.071 | 4.2E-09 |
| Abdomen-RT | cg05251190 | chr10 | 102,436,448 | 102,436,450 | *MIR146B* | -0.072 | (0.010) | 5.3E-09 | -0.052 | 2.2E-10 |
| Abdomen-RT | cg09317107 | chr1 | 56,788,784 | 56,788,786 | *FYB2* | -0.071 | (0.010) | 5.3E-09 | -0.048 | 3.8E-09 |
| Abdomen-RT | cg02606535 | chr16 | 2,083,030 | 2,083,032 | *TSC2* | 0.065 | (0.009) | 5.7E-09 | 0.041 | 2.6E-08 |
| Abdomen-RT | cg19713833 | chr13 | 106,600,452 | 106,600,454 | *NA* | -0.139 | (0.019) | 5.7E-09 | -0.092 | 7.1E-09 |
| Abdomen-RT | cg14322760 | chr3 | 160,402,675 | 160,402,677 | *RP11-432B6.3;SMC4* | -0.142 | (0.019) | 5.8E-09 | -0.094 | 6.1E-09 |
| Abdomen-RT | cg21393163 | chr1 | 12,157,571 | 12,157,573 | *NA* | -0.121 | (0.016) | 6.0E-09 | -0.084 | 1.2E-09 |
| Abdomen-RT | cg23135545 | chr15 | 101,024,297 | 101,024,299 | *LRRK1* | 0.053 | (0.007) | 6.0E-09 | 0.032 | 2.0E-07 |
| Abdomen-RT | cg14040894 | chr4 | 188,781,846 | 188,781,848 | *RP11-756P10.2;RP11-756P10.5* | -0.087 | (0.012) | 6.7E-09 | -0.056 | 1.8E-08 |
| Abdomen-RT | cg09466904 | chr2 | 26,969,319 | 26,969,321 | *AC013472.4;MAPRE3* | -0.131 | (0.018) | 6.8E-09 | -0.085 | 1.8E-08 |
| Abdomen-RT | cg06269255 | chr17 | 44,269,426 | 44,269,428 | *SLC4A1* | -0.066 | (0.009) | 6.9E-09 | -0.040 | 1.3E-07 |
| Abdomen-RT | cg01750375 | chr20 | 44,714,723 | 44,714,725 | *KCNK15-AS1;WISP2* | -0.079 | (0.011) | 7.1E-09 | -0.053 | 3.1E-09 |
| Abdomen-RT | cg07363855 | chr1 | 3,067,905 | 3,067,907 | *LINC00982;PRDM16* | 0.073 | (0.010) | 7.1E-09 | 0.047 | 1.6E-08 |
| Abdomen-RT | cg04433322 | chr17 | 44,269,525 | 44,269,527 | *SLC4A1* | -0.059 | (0.008) | 7.2E-09 | -0.040 | 5.4E-09 |
| Abdomen-RT | cg25371036 | chr11 | 94,767,582 | 94,767,584 | *AMOTL1* | -0.073 | (0.010) | 7.4E-09 | -0.051 | 9.0E-10 |
| Abdomen-RT | cg16548911 | chr16 | 50,313,854 | 50,313,856 | *ADCY7;BRD7* | 0.104 | (0.014) | 7.6E-09 | 0.069 | 7.5E-09 |
| Abdomen-RT | cg11093223 | chr9 | 34,363,683 | 34,363,685 | *NA* | 0.057 | (0.008) | 7.6E-09 | 0.039 | 2.6E-09 |
| Abdomen-RT | cg10202782 | chr9 | 101,372,961 | 101,372,963 | *BAAT* | -0.120 | (0.016) | 7.8E-09 | -0.081 | 5.7E-09 |
| Abdomen-RT | cg01360622 | chr17 | 42,311,406 | 42,311,408 | *STAT5A* | 0.070 | (0.009) | 8.0E-09 | 0.046 | 1.3E-08 |
| Abdomen-RT | cg23468816 | chr10 | 62,048,988 | 62,048,990 | *ARID5B* | -0.062 | (0.008) | 8.4E-09 | -0.044 | 7.4E-10 |
| Abdomen-RT | cg02000109 | chr20 | 38,357,665 | 38,357,667 | *LBP* | -0.130 | (0.018) | 8.5E-09 | -0.087 | 6.0E-09 |
| Abdomen-RT | cg11339839 | chr17 | 79,927,329 | 79,927,331 | *RP11-353N14.5* | -0.066 | (0.009) | 8.8E-09 | -0.043 | 1.1E-08 |
| Abdomen-RT | cg19567594 | chr11 | 10,693,627 | 10,693,629 | *MRVI1* | 0.096 | (0.013) | 9.0E-09 | 0.063 | 1.1E-08 |
| Abdomen-RT | cg01869007 | chr8 | 60,967,853 | 60,967,855 | *AC022182.3* | -0.158 | (0.021) | 9.1E-09 | -0.106 | 5.4E-09 |
| Abdomen-RT | cg24618605 | chr18 | 31,437,150 | 31,437,152 | *NA* | -0.075 | (0.010) | 9.5E-09 | -0.046 | 1.3E-07 |
| Abdomen-RT | cg17287921 | chr2 | 45,964,630 | 45,964,632 | *PRKCE* | -0.052 | (0.007) | 9.7E-09 | -0.035 | 7.5E-09 |
| Abdomen-RT | cg01022345 | chr14 | 100,851,481 | 100,851,483 | *MEG3;MIR770;RP11-123M6.2* | -0.067 | (0.009) | 9.9E-09 | -0.044 | 1.5E-08 |
| Abdomen-RT | cg09762316 | chr9 | 70,043,437 | 70,043,439 | *MAMDC2;MAMDC2-AS1* | 0.085 | (0.012) | 1.0E-08 | 0.052 | 1.1E-07 |
| Abdomen-RT | cg17516475 | chr22 | 46,044,588 | 46,044,590 | *LINC00899* | -0.111 | (0.015) | 1.1E-08 | -0.071 | 4.4E-08 |
| Abdomen-RT | cg16618104 | chr12 | 104,459,321 | 104,459,323 | *CHST11* | -0.118 | (0.016) | 1.1E-08 | -0.087 | 2.1E-10 |
| Abdomen-RT | cg01693697 | chr15 | 73,920,554 | 73,920,556 | *LOXL1-AS1* | -0.120 | (0.016) | 1.1E-08 | -0.078 | 2.4E-08 |
| Abdomen-RT | cg27136634 | chr11 | 17,414,582 | 17,414,584 | *ABCC8* | -0.101 | (0.014) | 1.2E-08 | -0.065 | 3.5E-08 |
| Abdomen-RT | cg03446675 | chr9 | 97,411,440 | 97,411,442 | *TDRD7* | -0.058 | (0.008) | 1.2E-08 | -0.037 | 4.2E-08 |
| Abdomen-RT | cg22385325 | chr11 | 66,326,605 | 66,326,607 | *NA* | -0.080 | (0.011) | 1.3E-08 | -0.052 | 3.7E-08 |
| Abdomen-RT | cg03984209 | chr3 | 55,885,989 | 55,885,991 | *ERC2* | -0.102 | (0.014) | 1.3E-08 | -0.067 | 2.0E-08 |
| Abdomen-RT | cg15572968 | chr20 | 2,525,549 | 2,525,551 | *ZNF343* | -0.060 | (0.008) | 1.3E-08 | -0.035 | 8.3E-07 |
| Abdomen-RT | cg16660220 | chr11 | 46,555,397 | 46,555,399 | *AMBRA1* | -0.085 | (0.012) | 1.3E-08 | -0.058 | 6.6E-09 |
| Abdomen-RT | cg26350754 | chr6 | 33,076,090 | 33,076,092 | *HLA-DPA1;HLA-DPB1* | -0.123 | (0.017) | 1.4E-08 | -0.072 | 4.9E-07 |
| Abdomen-RT | cg19075225 | chr2 | 241,070,097 | 241,070,099 | *SNED1* | 0.126 | (0.017) | 1.4E-08 | 0.078 | 1.3E-07 |
| Abdomen-RT | cg07784975 | chr12 | 23,360,735 | 23,360,737 | *NA* | -0.170 | (0.023) | 1.4E-08 | -0.118 | 3.2E-09 |
| Abdomen-RT | cg25175639 | chr19 | 53,866,149 | 53,866,151 | *AC008753.6;MYADM* | -0.166 | (0.023) | 1.4E-08 | -0.105 | 6.1E-08 |
| Abdomen-RT | cg04368942 | chr7 | 134,886,568 | 134,886,570 | *CALD1* | -0.085 | (0.012) | 1.5E-08 | -0.062 | 3.2E-10 |
| Abdomen-RT | cg13474360 | chr3 | 192,885,619 | 192,885,621 | *MB21D2* | 0.075 | (0.010) | 1.5E-08 | 0.050 | 1.2E-08 |
| Abdomen-RT | cg06130949 | chr7 | 48,088,412 | 48,088,414 | *UPP1* | -0.061 | (0.008) | 1.5E-08 | -0.040 | 1.7E-08 |
| Abdomen-RT | cg15393490 | chr1 | 207,823,113 | 207,823,115 | *MIR29B2CHG* | 0.094 | (0.013) | 1.5E-08 | 0.064 | 5.6E-09 |
| Abdomen-RT | cg25987136 | chr1 | 3,367,668 | 3,367,670 | *PRDM16* | -0.067 | (0.009) | 1.6E-08 | -0.042 | 6.1E-08 |
| Abdomen-RT | cg07323488 | chr3 | 168,467,524 | 168,467,526 | *EGFEM1P* | -0.084 | (0.012) | 1.7E-08 | -0.056 | 1.9E-08 |
| Abdomen-RT | cg06455616 | chr17 | 10,013,581 | 10,013,583 | *GAS7* | -0.110 | (0.015) | 1.7E-08 | -0.074 | 1.2E-08 |
| Abdomen-RT | cg05858126 | chr10 | 102,436,455 | 102,436,457 | *MIR146B* | -0.094 | (0.013) | 1.8E-08 | -0.070 | 2.2E-10 |
| Abdomen-RT | cg06009645 | chr6 | 111,714,895 | 111,714,897 | *FYN* | -0.070 | (0.010) | 1.8E-08 | -0.046 | 2.4E-08 |
| Abdomen-RT | cg27495424 | chr2 | 88,625,579 | 88,625,581 | *EIF2AK3* | -0.064 | (0.009) | 1.8E-08 | -0.046 | 1.7E-09 |
| Abdomen-RT | cg15862165 | chr12 | 93,767,339 | 93,767,341 | *CRADD* | -0.087 | (0.012) | 2.0E-08 | -0.060 | 8.3E-09 |
| Abdomen-RT | cg15444185 | chr8 | 66,542,471 | 66,542,473 | *NA* | 0.092 | (0.013) | 2.0E-08 | 0.059 | 6.0E-08 |
| Abdomen-RT | cg09326780 | chr3 | 192,843,493 | 192,843,495 | *MB21D2* | 0.065 | (0.009) | 2.0E-08 | 0.043 | 2.2E-08 |
| Abdomen-RT | cg01435315 | chr10 | 70,588,663 | 70,588,665 | *NA* | -0.117 | (0.016) | 2.0E-08 | -0.078 | 1.7E-08 |
| Abdomen-RT | cg19634849 | chr5 | 140,259,739 | 140,259,741 | *CYSTM1;PFDN1* | -0.070 | (0.010) | 2.0E-08 | -0.046 | 2.2E-08 |
| Abdomen-RT | cg17631924 | chr17 | 28,034,180 | 28,034,182 | *NA* | -0.074 | (0.010) | 2.2E-08 | -0.047 | 9.6E-08 |
| Abdomen-RT | cg00303773 | chr17 | 17,849,260 | 17,849,262 | *TOM1L2* | -0.161 | (0.022) | 2.3E-08 | -0.116 | 1.0E-09 |
| Abdomen-RT | cg06268875 | chr18 | 11,147,385 | 11,147,387 | *PIEZO2* | 0.120 | (0.017) | 2.3E-08 | 0.075 | 1.3E-07 |
| Abdomen-RT | cg12800962 | chr20 | 50,009,899 | 50,009,901 | *NA* | 0.101 | (0.014) | 2.3E-08 | 0.062 | 2.6E-07 |
| Abdomen-RT | cg18481613 | chr12 | 89,019,874 | 89,019,876 | *RP11-13A1.1* | -0.083 | (0.012) | 2.3E-08 | -0.052 | 9.7E-08 |
| Abdomen-RT | cg04313338 | chr16 | 55,701,881 | 55,701,883 | *SLC6A2* | -0.053 | (0.007) | 2.4E-08 | -0.031 | 6.5E-07 |
| Abdomen-RT | cg14436379 | chr3 | 122,245,755 | 122,245,757 | *CASR* | -0.128 | (0.018) | 2.6E-08 | -0.084 | 3.2E-08 |
| Abdomen-RT | cg04376114 | chr8 | 73,463,029 | 73,463,031 | *STAU2* | -0.067 | (0.009) | 2.6E-08 | -0.047 | 4.4E-09 |
| Abdomen-RT | cg13924715 | chr11 | 10,729,342 | 10,729,344 | *NA* | 0.177 | (0.025) | 2.7E-08 | 0.117 | 3.3E-08 |
| Abdomen-RT | cg05468212 | chr9 | 134,796,289 | 134,796,291 | *COL5A1* | 0.053 | (0.007) | 2.7E-08 | 0.034 | 8.1E-08 |
| Abdomen-RT | cg21411447 | chr9 | 16,262,654 | 16,262,656 | *C9orf92* | -0.100 | (0.014) | 2.7E-08 | -0.066 | 3.2E-08 |
| Abdomen-RT | cg03296370 | chr3 | 39,281,724 | 39,281,726 | *CX3CR1* | -0.052 | (0.007) | 2.7E-08 | -0.035 | 1.5E-08 |
| Abdomen-RT | cg15352568 | chr1 | 209,875,771 | 209,875,773 | *NA* | -0.085 | (0.012) | 2.8E-08 | -0.058 | 9.3E-09 |
| Abdomen-RT | cg08585946 | chr2 | 234,492,122 | 234,492,124 | *NA* | -0.064 | (0.009) | 3.2E-08 | -0.044 | 1.6E-08 |
| Abdomen-RT | cg27004870 | chr16 | 88,783,975 | 88,783,977 | *PIEZO1* | -0.089 | (0.013) | 3.3E-08 | -0.059 | 2.9E-08 |
| Abdomen-RT | cg02286081 | chr6 | 33,076,063 | 33,076,065 | *HLA-DPA1;HLA-DPB1* | -0.147 | (0.021) | 3.3E-08 | -0.088 | 5.6E-07 |
| Abdomen-RT | cg17107472 | chr3 | 50,574,267 | 50,574,269 | *HEMK1* | -0.047 | (0.007) | 3.3E-08 | -0.032 | 1.3E-08 |
| Abdomen-RT | cg22398226 | chr4 | 154,741,064 | 154,741,066 | *LRAT* | 0.080 | (0.011) | 3.4E-08 | 0.051 | 1.0E-07 |
| Abdomen-RT | cg25840926 | chr2 | 20,448,225 | 20,448,227 | *RHOB* | 0.112 | (0.016) | 3.4E-08 | 0.077 | 1.1E-08 |
| Abdomen-RT | cg21094405 | chr13 | 113,001,802 | 113,001,804 | *MCF2L* | -0.089 | (0.013) | 3.4E-08 | -0.062 | 6.4E-09 |
| Abdomen-RT | cg05211447 | chr14 | 103,880,242 | 103,880,244 | *CTD-2134A5.4* | -0.112 | (0.016) | 3.5E-08 | -0.073 | 5.6E-08 |
| Abdomen-RT | cg03181246 | chr20 | 43,243,756 | 43,243,758 | *NA* | -0.118 | (0.017) | 3.5E-08 | -0.077 | 6.7E-08 |
| Abdomen-RT | cg18880175 | chr2 | 114,543,268 | 114,543,270 | *DPP10* | -0.120 | (0.017) | 3.5E-08 | -0.083 | 9.2E-09 |
| Abdomen-RT | cg21771773 | chr5 | 164,296,864 | 164,296,866 | *CTC-340A15.2* | 0.146 | (0.021) | 3.6E-08 | 0.096 | 4.8E-08 |
| Abdomen-RT | cg26066589 | chr16 | 55,996,381 | 55,996,383 | *NA* | -0.080 | (0.011) | 3.7E-08 | -0.056 | 8.2E-09 |
| Abdomen-RT | cg02802072 | chr17 | 81,255,323 | 81,255,325 | *SLC38A10* | 0.067 | (0.010) | 3.7E-08 | 0.042 | 2.0E-07 |
| Abdomen-RT | cg00660643 | chr15 | 94,610,528 | 94,610,530 | *NA* | -0.099 | (0.014) | 4.0E-08 | -0.063 | 1.3E-07 |
| Abdomen-RT | cg24538457 | chr3 | 128,800,477 | 128,800,479 | *RAB7A;RPS15AP16* | -0.091 | (0.013) | 4.0E-08 | -0.065 | 2.8E-09 |
| Abdomen-RT | cg20970886 | chr3 | 192,850,924 | 192,850,926 | *MB21D2* | -0.085 | (0.012) | 4.1E-08 | -0.060 | 4.1E-09 |
| Abdomen-RT | cg05293861 | chr12 | 4,153,541 | 4,153,543 | *NA* | -0.137 | (0.019) | 4.2E-08 | -0.094 | 1.5E-08 |
| Abdomen-RT | cg27478635 | chr20 | 41,003,796 | 41,003,798 | *NA* | -0.144 | (0.020) | 4.2E-08 | -0.091 | 1.5E-07 |
| Abdomen-RT | cg00549798 | chr22 | 40,418,426 | 40,418,428 | *MRTFA* | 0.053 | (0.007) | 4.3E-08 | 0.035 | 2.9E-08 |
| Abdomen-RT | cg17550784 | chr19 | 13,836,589 | 13,836,591 | *CTD-3252C9.4;MIR23A;MIR24-2;MIR27A* | -0.051 | (0.007) | 4.3E-08 | -0.037 | 2.0E-09 |
| Abdomen-RT | cg00770773 | chr3 | 192,853,856 | 192,853,858 | *MB21D2* | 0.126 | (0.018) | 4.3E-08 | 0.080 | 1.5E-07 |
| Abdomen-RT | cg25898281 | chr7 | 89,335,048 | 89,335,050 | *ZNF804B* | -0.100 | (0.014) | 4.4E-08 | -0.068 | 2.0E-08 |
| Abdomen-RT | cg13511623 | chr9 | 123,716,678 | 123,716,680 | *DENND1A* | -0.136 | (0.019) | 4.4E-08 | -0.084 | 3.3E-07 |
| Abdomen-RT | cg22982242 | chr2 | 108,069,673 | 108,069,675 | *NA* | 0.078 | (0.011) | 4.4E-08 | 0.046 | 8.7E-07 |
| Abdomen-RT | cg04005059 | chr2 | 23,496,967 | 23,496,969 | *KLHL29* | -0.082 | (0.012) | 4.6E-08 | -0.052 | 1.6E-07 |
| Abdomen-RT | cg07070413 | chr2 | 27,090,275 | 27,090,277 | *KHK* | -0.081 | (0.012) | 4.7E-08 | -0.056 | 9.5E-09 |
| Abdomen-RT | cg16621591 | chr14 | 98,710,756 | 98,710,758 | *C14orf177* | -0.091 | (0.013) | 4.7E-08 | -0.060 | 7.6E-08 |
| Abdomen-RT | cg13683374 | chr17 | 74,368,627 | 74,368,629 | *GPR142* | -0.067 | (0.010) | 4.7E-08 | -0.044 | 4.3E-08 |
| Abdomen-RT | cg14620941 | chr9 | 136,882,476 | 136,882,478 | *RABL6;TRAF2* | -0.080 | (0.011) | 4.7E-08 | -0.052 | 8.4E-08 |
| Abdomen-RT | cg12009405 | chr6 | 148,330,194 | 148,330,196 | *SASH1* | -0.148 | (0.021) | 4.9E-08 | -0.094 | 2.0E-07 |
| Abdomen-RT | cg22108243 | chr1 | 68,725,462 | 68,725,464 | *NA* | -0.134 | (0.019) | 5.0E-08 | -0.094 | 6.2E-09 |
| Abdomen-RT | cg12049992 | chr18 | 11,147,785 | 11,147,787 | *PIEZO2* | 0.141 | (0.020) | 5.1E-08 | 0.089 | 2.5E-07 |
| Abdomen-RT | cg09130658 | chr17 | 79,927,230 | 79,927,232 | *RP11-353N14.5* | -0.092 | (0.013) | 5.2E-08 | -0.064 | 1.3E-08 |
| Abdomen-RT | cg23884784 | chr14 | 24,313,861 | 24,313,863 | *LTB4R* | -0.052 | (0.007) | 5.3E-08 | -0.035 | 1.8E-08 |
| Abdomen-RT | cg17344906 | chr19 | 13,091,692 | 13,091,694 | *NFIX* | 0.062 | (0.009) | 5.4E-08 | 0.037 | 1.3E-06 |
| Abdomen-RT | cg24597353 | chr4 | 176,793,463 | 176,793,465 | *VEGFC* | 0.139 | (0.020) | 5.5E-08 | 0.096 | 1.7E-08 |
| Abdomen-RT | cg13458803 | chr3 | 119,558,069 | 119,558,071 | *CD80* | -0.076 | (0.011) | 6.0E-08 | -0.053 | 1.5E-08 |
| Abdomen-RT | cg04567724 | chr8 | 138,680,241 | 138,680,243 | *COL22A1* | -0.107 | (0.015) | 6.0E-08 | -0.072 | 4.6E-08 |
| Abdomen-RT | cg01164187 | chr5 | 69,295,179 | 69,295,181 | *CCDC125* | -0.091 | (0.013) | 6.4E-08 | -0.055 | 1.0E-06 |
| Abdomen-RT | cg11614536 | chr10 | 30,059,779 | 30,059,781 | *JCAD* | -0.040 | (0.006) | 6.4E-08 | -0.026 | 9.1E-08 |
| Abdomen-RT | cg12231340 | chr12 | 52,291,436 | 52,291,438 | *KRT81;KRT86* | 0.128 | (0.018) | 6.4E-08 | 0.087 | 3.4E-08 |
| Abdomen-RT | cg25391820 | chr5 | 139,272,829 | 139,272,831 | *MATR3;SIL1;SNHG4* | -0.062 | (0.009) | 6.6E-08 | -0.042 | 2.6E-08 |
| Abdomen-RT | cg19758958 | chr11 | 62,551,749 | 62,551,751 | *AHNAK* | -0.059 | (0.009) | 6.7E-08 | -0.040 | 4.6E-08 |
| Abdomen-RT | cg02786370 | chr4 | 2,746,200 | 2,746,202 | *TNIP2* | -0.114 | (0.016) | 7.0E-08 | -0.068 | 1.1E-06 |
| Abdomen-RT | cg07403981 | chr1 | 98,792,375 | 98,792,377 | *NA* | -0.106 | (0.015) | 7.1E-08 | -0.077 | 4.1E-09 |
| Abdomen-RT | cg26581729 | chr9 | 137,045,339 | 137,045,341 | *NPDC1* | -0.079 | (0.011) | 7.2E-08 | -0.055 | 2.0E-08 |
| Abdomen-RT | cg09896909 | chr5 | 125,549,478 | 125,549,480 | *RP11-756H20.1* | -0.075 | (0.011) | 7.3E-08 | -0.049 | 8.1E-08 |
| Abdomen-RT | cg00931181 | chr2 | 6,770,499 | 6,770,501 | *LINC00487* | -0.089 | (0.013) | 7.4E-08 | -0.056 | 3.5E-07 |
| Abdomen-RT | cg04401758 | chr22 | 41,402,170 | 41,402,172 | *NA* | -0.065 | (0.009) | 7.6E-08 | -0.048 | 2.0E-09 |
| Abdomen-RT | cg06180200 | chr19 | 38,912,295 | 38,912,297 | *CCER2* | 0.089 | (0.013) | 7.7E-08 | 0.057 | 2.7E-07 |
| Abdomen-RT | cg14874646 | chr2 | 6,326,247 | 6,326,249 | *AC017053.1* | -0.088 | (0.013) | 7.8E-08 | -0.060 | 4.6E-08 |
| Abdomen-RT | cg18603250 | chr11 | 94,767,885 | 94,767,887 | *AMOTL1* | -0.067 | (0.010) | 7.8E-08 | -0.048 | 8.7E-09 |
| Abdomen-RT | cg16948781 | chr15 | 44,000,364 | 44,000,366 | *FRMD5* | -0.116 | (0.017) | 7.8E-08 | -0.081 | 1.8E-08 |
| Abdomen-RT | cg17342738 | chr2 | 109,265,641 | 109,265,643 | *SH3RF3* | -0.059 | (0.009) | 7.9E-08 | -0.038 | 1.5E-07 |
| Abdomen-RT | cg25433259 | chr6 | 158,850,751 | 158,850,753 | *OSTCP1* | -0.062 | (0.009) | 8.1E-08 | -0.043 | 1.7E-08 |
| Abdomen-RT | cg08838779 | chr8 | 93,398,777 | 93,398,779 | *LINC00535* | 0.080 | (0.012) | 8.1E-08 | 0.047 | 1.6E-06 |
| Abdomen-RT | cg21302696 | chr14 | 94,390,773 | 94,390,775 | *SERPINA1* | -0.069 | (0.010) | 8.1E-08 | -0.042 | 1.2E-06 |
| Abdomen-RT | cg16300030 | chr6 | 32,941,202 | 32,941,204 | *HLA-DMB;XXbac-BPG181M17.5* | -0.110 | (0.016) | 8.3E-08 | -0.066 | 1.4E-06 |
| Abdomen-RT | cg08578520 | chr16 | 57,462,411 | 57,462,413 | *AC009052.12;POLR2C* | -0.063 | (0.009) | 8.4E-08 | -0.041 | 2.0E-07 |
| Abdomen-RT | cg09853238 | chr6 | 149,211,153 | 149,211,155 | *NA* | -0.066 | (0.010) | 8.5E-08 | -0.046 | 2.5E-08 |
| Abdomen-RT | cg25204718 | chr12 | 96,241,952 | 96,241,954 | *ELK3* | -0.065 | (0.009) | 8.9E-08 | -0.046 | 1.0E-08 |
| Brain-RT | cg13360224 | chr6 | 14,232,103 | 14,232,105 | *RP11-359N11.1* | 0.055 | (0.008) | 1.6E-08 | 0.023 | 1.1E-08 |
| Brain-RT | cg26572901 | chr7 | 27,030,741 | 27,030,743 | *NA* | 0.062 | (0.010) | 1.7E-08 | 0.027 | 1.3E-08 |
| Brain-RT | cg12715065 | chr6 | 14,241,208 | 14,241,210 | *NA* | 0.042 | (0.007) | 1.7E-08 | 0.019 | 1.0E-09 |
| Brain-RT | cg18666944 | chr6 | 16,737,189 | 16,737,191 | *ATXN1* | 0.065 | (0.010) | 1.8E-08 | 0.029 | 6.1E-09 |
| Brain-RT | cg02819464 | chr2 | 73,938,964 | 73,938,966 | *DGUOK* | 0.047 | (0.007) | 2.0E-08 | 0.022 | 6.3E-10 |
| Brain-RT | cg10843537 | chr12 | 123,041,668 | 123,041,670 | *PITPNM2* | 0.060 | (0.009) | 2.1E-08 | 0.025 | 3.9E-08 |
| Brain-RT | cg12608132 | chr2 | 46,337,102 | 46,337,104 | *EPAS1* | 0.065 | (0.010) | 2.3E-08 | 0.027 | 3.8E-08 |
| Brain-RT | cg21163477 | chr16 | 17,345,306 | 17,345,308 | *XYLT1* | 0.050 | (0.008) | 3.2E-08 | 0.020 | 1.3E-07 |
| Brain-RT | cg10159215 | chr1 | 99,005,003 | 99,005,005 | *PLPPR5;RP5-896L10.1* | -0.092 | (0.015) | 8.8E-08 | -0.037 | 4.4E-07 |
| Chest-RT | cg16985233 | chr15 | 43,514,050 | 43,514,052 | *MAP1A* | -0.200 | (0.015) | 8.3E-26 | -0.124 | 7.1E-31 |
| Chest-RT | cg09921385 | chr4 | 6,999,154 | 6,999,156 | *TBC1D14* | -0.195 | (0.015) | 2.0E-25 | -0.123 | 2.5E-31 |
| Chest-RT | cg17593512 | chr4 | 40,439,495 | 40,439,497 | *RBM47* | -0.209 | (0.016) | 1.3E-24 | -0.123 | 1.9E-26 |
| Chest-RT | cg13294852 | chr2 | 102,055,667 | 102,055,669 | *NA* | -0.180 | (0.014) | 2.2E-24 | -0.105 | 6.5E-26 |
| Chest-RT | cg11662039 | chr5 | 100,648,041 | 100,648,043 | *NA* | -0.211 | (0.017) | 1.4E-23 | -0.133 | 4.1E-29 |
| Chest-RT | cg01641136 | chr3 | 50,607,810 | 50,607,812 | *CISH* | -0.166 | (0.013) | 8.2E-23 | -0.102 | 1.1E-26 |
| Chest-RT | cg21585138 | chr3 | 50,607,674 | 50,607,676 | *CISH* | -0.161 | (0.013) | 1.0E-22 | -0.099 | 1.2E-26 |
| Chest-RT | cg27589809 | chr3 | 50,612,978 | 50,612,980 | *CISH;MAPKAPK3* | -0.105 | (0.009) | 1.4E-19 | -0.063 | 1.4E-21 |
| Chest-RT | cg06728103 | chr12 | 93,536,922 | 93,536,924 | *NA* | -0.118 | (0.011) | 3.9E-18 | -0.069 | 1.9E-19 |
| Chest-RT | cg02453046 | chr21 | 15,837,853 | 15,837,855 | *USP25* | -0.112 | (0.010) | 4.8E-18 | -0.067 | 2.3E-20 |
| Chest-RT | cg13224583 | chr1 | 156,913,952 | 156,913,954 | *PEAR1* | 0.120 | (0.011) | 4.5E-17 | 0.066 | 1.9E-16 |
| Chest-RT | cg05293861 | chr12 | 4,153,541 | 4,153,543 | *NA* | -0.194 | (0.018) | 6.1E-17 | -0.115 | 1.2E-18 |
| Chest-RT | cg00973876 | chr17 | 79,925,408 | 79,925,410 | *RP11-353N14.4;RP11-353N14.5* | -0.116 | (0.011) | 2.0E-16 | -0.068 | 1.2E-17 |
| Chest-RT | cg22432387 | chr2 | 20,634,604 | 20,634,606 | *HS1BP3* | -0.208 | (0.020) | 2.6E-16 | -0.122 | 3.1E-17 |
| Chest-RT | cg03834031 | chr22 | 46,069,836 | 46,069,838 | *MIRLET7BHG;RP6-109B7.4* | -0.091 | (0.009) | 3.5E-16 | -0.052 | 1.7E-16 |
| Chest-RT | cg10334733 | chr12 | 93,541,835 | 93,541,837 | *NA* | -0.124 | (0.012) | 4.1E-16 | -0.074 | 5.3E-18 |
| Chest-RT | cg21752134 | chr16 | 79,640,594 | 79,640,596 | *NA* | -0.097 | (0.009) | 5.4E-16 | -0.057 | 2.5E-17 |
| Chest-RT | cg08866213 | chr3 | 192,812,987 | 192,812,989 | *MB21D2* | 0.181 | (0.018) | 5.5E-16 | 0.101 | 1.1E-15 |
| Chest-RT | cg11504495 | chr7 | 48,088,550 | 48,088,552 | *UPP1* | -0.180 | (0.018) | 5.7E-16 | -0.112 | 4.2E-19 |
| Chest-RT | cg11494773 | chr7 | 48,088,644 | 48,088,646 | *UPP1* | -0.156 | (0.015) | 6.3E-16 | -0.099 | 8.5E-20 |
| Chest-RT | cg08271909 | chr4 | 154,740,538 | 154,740,540 | *LRAT* | 0.152 | (0.015) | 8.0E-16 | 0.079 | 2.3E-13 |
| Chest-RT | cg09126320 | chr8 | 22,920,986 | 22,920,988 | *PEBP4* | -0.092 | (0.009) | 1.3E-15 | -0.052 | 2.1E-15 |
| Chest-RT | cg06991974 | chr1 | 3,067,653 | 3,067,655 | *LINC00982* | 0.117 | (0.012) | 1.3E-15 | 0.068 | 1.3E-16 |
| Chest-RT | cg01511232 | chr4 | 154,740,776 | 154,740,778 | *LRAT* | 0.180 | (0.018) | 3.3E-15 | 0.096 | 1.1E-13 |
| Chest-RT | cg22639195 | chr17 | 19,370,112 | 19,370,114 | *B9D1* | -0.101 | (0.010) | 4.6E-15 | -0.062 | 1.3E-17 |
| Chest-RT | cg21945120 | chr10 | 58,157,562 | 58,157,564 | *NA* | -0.130 | (0.013) | 7.4E-15 | -0.065 | 5.9E-12 |
| Chest-RT | cg05186879 | chr3 | 50,612,890 | 50,612,892 | *CISH;MAPKAPK3* | -0.085 | (0.009) | 1.2E-14 | -0.048 | 1.3E-14 |
| Chest-RT | cg25896883 | chr2 | 189,407,943 | 189,407,945 | *NA* | -0.150 | (0.015) | 2.0E-14 | -0.085 | 2.0E-14 |
| Chest-RT | cg03664096 | chr21 | 43,197,746 | 43,197,748 | *NA* | -0.114 | (0.012) | 2.4E-14 | -0.075 | 3.0E-19 |
| Chest-RT | cg17407859 | chr3 | 165,259,995 | 165,259,997 | *LINC01322* | -0.194 | (0.020) | 5.1E-14 | -0.120 | 1.7E-16 |
| Chest-RT | cg25325512 | chr6 | 37,174,443 | 37,174,445 | *PIM1* | -0.136 | (0.014) | 6.4E-14 | -0.084 | 2.7E-16 |
| Chest-RT | cg07946633 | chr1 | 3,067,680 | 3,067,682 | *LINC00982;PRDM16* | 0.091 | (0.010) | 9.3E-14 | 0.052 | 5.0E-14 |
| Chest-RT | cg21359950 | chr12 | 93,689,693 | 93,689,695 | *CRADD* | -0.165 | (0.018) | 1.2E-13 | -0.096 | 2.5E-14 |
| Chest-RT | cg13442016 | chr10 | 102,436,581 | 102,436,583 | *MIR146B* | -0.128 | (0.014) | 1.5E-13 | -0.080 | 3.2E-16 |
| Chest-RT | cg26621020 | chr8 | 122,647,819 | 122,647,821 | *NA* | -0.158 | (0.017) | 1.9E-13 | -0.079 | 6.7E-11 |
| Chest-RT | cg12140144 | chr1 | 3,067,710 | 3,067,712 | *LINC00982;PRDM16* | 0.329 | (0.035) | 2.0E-13 | 0.187 | 1.6E-13 |
| Chest-RT | cg13552692 | chr18 | 68,722,209 | 68,722,211 | *CCDC102B* | -0.126 | (0.014) | 2.3E-13 | -0.070 | 8.6E-13 |
| Chest-RT | cg00218103 | chr22 | 46,070,096 | 46,070,098 | *MIRLET7BHG;RP6-109B7.4* | -0.115 | (0.012) | 2.6E-13 | -0.063 | 1.2E-12 |
| Chest-RT | cg07872945 | chr9 | 134,010,913 | 134,010,915 | *NA* | -0.111 | (0.012) | 3.1E-13 | -0.054 | 4.6E-10 |
| Chest-RT | cg11988169 | chr5 | 53,543,411 | 53,543,413 | *NA* | -0.299 | (0.033) | 5.4E-13 | -0.169 | 6.3E-13 |
| Chest-RT | cg23839200 | chr14 | 51,072,092 | 51,072,094 | *TRIM9* | -0.089 | (0.010) | 5.5E-13 | -0.050 | 5.5E-13 |
| Chest-RT | cg11994851 | chr11 | 94,767,901 | 94,767,903 | *AMOTL1* | -0.078 | (0.009) | 6.4E-13 | -0.039 | 2.0E-10 |
| Chest-RT | cg08101174 | chr10 | 102,436,783 | 102,436,785 | *NA* | -0.099 | (0.011) | 6.6E-13 | -0.061 | 3.0E-15 |
| Chest-RT | cg26342454 | chr4 | 10,033,637 | 10,033,639 | *SLC2A9* | -0.227 | (0.025) | 6.7E-13 | -0.130 | 3.1E-13 |
| Chest-RT | cg17232357 | chr15 | 66,720,493 | 66,720,495 | *SMAD6* | -0.117 | (0.013) | 8.7E-13 | -0.070 | 3.7E-14 |
| Chest-RT | cg03026982 | chr11 | 19,932,152 | 19,932,154 | *NAV2* | -0.112 | (0.013) | 1.3E-12 | -0.064 | 1.2E-12 |
| Chest-RT | cg00117536 | chr7 | 48,088,526 | 48,088,528 | *UPP1* | -0.128 | (0.014) | 1.7E-12 | -0.080 | 4.3E-15 |
| Chest-RT | cg19283806 | chr18 | 68,722,182 | 68,722,184 | *CCDC102B* | -0.094 | (0.011) | 2.0E-12 | -0.053 | 4.4E-12 |
| Chest-RT | cg19634849 | chr5 | 140,259,739 | 140,259,741 | *CYSTM1;PFDN1* | -0.082 | (0.009) | 2.7E-12 | -0.048 | 3.0E-13 |
| Chest-RT | cg17183905 | chr12 | 109,816,036 | 109,816,038 | *TRPV4* | -0.086 | (0.010) | 2.7E-12 | -0.046 | 5.6E-11 |
| Chest-RT | cg04936619 | chr17 | 32,331,517 | 32,331,519 | *C17orf75;RP11-227G15.3* | -0.160 | (0.018) | 3.3E-12 | -0.093 | 6.9E-13 |
| Chest-RT | cg24765521 | chr9 | 90,296,803 | 90,296,805 | *NA* | -0.161 | (0.018) | 4.6E-12 | -0.088 | 1.9E-11 |
| Chest-RT | cg20112376 | chr4 | 6,116,715 | 6,116,717 | *JAKMIP1* | -0.102 | (0.012) | 4.7E-12 | -0.061 | 3.2E-13 |
| Chest-RT | cg15143202 | chr3 | 169,148,736 | 169,148,738 | *MECOM* | -0.139 | (0.016) | 4.7E-12 | -0.073 | 1.3E-10 |
| Chest-RT | cg23776628 | chr1 | 39,633,108 | 39,633,110 | *HEYL;RP1-144F13.3* | 0.127 | (0.015) | 5.2E-12 | 0.071 | 1.2E-11 |
| Chest-RT | cg21393163 | chr1 | 12,157,571 | 12,157,573 | *NA* | -0.135 | (0.015) | 5.5E-12 | -0.075 | 1.6E-11 |
| Chest-RT | cg27192248 | chr15 | 64,993,330 | 64,993,332 | *NA* | -0.239 | (0.028) | 6.5E-12 | -0.137 | 3.0E-12 |
| Chest-RT | cg19434960 | chr21 | 23,922,356 | 23,922,358 | *NA* | -0.106 | (0.012) | 6.6E-12 | -0.065 | 5.9E-14 |
| Chest-RT | cg18661060 | chr10 | 71,758,477 | 71,758,479 | *CDH23;VSIR* | -0.080 | (0.009) | 8.3E-12 | -0.048 | 4.7E-13 |
| Chest-RT | cg16112727 | chr1 | 156,913,429 | 156,913,431 | *PEAR1* | 0.082 | (0.010) | 8.5E-12 | 0.041 | 1.5E-09 |
| Chest-RT | cg27495424 | chr2 | 88,625,579 | 88,625,581 | *EIF2AK3* | -0.073 | (0.008) | 1.0E-11 | -0.047 | 1.3E-14 |
| Chest-RT | cg18787963 | chr17 | 78,413,111 | 78,413,113 | *PGS1* | -0.086 | (0.010) | 1.2E-11 | -0.054 | 7.2E-14 |
| Chest-RT | cg06576867 | chr22 | 46,070,030 | 46,070,032 | *MIRLET7BHG;RP6-109B7.4* | -0.114 | (0.013) | 1.3E-11 | -0.064 | 1.1E-11 |
| Chest-RT | cg04675507 | chr7 | 17,139,014 | 17,139,016 | *NA* | -0.081 | (0.009) | 1.4E-11 | -0.050 | 7.9E-14 |
| Chest-RT | cg13935634 | chr6 | 10,583,535 | 10,583,537 | *GCNT2* | -0.110 | (0.013) | 1.9E-11 | -0.064 | 3.3E-12 |
| Chest-RT | cg16783053 | chr10 | 110,501,436 | 110,501,438 | *DUSP5* | -0.065 | (0.008) | 2.0E-11 | -0.038 | 1.0E-11 |
| Chest-RT | cg01967102 | chr3 | 20,104,381 | 20,104,383 | *KAT2B* | -0.136 | (0.016) | 2.2E-11 | -0.086 | 8.3E-14 |
| Chest-RT | cg19805775 | chr7 | 82,534,800 | 82,534,802 | *NA* | -0.114 | (0.014) | 2.4E-11 | -0.069 | 1.4E-12 |
| Chest-RT | cg18419358 | chr6 | 157,962,976 | 157,962,978 | *NA* | -0.150 | (0.018) | 2.4E-11 | -0.079 | 5.6E-10 |
| Chest-RT | cg09158878 | chr22 | 46,070,027 | 46,070,029 | *MIRLET7BHG;RP6-109B7.4* | -0.115 | (0.014) | 2.4E-11 | -0.061 | 3.5E-10 |
| Chest-RT | cg15857661 | chr10 | 102,436,485 | 102,436,487 | *MIR146B* | -0.113 | (0.013) | 2.5E-11 | -0.068 | 2.0E-12 |
| Chest-RT | cg06963130 | chr2 | 235,434,127 | 235,434,129 | *NA* | -0.118 | (0.014) | 2.5E-11 | -0.059 | 5.0E-09 |
| Chest-RT | cg26663696 | chr3 | 160,403,486 | 160,403,488 | *MIR15B;MIR16-2;RP11-432B6.3;SMC4* | -0.150 | (0.018) | 2.8E-11 | -0.093 | 3.1E-13 |
| Chest-RT | cg07861288 | chr13 | 91,719,216 | 91,719,218 | *GPC5* | -0.082 | (0.010) | 3.8E-11 | -0.045 | 1.1E-10 |
| Chest-RT | cg13329407 | chr21 | 24,429,059 | 24,429,061 | *AP000476.1* | -0.201 | (0.024) | 4.1E-11 | -0.115 | 2.8E-11 |
| Chest-RT | cg17445936 | chr1 | 3,173,780 | 3,173,782 | *PRDM16* | 0.181 | (0.022) | 4.3E-11 | 0.107 | 6.3E-12 |
| Chest-RT | cg06009645 | chr6 | 111,714,895 | 111,714,897 | *FYN* | -0.077 | (0.009) | 4.4E-11 | -0.043 | 1.3E-10 |
| Chest-RT | cg13424484 | chr10 | 62,043,714 | 62,043,716 | *ARID5B* | -0.100 | (0.012) | 4.6E-11 | -0.059 | 5.4E-12 |
| Chest-RT | cg21242123 | chr12 | 52,244,349 | 52,244,351 | *KRT7* | -0.093 | (0.011) | 4.8E-11 | -0.048 | 1.8E-09 |
| Chest-RT | cg07086679 | chr7 | 140,316,321 | 140,316,323 | *SLC37A3* | -0.102 | (0.012) | 5.0E-11 | -0.063 | 1.2E-12 |
| Chest-RT | cg03219453 | chr3 | 138,456,230 | 138,456,232 | *ESYT3* | -0.064 | (0.008) | 5.2E-11 | -0.041 | 8.6E-14 |
| Chest-RT | cg02001279 | chr19 | 940,966 | 940,968 | *ARID3A* | -0.083 | (0.010) | 5.5E-11 | -0.039 | 6.6E-08 |
| Chest-RT | cg27335760 | chr4 | 40,621,877 | 40,621,879 | *RBM47* | 0.086 | (0.010) | 5.9E-11 | 0.048 | 1.1E-10 |
| Chest-RT | cg16621591 | chr14 | 98,710,756 | 98,710,758 | *C14orf177* | -0.102 | (0.012) | 6.2E-11 | -0.062 | 1.5E-12 |
| Chest-RT | cg00264650 | chr9 | 22,030,259 | 22,030,261 | *CDKN2B-AS1;RP11-145E5.5* | 0.074 | (0.009) | 6.3E-11 | 0.043 | 8.1E-12 |
| Chest-RT | cg18598117 | chr19 | 941,125 | 941,127 | *ARID3A* | -0.118 | (0.014) | 6.3E-11 | -0.056 | 4.2E-08 |
| Chest-RT | cg11414254 | chr5 | 177,022,010 | 177,022,012 | *UIMC1;ZNF346* | -0.113 | (0.014) | 6.3E-11 | -0.064 | 7.2E-11 |
| Chest-RT | cg24538457 | chr3 | 128,800,477 | 128,800,479 | *RAB7A;RPS15AP16* | -0.101 | (0.012) | 6.4E-11 | -0.052 | 3.8E-09 |
| Chest-RT | cg02188223 | chr1 | 4,603,220 | 4,603,222 | *NA* | -0.107 | (0.013) | 6.7E-11 | -0.059 | 2.0E-10 |
| Chest-RT | cg09978533 | chr22 | 46,069,279 | 46,069,281 | *MIRLET7BHG;RP6-109B7.4* | -0.085 | (0.010) | 8.0E-11 | -0.046 | 4.7E-10 |
| Chest-RT | cg27569829 | chr18 | 63,317,251 | 63,317,253 | *BCL2* | -0.073 | (0.009) | 8.6E-11 | -0.042 | 3.9E-11 |
| Chest-RT | cg03864568 | chr9 | 76,400,794 | 76,400,796 | *NA* | -0.128 | (0.016) | 8.8E-11 | -0.070 | 2.9E-10 |
| Chest-RT | cg16146033 | chr11 | 62,999,850 | 62,999,852 | *SLC22A8* | -0.102 | (0.012) | 9.7E-11 | -0.053 | 2.6E-09 |
| Chest-RT | cg22677985 | chr7 | 100,624,183 | 100,624,185 | *TFR2* | -0.100 | (0.012) | 1.2E-10 | -0.064 | 2.2E-13 |
| Chest-RT | cg14122922 | chr20 | 44,714,873 | 44,714,875 | *KCNK15-AS1;WISP2* | -0.101 | (0.012) | 1.2E-10 | -0.054 | 1.3E-09 |
| Chest-RT | cg02148547 | chr14 | 20,989,342 | 20,989,344 | *METTL17* | -0.085 | (0.010) | 1.2E-10 | -0.051 | 9.5E-12 |
| Chest-RT | cg24803517 | chr5 | 95,285,812 | 95,285,814 | *MCTP1* | 0.107 | (0.013) | 1.3E-10 | 0.059 | 4.3E-10 |
| Chest-RT | cg04011266 | chr9 | 22,040,986 | 22,040,988 | *CDKN2B-AS1* | -0.076 | (0.009) | 1.3E-10 | -0.049 | 1.8E-13 |
| Chest-RT | cg12051710 | chr12 | 2,879,903 | 2,879,905 | *RHNO1;TULP3* | -0.103 | (0.013) | 1.3E-10 | -0.057 | 3.4E-10 |
| Chest-RT | cg17221955 | chr8 | 92,668,780 | 92,668,782 | *RP11-100L22.1* | -0.084 | (0.010) | 1.4E-10 | -0.054 | 1.9E-13 |
| Chest-RT | cg21443659 | chr2 | 216,880,997 | 216,880,999 | *AC007563.5* | -0.120 | (0.015) | 1.4E-10 | -0.070 | 2.7E-11 |
| Chest-RT | cg10755723 | chr3 | 192,859,174 | 192,859,176 | *MB21D2* | 0.062 | (0.008) | 1.7E-10 | 0.035 | 2.3E-10 |
| Chest-RT | cg13866747 | chr13 | 91,313,553 | 91,313,555 | *NA* | -0.152 | (0.019) | 1.7E-10 | -0.084 | 5.2E-10 |
| Chest-RT | cg09835575 | chr20 | 9,985,215 | 9,985,217 | *ANKEF1;RP5-839B4.8* | -0.121 | (0.015) | 1.7E-10 | -0.073 | 1.1E-11 |
| Chest-RT | cg08178756 | chr22 | 46,070,024 | 46,070,026 | *MIRLET7BHG;RP6-109B7.4* | -0.127 | (0.016) | 1.8E-10 | -0.071 | 2.5E-10 |
| Chest-RT | cg00062245 | chr17 | 44,384,891 | 44,384,893 | *ITGA2B* | 0.095 | (0.012) | 1.8E-10 | 0.058 | 5.3E-12 |
| Chest-RT | cg16902294 | chr4 | 154,740,779 | 154,740,781 | *LRAT* | 0.105 | (0.013) | 1.8E-10 | 0.058 | 7.0E-10 |
| Chest-RT | cg12526942 | chr18 | 63,208,322 | 63,208,324 | *BCL2* | -0.091 | (0.011) | 1.9E-10 | -0.057 | 1.3E-12 |
| Chest-RT | cg08404702 | chr16 | 2,083,386 | 2,083,388 | *TSC2* | 0.075 | (0.009) | 2.3E-10 | 0.042 | 3.7E-10 |
| Chest-RT | cg03065175 | chr4 | 154,740,605 | 154,740,607 | *LRAT* | 0.114 | (0.014) | 2.4E-10 | 0.061 | 2.2E-09 |
| Chest-RT | cg12417457 | chr16 | 66,985,019 | 66,985,021 | *NA* | -0.067 | (0.008) | 2.8E-10 | -0.033 | 6.2E-08 |
| Chest-RT | cg05657694 | chr3 | 100,635,045 | 100,635,047 | *ADGRG7* | -0.144 | (0.018) | 2.8E-10 | -0.073 | 1.8E-08 |
| Chest-RT | cg01446044 | chr1 | 13,624,516 | 13,624,518 | *AL359771.1;RNA5SP41* | -0.059 | (0.007) | 2.8E-10 | -0.034 | 1.5E-10 |
| Chest-RT | cg12634306 | chr1 | 39,633,138 | 39,633,140 | *HEYL;RP1-144F13.3* | 0.120 | (0.015) | 2.9E-10 | 0.068 | 2.2E-10 |
| Chest-RT | cg15251748 | chr7 | 28,953,847 | 28,953,849 | *TRIL* | -0.132 | (0.017) | 3.0E-10 | -0.079 | 3.6E-11 |
| Chest-RT | cg13458803 | chr3 | 119,558,069 | 119,558,071 | *CD80* | -0.083 | (0.010) | 3.5E-10 | -0.047 | 2.7E-10 |
| Chest-RT | cg25790232 | chr16 | 58,215,272 | 58,215,274 | *RP11-459F6.1* | -0.115 | (0.014) | 3.5E-10 | -0.059 | 9.8E-09 |
| Chest-RT | cg11339839 | chr17 | 79,927,329 | 79,927,331 | *RP11-353N14.5* | -0.067 | (0.008) | 3.6E-10 | -0.040 | 2.1E-11 |
| Chest-RT | cg21302696 | chr14 | 94,390,773 | 94,390,775 | *SERPINA1* | -0.076 | (0.010) | 3.7E-10 | -0.045 | 5.3E-11 |
| Chest-RT | cg24769355 | chr9 | 72,041,875 | 72,041,877 | *NA* | -0.130 | (0.016) | 3.8E-10 | -0.067 | 1.5E-08 |
| Chest-RT | cg14263063 | chr16 | 21,150,859 | 21,150,861 | *DNAH3* | -0.125 | (0.016) | 4.8E-10 | -0.058 | 2.9E-07 |
| Chest-RT | cg03296370 | chr3 | 39,281,724 | 39,281,726 | *CX3CR1* | -0.055 | (0.007) | 5.2E-10 | -0.034 | 9.9E-12 |
| Chest-RT | cg10762064 | chr3 | 194,967,685 | 194,967,687 | *NA* | -0.096 | (0.012) | 5.3E-10 | -0.053 | 1.6E-09 |
| Chest-RT | cg25241559 | chr2 | 241,049,961 | 241,049,963 | *AC005237.4;SNED1* | 0.069 | (0.009) | 5.7E-10 | 0.036 | 1.1E-08 |
| Chest-RT | cg04188877 | chr22 | 42,721,943 | 42,721,945 | *A4GALT* | 0.078 | (0.010) | 7.1E-10 | 0.049 | 6.6E-12 |
| Chest-RT | cg11344352 | chr19 | 45,424,437 | 45,424,439 | *ERCC1* | -0.169 | (0.022) | 7.1E-10 | -0.095 | 7.8E-10 |
| Chest-RT | cg12597694 | chr16 | 8,183,208 | 8,183,210 | *NA* | -0.149 | (0.019) | 7.3E-10 | -0.085 | 5.2E-10 |
| Chest-RT | cg14879089 | chr4 | 5,827,008 | 5,827,010 | *CRMP1* | -0.114 | (0.015) | 7.5E-10 | -0.068 | 1.1E-10 |
| Chest-RT | cg17107472 | chr3 | 50,574,267 | 50,574,269 | *HEMK1* | -0.049 | (0.006) | 8.1E-10 | -0.027 | 4.5E-09 |
| Chest-RT | cg08587685 | chr10 | 114,632,446 | 114,632,448 | *ABLIM1* | -0.057 | (0.007) | 8.4E-10 | -0.033 | 3.1E-10 |
| Chest-RT | cg14143795 | chr16 | 21,150,833 | 21,150,835 | *DNAH3* | -0.133 | (0.017) | 8.6E-10 | -0.062 | 4.7E-07 |
| Chest-RT | cg05944249 | chr8 | 27,636,723 | 27,636,725 | *SCARA3* | 0.137 | (0.018) | 9.0E-10 | 0.074 | 4.6E-09 |
| Chest-RT | cg23468816 | chr10 | 62,048,988 | 62,048,990 | *ARID5B* | -0.062 | (0.008) | 9.0E-10 | -0.032 | 2.5E-08 |
| Chest-RT | cg12293170 | chr7 | 132,573,336 | 132,573,338 | *PLXNA4* | 0.089 | (0.012) | 9.4E-10 | 0.057 | 3.0E-12 |
| Chest-RT | cg19080839 | chr7 | 151,242,505 | 151,242,507 | *SMARCD3* | 0.086 | (0.011) | 1.1E-09 | 0.051 | 2.0E-10 |
| Chest-RT | cg02330078 | chr5 | 164,277,917 | 164,277,919 | *NA* | 0.107 | (0.014) | 1.1E-09 | 0.060 | 1.3E-09 |
| Chest-RT | cg21025494 | chr7 | 23,463,124 | 23,463,126 | *IGF2BP3* | -0.077 | (0.010) | 1.1E-09 | -0.044 | 8.9E-10 |
| Chest-RT | cg11614536 | chr10 | 30,059,779 | 30,059,781 | *JCAD* | -0.042 | (0.005) | 1.1E-09 | -0.023 | 3.4E-09 |
| Chest-RT | cg05478824 | chr17 | 82,012,258 | 82,012,260 | *ASPSCR1* | 0.085 | (0.011) | 1.1E-09 | 0.053 | 1.5E-11 |
| Chest-RT | cg11963452 | chr2 | 28,394,684 | 28,394,686 | *FLJ31356;FOSL2* | -0.097 | (0.013) | 1.2E-09 | -0.060 | 4.3E-11 |
| Chest-RT | cg08173263 | chr19 | 14,166,098 | 14,166,100 | *ADGRL1;CTB-55O6.12* | 0.146 | (0.019) | 1.2E-09 | 0.089 | 4.4E-11 |
| Chest-RT | cg18147181 | chr12 | 52,244,373 | 52,244,375 | *KRT7* | -0.084 | (0.011) | 1.3E-09 | -0.045 | 7.6E-09 |
| Chest-RT | cg17783244 | chr22 | 31,342,562 | 31,342,564 | *PATZ1* | 0.092 | (0.012) | 1.3E-09 | 0.055 | 1.5E-10 |
| Chest-RT | cg03957124 | chr6 | 37,049,092 | 37,049,094 | *NA* | -0.070 | (0.009) | 1.3E-09 | -0.038 | 6.3E-09 |
| Chest-RT | cg10473623 | chr11 | 20,097,629 | 20,097,631 | *NAV2* | -0.104 | (0.014) | 1.3E-09 | -0.064 | 3.8E-11 |
| Chest-RT | cg04368942 | chr7 | 134,886,568 | 134,886,570 | *CALD1* | -0.085 | (0.011) | 1.5E-09 | -0.052 | 4.2E-11 |
| Chest-RT | cg00907204 | chr7 | 92,832,656 | 92,832,658 | *CDK6* | -0.101 | (0.013) | 1.6E-09 | -0.064 | 1.4E-11 |
| Chest-RT | cg10941185 | chr8 | 13,131,006 | 13,131,008 | *DLC1* | -0.082 | (0.011) | 1.9E-09 | -0.053 | 6.0E-12 |
| Chest-RT | cg06308882 | chr18 | 2,986,173 | 2,986,175 | *LPIN2* | -0.080 | (0.011) | 1.9E-09 | -0.047 | 3.1E-10 |
| Chest-RT | cg08783988 | chr10 | 62,048,990 | 62,048,992 | *ARID5B* | -0.067 | (0.009) | 2.0E-09 | -0.036 | 8.0E-09 |
| Chest-RT | cg12713583 | chr19 | 940,723 | 940,725 | *ARID3A* | -0.141 | (0.019) | 2.2E-09 | -0.066 | 6.9E-07 |
| Chest-RT | cg13432102 | chr14 | 24,692,986 | 24,692,988 | *NA* | -0.073 | (0.010) | 2.4E-09 | -0.047 | 1.7E-11 |
| Chest-RT | cg05331731 | chr18 | 11,147,146 | 11,147,148 | *PIEZO2* | 0.160 | (0.021) | 2.4E-09 | 0.079 | 2.1E-07 |
| Chest-RT | cg17516475 | chr22 | 46,044,588 | 46,044,590 | *LINC00899* | -0.109 | (0.014) | 2.5E-09 | -0.058 | 2.6E-08 |
| Chest-RT | cg25788793 | chr4 | 10,032,955 | 10,032,957 | *SLC2A9* | -0.078 | (0.010) | 2.8E-09 | -0.047 | 2.1E-10 |
| Chest-RT | cg04495491 | chr22 | 42,671,552 | 42,671,554 | *NA* | -0.089 | (0.012) | 2.9E-09 | -0.058 | 8.4E-12 |
| Chest-RT | cg16060790 | chr9 | 101,372,935 | 101,372,937 | *BAAT* | -0.129 | (0.017) | 3.0E-09 | -0.065 | 1.1E-07 |
| Chest-RT | cg05251190 | chr10 | 102,436,448 | 102,436,450 | *MIR146B* | -0.069 | (0.009) | 3.0E-09 | -0.043 | 5.6E-11 |
| Chest-RT | cg02699612 | chr10 | 113,753,520 | 113,753,522 | *PLEKHS1* | -0.154 | (0.021) | 3.0E-09 | -0.086 | 4.3E-09 |
| Chest-RT | cg27021709 | chr15 | 81,141,587 | 81,141,589 | *CFAP161* | 0.066 | (0.009) | 3.0E-09 | 0.040 | 1.9E-10 |
| Chest-RT | cg13801402 | chr1 | 113,887,342 | 113,887,344 | *AP4B1-AS1;BCL2L15* | -0.118 | (0.016) | 3.1E-09 | -0.069 | 8.4E-10 |
| Chest-RT | cg12530994 | chr10 | 5,094,589 | 5,094,591 | *AKR1C1;AKR1C2;AKR1C3;SNORD118* | -0.114 | (0.015) | 3.2E-09 | -0.059 | 5.1E-08 |
| Chest-RT | cg15871086 | chr18 | 58,859,362 | 58,859,364 | *NA* | -0.086 | (0.011) | 3.7E-09 | -0.050 | 1.1E-09 |
| Chest-RT | cg15890546 | chr3 | 194,967,758 | 194,967,760 | *NA* | -0.081 | (0.011) | 3.8E-09 | -0.042 | 5.3E-08 |
| Chest-RT | cg12960124 | chr4 | 82,100,021 | 82,100,023 | *NA* | -0.109 | (0.015) | 3.9E-09 | -0.065 | 5.1E-10 |
| Chest-RT | cg18462381 | chr10 | 127,738,453 | 127,738,455 | *FOXI2;RP11-288A5.2* | 0.114 | (0.015) | 4.1E-09 | 0.057 | 2.2E-07 |
| Chest-RT | cg17200702 | chr11 | 30,992,376 | 30,992,378 | *DCDC1* | -0.124 | (0.017) | 4.9E-09 | -0.071 | 2.9E-09 |
| Chest-RT | cg08119153 | chr14 | 78,446,957 | 78,446,959 | *NRXN3* | 0.123 | (0.017) | 5.0E-09 | 0.074 | 4.9E-10 |
| Chest-RT | cg16618104 | chr12 | 104,459,321 | 104,459,323 | *CHST11* | -0.113 | (0.015) | 5.0E-09 | -0.067 | 9.5E-10 |
| Chest-RT | cg12442513 | chr2 | 157,807,352 | 157,807,354 | *ACVR1* | -0.102 | (0.014) | 5.0E-09 | -0.066 | 2.5E-11 |
| Chest-RT | cg11575350 | chr21 | 24,428,713 | 24,428,715 | *AP000476.1* | -0.102 | (0.014) | 5.0E-09 | -0.056 | 2.1E-08 |
| Chest-RT | cg20237595 | chr3 | 124,582,404 | 124,582,406 | *KALRN* | -0.071 | (0.010) | 5.5E-09 | -0.040 | 5.2E-09 |
| Chest-RT | cg15491247 | chr1 | 3,313,859 | 3,313,861 | *PRDM16* | -0.084 | (0.011) | 5.7E-09 | -0.046 | 1.4E-08 |
| Chest-RT | cg26910511 | chr19 | 39,308,396 | 39,308,398 | *LRFN1* | 0.081 | (0.011) | 5.8E-09 | 0.040 | 3.2E-07 |
| Chest-RT | cg00594907 | chr1 | 8,945,659 | 8,945,661 | *CA6* | -0.063 | (0.009) | 5.8E-09 | -0.039 | 2.9E-10 |
| Chest-RT | cg26189283 | chr1 | 155,136,901 | 155,136,903 | *SLC50A1* | -0.093 | (0.013) | 5.9E-09 | -0.052 | 1.1E-08 |
| Chest-RT | cg12627844 | chr2 | 64,017,865 | 64,017,867 | *VPS54* | -0.088 | (0.012) | 6.0E-09 | -0.052 | 1.3E-09 |
| Chest-RT | cg19789753 | chr2 | 241,863,809 | 241,863,811 | *NA* | -0.140 | (0.019) | 6.5E-09 | -0.088 | 1.2E-10 |
| Chest-RT | cg11154555 | chr5 | 176,555,643 | 176,555,645 | *CDHR2;Y_RNA* | -0.076 | (0.010) | 6.6E-09 | -0.047 | 1.9E-10 |
| Chest-RT | cg01595702 | chr5 | 53,069,789 | 53,069,791 | *ITGA2* | -0.087 | (0.012) | 6.8E-09 | -0.052 | 1.3E-09 |
| Chest-RT | cg22398226 | chr4 | 154,741,064 | 154,741,066 | *LRAT* | 0.078 | (0.011) | 6.9E-09 | 0.046 | 2.2E-09 |
| Chest-RT | cg11093223 | chr9 | 34,363,683 | 34,363,685 | *NA* | 0.054 | (0.007) | 7.7E-09 | 0.032 | 1.2E-09 |
| Chest-RT | cg06535121 | chr4 | 26,088,488 | 26,088,490 | *NA* | -0.071 | (0.010) | 8.3E-09 | -0.038 | 5.3E-08 |
| Chest-RT | cg05135521 | chr2 | 160,331,823 | 160,331,825 | *RBMS1* | -0.097 | (0.013) | 8.3E-09 | -0.052 | 5.9E-08 |
| Chest-RT | cg13910395 | chr22 | 43,577,140 | 43,577,142 | *EFCAB6* | -0.079 | (0.011) | 8.6E-09 | -0.040 | 3.4E-07 |
| Chest-RT | cg09519959 | chr17 | 28,643,897 | 28,643,899 | *KIAA0100* | -0.081 | (0.011) | 9.0E-09 | -0.048 | 2.9E-09 |
| Chest-RT | cg13806740 | chr17 | 17,971,714 | 17,971,716 | *DRC3;TOM1L2* | -0.057 | (0.008) | 9.1E-09 | -0.032 | 1.7E-08 |
| Chest-RT | cg23522611 | chr2 | 28,394,744 | 28,394,746 | *FLJ31356;FOSL2* | -0.059 | (0.008) | 9.1E-09 | -0.036 | 4.7E-10 |
| Chest-RT | cg05483571 | chr17 | 79,927,424 | 79,927,426 | *RP11-353N14.5* | -0.107 | (0.015) | 9.2E-09 | -0.061 | 8.2E-09 |
| Chest-RT | cg02209802 | chr16 | 11,231,457 | 11,231,459 | *NA* | -0.069 | (0.009) | 9.2E-09 | -0.038 | 1.5E-08 |
| Chest-RT | cg10541181 | chr12 | 23,559,658 | 23,559,660 | *SOX5* | -0.143 | (0.020) | 9.5E-09 | -0.081 | 7.2E-09 |
| Chest-RT | cg03918859 | chr3 | 50,581,057 | 50,581,059 | *HEMK1* | -0.077 | (0.011) | 9.6E-09 | -0.045 | 2.7E-09 |
| Chest-RT | cg22156456 | chr17 | 41,687,986 | 41,687,988 | *EIF1* | -0.069 | (0.010) | 9.7E-09 | -0.041 | 1.3E-09 |
| Chest-RT | cg08066673 | chr14 | 51,859,028 | 51,859,030 | *GNG2* | -0.070 | (0.010) | 9.9E-09 | -0.041 | 4.1E-09 |
| Chest-RT | cg06130949 | chr7 | 48,088,412 | 48,088,414 | *UPP1* | -0.058 | (0.008) | 1.0E-08 | -0.035 | 4.8E-10 |
| Chest-RT | cg25352208 | chr1 | 9,807,614 | 9,807,616 | *CLSTN1* | 0.089 | (0.012) | 1.0E-08 | 0.059 | 1.5E-11 |
| Chest-RT | cg05858126 | chr10 | 102,436,455 | 102,436,457 | *MIR146B* | -0.090 | (0.012) | 1.1E-08 | -0.056 | 3.9E-10 |
| Chest-RT | cg07886756 | chr3 | 139,339,491 | 139,339,493 | *MRPS22* | -0.070 | (0.010) | 1.2E-08 | -0.041 | 2.4E-09 |
| Chest-RT | cg07265924 | chr8 | 60,967,910 | 60,967,912 | *AC022182.3* | -0.169 | (0.023) | 1.2E-08 | -0.100 | 2.9E-09 |
| Chest-RT | cg00549798 | chr22 | 40,418,426 | 40,418,428 | *MRTFA* | 0.051 | (0.007) | 1.2E-08 | 0.028 | 5.1E-08 |
| Chest-RT | cg01869007 | chr8 | 60,967,853 | 60,967,855 | *AC022182.3* | -0.147 | (0.020) | 1.2E-08 | -0.086 | 3.1E-09 |
| Chest-RT | cg08585946 | chr2 | 234,492,122 | 234,492,124 | *NA* | -0.062 | (0.009) | 1.4E-08 | -0.035 | 1.3E-08 |
| Chest-RT | cg10767662 | chr19 | 13,016,914 | 13,016,916 | *NFIX* | 0.065 | (0.009) | 1.5E-08 | 0.032 | 5.8E-07 |
| Chest-RT | cg01002223 | chr1 | 8,926,974 | 8,926,976 | *NA* | -0.122 | (0.017) | 1.5E-08 | -0.073 | 1.8E-09 |
| Chest-RT | cg02606535 | chr16 | 2,083,030 | 2,083,032 | *TSC2* | 0.059 | (0.008) | 1.5E-08 | 0.035 | 3.0E-09 |
| Chest-RT | cg06586734 | chr4 | 61,143,607 | 61,143,609 | *RP11-16N2.1* | -0.108 | (0.015) | 1.5E-08 | -0.050 | 3.5E-06 |
| Chest-RT | cg12800962 | chr20 | 50,009,899 | 50,009,901 | *NA* | 0.095 | (0.013) | 1.6E-08 | 0.053 | 2.6E-08 |
| Chest-RT | cg16660220 | chr11 | 46,555,397 | 46,555,399 | *AMBRA1* | -0.080 | (0.011) | 1.7E-08 | -0.044 | 4.1E-08 |
| Chest-RT | cg09606564 | chr17 | 19,387,039 | 19,387,041 | *MFAP4* | 0.053 | (0.007) | 1.8E-08 | 0.031 | 4.0E-09 |
| Chest-RT | cg04433322 | chr17 | 44,269,525 | 44,269,527 | *SLC4A1* | -0.054 | (0.008) | 1.8E-08 | -0.030 | 2.8E-08 |
| Chest-RT | cg25940196 | chr14 | 33,184,862 | 33,184,864 | *NPAS3* | -0.084 | (0.012) | 1.9E-08 | -0.050 | 4.6E-09 |
| Chest-RT | cg05206206 | chr17 | 28,890,177 | 28,890,179 | *FLOT2* | -0.090 | (0.013) | 1.9E-08 | -0.050 | 3.0E-08 |
| Chest-RT | cg04665818 | chr4 | 88,737,743 | 88,737,745 | *FAM13A* | 0.053 | (0.008) | 1.9E-08 | 0.035 | 5.5E-11 |
| Chest-RT | cg22520453 | chr21 | 35,478,271 | 35,478,273 | *RUNX1* | -0.086 | (0.012) | 1.9E-08 | -0.052 | 1.7E-09 |
| Chest-RT | cg06178669 | chr11 | 63,567,135 | 63,567,137 | *NA* | -0.121 | (0.017) | 2.0E-08 | -0.065 | 1.2E-07 |
| Chest-RT | cg16394551 | chr1 | 3,313,792 | 3,313,794 | *PRDM16* | -0.088 | (0.012) | 2.0E-08 | -0.049 | 4.7E-08 |
| Chest-RT | cg03875496 | chr6 | 105,887,798 | 105,887,800 | *NA* | 0.060 | (0.008) | 2.0E-08 | 0.041 | 6.2E-12 |
| Chest-RT | cg05712639 | chr14 | 52,352,667 | 52,352,669 | *NA* | -0.159 | (0.022) | 2.1E-08 | -0.095 | 3.4E-09 |
| Chest-RT | cg09539785 | chr22 | 24,729,643 | 24,729,645 | *PIWIL3* | -0.085 | (0.012) | 2.1E-08 | -0.045 | 1.3E-07 |
| Chest-RT | cg05336220 | chr16 | 17,346,753 | 17,346,755 | *XYLT1* | -0.057 | (0.008) | 2.1E-08 | -0.039 | 1.1E-11 |
| Chest-RT | cg00135497 | chr4 | 154,740,696 | 154,740,698 | *LRAT* | 0.071 | (0.010) | 2.1E-08 | 0.039 | 5.8E-08 |
| Chest-RT | cg19152802 | chr5 | 110,514,185 | 110,514,187 | *MIR548F3;TMEM232* | -0.108 | (0.015) | 2.2E-08 | -0.060 | 4.9E-08 |
| Chest-RT | cg09853238 | chr6 | 149,211,153 | 149,211,155 | *NA* | -0.065 | (0.009) | 2.2E-08 | -0.039 | 3.6E-09 |
| Chest-RT | cg22976567 | chr1 | 156,104,390 | 156,104,392 | *LMNA* | -0.074 | (0.010) | 2.2E-08 | -0.040 | 1.0E-07 |
| Chest-RT | cg24914621 | chr5 | 146,825,185 | 146,825,187 | *PPP2R2B* | 0.098 | (0.014) | 2.2E-08 | 0.055 | 3.0E-08 |
| Chest-RT | cg16467725 | chr10 | 115,799,534 | 115,799,536 | *ATRNL1* | -0.110 | (0.016) | 2.3E-08 | -0.062 | 3.1E-08 |
| Chest-RT | cg21385305 | chr2 | 23,490,082 | 23,490,084 | *KLHL29* | -0.094 | (0.013) | 2.3E-08 | -0.052 | 4.1E-08 |
| Chest-RT | cg15705813 | chr2 | 70,070,366 | 70,070,368 | *PCBP1-AS1* | -0.062 | (0.009) | 2.5E-08 | -0.032 | 4.8E-07 |
| Chest-RT | cg13486805 | chr19 | 39,248,023 | 39,248,025 | *IFNL4* | 0.088 | (0.013) | 2.7E-08 | 0.049 | 6.3E-08 |
| Chest-RT | cg21781784 | chr1 | 227,437,757 | 227,437,759 | *NA* | -0.118 | (0.017) | 2.7E-08 | -0.064 | 8.3E-08 |
| Chest-RT | cg03480001 | chr13 | 113,633,340 | 113,633,342 | *TFDP1* | 0.099 | (0.014) | 2.7E-08 | 0.064 | 1.6E-10 |
| Chest-RT | cg14028115 | chr2 | 168,802,559 | 168,802,561 | *NOSTRIN* | -0.070 | (0.010) | 2.8E-08 | -0.042 | 3.7E-09 |
| Chest-RT | cg13924715 | chr11 | 10,729,342 | 10,729,344 | *NA* | 0.166 | (0.024) | 2.9E-08 | 0.080 | 2.2E-06 |
| Chest-RT | cg03877706 | chr21 | 21,200,312 | 21,200,314 | *NCAM2* | -0.166 | (0.024) | 2.9E-08 | -0.103 | 1.2E-09 |
| Chest-RT | cg15246238 | chr7 | 5,595,502 | 5,595,504 | *FSCN1* | -0.087 | (0.012) | 3.0E-08 | -0.049 | 2.7E-08 |
| Chest-RT | cg04973995 | chr10 | 72,298,218 | 72,298,220 | *NA* | -0.102 | (0.015) | 3.1E-08 | -0.061 | 4.5E-09 |
| Chest-RT | cg18603250 | chr11 | 94,767,885 | 94,767,887 | *AMOTL1* | -0.065 | (0.009) | 3.3E-08 | -0.036 | 9.3E-08 |
| Chest-RT | cg07501635 | chr3 | 53,680,140 | 53,680,142 | *CACNA1D* | 0.177 | (0.025) | 3.3E-08 | 0.096 | 1.3E-07 |
| Chest-RT | cg01143068 | chr15 | 42,595,980 | 42,595,982 | *STARD9* | 0.050 | (0.007) | 3.3E-08 | 0.031 | 2.9E-09 |
| Chest-RT | cg19458269 | chr1 | 155,979,629 | 155,979,631 | *ARHGEF2;RP11-336K24.12* | -0.053 | (0.008) | 3.3E-08 | -0.032 | 3.2E-09 |
| Chest-RT | cg04494298 | chr7 | 31,812,033 | 31,812,035 | *PDE1C* | -0.084 | (0.012) | 3.4E-08 | -0.050 | 8.6E-09 |
| Chest-RT | cg24327132 | chr15 | 72,228,290 | 72,228,292 | *PKM* | -0.077 | (0.011) | 3.6E-08 | -0.039 | 8.0E-07 |
| Chest-RT | cg14127237 | chr17 | 17,820,147 | 17,820,149 | *SREBF1* | -0.054 | (0.008) | 3.7E-08 | -0.034 | 7.8E-10 |
| Chest-RT | cg16449837 | chr12 | 15,279,392 | 15,279,394 | *RERG* | -0.066 | (0.009) | 3.7E-08 | -0.042 | 6.6E-10 |
| Chest-RT | cg01838523 | chr20 | 44,714,648 | 44,714,650 | *KCNK15-AS1;WISP2* | -0.065 | (0.009) | 3.7E-08 | -0.039 | 9.0E-09 |
| Chest-RT | cg07070413 | chr2 | 27,090,275 | 27,090,277 | *KHK* | -0.076 | (0.011) | 3.9E-08 | -0.045 | 1.1E-08 |
| Chest-RT | cg08774778 | chr5 | 62,082,559 | 62,082,561 | *NA* | -0.094 | (0.014) | 4.0E-08 | -0.054 | 2.3E-08 |
| Chest-RT | cg09130658 | chr17 | 79,927,230 | 79,927,232 | *RP11-353N14.5* | -0.087 | (0.013) | 4.1E-08 | -0.052 | 4.9E-09 |
| Chest-RT | cg15862165 | chr12 | 93,767,339 | 93,767,341 | *CRADD* | -0.080 | (0.012) | 4.1E-08 | -0.040 | 1.6E-06 |
| Chest-RT | cg03116755 | chr3 | 15,556,625 | 15,556,627 | *NA* | -0.092 | (0.013) | 4.2E-08 | -0.056 | 3.3E-09 |
| Chest-RT | cg20351364 | chr5 | 176,555,547 | 176,555,549 | *CDHR2;Y_RNA* | -0.089 | (0.013) | 4.3E-08 | -0.052 | 1.3E-08 |
| Chest-RT | cg05316065 | chr8 | 129,786,760 | 129,786,762 | *GSDMC* | -0.044 | (0.006) | 4.3E-08 | -0.025 | 3.9E-08 |
| Chest-RT | cg04882213 | chr8 | 60,967,832 | 60,967,834 | *AC022182.3* | -0.115 | (0.017) | 4.3E-08 | -0.065 | 4.4E-08 |
| Chest-RT | cg01360622 | chr17 | 42,311,406 | 42,311,408 | *STAT5A* | 0.063 | (0.009) | 4.4E-08 | 0.043 | 3.5E-11 |
| Chest-RT | cg05848650 | chr3 | 192,893,050 | 192,893,052 | *MB21D2* | -0.131 | (0.019) | 4.4E-08 | -0.078 | 1.1E-08 |
| Chest-RT | cg18880175 | chr2 | 114,543,268 | 114,543,270 | *DPP10* | -0.112 | (0.016) | 4.5E-08 | -0.060 | 2.6E-07 |
| Chest-RT | cg06007201 | chr16 | 88,783,809 | 88,783,811 | *PIEZO1* | -0.095 | (0.014) | 4.5E-08 | -0.057 | 6.9E-09 |
| Chest-RT | cg27136634 | chr11 | 17,414,582 | 17,414,584 | *ABCC8* | -0.091 | (0.013) | 4.5E-08 | -0.051 | 6.6E-08 |
| Chest-RT | cg27326027 | chr18 | 6,885,253 | 6,885,255 | *ARHGAP28* | -0.118 | (0.017) | 4.6E-08 | -0.075 | 6.1E-10 |
| Chest-RT | cg09317107 | chr1 | 56,788,784 | 56,788,786 | *FYB2* | -0.063 | (0.009) | 4.6E-08 | -0.033 | 3.1E-07 |
| Chest-RT | cg12467226 | chr3 | 13,652,983 | 13,652,985 | *LINC00620* | -0.051 | (0.007) | 4.6E-08 | -0.031 | 3.1E-09 |
| Chest-RT | cg25339841 | chr11 | 18,768,672 | 18,768,674 | *PTPN5* | -0.082 | (0.012) | 4.7E-08 | -0.053 | 3.7E-10 |
| Chest-RT | cg04849850 | chr2 | 85,893,876 | 85,893,878 | *NA* | -0.061 | (0.009) | 4.7E-08 | -0.037 | 7.6E-09 |
| Chest-RT | cg01464849 | chr3 | 160,402,692 | 160,402,694 | *RP11-432B6.3;SMC4* | -0.127 | (0.019) | 4.8E-08 | -0.078 | 2.8E-09 |
| Chest-RT | cg10570484 | chr1 | 158,038,015 | 158,038,017 | *KIRREL1* | -0.056 | (0.008) | 5.0E-08 | -0.033 | 1.9E-08 |
| Chest-RT | cg14270417 | chr14 | 63,864,100 | 63,864,102 | *SYNE2* | -0.060 | (0.009) | 5.1E-08 | -0.036 | 6.5E-09 |
| Chest-RT | cg21007318 | chr22 | 42,721,314 | 42,721,316 | *A4GALT* | 0.058 | (0.009) | 5.1E-08 | 0.034 | 1.7E-08 |
| Chest-RT | cg22234488 | chr2 | 105,179,803 | 105,179,805 | *NA* | -0.089 | (0.013) | 5.1E-08 | -0.054 | 5.8E-09 |
| Chest-RT | cg04372796 | chr11 | 128,525,189 | 128,525,191 | *ETS1;RP11-1007G5.2* | -0.070 | (0.010) | 5.3E-08 | -0.039 | 1.2E-07 |
| Chest-RT | cg12569593 | chr11 | 126,543,820 | 126,543,822 | *KIRREL3;KIRREL3-AS1* | -0.057 | (0.008) | 5.4E-08 | -0.035 | 5.5E-09 |
| Chest-RT | cg15352568 | chr1 | 209,875,771 | 209,875,773 | *NA* | -0.078 | (0.011) | 5.5E-08 | -0.039 | 1.3E-06 |
| Chest-RT | cg15444185 | chr8 | 66,542,471 | 66,542,473 | *NA* | 0.083 | (0.012) | 5.6E-08 | 0.047 | 8.4E-08 |
| Chest-RT | cg25204718 | chr12 | 96,241,952 | 96,241,954 | *ELK3* | -0.062 | (0.009) | 5.7E-08 | -0.035 | 4.1E-08 |
| Chest-RT | cg10202782 | chr9 | 101,372,961 | 101,372,963 | *BAAT* | -0.106 | (0.016) | 5.8E-08 | -0.058 | 2.0E-07 |
| Chest-RT | cg17897728 | chr3 | 172,132,290 | 172,132,292 | *FNDC3B* | -0.062 | (0.009) | 5.8E-08 | -0.037 | 9.8E-09 |
| Chest-RT | cg07097098 | chr19 | 55,210,269 | 55,210,271 | *PTPRH* | -0.076 | (0.011) | 5.9E-08 | -0.046 | 6.0E-09 |
| Chest-RT | cg04697265 | chr11 | 130,813,804 | 130,813,806 | *NA* | -0.073 | (0.011) | 5.9E-08 | -0.044 | 9.6E-09 |
| Chest-RT | cg23120900 | chr15 | 59,271,867 | 59,271,869 | *MYO1E;RP11-429D19.1* | -0.099 | (0.014) | 5.9E-08 | -0.060 | 5.4E-09 |
| Chest-RT | cg18676273 | chr7 | 41,693,234 | 41,693,236 | *AC005027.3;INHBA;INHBA-AS1* | -0.072 | (0.010) | 6.1E-08 | -0.041 | 3.7E-08 |
| Chest-RT | cg17631924 | chr17 | 28,034,180 | 28,034,182 | *NA* | -0.067 | (0.010) | 6.1E-08 | -0.034 | 1.0E-06 |
| Chest-RT | cg01435315 | chr10 | 70,588,663 | 70,588,665 | *NA* | -0.106 | (0.015) | 6.2E-08 | -0.061 | 3.1E-08 |
| Chest-RT | cg00002033 | chr19 | 39,307,840 | 39,307,842 | *LRFN1* | 0.097 | (0.014) | 6.2E-08 | 0.052 | 3.5E-07 |
| Chest-RT | cg07195404 | chr5 | 35,861,868 | 35,861,870 | *IL7R* | 0.084 | (0.012) | 6.2E-08 | 0.050 | 1.0E-08 |
| Chest-RT | cg24597353 | chr4 | 176,793,463 | 176,793,465 | *VEGFC* | 0.130 | (0.019) | 6.3E-08 | 0.084 | 7.0E-10 |
| Chest-RT | cg23310123 | chr1 | 28,144,696 | 28,144,698 | *NA* | -0.090 | (0.013) | 6.4E-08 | -0.051 | 7.2E-08 |
| Chest-RT | cg24833462 | chr2 | 108,053,726 | 108,053,728 | *AC023672.2* | -0.160 | (0.024) | 6.8E-08 | -0.094 | 2.4E-08 |
| Chest-RT | cg06855546 | chr16 | 1,488,824 | 1,488,826 | *PTX4* | 0.065 | (0.010) | 6.8E-08 | 0.035 | 2.5E-07 |
| Chest-RT | cg25695450 | chr7 | 41,219,438 | 41,219,440 | *NA* | -0.087 | (0.013) | 7.1E-08 | -0.047 | 2.7E-07 |
| Chest-RT | cg14200569 | chr1 | 3,135,360 | 3,135,362 | *PRDM16* | 0.126 | (0.019) | 7.1E-08 | 0.078 | 4.0E-09 |
| Chest-RT | cg15416179 | chr17 | 21,286,546 | 21,286,548 | *MAP2K3* | -0.095 | (0.014) | 7.1E-08 | -0.056 | 2.8E-08 |
| Chest-RT | cg20833121 | chr2 | 127,276,844 | 127,276,846 | *ERCC3* | -0.093 | (0.014) | 7.2E-08 | -0.055 | 1.7E-08 |
| Chest-RT | cg11840258 | chr20 | 50,226,580 | 50,226,582 | *NA* | -0.048 | (0.007) | 7.6E-08 | -0.028 | 2.0E-08 |
| Chest-RT | cg16548911 | chr16 | 50,313,854 | 50,313,856 | *ADCY7;BRD7* | 0.091 | (0.013) | 7.7E-08 | 0.057 | 1.8E-09 |
| Chest-RT | cg12769615 | chr4 | 94,732,004 | 94,732,006 | *NA* | -0.081 | (0.012) | 7.7E-08 | -0.047 | 3.9E-08 |
| Chest-RT | cg16198075 | chr4 | 6,417,112 | 6,417,114 | *PPP2R2C* | 0.103 | (0.015) | 7.8E-08 | 0.058 | 7.0E-08 |
| Chest-RT | cg03479114 | chr4 | 165,359,572 | 165,359,574 | *NA* | -0.098 | (0.014) | 7.8E-08 | -0.065 | 3.1E-10 |
| Chest-RT | cg25987136 | chr1 | 3,367,668 | 3,367,670 | *PRDM16* | -0.059 | (0.009) | 7.9E-08 | -0.031 | 7.7E-07 |
| Chest-RT | cg02541477 | chr13 | 52,061,901 | 52,061,903 | *NEK5* | -0.144 | (0.021) | 8.2E-08 | -0.083 | 5.0E-08 |
| Chest-RT | cg13848363 | chr1 | 206,575,860 | 206,575,862 | *EIF2D;RASSF5* | -0.058 | (0.009) | 8.2E-08 | -0.033 | 9.1E-08 |
| Chest-RT | cg09762316 | chr9 | 70,043,437 | 70,043,439 | *MAMDC2;MAMDC2-AS1* | 0.075 | (0.011) | 8.3E-08 | 0.044 | 2.5E-08 |
| Chest-RT | cg12202498 | chr3 | 15,441,097 | 15,441,099 | *EAF1;EAF1-AS1;METTL6* | -0.057 | (0.009) | 8.3E-08 | -0.028 | 3.0E-06 |
| Chest-RT | cg14129477 | chr17 | 55,344,858 | 55,344,860 | *NA* | -0.079 | (0.012) | 8.5E-08 | -0.043 | 1.8E-07 |
| Chest-RT | cg07323488 | chr3 | 168,467,524 | 168,467,526 | *EGFEM1P* | -0.075 | (0.011) | 8.8E-08 | -0.043 | 4.2E-08 |
| Pelvic-RT | cg07872945 | chr9 | 134,010,913 | 134,010,915 | *NA* | -0.141 | (0.013) | 3.2E-19 | -0.080 | 9.6E-20 |
| Pelvic-RT | cg13224583 | chr1 | 156,913,952 | 156,913,954 | *PEAR1* | 0.130 | (0.012) | 1.5E-18 | 0.076 | 1.7E-20 |
| Pelvic-RT | cg21945120 | chr10 | 58,157,562 | 58,157,564 | *NA* | -0.151 | (0.014) | 2.7E-18 | -0.086 | 3.4E-19 |
| Pelvic-RT | cg22432387 | chr2 | 20,634,604 | 20,634,606 | *HS1BP3* | -0.229 | (0.022) | 4.2E-18 | -0.135 | 4.3E-20 |
| Pelvic-RT | cg26342454 | chr4 | 10,033,637 | 10,033,639 | *SLC2A9* | -0.279 | (0.027) | 1.3E-17 | -0.167 | 3.2E-20 |
| Pelvic-RT | cg16112727 | chr1 | 156,913,429 | 156,913,431 | *PEAR1* | 0.100 | (0.010) | 8.7E-16 | 0.056 | 5.8E-16 |
| Pelvic-RT | cg11988169 | chr5 | 53,543,411 | 53,543,413 | *NA* | -0.344 | (0.035) | 1.0E-15 | -0.202 | 2.9E-17 |
| Pelvic-RT | cg03834031 | chr22 | 46,069,836 | 46,069,838 | *MIRLET7BHG;RP6-109B7.4* | -0.092 | (0.010) | 2.2E-15 | -0.053 | 2.0E-16 |
| Pelvic-RT | cg03877706 | chr21 | 21,200,312 | 21,200,314 | *NCAM2* | -0.243 | (0.025) | 3.0E-15 | -0.148 | 6.7E-18 |
| Pelvic-RT | cg21242123 | chr12 | 52,244,349 | 52,244,351 | *KRT7* | -0.116 | (0.012) | 3.1E-15 | -0.066 | 4.6E-16 |
| Pelvic-RT | cg01511232 | chr4 | 154,740,776 | 154,740,778 | *LRAT* | 0.187 | (0.020) | 3.4E-15 | 0.107 | 4.2E-16 |
| Pelvic-RT | cg27192248 | chr15 | 64,993,330 | 64,993,332 | *NA* | -0.283 | (0.030) | 3.5E-15 | -0.171 | 1.2E-17 |
| Pelvic-RT | cg08866213 | chr3 | 192,812,987 | 192,812,989 | *MB21D2* | 0.181 | (0.019) | 5.9E-15 | 0.105 | 3.5E-16 |
| Pelvic-RT | cg07086679 | chr7 | 140,316,321 | 140,316,323 | *SLC37A3* | -0.126 | (0.013) | 6.1E-15 | -0.079 | 7.6E-19 |
| Pelvic-RT | cg12960124 | chr4 | 82,100,021 | 82,100,023 | *NA* | -0.146 | (0.016) | 2.1E-14 | -0.090 | 1.5E-17 |
| Pelvic-RT | cg06963130 | chr2 | 235,434,127 | 235,434,129 | *NA* | -0.140 | (0.015) | 2.5E-14 | -0.081 | 2.7E-15 |
| Pelvic-RT | cg17183905 | chr12 | 109,816,036 | 109,816,038 | *TRPV4* | -0.097 | (0.011) | 2.6E-14 | -0.053 | 1.3E-13 |
| Pelvic-RT | cg11994851 | chr11 | 94,767,901 | 94,767,903 | *AMOTL1* | -0.086 | (0.009) | 2.6E-14 | -0.046 | 3.5E-13 |
| Pelvic-RT | cg00973876 | chr17 | 79,925,408 | 79,925,410 | *RP11-353N14.4;RP11-353N14.5* | -0.111 | (0.012) | 3.0E-14 | -0.067 | 2.8E-16 |
| Pelvic-RT | cg08271909 | chr4 | 154,740,538 | 154,740,540 | *LRAT* | 0.149 | (0.016) | 3.2E-14 | 0.082 | 6.3E-14 |
| Pelvic-RT | cg26621020 | chr8 | 122,647,819 | 122,647,821 | *NA* | -0.168 | (0.018) | 3.4E-14 | -0.094 | 3.0E-14 |
| Pelvic-RT | cg15143202 | chr3 | 169,148,736 | 169,148,738 | *MECOM* | -0.157 | (0.017) | 4.9E-14 | -0.090 | 9.2E-15 |
| Pelvic-RT | cg20112376 | chr4 | 6,116,715 | 6,116,717 | *JAKMIP1* | -0.115 | (0.013) | 5.7E-14 | -0.071 | 4.1E-17 |
| Pelvic-RT | cg17200702 | chr11 | 30,992,376 | 30,992,378 | *DCDC1* | -0.164 | (0.018) | 6.9E-14 | -0.097 | 1.7E-15 |
| Pelvic-RT | cg06991974 | chr1 | 3,067,653 | 3,067,655 | *LINC00982* | 0.114 | (0.013) | 7.0E-14 | 0.066 | 5.5E-15 |
| Pelvic-RT | cg12140144 | chr1 | 3,067,710 | 3,067,712 | *LINC00982;PRDM16* | 0.346 | (0.038) | 8.1E-14 | 0.199 | 1.2E-14 |
| Pelvic-RT | cg13286857 | chr5 | 66,822,120 | 66,822,122 | *MAST4* | -0.094 | (0.010) | 9.4E-14 | -0.056 | 1.7E-15 |
| Pelvic-RT | cg18419358 | chr6 | 157,962,976 | 157,962,978 | *NA* | -0.172 | (0.019) | 1.1E-13 | -0.095 | 1.6E-13 |
| Pelvic-RT | cg24765521 | chr9 | 90,296,803 | 90,296,805 | *NA* | -0.178 | (0.020) | 1.4E-13 | -0.104 | 8.3E-15 |
| Pelvic-RT | cg05331731 | chr18 | 11,147,146 | 11,147,148 | *PIEZO2* | 0.204 | (0.023) | 1.9E-13 | 0.116 | 6.3E-14 |
| Pelvic-RT | cg05135521 | chr2 | 160,331,823 | 160,331,825 | *RBMS1* | -0.127 | (0.014) | 2.2E-13 | -0.076 | 2.5E-15 |
| Pelvic-RT | cg12634306 | chr1 | 39,633,138 | 39,633,140 | *HEYL;RP1-144F13.3* | 0.144 | (0.016) | 2.5E-13 | 0.085 | 1.1E-14 |
| Pelvic-RT | cg07946633 | chr1 | 3,067,680 | 3,067,682 | *LINC00982;PRDM16* | 0.093 | (0.011) | 2.9E-13 | 0.055 | 8.6E-15 |
| Pelvic-RT | cg09158878 | chr22 | 46,070,027 | 46,070,029 | *MIRLET7BHG;RP6-109B7.4* | -0.130 | (0.015) | 3.6E-13 | -0.073 | 2.9E-13 |
| Pelvic-RT | cg06576867 | chr22 | 46,070,030 | 46,070,032 | *MIRLET7BHG;RP6-109B7.4* | -0.126 | (0.014) | 4.2E-13 | -0.075 | 1.2E-14 |
| Pelvic-RT | cg08178756 | chr22 | 46,070,024 | 46,070,026 | *MIRLET7BHG;RP6-109B7.4* | -0.149 | (0.017) | 4.8E-13 | -0.088 | 1.1E-14 |
| Pelvic-RT | cg18147181 | chr12 | 52,244,373 | 52,244,375 | *KRT7* | -0.103 | (0.012) | 7.5E-13 | -0.060 | 3.7E-14 |
| Pelvic-RT | cg00218103 | chr22 | 46,070,096 | 46,070,098 | *MIRLET7BHG;RP6-109B7.4* | -0.117 | (0.013) | 8.3E-13 | -0.067 | 2.3E-13 |
| Pelvic-RT | cg02699612 | chr10 | 113,753,520 | 113,753,522 | *PLEKHS1* | -0.191 | (0.022) | 9.9E-13 | -0.113 | 3.2E-14 |
| Pelvic-RT | cg03026982 | chr11 | 19,932,152 | 19,932,154 | *NAV2* | -0.117 | (0.014) | 1.1E-12 | -0.069 | 6.6E-14 |
| Pelvic-RT | cg23776628 | chr1 | 39,633,108 | 39,633,110 | *HEYL;RP1-144F13.3* | 0.136 | (0.016) | 1.1E-12 | 0.081 | 4.0E-14 |
| Pelvic-RT | cg04882213 | chr8 | 60,967,832 | 60,967,834 | *AC022182.3* | -0.154 | (0.018) | 1.2E-12 | -0.090 | 7.6E-14 |
| Pelvic-RT | cg02001279 | chr19 | 940,966 | 940,968 | *ARID3A* | -0.093 | (0.011) | 1.3E-12 | -0.054 | 3.0E-13 |
| Pelvic-RT | cg24769355 | chr9 | 72,041,875 | 72,041,877 | *NA* | -0.152 | (0.018) | 1.3E-12 | -0.086 | 6.0E-13 |
| Pelvic-RT | cg14122922 | chr20 | 44,714,873 | 44,714,875 | *KCNK15-AS1;WISP2* | -0.115 | (0.013) | 1.4E-12 | -0.067 | 1.3E-13 |
| Pelvic-RT | cg08119153 | chr14 | 78,446,957 | 78,446,959 | *NRXN3* | 0.154 | (0.018) | 1.4E-12 | 0.093 | 1.2E-14 |
| Pelvic-RT | cg04188877 | chr22 | 42,721,943 | 42,721,945 | *A4GALT* | 0.093 | (0.011) | 1.5E-12 | 0.059 | 9.1E-16 |
| Pelvic-RT | cg05657694 | chr3 | 100,635,045 | 100,635,047 | *ADGRG7* | -0.166 | (0.020) | 2.1E-12 | -0.091 | 4.0E-12 |
| Pelvic-RT | cg26189283 | chr1 | 155,136,901 | 155,136,903 | *SLC50A1* | -0.116 | (0.014) | 2.2E-12 | -0.067 | 2.5E-13 |
| Pelvic-RT | cg05712639 | chr14 | 52,352,667 | 52,352,669 | *NA* | -0.205 | (0.024) | 2.6E-12 | -0.121 | 1.0E-13 |
| Pelvic-RT | cg10541181 | chr12 | 23,559,658 | 23,559,660 | *SOX5* | -0.180 | (0.021) | 2.7E-12 | -0.108 | 4.6E-14 |
| Pelvic-RT | cg16060790 | chr9 | 101,372,935 | 101,372,937 | *BAAT* | -0.156 | (0.019) | 3.0E-12 | -0.090 | 7.4E-13 |
| Pelvic-RT | cg05944249 | chr8 | 27,636,723 | 27,636,725 | *SCARA3* | 0.162 | (0.019) | 3.1E-12 | 0.096 | 1.4E-13 |
| Pelvic-RT | cg10755723 | chr3 | 192,859,174 | 192,859,176 | *MB21D2* | 0.070 | (0.008) | 3.3E-12 | 0.042 | 9.9E-14 |
| Pelvic-RT | cg13910395 | chr22 | 43,577,140 | 43,577,142 | *EFCAB6* | -0.098 | (0.012) | 4.2E-12 | -0.054 | 1.1E-11 |
| Pelvic-RT | cg21585138 | chr3 | 50,607,674 | 50,607,676 | *CISH* | -0.120 | (0.014) | 4.5E-12 | -0.071 | 1.9E-13 |
| Pelvic-RT | cg07265924 | chr8 | 60,967,910 | 60,967,912 | *AC022182.3* | -0.211 | (0.025) | 5.9E-12 | -0.125 | 2.5E-13 |
| Pelvic-RT | cg19080839 | chr7 | 151,242,505 | 151,242,507 | *SMARCD3* | 0.100 | (0.012) | 6.1E-12 | 0.060 | 1.1E-13 |
| Pelvic-RT | cg10570484 | chr1 | 158,038,015 | 158,038,017 | *KIRREL1* | -0.073 | (0.009) | 6.8E-12 | -0.042 | 2.3E-12 |
| Pelvic-RT | cg23839200 | chr14 | 51,072,092 | 51,072,094 | *TRIM9* | -0.087 | (0.011) | 7.4E-12 | -0.053 | 1.2E-13 |
| Pelvic-RT | cg09126320 | chr8 | 22,920,986 | 22,920,988 | *PEBP4* | -0.082 | (0.010) | 7.8E-12 | -0.046 | 6.2E-12 |
| Pelvic-RT | cg12202498 | chr3 | 15,441,097 | 15,441,099 | *EAF1;EAF1-AS1;METTL6* | -0.076 | (0.009) | 8.3E-12 | -0.042 | 1.7E-11 |
| Pelvic-RT | cg24803517 | chr5 | 95,285,812 | 95,285,814 | *MCTP1* | 0.117 | (0.014) | 9.5E-12 | 0.067 | 3.3E-12 |
| Pelvic-RT | cg13294852 | chr2 | 102,055,667 | 102,055,669 | *NA* | -0.127 | (0.015) | 1.1E-11 | -0.077 | 1.1E-13 |
| Pelvic-RT | cg18598117 | chr19 | 941,125 | 941,127 | *ARID3A* | -0.126 | (0.015) | 1.6E-11 | -0.073 | 2.6E-12 |
| Pelvic-RT | cg17109563 | chr6 | 125,363,279 | 125,363,281 | *NA* | -0.129 | (0.016) | 1.7E-11 | -0.077 | 7.3E-13 |
| Pelvic-RT | cg27589809 | chr3 | 50,612,978 | 50,612,980 | *CISH;MAPKAPK3* | -0.082 | (0.010) | 1.8E-11 | -0.049 | 3.7E-13 |
| Pelvic-RT | cg00062245 | chr17 | 44,384,891 | 44,384,893 | *ITGA2B* | 0.104 | (0.013) | 2.0E-11 | 0.066 | 1.5E-14 |
| Pelvic-RT | cg15251748 | chr7 | 28,953,847 | 28,953,849 | *TRIL* | -0.146 | (0.018) | 2.1E-11 | -0.087 | 9.1E-13 |
| Pelvic-RT | cg17593512 | chr4 | 40,439,495 | 40,439,497 | *RBM47* | -0.144 | (0.018) | 2.4E-11 | -0.085 | 1.7E-12 |
| Pelvic-RT | cg01869007 | chr8 | 60,967,853 | 60,967,855 | *AC022182.3* | -0.178 | (0.022) | 2.4E-11 | -0.106 | 1.2E-12 |
| Pelvic-RT | cg17445936 | chr1 | 3,173,780 | 3,173,782 | *PRDM16* | 0.190 | (0.024) | 2.6E-11 | 0.102 | 1.7E-10 |
| Pelvic-RT | cg25371036 | chr11 | 94,767,582 | 94,767,584 | *AMOTL1* | -0.081 | (0.010) | 3.2E-11 | -0.048 | 1.9E-12 |
| Pelvic-RT | cg14263063 | chr16 | 21,150,859 | 21,150,861 | *DNAH3* | -0.138 | (0.017) | 3.3E-11 | -0.075 | 7.8E-11 |
| Pelvic-RT | cg01063461 | chr14 | 100,156,475 | 100,156,477 | *DEGS2* | -0.056 | (0.007) | 4.1E-11 | -0.030 | 2.1E-10 |
| Pelvic-RT | cg09978533 | chr22 | 46,069,279 | 46,069,281 | *MIRLET7BHG;RP6-109B7.4* | -0.089 | (0.011) | 4.5E-11 | -0.050 | 3.7E-11 |
| Pelvic-RT | cg14143795 | chr16 | 21,150,833 | 21,150,835 | *DNAH3* | -0.148 | (0.019) | 4.6E-11 | -0.078 | 5.1E-10 |
| Pelvic-RT | cg24914621 | chr5 | 146,825,185 | 146,825,187 | *PPP2R2B* | 0.119 | (0.015) | 5.1E-11 | 0.070 | 4.1E-12 |
| Pelvic-RT | cg11414254 | chr5 | 177,022,010 | 177,022,012 | *UIMC1;ZNF346* | -0.118 | (0.015) | 5.4E-11 | -0.069 | 4.4E-12 |
| Pelvic-RT | cg07861288 | chr13 | 91,719,216 | 91,719,218 | *GPC5* | -0.084 | (0.011) | 6.3E-11 | -0.048 | 3.0E-11 |
| Pelvic-RT | cg26408927 | chr3 | 53,725,695 | 53,725,697 | *CACNA1D* | -0.162 | (0.020) | 6.4E-11 | -0.105 | 3.0E-14 |
| Pelvic-RT | cg13806740 | chr17 | 17,971,714 | 17,971,716 | *DRC3;TOM1L2* | -0.067 | (0.008) | 6.7E-11 | -0.038 | 1.8E-11 |
| Pelvic-RT | cg15179400 | chr1 | 15,343,955 | 15,343,957 | *FHAD1;RP3-467K16.2* | -0.100 | (0.013) | 6.8E-11 | -0.060 | 3.5E-12 |
| Pelvic-RT | cg13552692 | chr18 | 68,722,209 | 68,722,211 | *CCDC102B* | -0.117 | (0.015) | 6.9E-11 | -0.069 | 6.0E-12 |
| Pelvic-RT | cg03864568 | chr9 | 76,400,794 | 76,400,796 | *NA* | -0.133 | (0.017) | 7.3E-11 | -0.076 | 2.6E-11 |
| Pelvic-RT | cg17407859 | chr3 | 165,259,995 | 165,259,997 | *LINC01322* | -0.174 | (0.022) | 7.8E-11 | -0.108 | 3.9E-13 |
| Pelvic-RT | cg13866747 | chr13 | 91,313,553 | 91,313,555 | *NA* | -0.161 | (0.021) | 7.8E-11 | -0.094 | 8.3E-12 |
| Pelvic-RT | cg08774778 | chr5 | 62,082,559 | 62,082,561 | *NA* | -0.115 | (0.015) | 9.8E-11 | -0.071 | 5.2E-13 |
| Pelvic-RT | cg03065175 | chr4 | 154,740,605 | 154,740,607 | *LRAT* | 0.120 | (0.015) | 1.0E-10 | 0.070 | 1.8E-11 |
| Pelvic-RT | cg16146033 | chr11 | 62,999,850 | 62,999,852 | *SLC22A8* | -0.105 | (0.013) | 1.1E-10 | -0.059 | 7.6E-11 |
| Pelvic-RT | cg17232357 | chr15 | 66,720,493 | 66,720,495 | *SMAD6* | -0.110 | (0.014) | 1.2E-10 | -0.063 | 3.0E-11 |
| Pelvic-RT | cg02188223 | chr1 | 4,603,220 | 4,603,222 | *NA* | -0.109 | (0.014) | 1.3E-10 | -0.061 | 1.2E-10 |
| Pelvic-RT | cg07784975 | chr12 | 23,360,735 | 23,360,737 | *NA* | -0.186 | (0.024) | 1.7E-10 | -0.110 | 1.2E-11 |
| Pelvic-RT | cg19805775 | chr7 | 82,534,800 | 82,534,802 | *NA* | -0.113 | (0.015) | 2.0E-10 | -0.069 | 3.9E-12 |
| Pelvic-RT | cg25896883 | chr2 | 189,407,943 | 189,407,945 | *NA* | -0.130 | (0.017) | 2.2E-10 | -0.075 | 4.8E-11 |
| Pelvic-RT | cg25719685 | chr3 | 100,635,047 | 100,635,049 | *ADGRG7* | -0.141 | (0.018) | 2.3E-10 | -0.076 | 9.8E-10 |
| Pelvic-RT | cg25790232 | chr16 | 58,215,272 | 58,215,274 | *RP11-459F6.1* | -0.120 | (0.016) | 2.6E-10 | -0.067 | 2.5E-10 |
| Pelvic-RT | cg15246238 | chr7 | 5,595,502 | 5,595,504 | *FSCN1* | -0.102 | (0.013) | 3.2E-10 | -0.060 | 3.9E-11 |
| Pelvic-RT | cg24833462 | chr2 | 108,053,726 | 108,053,728 | *AC023672.2* | -0.192 | (0.025) | 3.8E-10 | -0.115 | 2.0E-11 |
| Pelvic-RT | cg05139637 | chr8 | 60,967,183 | 60,967,185 | *AC022182.3* | -0.151 | (0.020) | 4.0E-10 | -0.086 | 1.9E-10 |
| Pelvic-RT | cg15871086 | chr18 | 58,859,362 | 58,859,364 | *NA* | -0.094 | (0.012) | 4.2E-10 | -0.055 | 5.9E-11 |
| Pelvic-RT | cg14200569 | chr1 | 3,135,360 | 3,135,362 | *PRDM16* | 0.151 | (0.020) | 4.3E-10 | 0.090 | 1.8E-11 |
| Pelvic-RT | cg01530498 | chr22 | 27,475,264 | 27,475,266 | *NA* | -0.091 | (0.012) | 4.7E-10 | -0.053 | 5.6E-11 |
| Pelvic-RT | cg20517202 | chr16 | 21,150,520 | 21,150,522 | *DNAH3* | -0.131 | (0.017) | 4.8E-10 | -0.072 | 7.2E-10 |
| Pelvic-RT | cg13935634 | chr6 | 10,583,535 | 10,583,537 | *GCNT2* | -0.106 | (0.014) | 5.0E-10 | -0.064 | 1.7E-11 |
| Pelvic-RT | cg08587685 | chr10 | 114,632,446 | 114,632,448 | *ABLIM1* | -0.059 | (0.008) | 5.2E-10 | -0.034 | 2.6E-10 |
| Pelvic-RT | cg19743666 | chr2 | 30,148,617 | 30,148,619 | *YPEL5* | -0.141 | (0.019) | 5.3E-10 | -0.081 | 1.9E-10 |
| Pelvic-RT | cg16548911 | chr16 | 50,313,854 | 50,313,856 | *ADCY7;BRD7* | 0.108 | (0.014) | 5.4E-10 | 0.066 | 1.1E-11 |
| Pelvic-RT | cg25325512 | chr6 | 37,174,443 | 37,174,445 | *PIM1* | -0.116 | (0.016) | 6.7E-10 | -0.070 | 3.7E-11 |
| Pelvic-RT | cg22234488 | chr2 | 105,179,803 | 105,179,805 | *NA* | -0.105 | (0.014) | 6.7E-10 | -0.060 | 1.8E-10 |
| Pelvic-RT | cg01641136 | chr3 | 50,607,810 | 50,607,812 | *CISH* | -0.110 | (0.015) | 6.9E-10 | -0.065 | 6.7E-11 |
| Pelvic-RT | cg13442016 | chr10 | 102,436,581 | 102,436,583 | *MIR146B* | -0.112 | (0.015) | 7.3E-10 | -0.069 | 9.3E-12 |
| Pelvic-RT | cg08101174 | chr10 | 102,436,783 | 102,436,785 | *NA* | -0.088 | (0.012) | 7.6E-10 | -0.056 | 1.8E-12 |
| Pelvic-RT | cg16902294 | chr4 | 154,740,779 | 154,740,781 | *LRAT* | 0.105 | (0.014) | 8.0E-10 | 0.060 | 4.1E-10 |
| Pelvic-RT | cg05478824 | chr17 | 82,012,258 | 82,012,260 | *ASPSCR1* | 0.089 | (0.012) | 8.9E-10 | 0.051 | 3.8E-10 |
| Pelvic-RT | cg15491247 | chr1 | 3,313,859 | 3,313,861 | *PRDM16* | -0.091 | (0.012) | 9.7E-10 | -0.048 | 7.9E-09 |
| Pelvic-RT | cg13903421 | chr2 | 218,873,991 | 218,873,993 | *WNT6* | 0.200 | (0.027) | 9.8E-10 | 0.128 | 1.9E-12 |
| Pelvic-RT | cg13924715 | chr11 | 10,729,342 | 10,729,344 | *NA* | 0.189 | (0.026) | 1.0E-09 | 0.111 | 1.3E-10 |
| Pelvic-RT | cg14395060 | chr22 | 32,045,182 | 32,045,184 | *SLC5A1* | -0.113 | (0.015) | 1.1E-09 | -0.071 | 7.5E-12 |
| Pelvic-RT | cg25695450 | chr7 | 41,219,438 | 41,219,440 | *NA* | -0.101 | (0.014) | 1.2E-09 | -0.059 | 2.3E-10 |
| Pelvic-RT | cg07501635 | chr3 | 53,680,140 | 53,680,142 | *CACNA1D* | 0.201 | (0.027) | 1.3E-09 | 0.113 | 9.9E-10 |
| Pelvic-RT | cg01446044 | chr1 | 13,624,516 | 13,624,518 | *AL359771.1;RNA5SP41* | -0.059 | (0.008) | 1.3E-09 | -0.034 | 1.9E-10 |
| Pelvic-RT | cg21025494 | chr7 | 23,463,124 | 23,463,126 | *IGF2BP3* | -0.079 | (0.011) | 1.3E-09 | -0.046 | 2.9E-10 |
| Pelvic-RT | cg03479114 | chr4 | 165,359,572 | 165,359,574 | *NA* | -0.114 | (0.016) | 1.4E-09 | -0.073 | 4.4E-12 |
| Pelvic-RT | cg15890546 | chr3 | 194,967,758 | 194,967,760 | *NA* | -0.086 | (0.012) | 1.4E-09 | -0.049 | 5.4E-10 |
| Pelvic-RT | cg02000109 | chr20 | 38,357,665 | 38,357,667 | *LBP* | -0.132 | (0.018) | 1.4E-09 | -0.075 | 6.4E-10 |
| Pelvic-RT | cg08662753 | chr9 | 134,669,653 | 134,669,655 | *COL5A1* | 0.095 | (0.013) | 1.5E-09 | 0.054 | 8.4E-10 |
| Pelvic-RT | cg05848650 | chr3 | 192,893,050 | 192,893,052 | *MB21D2* | -0.150 | (0.021) | 1.5E-09 | -0.087 | 2.8E-10 |
| Pelvic-RT | cg19283806 | chr18 | 68,722,182 | 68,722,184 | *CCDC102B* | -0.084 | (0.012) | 1.7E-09 | -0.049 | 4.3E-10 |
| Pelvic-RT | cg15857661 | chr10 | 102,436,485 | 102,436,487 | *MIR146B* | -0.106 | (0.015) | 1.8E-09 | -0.066 | 1.8E-11 |
| Pelvic-RT | cg06586734 | chr4 | 61,143,607 | 61,143,609 | *RP11-16N2.1* | -0.119 | (0.016) | 1.8E-09 | -0.066 | 1.7E-09 |
| Pelvic-RT | cg10202782 | chr9 | 101,372,961 | 101,372,963 | *BAAT* | -0.122 | (0.017) | 1.8E-09 | -0.073 | 1.1E-10 |
| Pelvic-RT | cg08573679 | chr6 | 125,363,363 | 125,363,365 | *NA* | -0.121 | (0.017) | 1.8E-09 | -0.072 | 1.1E-10 |
| Pelvic-RT | cg15572968 | chr20 | 2,525,549 | 2,525,551 | *ZNF343* | -0.062 | (0.008) | 1.9E-09 | -0.035 | 6.4E-10 |
| Pelvic-RT | cg09992085 | chr19 | 41,456,428 | 41,456,430 | *PCAT19* | -0.101 | (0.014) | 1.9E-09 | -0.061 | 9.2E-11 |
| Pelvic-RT | cg05697274 | chr1 | 230,279,630 | 230,279,632 | *GALNT2* | 0.121 | (0.017) | 1.9E-09 | 0.066 | 5.5E-09 |
| Pelvic-RT | cg23205858 | chr22 | 20,734,517 | 20,734,519 | *NA* | 0.109 | (0.015) | 2.0E-09 | 0.062 | 7.0E-10 |
| Pelvic-RT | cg21242613 | chr1 | 15,344,049 | 15,344,051 | *FHAD1;RP3-467K16.2* | -0.075 | (0.010) | 2.0E-09 | -0.046 | 6.8E-11 |
| Pelvic-RT | cg01004017 | chr2 | 120,899,770 | 120,899,772 | *GLI2* | 0.121 | (0.017) | 2.3E-09 | 0.072 | 2.0E-10 |
| Pelvic-RT | cg06269255 | chr17 | 44,269,426 | 44,269,428 | *SLC4A1* | -0.067 | (0.009) | 2.5E-09 | -0.040 | 1.6E-10 |
| Pelvic-RT | cg23135545 | chr15 | 101,024,297 | 101,024,299 | *LRRK1* | 0.053 | (0.007) | 2.5E-09 | 0.031 | 8.0E-10 |
| Pelvic-RT | cg07363855 | chr1 | 3,067,905 | 3,067,907 | *LINC00982;PRDM16* | 0.073 | (0.010) | 2.6E-09 | 0.040 | 7.0E-09 |
| Pelvic-RT | cg25987136 | chr1 | 3,367,668 | 3,367,670 | *PRDM16* | -0.068 | (0.009) | 2.6E-09 | -0.036 | 1.3E-08 |
| Pelvic-RT | cg04697265 | chr11 | 130,813,804 | 130,813,806 | *NA* | -0.083 | (0.012) | 2.8E-09 | -0.050 | 9.9E-11 |
| Pelvic-RT | cg02606535 | chr16 | 2,083,030 | 2,083,032 | *TSC2* | 0.065 | (0.009) | 3.1E-09 | 0.035 | 7.8E-09 |
| Pelvic-RT | cg10762064 | chr3 | 194,967,685 | 194,967,687 | *NA* | -0.095 | (0.013) | 3.1E-09 | -0.056 | 4.1E-10 |
| Pelvic-RT | cg27478635 | chr20 | 41,003,796 | 41,003,798 | *NA* | -0.151 | (0.021) | 3.1E-09 | -0.096 | 1.3E-11 |
| Pelvic-RT | cg08838779 | chr8 | 93,398,777 | 93,398,779 | *LINC00535* | 0.085 | (0.012) | 3.4E-09 | 0.047 | 5.0E-09 |
| Pelvic-RT | cg21302696 | chr14 | 94,390,773 | 94,390,775 | *SERPINA1* | -0.074 | (0.010) | 3.5E-09 | -0.044 | 2.1E-10 |
| Pelvic-RT | cg19075225 | chr2 | 241,070,097 | 241,070,099 | *SNED1* | 0.127 | (0.018) | 3.9E-09 | 0.069 | 7.7E-09 |
| Pelvic-RT | cg09539785 | chr22 | 24,729,643 | 24,729,645 | *PIWIL3* | -0.092 | (0.013) | 4.0E-09 | -0.054 | 4.7E-10 |
| Pelvic-RT | cg17783244 | chr22 | 31,342,562 | 31,342,564 | *PATZ1* | 0.092 | (0.013) | 4.7E-09 | 0.051 | 6.2E-09 |
| Pelvic-RT | cg26910511 | chr19 | 39,308,396 | 39,308,398 | *LRFN1* | 0.084 | (0.012) | 4.8E-09 | 0.048 | 2.3E-09 |
| Pelvic-RT | cg09835575 | chr20 | 9,985,215 | 9,985,217 | *ANKEF1;RP5-839B4.8* | -0.115 | (0.016) | 5.0E-09 | -0.067 | 1.4E-09 |
| Pelvic-RT | cg09519959 | chr17 | 28,643,897 | 28,643,899 | *KIAA0100* | -0.086 | (0.012) | 5.2E-09 | -0.053 | 6.8E-11 |
| Pelvic-RT | cg25241559 | chr2 | 241,049,961 | 241,049,963 | *AC005237.4;SNED1* | 0.067 | (0.010) | 5.3E-09 | 0.037 | 8.7E-09 |
| Pelvic-RT | cg14436379 | chr3 | 122,245,755 | 122,245,757 | *CASR* | -0.130 | (0.018) | 5.3E-09 | -0.077 | 7.2E-10 |
| Pelvic-RT | cg22872396 | chr2 | 166,366,162 | 166,366,164 | *SCN9A* | -0.126 | (0.018) | 5.4E-09 | -0.076 | 2.9E-10 |
| Pelvic-RT | cg16983486 | chr11 | 8,832,696 | 8,832,698 | *ST5* | -0.057 | (0.008) | 5.4E-09 | -0.035 | 1.5E-10 |
| Pelvic-RT | cg21781784 | chr1 | 227,437,757 | 227,437,759 | *NA* | -0.128 | (0.018) | 5.6E-09 | -0.078 | 1.6E-10 |
| Pelvic-RT | cg03054277 | chr1 | 228,212,515 | 228,212,517 | *OBSCN;OBSCN-AS1* | 0.078 | (0.011) | 5.9E-09 | 0.046 | 1.2E-09 |
| Pelvic-RT | cg18603250 | chr11 | 94,767,885 | 94,767,887 | *AMOTL1* | -0.071 | (0.010) | 6.0E-09 | -0.040 | 3.0E-09 |
| Pelvic-RT | cg00589617 | chr1 | 230,279,596 | 230,279,598 | *GALNT2* | 0.140 | (0.020) | 6.1E-09 | 0.074 | 4.7E-08 |
| Pelvic-RT | cg22156456 | chr17 | 41,687,986 | 41,687,988 | *EIF1* | -0.073 | (0.010) | 6.2E-09 | -0.043 | 6.7E-10 |
| Pelvic-RT | cg03670162 | chr12 | 107,903,561 | 107,903,563 | *RP11-554D14.2* | -0.102 | (0.015) | 6.8E-09 | -0.056 | 1.5E-08 |
| Pelvic-RT | cg05186879 | chr3 | 50,612,890 | 50,612,892 | *CISH;MAPKAPK3* | -0.067 | (0.010) | 7.2E-09 | -0.038 | 3.9E-09 |
| Pelvic-RT | cg06535121 | chr4 | 26,088,488 | 26,088,490 | *NA* | -0.074 | (0.011) | 7.4E-09 | -0.042 | 3.9E-09 |
| Pelvic-RT | cg08909363 | chr11 | 72,181,494 | 72,181,496 | *RP11-807H22.7* | 0.063 | (0.009) | 8.3E-09 | 0.037 | 2.1E-09 |
| Pelvic-RT | cg16198075 | chr4 | 6,417,112 | 6,417,114 | *PPP2R2C* | 0.114 | (0.016) | 8.7E-09 | 0.067 | 1.0E-09 |
| Pelvic-RT | cg02757819 | chr3 | 172,581,715 | 172,581,717 | *RP11-408H1.3* | -0.075 | (0.011) | 9.1E-09 | -0.042 | 9.7E-09 |
| Pelvic-RT | cg12569593 | chr11 | 126,543,820 | 126,543,822 | *KIRREL3;KIRREL3-AS1* | -0.062 | (0.009) | 9.4E-09 | -0.037 | 1.3E-09 |
| Pelvic-RT | cg13474360 | chr3 | 192,885,619 | 192,885,621 | *MB21D2* | 0.074 | (0.011) | 9.4E-09 | 0.045 | 4.8E-10 |
| Pelvic-RT | cg12530994 | chr10 | 5,094,589 | 5,094,591 | *AKR1C1;AKR1C2;AKR1C3;SNORD118* | -0.114 | (0.017) | 9.7E-09 | -0.063 | 1.9E-08 |
| Pelvic-RT | cg03129600 | chr2 | 51,028,939 | 51,028,941 | *NRXN1* | 0.073 | (0.011) | 1.2E-08 | 0.044 | 7.8E-10 |
| Pelvic-RT | cg01693697 | chr15 | 73,920,554 | 73,920,556 | *LOXL1-AS1* | -0.117 | (0.017) | 1.2E-08 | -0.067 | 4.8E-09 |
| Pelvic-RT | cg16394551 | chr1 | 3,313,792 | 3,313,794 | *PRDM16* | -0.093 | (0.013) | 1.2E-08 | -0.049 | 6.6E-08 |
| Pelvic-RT | cg12009405 | chr6 | 148,330,194 | 148,330,196 | *SASH1* | -0.151 | (0.022) | 1.2E-08 | -0.093 | 2.4E-10 |
| Pelvic-RT | cg21385305 | chr2 | 23,490,082 | 23,490,084 | *KLHL29* | -0.100 | (0.014) | 1.3E-08 | -0.059 | 1.6E-09 |
| Pelvic-RT | cg20970886 | chr3 | 192,850,924 | 192,850,926 | *MB21D2* | -0.085 | (0.012) | 1.3E-08 | -0.048 | 1.2E-08 |
| Pelvic-RT | cg13329407 | chr21 | 24,429,059 | 24,429,061 | *AP000476.1* | -0.180 | (0.026) | 1.5E-08 | -0.106 | 2.0E-09 |
| Pelvic-RT | cg05449815 | chr17 | 3,640,570 | 3,640,572 | *CTNS* | -0.084 | (0.012) | 1.5E-08 | -0.052 | 4.4E-10 |
| Pelvic-RT | cg26066589 | chr16 | 55,996,381 | 55,996,383 | *NA* | -0.080 | (0.012) | 1.7E-08 | -0.047 | 2.1E-09 |
| Pelvic-RT | cg05483571 | chr17 | 79,927,424 | 79,927,426 | *RP11-353N14.5* | -0.109 | (0.016) | 1.8E-08 | -0.062 | 8.6E-09 |
| Pelvic-RT | cg12307404 | chr3 | 186,552,703 | 186,552,705 | *TBCCD1* | -0.067 | (0.010) | 1.9E-08 | -0.039 | 4.1E-09 |
| Pelvic-RT | cg19152802 | chr5 | 110,514,185 | 110,514,187 | *MIR548F3;TMEM232* | -0.113 | (0.017) | 2.0E-08 | -0.071 | 2.1E-10 |
| Pelvic-RT | cg05512561 | chr13 | 33,351,518 | 33,351,520 | *STARD13* | -0.084 | (0.012) | 2.1E-08 | -0.051 | 1.1E-09 |
| Pelvic-RT | cg01543150 | chr3 | 192,916,271 | 192,916,273 | *MB21D2* | 0.094 | (0.014) | 2.2E-08 | 0.053 | 1.6E-08 |
| Pelvic-RT | cg21771773 | chr5 | 164,296,864 | 164,296,866 | *CTC-340A15.2* | 0.144 | (0.021) | 2.3E-08 | 0.083 | 7.5E-09 |
| Pelvic-RT | cg01821042 | chr13 | 51,995,789 | 51,995,791 | *ATP7B* | -0.058 | (0.009) | 2.3E-08 | -0.032 | 2.3E-08 |
| Pelvic-RT | cg04178438 | chr7 | 157,300,377 | 157,300,379 | *NA* | 0.044 | (0.007) | 2.3E-08 | 0.023 | 1.3E-07 |
| Pelvic-RT | cg12940522 | chr20 | 63,441,799 | 63,441,801 | *KCNQ2* | -0.056 | (0.008) | 2.4E-08 | -0.030 | 7.8E-08 |
| Pelvic-RT | cg03868978 | chr21 | 41,165,366 | 41,165,368 | *NA* | -0.074 | (0.011) | 2.5E-08 | -0.042 | 1.1E-08 |
| Pelvic-RT | cg02390408 | chr1 | 47,521,608 | 47,521,610 | *NA* | -0.061 | (0.009) | 2.5E-08 | -0.032 | 1.5E-07 |
| Pelvic-RT | cg16985233 | chr15 | 43,514,050 | 43,514,052 | *MAP1A* | -0.112 | (0.017) | 2.6E-08 | -0.070 | 4.1E-10 |
| Pelvic-RT | cg17850049 | chr1 | 15,344,019 | 15,344,021 | *FHAD1;RP3-467K16.2* | -0.086 | (0.013) | 2.7E-08 | -0.051 | 3.2E-09 |
| Pelvic-RT | cg11144555 | chr5 | 164,296,927 | 164,296,929 | *CTC-340A15.2* | 0.135 | (0.020) | 2.7E-08 | 0.078 | 8.1E-09 |
| Pelvic-RT | cg13511623 | chr9 | 123,716,678 | 123,716,680 | *DENND1A* | -0.135 | (0.020) | 2.7E-08 | -0.081 | 1.9E-09 |
| Pelvic-RT | cg17254414 | chr14 | 103,881,057 | 103,881,059 | *CTD-2134A5.4* | -0.053 | (0.008) | 2.8E-08 | -0.031 | 3.4E-09 |
| Pelvic-RT | cg14292870 | chr12 | 6,778,376 | 6,778,378 | *LAG3* | 0.107 | (0.016) | 2.8E-08 | 0.063 | 5.9E-09 |
| Pelvic-RT | cg12769615 | chr4 | 94,732,004 | 94,732,006 | *NA* | -0.087 | (0.013) | 2.9E-08 | -0.050 | 8.0E-09 |
| Pelvic-RT | cg17287921 | chr2 | 45,964,630 | 45,964,632 | *PRKCE* | -0.049 | (0.007) | 3.0E-08 | -0.029 | 5.9E-09 |
| Pelvic-RT | cg01838523 | chr20 | 44,714,648 | 44,714,650 | *KCNK15-AS1;WISP2* | -0.068 | (0.010) | 3.2E-08 | -0.039 | 8.4E-09 |
| Pelvic-RT | cg21411447 | chr9 | 16,262,654 | 16,262,656 | *C9orf92* | -0.097 | (0.015) | 3.2E-08 | -0.052 | 1.1E-07 |
| Pelvic-RT | cg02330078 | chr5 | 164,277,917 | 164,277,919 | *NA* | 0.101 | (0.015) | 3.4E-08 | 0.056 | 4.1E-08 |
| Pelvic-RT | cg10358466 | chr3 | 169,240,939 | 169,240,941 | *MECOM* | -0.095 | (0.014) | 3.4E-08 | -0.054 | 1.7E-08 |
| Pelvic-RT | cg08903740 | chr1 | 162,317,927 | 162,317,929 | *NOS1AP;RP11-565P22.2* | 0.077 | (0.012) | 3.4E-08 | 0.049 | 3.1E-10 |
| Pelvic-RT | cg00494337 | chr20 | 31,606,220 | 31,606,222 | *ID1;MIR3193* | -0.078 | (0.012) | 3.4E-08 | -0.045 | 1.5E-08 |
| Pelvic-RT | cg09317107 | chr1 | 56,788,784 | 56,788,786 | *FYB2* | -0.066 | (0.010) | 3.6E-08 | -0.036 | 5.8E-08 |
| Pelvic-RT | cg01435315 | chr10 | 70,588,663 | 70,588,665 | *NA* | -0.111 | (0.017) | 3.7E-08 | -0.068 | 1.9E-09 |
| Pelvic-RT | cg18230175 | chr3 | 192,802,836 | 192,802,838 | *MB21D2* | 0.094 | (0.014) | 3.7E-08 | 0.053 | 3.4E-08 |
| Pelvic-RT | cg06180200 | chr19 | 38,912,295 | 38,912,297 | *CCER2* | 0.089 | (0.013) | 3.8E-08 | 0.056 | 3.6E-10 |
| Pelvic-RT | cg05726109 | chr22 | 19,722,231 | 19,722,233 | *GP1BB;SEPT5* | 0.131 | (0.020) | 3.8E-08 | 0.073 | 3.6E-08 |
| Pelvic-RT | cg26936966 | chr8 | 135,599,881 | 135,599,883 | *KHDRBS3* | 0.069 | (0.010) | 3.8E-08 | 0.040 | 9.3E-09 |
| Pelvic-RT | cg03181246 | chr20 | 43,243,756 | 43,243,758 | *NA* | -0.114 | (0.017) | 4.0E-08 | -0.071 | 9.9E-10 |
| Pelvic-RT | cg12195314 | chr19 | 2,491,256 | 2,491,258 | *NA* | -0.062 | (0.009) | 4.1E-08 | -0.036 | 2.2E-08 |
| Pelvic-RT | cg06728103 | chr12 | 93,536,922 | 93,536,924 | *NA* | -0.078 | (0.012) | 4.2E-08 | -0.045 | 1.7E-08 |
| Pelvic-RT | cg15688339 | chr5 | 164,296,732 | 164,296,734 | *CTC-340A15.2* | 0.164 | (0.025) | 4.3E-08 | 0.092 | 3.8E-08 |
| Pelvic-RT | cg04948230 | chr19 | 38,912,205 | 38,912,207 | *CCER2* | 0.125 | (0.019) | 4.4E-08 | 0.078 | 8.2E-10 |
| Pelvic-RT | cg02276831 | chr9 | 35,406,119 | 35,406,121 | *ATP8B5P* | 0.072 | (0.011) | 5.1E-08 | 0.043 | 8.8E-09 |
| Pelvic-RT | cg00008629 | chr9 | 112,331,380 | 112,331,382 | *PTBP3* | -0.140 | (0.021) | 5.1E-08 | -0.082 | 9.6E-09 |
| Pelvic-RT | cg16396718 | chr21 | 26,840,493 | 26,840,495 | *ADAMTS1* | -0.096 | (0.015) | 5.1E-08 | -0.048 | 8.8E-07 |
| Pelvic-RT | cg06268875 | chr18 | 11,147,385 | 11,147,387 | *PIEZO2* | 0.114 | (0.017) | 5.3E-08 | 0.066 | 1.2E-08 |
| Pelvic-RT | cg17342738 | chr2 | 109,265,641 | 109,265,643 | *SH3RF3* | -0.058 | (0.009) | 5.4E-08 | -0.036 | 1.5E-09 |
| Pelvic-RT | cg25788793 | chr4 | 10,032,955 | 10,032,957 | *SLC2A9* | -0.074 | (0.011) | 5.4E-08 | -0.046 | 1.6E-09 |
| Pelvic-RT | cg18880175 | chr2 | 114,543,268 | 114,543,270 | *DPP10* | -0.115 | (0.018) | 5.5E-08 | -0.068 | 1.1E-08 |
| Pelvic-RT | cg03193328 | chr7 | 66,281,864 | 66,281,866 | *TPST1* | -0.099 | (0.015) | 5.6E-08 | -0.058 | 1.1E-08 |
| Pelvic-RT | cg26663696 | chr3 | 160,403,486 | 160,403,488 | *MIR15B;MIR16-2;RP11-432B6.3;SMC4* | -0.127 | (0.019) | 5.7E-08 | -0.080 | 1.1E-09 |
| Pelvic-RT | cg02541477 | chr13 | 52,061,901 | 52,061,903 | *NEK5* | -0.151 | (0.023) | 5.7E-08 | -0.091 | 4.1E-09 |
| Pelvic-RT | cg25556035 | chr19 | 13,017,058 | 13,017,060 | *NFIX* | 0.071 | (0.011) | 5.8E-08 | 0.039 | 5.1E-08 |
| Pelvic-RT | cg02045948 | chr3 | 32,815,824 | 32,815,826 | *NA* | -0.082 | (0.013) | 6.0E-08 | -0.048 | 9.5E-09 |
| Pelvic-RT | cg14879089 | chr4 | 5,827,008 | 5,827,010 | *CRMP1* | -0.104 | (0.016) | 6.2E-08 | -0.060 | 1.9E-08 |
| Pelvic-RT | cg23258188 | chr1 | 3,159,281 | 3,159,283 | *PRDM16* | 0.047 | (0.007) | 6.5E-08 | 0.027 | 4.5E-08 |
| Pelvic-RT | cg06855546 | chr16 | 1,488,824 | 1,488,826 | *PTX4* | 0.067 | (0.010) | 6.6E-08 | 0.037 | 1.0E-07 |
| Pelvic-RT | cg17107472 | chr3 | 50,574,267 | 50,574,269 | *HEMK1* | -0.045 | (0.007) | 6.7E-08 | -0.025 | 6.4E-08 |
| Pelvic-RT | cg12597694 | chr16 | 8,183,208 | 8,183,210 | *NA* | -0.136 | (0.021) | 6.7E-08 | -0.078 | 2.4E-08 |
| Pelvic-RT | cg16848289 | chr5 | 164,296,714 | 164,296,716 | *CTC-340A15.2* | 0.144 | (0.022) | 6.8E-08 | 0.082 | 3.5E-08 |
| Pelvic-RT | cg04585694 | chr6 | 125,363,713 | 125,363,715 | *NA* | -0.157 | (0.024) | 6.8E-08 | -0.086 | 1.4E-07 |
| Pelvic-RT | cg13152952 | chr8 | 41,713,035 | 41,713,037 | *ANK1* | -0.093 | (0.014) | 6.8E-08 | -0.050 | 1.7E-07 |
| Pelvic-RT | cg00303773 | chr17 | 17,849,260 | 17,849,262 | *TOM1L2* | -0.151 | (0.023) | 6.9E-08 | -0.093 | 3.0E-09 |
| Pelvic-RT | cg25840926 | chr2 | 20,448,225 | 20,448,227 | *RHOB* | 0.107 | (0.016) | 7.1E-08 | 0.059 | 1.1E-07 |
| Pelvic-RT | cg12713583 | chr19 | 940,723 | 940,725 | *ARID3A* | -0.132 | (0.020) | 7.2E-08 | -0.074 | 7.0E-08 |
| Pelvic-RT | cg12351310 | chr5 | 53,642,161 | 53,642,163 | *NDUFS4* | -0.080 | (0.012) | 7.2E-08 | -0.047 | 1.2E-08 |
| Pelvic-RT | cg12800962 | chr20 | 50,009,899 | 50,009,901 | *NA* | 0.094 | (0.015) | 7.9E-08 | 0.058 | 3.8E-09 |
| Pelvic-RT | cg04401758 | chr22 | 41,402,170 | 41,402,172 | *NA* | -0.063 | (0.010) | 7.9E-08 | -0.037 | 1.3E-08 |
| Pelvic-RT | cg26964651 | chr5 | 78,653,253 | 78,653,255 | *LHFPL2* | -0.108 | (0.017) | 8.2E-08 | -0.068 | 1.1E-09 |
| Pelvic-RT | cg09762316 | chr9 | 70,043,437 | 70,043,439 | *MAMDC2;MAMDC2-AS1* | 0.078 | (0.012) | 8.3E-08 | 0.046 | 1.7E-08 |
| Pelvic-RT | cg05463027 | chr6 | 17,988,516 | 17,988,518 | *KIF13A* | -0.094 | (0.015) | 8.7E-08 | -0.056 | 1.1E-08 |
| Pelvic-RT | cg03485674 | chr16 | 50,313,983 | 50,313,985 | *ADCY7;BRD7* | 0.052 | (0.008) | 8.8E-08 | 0.031 | 1.3E-08 |

| **Table S3. 276 additional CpG hits associated with 9 paired combinations of treatments (*P*<9x10^-8^)** | | | | | | | |  |
| --- | --- | --- | --- | --- | --- | --- | --- | --- |
| Treatment pair | CpG | Chromosome | Start position | End position | HGNC gene | Coefficient | (SE) | *P* |
| Abdomen-RT and pelvis-RT | cg10493186 | chr1 | 3218191 | 3218193 | *PRDM16* | 0.086 | (0.01) | 5.23E-08 |
| Abdomen-RT and pelvis-RT | cg15015917 | chr19 | 38912182 | 38912184 | *CCER2* | 0.076 | (0.01) | 7.78E-08 |
| Abdomen-RT and pelvis-RT | cg00985388 | chr3 | 184234423 | 184234425 | *EIF2B5;VWA5B2* | -0.079 | (0.01) | 4.04E-08 |
| Abdomen-RT and pelvis-RT | cg23954423 | chr6 | 46323478 | 46323480 | *RCAN2* | -0.077 | (0.01) | 7.92E-08 |
| Abdomen-RT and pelvis-RT | cg22815707 | chr18 | 9138607 | 9138609 | *ANKRD12;RP11-21J18.1* | -0.142 | (0.02) | 5.54E-08 |
| Abdomen-RT and pelvis-RT | cg13301454 | chr5 | 146620729 | 146620731 | *PPP2R2B* | 0.051 | (0.01) | 8.89E-08 |
| Abdomen-RT and pelvis-RT | cg04239194 | chr6 | 134981230 | 134981232 | *HBS1L* | 0.155 | (0.02) | 4.77E-08 |
| Abdomen-RT and pelvis-RT | cg03216822 | chr11 | 23805197 | 23805199 | *RP11-945A11.2* | -0.099 | (0.01) | 4.65E-08 |
| Abdomen-RT and pelvis-RT | cg04201727 | chr9 | 122228929 | 122228931 | *LHX6* | 0.318 | (0.05) | 5.58E-08 |
| Abdomen-RT and pelvis-RT | cg09223475 | chr1 | 15344125 | 15344127 | *FHAD1;RP3-467K16.2* | -0.086 | (0.01) | 4.72E-08 |
| Abdomen-RT and pelvis-RT | cg12393272 | chr17 | 4580527 | 4580529 | *NA* | -0.049 | (0.01) | 7.89E-08 |
| Abdomen-RT and pelvis-RT | cg19936380 | chr10 | 114632356 | 114632358 | *ABLIM1* | -0.096 | (0.01) | 5.51E-08 |
| Abdomen-RT and pelvis-RT | cg16733993 | chr4 | 76948498 | 76948500 | *SEPT11* | 0.085 | (0.01) | 5.72E-08 |
| Abdomen-RT and pelvis-RT | cg14844584 | chr20 | 49230994 | 49230996 | *DDX27* | 0.055 | (0.01) | 6.66E-08 |
| Abdomen-RT and pelvis-RT | cg06347782 | chr9 | 122228902 | 122228904 | *LHX6* | 0.367 | (0.05) | 6.57E-08 |
| Abdomen-RT and pelvis-RT | cg01012330 | chr3 | 192916269 | 192916271 | *MB21D2* | 0.087 | (0.01) | 8.00E-08 |
| Abdomen-RT and pelvis-RT | cg21150701 | chr2 | 120899241 | 120899243 | *GLI2* | 0.081 | (0.01) | 8.72E-08 |
| Abdomen-RT and pelvis-RT | cg13959831 | chr9 | 36938343 | 36938345 | *PAX5* | -0.107 | (0.02) | 4.83E-08 |
| Abdomen-RT and pelvis-RT | cg07472795 | chr10 | 29860326 | 29860328 | *NA* | -0.072 | (0.01) | 2.07E-08 |
| Chest-RT and pelvis-RT | cg13301454 | chr5 | 146620729 | 146620731 | *PPP2R2B* | 0.055 | (0.01) | 8.79E-08 |
| Chest-RT and pelvis-RT | cg00985388 | chr3 | 184234423 | 184234425 | *EIF2B5;VWA5B2* | -0.089 | (0.01) | 4.95E-09 |
| Chest-RT and pelvis-RT | cg21734651 | chr10 | 74145284 | 74145286 | *AP3M1* | -0.158 | (0.02) | 2.66E-08 |
| Chest-RT and pelvis-RT | cg22815707 | chr18 | 9138607 | 9138609 | *ANKRD12;RP11-21J18.1* | -0.167 | (0.02) | 1.21E-09 |
| Chest-RT and pelvis-RT | cg07936137 | chr11 | 63902658 | 63902660 | *MARK2* | 0.085 | (0.01) | 3.84E-08 |
| Chest-RT and pelvis-RT | cg15167811 | chr9 | 112331777 | 112331779 | *PTBP3* | -0.166 | (0.02) | 4.09E-08 |
| Chest-RT and pelvis-RT | cg16733993 | chr4 | 76948498 | 76948500 | *SETP11* | 0.091 | (0.01) | 5.63E-08 |
| Chest-RT and pelvis-RT | cg04567724 | chr8 | 138680241 | 138680243 | *COL22A1* | -0.122 | (0.02) | 5.11E-08 |
| Chest-RT and pelvis-RT | cg12022558 | chr2 | 205245819 | 205245821 | *PARD3B* | -0.161 | (0.02) | 1.26E-08 |
| Chest-RT and pelvis-RT | cg10687107 | chr8 | 66542431 | 66542433 | *NA* | 0.135 | (0.02) | 6.93E-08 |
| Chest-RT and pelvis-RT | cg04313338 | chr16 | 55701881 | 55701883 | *SLC6A2* | -0.060 | (0.01) | 3.34E-08 |
| Chest-RT and pelvis-RT | cg23954423 | chr6 | 46323478 | 46323480 | *RCAN2* | -0.083 | (0.01) | 3.93E-08 |
| Chest-RT and pelvis-RT | cg22982242 | chr2 | 108069673 | 108069675 | *NA* | 0.089 | (0.01) | 2.52E-08 |
| Chest-RT and pelvis-RT | cg03216822 | chr11 | 23805197 | 23805199 | *RP11-945A11.2* | -0.109 | (0.02) | 1.14E-08 |
| Chest-RT and pelvis-RT | cg22108243 | chr1 | 68725462 | 68725464 | *NA* | -0.152 | (0.02) | 4.96E-08 |
| Chest-RT and pelvis-RT | cg16944069 | chr3 | 169582756 | 169582758 | *MECOM* | 0.090 | (0.01) | 6.13E-08 |
| Chest-RT and pelvis-RT | cg21207019 | chr22 | 37727878 | 37727880 | *RP1-37E16.12;TRIOBP* | -0.135 | (0.02) | 4.74E-08 |
| Chest-RT and pelvis-RT | cg03984209 | chr3 | 55885989 | 55885991 | *ERC2* | -0.115 | (0.02) | 2.35E-08 |
| Chest-RT and pelvis-RT | cg22948745 | chr8 | 138688051 | 138688053 | *COL22A1* | -0.132 | (0.02) | 1.34E-08 |
| Chest-RT and pelvis-RT | cg07472795 | chr10 | 29860326 | 29860328 | *NA* | -0.076 | (0.01) | 1.65E-08 |
| Chest-RT and pelvis-RT | cg09223475 | chr1 | 15344125 | 15344127 | *FHAD1;RP3-467K16.2* | -0.095 | (0.01) | 1.51E-08 |
| Chest-RT and pelvis-RT | cg19936380 | chr10 | 114632356 | 114632358 | *ABLIM1* | -0.102 | (0.01) | 3.70E-08 |
| Chest-RT and pelvis-RT | cg13930000 | chr5 | 84451826 | 84451828 | *CTD-2269F5.1* | -0.088 | (0.01) | 8.95E-08 |
| Chest-RT and pelvis-RT | cg24618605 | chr18 | 31437150 | 31437152 | *NA* | -0.080 | (0.01) | 7.55E-08 |
| Chest-RT and pelvis-RT | cg12049992 | chr18 | 11147785 | 11147787 | *PIEZO2* | 0.160 | (0.02) | 5.82E-08 |
| Chest-RT and pelvis-RT | cg07433723 | chr17 | 66945034 | 66945036 | *NA* | -0.137 | (0.02) | 7.31E-08 |
| Chest-RT and pelvis-RT | cg15999700 | chr8 | 1772981 | 1772983 | *CLN8* | 0.131 | (0.02) | 4.83E-08 |
| Chest-RT and pelvis-RT | cg12393272 | chr17 | 4580527 | 4580529 | *NA* | -0.052 | (0.01) | 2.74E-08 |
| Chest-RT and pelvis-RT | cg26784032 | chr1 | 215523658 | 215523660 | *NA* | -0.137 | (0.02) | 8.17E-08 |
| Chest-RT and pelvis-RT | cg00802613 | chr19 | 2148566 | 2148568 | *AP3D1* | 0.132 | (0.02) | 4.02E-08 |
| Chest-RT and pelvis-RT | cg15004842 | chr7 | 23073105 | 23073107 | *NA* | -0.062 | (0.01) | 5.68E-08 |
| Chest-RT and pelvis-RT | cg18481613 | chr12 | 89019874 | 89019876 | *RP11-13A1.1* | -0.094 | (0.01) | 1.80E-08 |
| Chest-RT and pelvis-RT | cg22510074 | chr7 | 72338137 | 72338139 | *CALN1* | -0.097 | (0.01) | 6.28E-08 |
| Chest-RT and pelvis-RT | cg20598190 | chr2 | 239927506 | 239927508 | *NDUFA10* | -0.216 | (0.03) | 1.43E-08 |
| Chest-RT and pelvis-RT | cg05211447 | chr14 | 103880242 | 103880244 | *CTD-2134A5.4* | -0.128 | (0.02) | 3.45E-08 |
| Chest-RT and pelvis-RT | cg09322375 | chr1 | 33145797 | 33145799 | *TRIM62* | -0.066 | (0.01) | 4.18E-08 |
| Chest-RT and pelvis-RT | cg14040894 | chr4 | 188781846 | 188781848 | *RP11-756P10.2;RP11-756P10.5* | -0.098 | (0.01) | 7.07E-09 |
| Chest-RT and pelvis-RT | cg26216433 | chr14 | 103880253 | 103880255 | *CTD-2134A5.4* | -0.114 | (0.02) | 5.39E-08 |
| Chest-RT and pelvis-RT | cg01340229 | chr3 | 161072613 | 161072615 | *PPM1L* | -0.134 | (0.02) | 1.79E-08 |
| Chest-RT and pelvis-RT | cg06430220 | chr19 | 56102189 | 56102191 | *ZNF787* | 0.094 | (0.01) | 5.15E-08 |
| Chest-RT and pelvis-RT | cg01750375 | chr20 | 44714723 | 44714725 | *KCNK15-AS1;WISP2* | -0.082 | (0.01) | 6.83E-08 |
| Chest-RT and pelvis-RT | cg21094405 | chr13 | 113001802 | 113001804 | *MCF2L* | -0.105 | (0.01) | 1.58E-08 |
| Chest-RT and abdomen-RT | cg00985388 | chr3 | 184234423 | 184234425 | *EIF2B5;VWA5B2* | -0.079 | (0.01) | 3.57E-08 |
| Chest-RT and abdomen-RT | cg09701700 | chr10 | 102435085 | 102435087 | *MIR146B* | -0.080 | (0.01) | 8.71E-08 |
| Chest-RT and abdomen-RT | cg21734651 | chr10 | 74145284 | 74145286 | *AP3M1* | -0.143 | (0.02) | 6.49E-08 |
| Chest-RT and abdomen-RT | cg22815707 | chr18 | 9138607 | 9138609 | *ANKRD12;RP11-21J18.1* | -0.141 | (0.02) | 6.74E-08 |
| Chest-RT and abdomen-RT | cg05449815 | chr17 | 3640570 | 3640572 | *CTNS* | -0.089 | (0.01) | 6.81E-08 |
| Chest-RT and abdomen-RT | cg20338572 | chr16 | 31354820 | 31354822 | *ITGAX* | -0.062 | (0.01) | 6.33E-08 |
| Chest-RT and abdomen-RT | cg19120513 | chr11 | 102318571 | 102318573 | *BIRC3* | -0.059 | (0.01) | 8.05E-08 |
| Chest-RT and abdomen-RT | cg05139637 | chr8 | 60967183 | 60967185 | *AC022182.3* | -0.143 | (0.02) | 8.90E-08 |
| Chest-RT and abdomen-RT | cg10358466 | chr3 | 169240939 | 169240941 | *MECOM* | -0.105 | (0.01) | 2.35E-08 |
| Chest-RT and abdomen-RT | cg21207019 | chr22 | 37727878 | 37727880 | *RP1-37E16.12;TRIOBP* | -0.128 | (0.02) | 3.92E-08 |
| Chest-RT and abdomen-RT | cg19188597 | chr22 | 46074061 | 46074063 | *MIRLET7BHG* | -0.103 | (0.01) | 6.91E-08 |
| Chest-RT and abdomen-RT | cg03872831 | chr22 | 31343379 | 31343381 | *PATZ1* | 0.055 | (0.01) | 8.06E-08 |
| Chest-RT and abdomen-RT | cg09223475 | chr1 | 15344125 | 15344127 | *FHAD1;RP3-467K16.2* | -0.086 | (0.01) | 6.84E-08 |
| Chest-RT and abdomen-RT | cg13930000 | chr5 | 84451826 | 84451828 | *CTD-2269F5.1* | -0.085 | (0.01) | 4.35E-08 |
| Chest-RT and abdomen-RT | cg05726109 | chr22 | 19722231 | 19722233 | *GP1BB;SEPT5* | 0.144 | (0.02) | 3.98E-08 |
| Chest-RT and abdomen-RT | cg03216822 | chr11 | 23805197 | 23805199 | *RP11-945A11.2* | -0.099 | (0.01) | 4.72E-08 |
| Chest-RT and abdomen-RT | cg12393272 | chr17 | 4580527 | 4580529 | *NA* | -0.048 | (0.01) | 7.06E-08 |
| Chest-RT and abdomen-RT | cg01821042 | chr13 | 51995789 | 51995791 | *ATP7B* | -0.065 | (0.01) | 8.25E-09 |
| Chest-RT and abdomen-RT | cg17996394 | chr19 | 4075485 | 4075487 | *NA* | -0.064 | (0.01) | 4.97E-08 |
| Chest-RT and abdomen-RT | cg22510074 | chr7 | 72338137 | 72338139 | *CALN1* | -0.089 | (0.01) | 8.76E-08 |
| Chest-RT and abdomen-RT | cg17850049 | chr1 | 15344019 | 15344021 | *FHAD1;RP3-467K16.2* | -0.092 | (0.01) | 8.35E-08 |
| Chest-RT and abdomen-RT | cg06430220 | chr19 | 56102189 | 56102191 | *ZNF787* | 0.086 | (0.01) | 7.08E-08 |
| Chest-RT and abdomen-RT | cg22013747 | chr12 | 18999131 | 18999133 | *NA* | -0.070 | (0.01) | 4.65E-08 |
| Antimetabolites and vinca alkaloids | cg15159069 | chr3 | 192916299 | 192916301 | *MB21D2* | 0.116 | (0.02) | 1.20E-08 |
| Antimetabolites and vinca alkaloids | cg01543150 | chr3 | 192916271 | 192916273 | *MB21D2* | 0.114 | (0.02) | 1.28E-09 |
| Antimetabolites and vinca alkaloids | cg07784975 | chr12 | 23360735 | 23360737 | *NA* | -0.176 | (0.03) | 1.83E-08 |
| Antimetabolites and vinca alkaloids | cg13224583 | chr1 | 156913952 | 156913954 | *PEAR1* | 0.093 | (0.01) | 5.40E-09 |
| Antimetabolites and vinca alkaloids | cg22587479 | chr2 | 218873503 | 218873505 | *WNT6* | 0.322 | (0.05) | 2.92E-09 |
| Antimetabolites and vinca alkaloids | cg20996351 | chr1 | 27360740 | 27360742 | *MAP3K6* | 0.323 | (0.05) | 7.74E-08 |
| Antimetabolites and vinca alkaloids | cg13959371 | chr1 | 4603882 | 4603884 | *NA* | -0.105 | (0.02) | 8.08E-08 |
| Antimetabolites and vinca alkaloids | cg06795233 | chr2 | 218872669 | 218872671 | *WNT6* | 0.132 | (0.02) | 4.85E-08 |
| Antimetabolites and vinca alkaloids | cg01012330 | chr3 | 192916269 | 192916271 | *MB21D2* | 0.099 | (0.01) | 1.02E-09 |
| Antimetabolites and vinca alkaloids | cg01004017 | chr2 | 120899770 | 120899772 | *GLI2* | 0.121 | (0.02) | 2.13E-08 |
| Antimetabolites and vinca alkaloids | cg25242471 | chr2 | 218874009 | 218874011 | *WNT6* | 0.198 | (0.03) | 4.46E-08 |
| Antimetabolites and vinca alkaloids | cg00011225 | chr2 | 218873591 | 218873593 | *WNT6* | 0.392 | (0.06) | 2.07E-09 |
| Antimetabolites and asparaginase enzymes | cg16586518 | chr3 | 46998723 | 46998725 | *NBEAL2* | 0.060 | (0.01) | 4.75E-09 |
| Antimetabolites and asparaginase enzymes | cg03661817 | chr16 | 57797832 | 57797834 | *CTD-2600O9.1;KIFC3* | 0.119 | (0.02) | 3.30E-08 |
| Antimetabolites and asparaginase enzymes | cg08257257 | chr11 | 68750335 | 68750337 | *TESMIN* | 0.046 | (0.01) | 3.18E-08 |
| Antimetabolites and asparaginase enzymes | cg19611364 | chr19 | 13024679 | 13024681 | *NFIX* | 0.132 | (0.02) | 8.30E-10 |
| Antimetabolites and asparaginase enzymes | cg22729726 | chr1 | 3207289 | 3207291 | *PRDM16;RP11-193J6.1* | 0.075 | (0.01) | 1.66E-10 |
| Antimetabolites and asparaginase enzymes | cg15159069 | chr3 | 192916299 | 192916301 | *MB21D2* | 0.104 | (0.01) | 2.20E-09 |
| Antimetabolites and asparaginase enzymes | cg25406206 | chr19 | 13024537 | 13024539 | *NFIX* | 0.139 | (0.02) | 1.04E-08 |
| Antimetabolites and asparaginase enzymes | cg19151852 | chr17 | 81980734 | 81980736 | *ASPSCR1* | 0.098 | (0.02) | 3.55E-08 |
| Antimetabolites and asparaginase enzymes | cg07588775 | chr6 | 148364586 | 148364588 | *SASH1* | -0.088 | (0.01) | 4.74E-10 |
| Antimetabolites and asparaginase enzymes | cg19773937 | chr1 | 59750958 | 59750960 | *FGGY* | -0.103 | (0.01) | 8.90E-12 |
| Antimetabolites and asparaginase enzymes | cg13750905 | chr16 | 57798087 | 57798089 | *CTD-2600O9.1;KIFC3* | 0.132 | (0.02) | 1.67E-10 |
| Antimetabolites and asparaginase enzymes | cg25236398 | chr12 | 9775728 | 9775730 | *NA* | -0.109 | (0.02) | 5.72E-10 |
| Antimetabolites and asparaginase enzymes | cg08189998 | chr6 | 39471996 | 39471998 | *KIF6* | 0.042 | (0.01) | 5.27E-08 |
| Antimetabolites and asparaginase enzymes | cg16013325 | chr14 | 42429138 | 42429140 | *CTD-2307P3.1;RP11-214N1.1* | -0.064 | (0.01) | 7.84E-09 |
| Antimetabolites and asparaginase enzymes | cg13108341 | chr17 | 11705393 | 11705395 | *DNAH9* | 0.169 | (0.03) | 1.36E-08 |
| Antimetabolites and asparaginase enzymes | cg07097098 | chr19 | 55210269 | 55210271 | *PTPRH* | -0.076 | (0.01) | 1.52E-08 |
| Antimetabolites and asparaginase enzymes | cg10106284 | chr2 | 16666719 | 16666721 | *FAM49A* | -0.095 | (0.01) | 8.21E-10 |
| Antimetabolites and asparaginase enzymes | cg18817664 | chr19 | 2987338 | 2987340 | *TLE6* | 0.064 | (0.01) | 3.59E-10 |
| Antimetabolites and asparaginase enzymes | cg00713549 | chr7 | 41630893 | 41630895 | *NA* | -0.117 | (0.02) | 3.05E-10 |
| Antimetabolites and asparaginase enzymes | cg15627357 | chr16 | 57798142 | 57798144 | *CTD-2600O9.1;KIFC3* | 0.101 | (0.02) | 2.48E-08 |
| Antimetabolites and asparaginase enzymes | cg21211039 | chr22 | 45159729 | 45159731 | *NUP50-DT* | -0.078 | (0.01) | 3.74E-09 |
| Antimetabolites and asparaginase enzymes | cg02773764 | chr4 | 128387233 | 128387235 | *RP11-420A23.1* | -0.078 | (0.01) | 2.75E-08 |
| Antimetabolites and asparaginase enzymes | cg06268875 | chr18 | 11147385 | 11147387 | *PIEZO2* | 0.117 | (0.02) | 1.58E-09 |
| Antimetabolites and asparaginase enzymes | cg10755723 | chr3 | 192859174 | 192859176 | *MB21D2* | 0.052 | (0.01) | 2.32E-08 |
| Antimetabolites and asparaginase enzymes | cg05207048 | chr5 | 168086450 | 168086452 | *CTB-178M22.1;TENM2* | 0.097 | (0.01) | 3.35E-10 |
| Antimetabolites and asparaginase enzymes | cg17976191 | chr19 | 13024712 | 13024714 | *NFIX* | 0.250 | (0.04) | 3.95E-08 |
| Antimetabolites and asparaginase enzymes | cg02274584 | chr21 | 28230124 | 28230126 | *AL035610.2* | 0.129 | (0.02) | 4.49E-08 |
| Antimetabolites and asparaginase enzymes | cg11054905 | chr18 | 46718997 | 46718999 | *ST8SIA5* | -0.084 | (0.01) | 4.41E-09 |
| Antimetabolites and asparaginase enzymes | cg05468212 | chr9 | 134796289 | 134796291 | *COL5A1* | 0.047 | (0.01) | 2.66E-08 |
| Antimetabolites and asparaginase enzymes | cg22499797 | chr17 | 32258886 | 32258888 | *NA* | 0.054 | (0.01) | 6.70E-11 |
| Antimetabolites and asparaginase enzymes | cg17930194 | chr9 | 122220554 | 122220556 | *LHX6* | -0.167 | (0.03) | 6.49E-08 |
| Antimetabolites and asparaginase enzymes | cg12492273 | chr7 | 2079863 | 2079865 | *MAD1L1* | 0.109 | (0.02) | 8.08E-10 |
| Antimetabolites and asparaginase enzymes | cg13817952 | chr9 | 122228176 | 122228178 | *LHX6* | 0.344 | (0.05) | 1.86E-09 |
| Antimetabolites and asparaginase enzymes | cg20019675 | chr8 | 47659495 | 47659497 | *SPIDR* | -0.085 | (0.01) | 1.80E-08 |
| Antimetabolites and asparaginase enzymes | cg25608626 | chr11 | 117492127 | 117492129 | *DSCAML1* | 0.053 | (0.01) | 2.50E-08 |
| Antimetabolites and asparaginase enzymes | cg10279736 | chr4 | 40388508 | 40388510 | *NA* | 0.060 | (0.01) | 6.11E-09 |
| Antimetabolites and asparaginase enzymes | cg08756830 | chr2 | 18337192 | 18337194 | *KCNS3* | -0.100 | (0.02) | 1.56E-08 |
| Antimetabolites and asparaginase enzymes | cg01600165 | chr12 | 62269167 | 62269169 | *FAM19A2;USP15* | -0.111 | (0.02) | 1.84E-09 |
| Antimetabolites and asparaginase enzymes | cg17661220 | chr20 | 3712531 | 3712533 | *NA* | 0.046 | (0.01) | 1.75E-08 |
| Antimetabolites and asparaginase enzymes | cg09720404 | chr9 | 79801729 | 79801731 | *NA* | -0.081 | (0.01) | 5.82E-10 |
| Antimetabolites and asparaginase enzymes | cg07524540 | chr17 | 15490701 | 15490703 | *CDRT4;TVP23C-CDRT4* | -0.100 | (0.02) | 6.51E-08 |
| Antimetabolites and asparaginase enzymes | cg06795233 | chr2 | 218872669 | 218872671 | *WNT6* | 0.125 | (0.02) | 8.34E-09 |
| Antimetabolites and asparaginase enzymes | cg26299084 | chr4 | 80197433 | 80197435 | *PRDM8* | 0.238 | (0.03) | 4.66E-10 |
| Antimetabolites and asparaginase enzymes | cg01693697 | chr15 | 73920554 | 73920556 | *LOXL1-AS1* | -0.103 | (0.02) | 7.45E-08 |
| Antimetabolites and asparaginase enzymes | cg07598481 | chr13 | 72194680 | 72194682 | *NA* | -0.124 | (0.02) | 6.33E-09 |
| Antimetabolites and asparaginase enzymes | cg17389813 | chr11 | 122189556 | 122189558 | *MIR100HG* | -0.155 | (0.02) | 9.58E-10 |
| Antimetabolites and asparaginase enzymes | cg24470086 | chr4 | 157316279 | 157316281 | *GRIA2* | -0.123 | (0.02) | 2.69E-08 |
| Antimetabolites and asparaginase enzymes | cg19099872 | chr1 | 231929466 | 231929468 | *DISC1;DISC1-IT1* | 0.080 | (0.01) | 3.43E-08 |
| Antimetabolites and asparaginase enzymes | cg07396746 | chr8 | 23304781 | 23304783 | *LOXL2* | 0.065 | (0.01) | 7.61E-08 |
| Antimetabolites and asparaginase enzymes | cg06192447 | chr11 | 18793697 | 18793699 | *PTPN5* | 0.051 | (0.01) | 3.94E-08 |
| Antimetabolites and asparaginase enzymes | cg15648792 | chr18 | 11146255 | 11146257 | *PIEZO2* | 0.064 | (0.01) | 7.81E-08 |
| Antimetabolites and asparaginase enzymes | cg01521220 | chr17 | 48156436 | 48156438 | *MIR1203;SKAP1* | 0.057 | (0.01) | 6.51E-08 |
| Antimetabolites and asparaginase enzymes | cg08214927 | chr14 | 91299479 | 91299481 | *CCDC88C* | 0.104 | (0.01) | 1.13E-09 |
| Antimetabolites and asparaginase enzymes | cg04084749 | chr2 | 186575639 | 186575641 | *NA* | -0.121 | (0.02) | 6.91E-08 |
| Antimetabolites and asparaginase enzymes | cg25466245 | chr1 | 223233752 | 223233754 | *SUSD4* | -0.066 | (0.01) | 3.42E-10 |
| Antimetabolites and asparaginase enzymes | cg17125362 | chr18 | 8526137 | 8526139 | *NA* | -0.064 | (0.01) | 7.36E-08 |
| Antimetabolites and asparaginase enzymes | cg19664938 | chr5 | 6493144 | 6493146 | *UBE2QL1* | -0.068 | (0.01) | 4.28E-08 |
| Antimetabolites and asparaginase enzymes | cg02509576 | chr2 | 67826054 | 67826056 | *AC010987.6* | -0.091 | (0.01) | 4.96E-09 |
| Antimetabolites and asparaginase enzymes | cg01001422 | chr19 | 13024816 | 13024818 | *NFIX* | 0.102 | (0.01) | 2.58E-09 |
| Antimetabolites and asparaginase enzymes | cg02476619 | chr17 | 19545030 | 19545032 | *SLC47A1* | 0.045 | (0.01) | 7.97E-09 |
| Antimetabolites and asparaginase enzymes | cg19550755 | chr11 | 18773348 | 18773350 | *PTPN5* | 0.059 | (0.01) | 2.41E-08 |
| Antimetabolites and asparaginase enzymes | cg16055613 | chr6 | 163766368 | 163766370 | *NA* | -0.093 | (0.01) | 6.43E-09 |
| Antimetabolites and asparaginase enzymes | cg00268509 | chr6 | 148364301 | 148364303 | *SASH1* | -0.112 | (0.02) | 7.36E-09 |
| Antimetabolites and asparaginase enzymes | cg15391651 | chr1 | 165163035 | 165163037 | *NA* | 0.110 | (0.02) | 1.72E-09 |
| Antimetabolites and asparaginase enzymes | cg06307913 | chr4 | 80197639 | 80197641 | *PRDM8* | 0.213 | (0.03) | 3.47E-08 |
| Antimetabolites and asparaginase enzymes | cg08120188 | chr1 | 230360054 | 230360056 | *PGBD5* | 0.106 | (0.02) | 9.73E-10 |
| Antimetabolites and asparaginase enzymes | cg23165574 | chr9 | 16510110 | 16510112 | *BNC2* | -0.079 | (0.01) | 1.26E-11 |
| Antimetabolites and asparaginase enzymes | cg13352096 | chr16 | 57798172 | 57798174 | *CTD-2600O9.1;KIFC3* | 0.139 | (0.02) | 3.45E-10 |
| Antimetabolites and asparaginase enzymes | cg10119082 | chr7 | 99393002 | 99393004 | *ARPC1B;PDAP1* | 0.227 | (0.04) | 8.70E-08 |
| Antimetabolites and asparaginase enzymes | cg05429921 | chr19 | 13024503 | 13024505 | *NFIX* | 0.123 | (0.02) | 9.66E-09 |
| Antimetabolites and asparaginase enzymes | cg27192248 | chr15 | 64993330 | 64993332 | *NA* | -0.199 | (0.03) | 3.41E-09 |
| Antimetabolites and asparaginase enzymes | cg12641545 | chr18 | 7601197 | 7601199 | *PTPRM* | -0.138 | (0.02) | 1.37E-08 |
| Antimetabolites and asparaginase enzymes | cg01543150 | chr3 | 192916271 | 192916273 | *MB21D2* | 0.112 | (0.01) | 7.04E-13 |
| Antimetabolites and asparaginase enzymes | cg13474360 | chr3 | 192885619 | 192885621 | *MB21D2* | 0.067 | (0.01) | 3.70E-08 |
| Antimetabolites and asparaginase enzymes | cg14193434 | chr13 | 67228751 | 67228753 | *PCDH9* | 0.115 | (0.02) | 8.74E-08 |
| Antimetabolites and asparaginase enzymes | cg18238078 | chr4 | 173834349 | 173834351 | *NA* | 0.097 | (0.01) | 7.16E-09 |
| Antimetabolites and asparaginase enzymes | cg23344395 | chr3 | 192916212 | 192916214 | *MB21D2* | 0.078 | (0.01) | 8.28E-09 |
| Antimetabolites and asparaginase enzymes | cg15975895 | chr3 | 178950818 | 178950820 | *NA* | -0.115 | (0.02) | 2.13E-10 |
| Antimetabolites and asparaginase enzymes | cg03479114 | chr4 | 165359572 | 165359574 | *NA* | -0.110 | (0.02) | 3.03E-10 |
| Antimetabolites and asparaginase enzymes | cg03984209 | chr3 | 55885989 | 55885991 | *ERC2* | -0.100 | (0.01) | 1.06E-09 |
| Antimetabolites and asparaginase enzymes | cg01564268 | chr17 | 38510716 | 38510718 | *ARHGAP23* | 0.160 | (0.03) | 4.72E-08 |
| Antimetabolites and asparaginase enzymes | cg11196506 | chr14 | 38290976 | 38290978 | *RP11-96D24.1* | -0.117 | (0.02) | 4.59E-08 |
| Antimetabolites and asparaginase enzymes | cg03463411 | chr4 | 80197033 | 80197035 | *PRDM8* | 0.203 | (0.03) | 2.51E-08 |
| Antimetabolites and asparaginase enzymes | cg10129063 | chr4 | 80190230 | 80190232 | *PRDM8;RP11-377G16.2* | 0.159 | (0.03) | 5.97E-08 |
| Antimetabolites and asparaginase enzymes | cg06815976 | chr6 | 32197038 | 32197040 | *NOTCH4* | 0.086 | (0.01) | 2.06E-08 |
| Antimetabolites and asparaginase enzymes | cg17138053 | chr6 | 137282317 | 137282319 | *NA* | 0.050 | (0.01) | 1.72E-09 |
| Antimetabolites and asparaginase enzymes | cg08650597 | chr15 | 100902500 | 100902502 | *ALDH1A3;RP11-66B24.4* | -0.072 | (0.01) | 7.49E-08 |
| Antimetabolites and asparaginase enzymes | cg02462487 | chr11 | 2899119 | 2899121 | *SLC22A18;SLC22A18AS* | -0.078 | (0.01) | 1.12E-09 |
| Antimetabolites and asparaginase enzymes | cg09862081 | chr15 | 48857189 | 48857191 | *SHC4* | 0.074 | (0.01) | 6.32E-09 |
| Antimetabolites and asparaginase enzymes | cg03250223 | chr3 | 23206514 | 23206516 | *UBE2E2* | 0.047 | (0.01) | 5.00E-08 |
| Antimetabolites and asparaginase enzymes | cg02329226 | chr11 | 66209316 | 66209318 | *PACS1* | 0.051 | (0.01) | 6.75E-08 |
| Antimetabolites and asparaginase enzymes | cg04252928 | chr16 | 89102190 | 89102192 | *ACSF3* | 0.205 | (0.03) | 3.59E-11 |
| Antimetabolites and asparaginase enzymes | cg17077319 | chr3 | 150168879 | 150168881 | *RP11-167H9.4* | 0.110 | (0.02) | 1.15E-08 |
| Antimetabolites and asparaginase enzymes | cg18073471 | chr4 | 80198043 | 80198045 | *PRDM8* | 0.070 | (0.01) | 7.10E-09 |
| Antimetabolites and asparaginase enzymes | cg21940313 | chr17 | 43543542 | 43543544 | *DHX8;ETV4;RP11-392O1.4* | 0.109 | (0.02) | 8.26E-10 |
| Antimetabolites and asparaginase enzymes | cg05478824 | chr17 | 82012258 | 82012260 | *ASPSCR1* | 0.073 | (0.01) | 6.21E-08 |
| Antimetabolites and asparaginase enzymes | cg02259914 | chr20 | 3712573 | 3712575 | *NA* | 0.060 | (0.01) | 1.77E-09 |
| Antimetabolites and asparaginase enzymes | cg06846279 | chr1 | 115670656 | 115670658 | *VANGL1* | 0.074 | (0.01) | 3.11E-08 |
| Antimetabolites and asparaginase enzymes | cg13631444 | chr8 | 109086636 | 109086638 | *TRHR* | 0.108 | (0.02) | 9.66E-09 |
| Antimetabolites and asparaginase enzymes | cg07839457 | chr16 | 56989109 | 56989111 | *NLRC5* | -0.118 | (0.02) | 2.19E-08 |
| Antimetabolites and asparaginase enzymes | cg01978236 | chr6 | 32223701 | 32223703 | *NOTCH4* | -0.069 | (0.01) | 5.28E-08 |
| Antimetabolites and asparaginase enzymes | cg16776685 | chr19 | 46076475 | 46076477 | *IGFL4* | -0.115 | (0.02) | 7.43E-08 |
| Antimetabolites and asparaginase enzymes | cg06373870 | chr4 | 80196698 | 80196700 | *PRDM8* | 0.174 | (0.03) | 3.11E-08 |
| Antimetabolites and asparaginase enzymes | cg04965297 | chr19 | 13024514 | 13024516 | *NFIX* | 0.122 | (0.02) | 4.09E-08 |
| Antimetabolites and asparaginase enzymes | cg26833883 | chr1 | 154993095 | 154993097 | *FLAD1;LENEP* | 0.037 | (0.01) | 8.24E-08 |
| Antimetabolites and asparaginase enzymes | cg13490403 | chr9 | 122220133 | 122220135 | *LHX6* | -0.134 | (0.02) | 2.40E-08 |
| Antimetabolites and asparaginase enzymes | cg14094320 | chr1 | 209405369 | 209405371 | *NA* | 0.061 | (0.01) | 1.82E-08 |
| Antimetabolites and asparaginase enzymes | cg04688366 | chr7 | 11458999 | 11459001 | *AC004538.3;THSD7A* | -0.091 | (0.01) | 1.75E-09 |
| Antimetabolites and asparaginase enzymes | cg16887422 | chr2 | 239113410 | 239113412 | *HDAC4* | 0.096 | (0.01) | 1.37E-09 |
| Antimetabolites and asparaginase enzymes | cg26378115 | chr3 | 190109234 | 190109236 | *P3H2* | 0.089 | (0.01) | 2.12E-08 |
| Antimetabolites and asparaginase enzymes | cg09022082 | chr1 | 236107284 | 236107286 | *NA* | -0.065 | (0.01) | 1.04E-08 |
| Antimetabolites and asparaginase enzymes | cg07051715 | chr12 | 14654652 | 14654654 | *GUCY2C* | -0.092 | (0.01) | 3.76E-10 |
| Antimetabolites and asparaginase enzymes | cg11031221 | chr17 | 72421375 | 72421377 | *LINC00511* | -0.081 | (0.01) | 3.35E-11 |
| Antimetabolites and asparaginase enzymes | cg25989755 | chr4 | 150213330 | 150213332 | *DCLK2* | 0.098 | (0.02) | 8.53E-08 |
| Antimetabolites and asparaginase enzymes | cg24791731 | chr10 | 19818855 | 19818857 | *PLXDC2* | 0.124 | (0.02) | 1.08E-08 |
| Antimetabolites and asparaginase enzymes | cg27446735 | chr1 | 169465943 | 169465945 | *SLC19A2* | 0.108 | (0.02) | 4.62E-09 |
| Antimetabolites and asparaginase enzymes | cg12496433 | chr2 | 16666494 | 16666496 | *FAM49A* | -0.087 | (0.01) | 7.25E-08 |
| Antimetabolites and asparaginase enzymes | cg14547509 | chr9 | 14318498 | 14318500 | *NFIB;RP11-120J1.1* | -0.075 | (0.01) | 4.50E-08 |
| Antimetabolites and asparaginase enzymes | cg08119153 | chr14 | 78446957 | 78446959 | *NRXN3* | 0.118 | (0.02) | 5.68E-09 |
| Antimetabolites and asparaginase enzymes | cg07172149 | chr7 | 136883124 | 136883126 | *AC009264.1;CHRM2* | -0.104 | (0.01) | 1.72E-10 |
| Asparaginase enzymes and epipodophyllotoxins | cg13648318 | chr9 | 122229128 | 122229130 | *LHX6* | 0.193 | (0.03) | 5.14E-08 |
| Asparaginase enzymes and epipodophyllotoxins | cg02259914 | chr20 | 3712573 | 3712575 | *NA* | 0.059 | (0.01) | 2.66E-08 |
| Asparaginase enzymes and epipodophyllotoxins | cg02533526 | chr1 | 149939390 | 149939392 | *OTUD7B* | -0.067 | (0.01) | 9.14E-09 |
| Asparaginase enzymes and epipodophyllotoxins | cg12492273 | chr7 | 2079863 | 2079865 | *MAD1L1* | 0.105 | (0.02) | 4.94E-08 |
| Asparaginase enzymes and epipodophyllotoxins | cg03029958 | chr3 | 2090634 | 2090636 | *RN7SKP144* | -0.129 | (0.02) | 1.23E-09 |
| Asparaginase enzymes and epipodophyllotoxins | cg25406206 | chr19 | 13024537 | 13024539 | *NFIX* | 0.144 | (0.02) | 6.81E-09 |
| Asparaginase enzymes and epipodophyllotoxins | cg02626395 | chr7 | 151918463 | 151918465 | *NA* | 0.069 | (0.01) | 8.84E-08 |
| Asparaginase enzymes and epipodophyllotoxins | cg07661704 | chr4 | 138223278 | 138223280 | *SLC7A11* | 0.099 | (0.02) | 4.34E-08 |
| Asparaginase enzymes and epipodophyllotoxins | cg26766364 | chr16 | 89921077 | 89921079 | *RP11-566K11.4;TUBB3* | 0.070 | (0.01) | 8.75E-10 |
| Asparaginase enzymes and epipodophyllotoxins | cg23256579 | chr12 | 10849803 | 10849805 | *PRH1;PRH1-PRR4;PRR4* | -0.126 | (0.02) | 1.34E-08 |
| Asparaginase enzymes and epipodophyllotoxins | cg21756476 | chr15 | 60890565 | 60890567 | *RORA* | 0.062 | (0.01) | 6.15E-08 |
| Asparaginase enzymes and epipodophyllotoxins | cg02041498 | chr2 | 66508716 | 66508718 | *MEIS1* | 0.123 | (0.02) | 3.77E-08 |
| Asparaginase enzymes and epipodophyllotoxins | cg01217608 | chr10 | 35267482 | 35267484 | *CCNY* | 0.056 | (0.01) | 5.09E-08 |
| Asparaginase enzymes and epipodophyllotoxins | cg24470086 | chr4 | 157316279 | 157316281 | *GRIA2* | -0.134 | (0.02) | 1.85E-08 |
| Asparaginase enzymes and epipodophyllotoxins | cg26066361 | chr12 | 10130021 | 10130023 | *CLEC7A* | -0.094 | (0.02) | 3.36E-08 |
| Asparaginase enzymes and epipodophyllotoxins | cg18381051 | chr1 | 3071080 | 3071082 | *PRDM16* | 0.185 | (0.03) | 3.94E-08 |
| Asparaginase enzymes and epipodophyllotoxins | cg17138053 | chr6 | 137282317 | 137282319 | *NA* | 0.053 | (0.01) | 1.72E-09 |
| Asparaginase enzymes and epipodophyllotoxins | cg25571095 | chr6 | 35411068 | 35411070 | *PPARD* | 0.071 | (0.01) | 3.75E-08 |
| Asparaginase enzymes and epipodophyllotoxins | cg10106284 | chr2 | 16666719 | 16666721 | *FAM49A* | -0.088 | (0.01) | 7.53E-08 |
| Asparaginase enzymes and epipodophyllotoxins | cg16983588 | chr11 | 129923212 | 129923214 | *PRDM10* | 0.117 | (0.02) | 5.67E-08 |
| Asparaginase enzymes and epipodophyllotoxins | cg18817664 | chr19 | 2987338 | 2987340 | *TLE6* | 0.063 | (0.01) | 1.60E-08 |
| Asparaginase enzymes and epipodophyllotoxins | cg27446735 | chr1 | 169465943 | 169465945 | *SLC19A2* | 0.106 | (0.02) | 4.30E-08 |
| Asparaginase enzymes and epipodophyllotoxins | cg00794400 | chr1 | 17420013 | 17420015 | *RCC2* | 0.167 | (0.03) | 4.95E-09 |
| Asparaginase enzymes and epipodophyllotoxins | cg16353615 | chr17 | 21464251 | 21464253 | *NA* | 0.085 | (0.01) | 2.70E-08 |
| Asparaginase enzymes and epipodophyllotoxins | cg16234884 | chr6 | 21067733 | 21067735 | *CDKAL1* | -0.091 | (0.02) | 8.79E-08 |
| Asparaginase enzymes and epipodophyllotoxins | cg08119153 | chr14 | 78446957 | 78446959 | *NRXN3* | 0.118 | (0.02) | 2.16E-08 |
| Asparaginase enzymes and epipodophyllotoxins | cg05202300 | chr11 | 24324614 | 24324616 | *NA* | 0.170 | (0.03) | 3.33E-08 |
| Asparaginase enzymes and epipodophyllotoxins | cg04839576 | chr9 | 107213641 | 107213643 | *NA* | -0.112 | (0.02) | 4.31E-08 |
| Asparaginase enzymes and epipodophyllotoxins | cg00268509 | chr6 | 148364301 | 148364303 | *SASH1* | -0.110 | (0.02) | 7.00E-08 |
| Asparaginase enzymes and epipodophyllotoxins | cg26487948 | chr5 | 172932571 | 172932573 | *ERGIC1* | 0.143 | (0.02) | 6.31E-08 |
| Asparaginase enzymes and epipodophyllotoxins | cg15572436 | chr11 | 62999696 | 62999698 | *SLC22A8* | 0.059 | (0.01) | 1.03E-08 |
| Asparaginase enzymes and corticosteroids | cg00280812 | chr12 | 52192059 | 52192061 | *KRT80* | -0.063 | (0.01) | 3.14E-11 |
| Asparaginase enzymes and corticosteroids | cg08120188 | chr1 | 230360054 | 230360056 | *PGBD5* | 0.091 | (0.01) | 3.20E-08 |
| Asparaginase enzymes and corticosteroids | cg04235768 | chr4 | 80197188 | 80197190 | *PRDM8* | 0.200 | (0.03) | 4.90E-08 |
| Asparaginase enzymes and corticosteroids | cg07116861 | chr6 | 53615893 | 53615895 | *GCLC;RP1-27K12.2* | 0.134 | (0.02) | 8.06E-08 |
| Asparaginase enzymes and corticosteroids | cg13352096 | chr16 | 57798172 | 57798174 | *CTD-2600O9.1;KIFC3* | 0.113 | (0.02) | 5.80E-08 |
| Asparaginase enzymes and corticosteroids | cg03029958 | chr3 | 2090634 | 2090636 | *RN7SKP144* | -0.113 | (0.02) | 2.70E-09 |
| Asparaginase enzymes and corticosteroids | cg13839439 | chr12 | 53750736 | 53750738 | *CISTR* | 0.058 | (0.01) | 3.49E-08 |
| Asparaginase enzymes and corticosteroids | cg15159069 | chr3 | 192916299 | 192916301 | *MB21D2* | 0.097 | (0.01) | 4.54E-09 |
| Asparaginase enzymes and corticosteroids | cg01543150 | chr3 | 192916271 | 192916273 | *MB21D2* | 0.094 | (0.01) | 2.39E-10 |
| Asparaginase enzymes and corticosteroids | cg22794504 | chr19 | 5173246 | 5173248 | *PTPRS* | 0.101 | (0.02) | 4.64E-09 |
| Asparaginase enzymes and corticosteroids | cg13787850 | chr9 | 99433668 | 99433670 | *NA* | -0.070 | (0.01) | 4.62E-08 |
| Asparaginase enzymes and corticosteroids | cg19773937 | chr1 | 59750958 | 59750960 | *FGGY* | -0.081 | (0.01) | 1.58E-08 |
| Asparaginase enzymes and corticosteroids | cg08590873 | chr14 | 66496859 | 66496861 | *CCDC196;RP11-72M17.1* | 0.125 | (0.02) | 4.74E-08 |
| Asparaginase enzymes and corticosteroids | cg04839576 | chr9 | 107213641 | 107213643 | *NA* | -0.108 | (0.02) | 1.76E-09 |
| Asparaginase enzymes and corticosteroids | cg26378115 | chr3 | 190109234 | 190109236 | *P3H2* | 0.084 | (0.01) | 3.06E-08 |
| Asparaginase enzymes and corticosteroids | cg08189998 | chr6 | 39471996 | 39471998 | *KIF6* | 0.040 | (0.01) | 5.33E-08 |
| Asparaginase enzymes and corticosteroids | cg17661220 | chr20 | 3712531 | 3712533 | *NA* | 0.043 | (0.01) | 3.27E-08 |
| Asparaginase enzymes and corticosteroids | cg07065756 | chr7 | 2079704 | 2079706 | *MAD1L1* | 0.082 | (0.01) | 3.71E-08 |
| Asparaginase enzymes and corticosteroids | cg01440687 | chr14 | 42541813 | 42541815 | *CTD-2307P3.1* | -0.121 | (0.02) | 6.19E-08 |
| Asparaginase enzymes and corticosteroids | cg06815976 | chr6 | 32197038 | 32197040 | *NOTCH4* | 0.083 | (0.01) | 8.47E-09 |
| Asparaginase enzymes and corticosteroids | cg13750905 | chr16 | 57798087 | 57798089 | *CTD-2600O9.1;KIFC3* | 0.107 | (0.02) | 3.23E-08 |
| Asparaginase enzymes and corticosteroids | cg02259914 | chr20 | 3712573 | 3712575 | *NA* | 0.051 | (0.01) | 6.61E-08 |
| Asparaginase enzymes and corticosteroids | cg02476619 | chr17 | 19545030 | 19545032 | *SLC47A1* | 0.040 | (0.01) | 5.55E-08 |
| Asparaginase enzymes and corticosteroids | cg03250223 | chr3 | 23206514 | 23206516 | *UBE2E2* | 0.044 | (0.01) | 3.86E-08 |
| Asparaginase enzymes and corticosteroids | cg21211039 | chr22 | 45159729 | 45159731 | *NUP50-DT* | -0.076 | (0.01) | 8.16E-10 |
| Asparaginase enzymes and corticosteroids | cg01635117 | chr13 | 106859783 | 106859785 | *NA* | 0.073 | (0.01) | 6.73E-08 |
| Asparaginase enzymes and corticosteroids | cg08847140 | chr1 | 146018234 | 146018236 | *HJV* | 0.052 | (0.01) | 6.17E-08 |
| Asparaginase enzymes and corticosteroids | cg15391651 | chr1 | 165163035 | 165163037 | *NA* | 0.097 | (0.01) | 1.66E-08 |
| Corticosteroids and vinca alkaloids | cg01504762 | chr2 | 115647916 | 115647918 | *DPP10* | -0.101 | (0.02) | 4.16E-08 |

| **Table S4. GO biological process annotations of specific treatment-associated CpG sites** | | | | | | | |
| --- | --- | --- | --- | --- | --- | --- | --- |
|  | REFLIST (N=20851) | CpG sites (N=187) | Expected value | Over/under | Fold enrichment | *P*_raw_ | *P*_FDR_ |
| ***Chest-RT associated CpG sites and GO biological process complete*** | | | | | | | |
| positive regulation of cellular process (GO:0048522) | 5695 | 82 | 51.08 | + | 1.61 | 1.41E-06 | 2.24E-02 |
| response to jasmonic acid (GO:0009753) | 4 | 3 | 0.04 | + | 83.63 | 2.36E-05 | 3.12E-02 |
| positive regulation of cell differentiation (GO:0045597) | 837 | 21 | 7.51 | + | 2.8 | 2.69E-05 | 3.28E-02 |
| cellular response to jasmonic acid stimulus (GO:0071395) | 4 | 3 | 0.04 | + | 83.63 | 2.36E-05 | 3.40E-02 |
| negative regulation of cellular process (GO:0048523) | 4952 | 71 | 44.41 | + | 1.6 | 1.99E-05 | 3.50E-02 |
| anatomical structure development (GO:0048856) | 5301 | 74 | 47.54 | + | 1.56 | 2.26E-05 | 3.58E-02 |
| regulation of cellular component organization (GO:0051128) | 2373 | 41 | 21.28 | + | 1.93 | 4.12E-05 | 3.63E-02 |
| animal organ development (GO:0048513) | 3247 | 51 | 29.12 | + | 1.75 | 4.45E-05 | 3.71E-02 |
| cell differentiation (GO:0030154) | 3540 | 55 | 31.75 | + | 1.73 | 3.29E-05 | 3.73E-02 |
| cellular developmental process (GO:0048869) | 3596 | 55 | 32.25 | + | 1.71 | 4.01E-05 | 3.74E-02 |
| positive regulation of nitrogen compound metabolic process (GO:0051173) | 3206 | 51 | 28.75 | + | 1.77 | 3.81E-05 | 3.78E-02 |
| biological adhesion (GO:0022610) | 953 | 23 | 8.55 | + | 2.69 | 1.98E-05 | 3.92E-02 |
| positive regulation of cellular metabolic process (GO:0031325) | 3392 | 53 | 30.42 | + | 1.74 | 3.77E-05 | 3.99E-02 |
| regulation of cell population proliferation (GO:0042127) | 1667 | 33 | 14.95 | + | 2.21 | 1.85E-05 | 4.20E-02 |
| tissue development (GO:0009888) | 1763 | 35 | 15.81 | + | 2.21 | 8.98E-06 | 4.75E-02 |
| positive regulation of blood vessel endothelial cell migration (GO:0043536) | 56 | 6 | 0.5 | + | 11.95 | 1.85E-05 | 4.89E-02 |
| positive regulation of cell-cell adhesion (GO:0022409) | 280 | 11 | 2.51 | + | 4.38 | 6.20E-05 | 4.92E-02 |
| positive regulation of biological process (GO:0048518) | 6290 | 86 | 56.41 | + | 1.52 | 6.32E-06 | 5.02E-02 |
| UV protection (GO:0009650) | 13 | 4 | 0.12 | + | 34.31 | 1.31E-05 | 5.21E-02 |
| cell adhesion (GO:0007155) | 947 | 23 | 8.49 | + | 2.71 | 1.80E-05 | 5.70E-02 |
|  | REFLIST (N=20851) | CpG sites (N=187) | Expected value | Over/under | Fold enrichment | *P*_raw_ | *P*_FDR_ |
| ***Epipodophyllotoxins associated CpG sites and GO biological process complete*** | | | | | | | |
| anatomical structure development (GO:0048856) | 5301 | 83 | 49.58 | + | 1.67 | 2.25E-07 | 3.56E-03 |
| system development (GO:0048731) | 4317 | 70 | 40.37 | + | 1.73 | 1.23E-06 | 6.51E-03 |
| multicellular organism development (GO:0007275) | 4906 | 76 | 45.88 | + | 1.66 | 1.68E-06 | 6.66E-03 |
| regulation of multicellular organismal process (GO:0051239) | 2698 | 51 | 25.23 | + | 2.02 | 9.09E-07 | 7.21E-03 |
| developmental process (GO:0032502) | 5765 | 85 | 53.91 | + | 1.58 | 2.49E-06 | 7.90E-03 |
| regulation of multicellular organismal development (GO:2000026) | 1332 | 31 | 12.46 | + | 2.49 | 5.12E-06 | 1.35E-02 |
| positive regulation of cellular process (GO:0048522) | 5695 | 82 | 53.26 | + | 1.54 | 1.12E-05 | 1.78E-02 |
| regulation of developmental process (GO:0050793) | 2469 | 45 | 23.09 | + | 1.95 | 1.12E-05 | 1.98E-02 |
| nervous system development (GO:0007399) | 2203 | 42 | 20.6 | + | 2.04 | 1.05E-05 | 2.08E-02 |
| animal organ development (GO:0048513) | 3247 | 55 | 30.37 | + | 1.81 | 9.93E-06 | 2.25E-02 |
| animal organ morphogenesis (GO:0009887) | 972 | 24 | 9.09 | + | 2.64 | 1.80E-05 | 2.60E-02 |
| anatomical structure morphogenesis (GO:0009653) | 2182 | 40 | 20.41 | + | 1.96 | 3.35E-05 | 4.42E-02 |

| **Table S5. Association of treatment-associated methylation sites with CHCs (*P_FDR_*<0.05)** | | | | | | | | | | |
| --- | --- | --- | --- | --- | --- | --- | --- | --- | --- | --- |
| Treatment | CHC | CpG | Chromosome | Start position | End position | HGNC gene | Coefficient | (SE) | *P_raw_* | *P_FDR_* |
| Abdomen-RT | HTG | cg19634849 | chr5 | 140,259,739 | 140,259,741 | CYSTM1;PFDN1 | -2.014 | (0.388) | 2.0E-07 | 6.7E-05 |
| Abdomen-RT | HTG | cg13552692 | chr18 | 68,722,209 | 68,722,211 | CCDC102B | -1.071 | (0.259) | 3.5E-05 | 5.7E-03 |
| Abdomen-RT | HTG | cg21393163 | chr1 | 12,157,571 | 12,157,573 | NA | -0.907 | (0.225) | 5.7E-05 | 5.8E-03 |
| Abdomen-RT | HTG | cg12051710 | chr12 | 2,879,903 | 2,879,905 | RHNO1;TULP3 | -1.118 | (0.287) | 1.0E-04 | 5.8E-03 |
| Abdomen-RT | HTG | cg08578520 | chr16 | 57,462,411 | 57,462,413 | AC009052.12;POLR2C | -1.524 | (0.396) | 1.2E-04 | 5.8E-03 |
| Abdomen-RT | HTG | cg03957124 | chr6 | 37,049,092 | 37,049,094 | NA | -1.493 | (0.389) | 1.2E-04 | 5.8E-03 |
| Abdomen-RT | HTG | cg02786370 | chr4 | 2,746,200 | 2,746,202 | TNIP2 | -0.863 | (0.225) | 1.2E-04 | 5.8E-03 |
| Abdomen-RT | HTG | cg05293861 | chr12 | 4,153,541 | 4,153,543 | NA | -0.696 | (0.187) | 2.0E-04 | 8.3E-03 |
| Abdomen-RT | HTG | cg06009645 | chr6 | 111,714,895 | 111,714,897 | FYN | -1.375 | (0.377) | 2.6E-04 | 9.6E-03 |
| Abdomen-RT | HTG | cg19758958 | chr11 | 62,551,749 | 62,551,751 | AHNAK | -1.581 | (0.448) | 4.2E-04 | 1.4E-02 |
| Abdomen-RT | HTG | cg16621591 | chr14 | 98,710,756 | 98,710,758 | C14orf177 | -0.990 | (0.287) | 5.6E-04 | 1.7E-02 |
| Abdomen-RT | HTG | cg07545728 | chr10 | 80,501,100 | 80,501,102 | TSPAN14 | -0.883 | (0.259) | 6.5E-04 | 1.8E-02 |
| Abdomen-RT | HTG | cg09921385 | chr4 | 6,999,154 | 6,999,156 | TBC1D14 | -0.770 | (0.232) | 9.0E-04 | 2.3E-02 |
| Abdomen-RT | HTG | cg09853238 | chr6 | 149,211,153 | 149,211,155 | NA | -1.213 | (0.381) | 1.4E-03 | 3.4E-02 |
| Abdomen-RT | HTG | cg13294852 | chr2 | 102,055,667 | 102,055,669 | NA | -0.755 | (0.241) | 1.7E-03 | 3.8E-02 |
| Abdomen-RT | HTG | cg07872945 | chr9 | 134,010,913 | 134,010,915 | NA | -0.877 | (0.286) | 2.2E-03 | 4.5E-02 |
| Brain-RT | HTG | cg13360224 | chr6 | 14,232,103 | 14,232,105 | RP11-359N11.1 | 1.482 | (0.385) | 1.2E-04 | 1.1E-03 |
| Abdomen-RT | HCL | cg01750375 | chr20 | 44,714,723 | 44,714,725 | KCNK15-AS1;WISP2 | -1.675 | (0.339) | 7.7E-07 | 2.5E-04 |
| Abdomen-RT | HCL | cg01511232 | chr4 | 154,740,776 | 154,740,778 | LRAT | 0.849 | (0.182) | 3.1E-06 | 3.6E-04 |
| Abdomen-RT | HCL | cg02286081 | chr6 | 33,076,063 | 33,076,065 | HLA-DPA1;HLA-DPB1 | -0.801 | (0.172) | 3.3E-06 | 3.6E-04 |
| Abdomen-RT | HCL | cg21922478 | chr5 | 52,945,259 | 52,945,261 | CTD-2175A23.1;ITGA1 | -0.895 | (0.198) | 6.2E-06 | 5.1E-04 |
| Abdomen-RT | HCL | cg09762316 | chr9 | 70,043,437 | 70,043,439 | MAMDC2;MAMDC2-AS1 | 1.312 | (0.309) | 2.2E-05 | 9.7E-04 |
| Abdomen-RT | HCL | cg26350754 | chr6 | 33,076,090 | 33,076,092 | HLA-DPA1;HLA-DPB1 | -0.895 | (0.212) | 2.3E-05 | 9.7E-04 |
| Abdomen-RT | HCL | cg06963130 | chr2 | 235,434,127 | 235,434,129 | NA | -1.009 | (0.239) | 2.4E-05 | 9.7E-04 |
| Abdomen-RT | HCL | cg22976567 | chr1 | 156,104,390 | 156,104,392 | LMNA | -1.423 | (0.337) | 2.4E-05 | 9.7E-04 |
| Abdomen-RT | HCL | cg07323488 | chr3 | 168,467,524 | 168,467,526 | EGFEM1P | -1.268 | (0.307) | 3.6E-05 | 1.3E-03 |
| Abdomen-RT | HCL | cg25241559 | chr2 | 241,049,961 | 241,049,963 | AC005237.4;SNED1 | 1.822 | (0.444) | 4.0E-05 | 1.3E-03 |
| Abdomen-RT | HCL | cg12530994 | chr10 | 5,094,589 | 5,094,591 | AKR1C1;AKR1C2;AKR1C3;SNORD118 | -0.893 | (0.220) | 4.8E-05 | 1.4E-03 |
| Abdomen-RT | HCL | cg25790232 | chr16 | 58,215,272 | 58,215,274 | RP11-459F6.1 | -0.945 | (0.233) | 5.1E-05 | 1.4E-03 |
| Abdomen-RT | HCL | cg07872945 | chr9 | 134,010,913 | 134,010,915 | NA | -1.118 | (0.281) | 6.8E-05 | 1.7E-03 |
| Abdomen-RT | HCL | cg03065175 | chr4 | 154,740,605 | 154,740,607 | LRAT | 0.875 | (0.229) | 1.3E-04 | 3.1E-03 |
| Abdomen-RT | HCL | cg08271909 | chr4 | 154,740,538 | 154,740,540 | LRAT | 0.836 | (0.220) | 1.5E-04 | 3.2E-03 |
| Abdomen-RT | HCL | cg19152802 | chr5 | 110,514,185 | 110,514,187 | MIR548F3;TMEM232 | -0.845 | (0.226) | 1.9E-04 | 3.9E-03 |
| Abdomen-RT | HCL | cg05293861 | chr12 | 4,153,541 | 4,153,543 | NA | -0.672 | (0.184) | 2.6E-04 | 5.1E-03 |
| Abdomen-RT | HCL | cg13329407 | chr21 | 24,429,059 | 24,429,061 | AP000476.1 | -0.493 | (0.136) | 2.8E-04 | 5.2E-03 |
| Abdomen-RT | HCL | cg21393163 | chr1 | 12,157,571 | 12,157,573 | NA | -0.780 | (0.216) | 3.0E-04 | 5.3E-03 |
| Abdomen-RT | HCL | cg12597694 | chr16 | 8,183,208 | 8,183,210 | NA | -0.629 | (0.178) | 4.0E-04 | 6.3E-03 |
| Abdomen-RT | HCL | cg13224583 | chr1 | 156,913,952 | 156,913,954 | PEAR1 | 1.003 | (0.283) | 4.0E-04 | 6.3E-03 |
| Abdomen-RT | HCL | cg14263063 | chr16 | 21,150,859 | 21,150,861 | DNAH3 | -0.747 | (0.215) | 5.1E-04 | 7.5E-03 |
| Abdomen-RT | HCL | cg24769355 | chr9 | 72,041,875 | 72,041,877 | NA | -0.720 | (0.207) | 5.2E-04 | 7.5E-03 |
| Abdomen-RT | HCL | cg05211447 | chr14 | 103,880,242 | 103,880,244 | CTD-2134A5.4 | -0.782 | (0.227) | 5.9E-04 | 7.8E-03 |
| Abdomen-RT | HCL | cg26216433 | chr14 | 103,880,253 | 103,880,255 | CTD-2134A5.4 | -0.880 | (0.256) | 5.9E-04 | 7.8E-03 |
| Abdomen-RT | HCL | cg18462381 | chr10 | 127,738,453 | 127,738,455 | FOXI2;RP11-288A5.2 | 0.766 | (0.224) | 6.4E-04 | 8.0E-03 |
| Abdomen-RT | HCL | cg18661060 | chr10 | 71,758,477 | 71,758,479 | CDH23;VSIR | -1.227 | (0.360) | 6.6E-04 | 8.0E-03 |
| Abdomen-RT | HCL | cg26621020 | chr8 | 122,647,819 | 122,647,821 | NA | -0.646 | (0.191) | 7.2E-04 | 8.5E-03 |
| Abdomen-RT | HCL | cg12713583 | chr19 | 940,723 | 940,725 | ARID3A | -0.616 | (0.184) | 8.0E-04 | 9.1E-03 |
| Abdomen-RT | HCL | cg21945120 | chr10 | 58,157,562 | 58,157,564 | NA | -0.808 | (0.243) | 9.0E-04 | 9.9E-03 |
| Abdomen-RT | HCL | cg22234488 | chr2 | 105,179,803 | 105,179,805 | NA | -0.876 | (0.266) | 9.8E-04 | 1.0E-02 |
| Abdomen-RT | HCL | cg19634849 | chr5 | 140,259,739 | 140,259,741 | CYSTM1;PFDN1 | -1.210 | (0.372) | 1.1E-03 | 1.2E-02 |
| Abdomen-RT | HCL | cg08066673 | chr14 | 51,859,028 | 51,859,030 | GNG2 | -1.137 | (0.351) | 1.2E-03 | 1.2E-02 |
| Abdomen-RT | HCL | cg16146033 | chr11 | 62,999,850 | 62,999,852 | SLC22A8 | -0.846 | (0.265) | 1.4E-03 | 1.4E-02 |
| Abdomen-RT | HCL | cg07403981 | chr1 | 98,792,375 | 98,792,377 | NA | -0.725 | (0.228) | 1.5E-03 | 1.4E-02 |
| Abdomen-RT | HCL | cg16902294 | chr4 | 154,740,779 | 154,740,781 | LRAT | 0.793 | (0.251) | 1.6E-03 | 1.4E-02 |
| Abdomen-RT | HCL | cg19075225 | chr2 | 241,070,097 | 241,070,099 | SNED1 | 0.637 | (0.202) | 1.6E-03 | 1.4E-02 |
| Abdomen-RT | HCL | cg22948745 | chr8 | 138,688,051 | 138,688,053 | COL22A1 | -0.680 | (0.218) | 1.9E-03 | 1.6E-02 |
| Abdomen-RT | HCL | cg25840926 | chr2 | 20,448,225 | 20,448,227 | RHOB | 0.676 | (0.221) | 2.2E-03 | 1.9E-02 |
| Abdomen-RT | HCL | cg03054277 | chr1 | 228,212,515 | 228,212,517 | OBSCN;OBSCN-AS1 | 1.042 | (0.345) | 2.6E-03 | 2.1E-02 |
| Abdomen-RT | HCL | cg25433259 | chr6 | 158,850,751 | 158,850,753 | OSTCP1 | -1.242 | (0.415) | 2.7E-03 | 2.2E-02 |
| Abdomen-RT | HCL | cg09466904 | chr2 | 26,969,319 | 26,969,321 | AC013472.4;MAPRE3 | -0.606 | (0.203) | 2.8E-03 | 2.2E-02 |
| Abdomen-RT | HCL | cg16300030 | chr6 | 32,941,202 | 32,941,204 | HLA-DMB;XXbac-BPG181M17.5 | -0.668 | (0.224) | 2.9E-03 | 2.2E-02 |
| Abdomen-RT | HCL | cg17061862 | chr11 | 9,568,883 | 9,568,885 | NA | -0.786 | (0.271) | 3.8E-03 | 2.8E-02 |
| Abdomen-RT | HCL | cg15246238 | chr7 | 5,595,502 | 5,595,504 | FSCN1 | -0.797 | (0.276) | 3.9E-03 | 2.8E-02 |
| Abdomen-RT | HCL | cg18598117 | chr19 | 941,125 | 941,127 | ARID3A | -0.674 | (0.235) | 4.1E-03 | 2.9E-02 |
| Abdomen-RT | HCL | cg19758958 | chr11 | 62,551,749 | 62,551,751 | AHNAK | -1.235 | (0.431) | 4.1E-03 | 2.9E-02 |
| Abdomen-RT | HCL | cg08585946 | chr2 | 234,492,122 | 234,492,124 | NA | -1.149 | (0.402) | 4.2E-03 | 2.9E-02 |
| Abdomen-RT | HCL | cg21025494 | chr7 | 23,463,124 | 23,463,126 | IGF2BP3 | -0.969 | (0.341) | 4.5E-03 | 2.9E-02 |
| Abdomen-RT | HCL | cg26663696 | chr3 | 160,403,486 | 160,403,488 | MIR15B;MIR16-2;RP11-432B6.3;SMC4 | -0.533 | (0.188) | 4.5E-03 | 2.9E-02 |
| Abdomen-RT | HCL | cg13552692 | chr18 | 68,722,209 | 68,722,211 | CCDC102B | -0.709 | (0.250) | 4.5E-03 | 2.9E-02 |
| Abdomen-RT | HCL | cg05657694 | chr3 | 100,635,045 | 100,635,047 | ADGRG7 | -0.524 | (0.186) | 4.9E-03 | 3.1E-02 |
| Abdomen-RT | HCL | cg21781784 | chr1 | 227,437,757 | 227,437,759 | NA | -0.565 | (0.203) | 5.3E-03 | 3.3E-02 |
| Abdomen-RT | HCL | cg08587685 | chr10 | 114,632,446 | 114,632,448 | ABLIM1 | -1.255 | (0.451) | 5.4E-03 | 3.3E-02 |
| Abdomen-RT | HCL | cg02001279 | chr19 | 940,966 | 940,968 | ARID3A | -0.919 | (0.332) | 5.7E-03 | 3.4E-02 |
| Abdomen-RT | HCL | cg15143202 | chr3 | 169,148,736 | 169,148,738 | MECOM | -0.571 | (0.207) | 5.8E-03 | 3.4E-02 |
| Abdomen-RT | HCL | cg14122922 | chr20 | 44,714,873 | 44,714,875 | KCNK15-AS1;WISP2 | -0.739 | (0.268) | 5.9E-03 | 3.4E-02 |
| Abdomen-RT | HCL | cg21242123 | chr12 | 52,244,349 | 52,244,351 | KRT7 | -0.870 | (0.320) | 6.6E-03 | 3.8E-02 |
| Abdomen-RT | HCL | cg03479114 | chr4 | 165,359,572 | 165,359,574 | NA | -0.604 | (0.225) | 7.3E-03 | 4.1E-02 |
| Abdomen-RT | HCL | cg06586734 | chr4 | 61,143,607 | 61,143,609 | RP11-16N2.1 | -0.587 | (0.220) | 7.5E-03 | 4.1E-02 |
| Abdomen-RT | HCL | cg11414254 | chr5 | 177,022,010 | 177,022,012 | UIMC1;ZNF346 | -0.658 | (0.247) | 7.7E-03 | 4.2E-02 |
| Abdomen-RT | HCL | cg13910395 | chr22 | 43,577,140 | 43,577,142 | EFCAB6 | -0.814 | (0.308) | 8.2E-03 | 4.4E-02 |
| Abdomen-RT | HCL | cg21359950 | chr12 | 93,689,693 | 93,689,695 | CRADD | -0.508 | (0.193) | 8.7E-03 | 4.6E-02 |
| Brain-RT | Obesity | cg26572901 | chr7 | 27,030,741 | 27,030,743 | NA | 1.426 | (0.303) | 2.5E-06 | 2.3E-05 |
| Brain-RT | Obesity | cg21163477 | chr16 | 17,345,306 | 17,345,308 | XYLT1 | 1.679 | (0.375) | 7.4E-06 | 2.9E-05 |
| Brain-RT | Obesity | cg12715065 | chr6 | 14,241,208 | 14,241,210 | NA | 2.002 | (0.452) | 9.5E-06 | 2.9E-05 |
| Brain-RT | Obesity | cg10843537 | chr12 | 123,041,668 | 123,041,670 | PITPNM2 | 1.166 | (0.307) | 1.4E-04 | 2.6E-04 |
| Brain-RT | Obesity | cg12608132 | chr2 | 46,337,102 | 46,337,104 | EPAS1 | 1.077 | (0.283) | 1.4E-04 | 2.6E-04 |
| Brain-RT | Obesity | cg18666944 | chr6 | 16,737,189 | 16,737,191 | ATXN1 | 1.078 | (0.294) | 2.4E-04 | 3.6E-04 |
| Brain-RT | Obesity | cg13360224 | chr6 | 14,232,103 | 14,232,105 | RP11-359N11.1 | 1.207 | (0.346) | 4.9E-04 | 6.3E-04 |
| Corticosteroids | Obesity | cg22351187 | chr12 | 52,192,304 | 52,192,306 | KRT80 | -1.279 | (0.329) | 1.0E-04 | 8.3E-04 |

| **Table S6. Multivariable associations of treatment exposures with CHCs** | | | | | |
| --- | --- | --- | --- | --- | --- |
| CHC | Characteristics | Estimate | (SE) | z-value | *P* |
| AGM | (Intercept) | -2.081 | (0.460) | -4.520 | 6.19E-06 |
|  | Attained age | 0.014 | (0.008) | 1.753 | 7.96E-02 |
|  | Gender, female vs. male | -0.636 | (0.136) | -4.692 | 2.70E-06 |
|  | Polygenic risk score | 0.020 | (0.043) | 0.463 | 6.44E-01 |
|  | Chest-RT | -0.946 | (0.327) | -2.893 | 3.82E-03 |
|  | abdomen-RT | 1.270 | (0.393) | 3.228 | 1.24E-03 |
|  | Pelvis-RT | 0.064 | (0.271) | 0.236 | 8.14E-01 |
|  | Brain-RT | 0.201 | (0.155) | 1.299 | 1.94E-01 |
|  | Anthracyclines | 0.070 | (0.165) | 0.426 | 6.70E-01 |
|  | Alkylating agents, classic | -0.170 | (0.153) | -1.108 | 2.68E-01 |
|  | Corticosteroids | 0.009 | (0.253) | 0.036 | 9.71E-01 |
|  | Vinca alkaloids | 0.245 | (0.198) | 1.233 | 2.17E-01 |
|  | Epipodophyllotoxins | -0.282 | (0.187) | -1.505 | 1.32E-01 |
|  | Asparaginase enzymes | -0.161 | (0.250) | -0.644 | 5.20E-01 |
|  | Antimetabolites | 0.233 | (0.235) | 0.992 | 3.21E-01 |
| CM | (Intercept) | -3.089 | (0.418) | -7.396 | 1.41E-13 |
|  | Attained age | 0.016 | (0.010) | 1.685 | 9.20E-02 |
|  | Gender, female vs. male | -0.295 | (0.169) | -1.746 | 8.08E-02 |
|  | Chest-RT | -0.194 | (0.329) | -0.589 | 5.56E-01 |
|  | abdomen-RT | 0.743 | (0.402) | 1.847 | 6.47E-02 |
|  | Pelvis-RT | -0.240 | (0.318) | -0.753 | 4.51E-01 |
|  | Brain-RT | -0.058 | (0.206) | -0.283 | 7.77E-01 |
|  | Anthracyclines | 0.544 | (0.222) | 2.445 | 1.45E-02 |
|  | Alkylating agents, classic | 0.308 | (0.200) | 1.537 | 1.24E-01 |
|  | Corticosteroids | 0.048 | (0.296) | 0.162 | 8.71E-01 |
|  | Vinca alkaloids | -0.192 | (0.252) | -0.762 | 4.46E-01 |
|  | Epipodophyllotoxins | -0.161 | (0.231) | -0.697 | 4.86E-01 |
|  | Asparaginase enzymes | -0.147 | (0.314) | -0.468 | 6.40E-01 |
|  | Antimetabolites | -0.047 | (0.274) | -0.170 | 8.65E-01 |
| HCL | (Intercept) | -10.593 | (1.577) | -6.716 | 1.87E-11 |
|  | Attained age | 0.036 | (0.007) | 5.031 | 4.88E-07 |
|  | Gender, female vs. male | -0.219 | (0.116) | -1.882 | 5.99E-02 |
|  | Polygenic risk score | 0.034 | (0.006) | 5.440 | 5.34E-08 |
|  | Chest-RT | -0.049 | (0.229) | -0.214 | 8.31E-01 |
|  | abdomen-RT | 0.677 | (0.314) | 2.159 | 3.09E-02 |
|  | Pelvis-RT | -0.384 | (0.271) | -1.416 | 1.57E-01 |
|  | Brain-RT | 0.458 | (0.139) | 3.298 | 9.73E-04 |
|  | Anthracyclines | 0.160 | (0.147) | 1.082 | 2.79E-01 |
|  | Alkylating agents, classic | -0.013 | (0.136) | -0.099 | 9.21E-01 |
|  | Corticosteroids | 0.364 | (0.226) | 1.612 | 1.07E-01 |
|  | Vinca alkaloids | 0.182 | (0.178) | 1.022 | 3.07E-01 |
|  | Epipodophyllotoxins | -0.385 | (0.165) | -2.333 | 1.96E-02 |
|  | Asparaginase enzymes | -0.229 | (0.227) | -1.010 | 3.12E-01 |
|  | Antimetabolites | -0.023 | (0.209) | -0.111 | 9.11E-01 |
| HTG | (Intercept) | -2.027 | (0.291) | -6.961 | 3.37E-12 |
|  | Attained age | 0.016 | (0.007) | 2.298 | 2.15E-02 |
|  | Gender, female vs. male | -0.721 | (0.123) | -5.876 | 4.19E-09 |
|  | Polygenic risk score | 0.394 | (0.085) | 4.624 | 3.76E-06 |
|  | Chest-RT | -0.262 | (0.260) | -1.010 | 3.12E-01 |
|  | abdomen-RT | 1.038 | (0.326) | 3.183 | 1.46E-03 |
|  | Pelvis-RT | -0.498 | (0.265) | -1.882 | 5.99E-02 |
|  | Brain-RT | 0.560 | (0.140) | 3.989 | 6.63E-05 |
|  | Anthracyclines | 0.075 | (0.156) | 0.483 | 6.29E-01 |
|  | Alkylating agents, classic | -0.162 | (0.143) | -1.132 | 2.58E-01 |
|  | Corticosteroids | 0.336 | (0.237) | 1.415 | 1.57E-01 |
|  | Vinca alkaloids | -0.014 | (0.189) | -0.075 | 9.40E-01 |
|  | Epipodophyllotoxins | 0.271 | (0.169) | 1.607 | 1.08E-01 |
|  | Asparaginase enzymes | -0.215 | (0.237) | -0.905 | 3.65E-01 |
|  | Antimetabolites | -0.047 | (0.224) | -0.210 | 8.34E-01 |
| HTN | (Intercept) | 0.082 | (0.260) | 0.314 | 7.53E-01 |
|  | Attained age | 0.016 | (0.007) | 2.215 | 2.68E-02 |
|  | Gender, female vs. male | -1.023 | (0.114) | -8.934 | 4.10E-19 |
|  | Chest-RT | -0.518 | (0.236) | -2.195 | 2.81E-02 |
|  | abdomen-RT | 0.378 | (0.308) | 1.226 | 2.20E-01 |
|  | Pelvis-RT | -0.004 | (0.258) | -0.015 | 9.88E-01 |
|  | Brain-RT | 0.111 | (0.138) | 0.802 | 4.23E-01 |
|  | Anthracyclines | 0.184 | (0.151) | 1.220 | 2.22E-01 |
|  | Alkylating agents, classic | -0.046 | (0.134) | -0.348 | 7.28E-01 |
|  | Corticosteroids | 0.371 | (0.235) | 1.576 | 1.15E-01 |
|  | Vinca alkaloids | -0.124 | (0.170) | -0.731 | 4.65E-01 |
|  | Epipodophyllotoxins | -0.138 | (0.153) | -0.903 | 3.66E-01 |
|  | Asparaginase enzymes | -0.374 | (0.231) | -1.620 | 1.05E-01 |
|  | Antimetabolites | 0.120 | (0.211) | 0.569 | 5.69E-01 |
| MI | (Intercept) | -5.747 | (0.827) | -6.952 | 3.60E-12 |
|  | Attained age | 0.047 | (0.018) | 2.520 | 1.17E-02 |
|  | Gender, female vs. male | -0.866 | (0.337) | -2.570 | 1.02E-02 |
|  | Chest-RT | 1.026 | (0.533) | 1.927 | 5.40E-02 |
|  | abdomen-RT | 1.033 | (0.539) | 1.916 | 5.54E-02 |
|  | Pelvis-RT | -0.939 | (0.455) | -2.062 | 3.92E-02 |
|  | Brain-RT | -0.115 | (0.448) | -0.256 | 7.98E-01 |
|  | Anthracyclines | 0.376 | (0.386) | 0.974 | 3.30E-01 |
|  | Alkylating agents, classic | 0.110 | (0.383) | 0.288 | 7.73E-01 |
|  | Corticosteroids | -0.707 | (0.537) | -1.317 | 1.88E-01 |
|  | Vinca alkaloids | 0.198 | (0.454) | 0.436 | 6.63E-01 |
|  | Epipodophyllotoxins | 0.364 | (0.466) | 0.780 | 4.35E-01 |
|  | Asparaginase enzymes | -0.493 | (0.752) | -0.656 | 5.12E-01 |
|  | Antimetabolites | -0.181 | (0.546) | -0.331 | 7.41E-01 |
| Obesity | (Intercept) | -0.488 | (0.459) | -1.062 | 2.88E-01 |
|  | Attained age | -0.004 | (0.007) | -0.634 | 5.26E-01 |
|  | Gender, female vs. male | -0.539 | (0.111) | -4.841 | 1.29E-06 |
|  | Polygenic risk score | 0.386 | (0.098) | 3.941 | 8.12E-05 |
|  | Chest-RT | -0.269 | (0.227) | -1.183 | 2.37E-01 |
|  | abdomen-RT | 0.056 | (0.304) | 0.183 | 8.55E-01 |
|  | Pelvis-RT | -0.423 | (0.245) | -1.725 | 8.45E-02 |
|  | Brain-RT | 0.422 | (0.138) | 3.054 | 2.26E-03 |
|  | Anthracyclines | -0.098 | (0.143) | -0.687 | 4.92E-01 |
|  | Alkylating agents, classic | -0.298 | (0.132) | -2.257 | 2.40E-02 |
|  | Corticosteroids | 0.664 | (0.214) | 3.097 | 1.95E-03 |
|  | Vinca alkaloids | -0.175 | (0.162) | -1.081 | 2.80E-01 |
|  | Epipodophyllotoxins | 0.101 | (0.148) | 0.680 | 4.97E-01 |
|  | Asparaginase enzymes | -0.210 | (0.222) | -0.944 | 3.45E-01 |
|  | Antimetabolites | -0.021 | (0.196) | -0.107 | 9.15E-01 |

| **Table S7. Previously published associations of blood-based DNA methylation on CpG sites with health conditions** | | | | | | |
| --- | --- | --- | --- | --- | --- | --- |
| CpG | Current study | Previously published study | | | | |
|  | Mediation | Related trait | Correlation | *P* | Reference | PMID |
| cg09853238 | Abdomen RT - HTG | systemic lupus erythematosus (SLE) | Negative | 6.9E-82 | Imgenberg-Kreuz et al. 2018 | 29437559 |
| cg09853238 | Abdomen RT - HTG | down syndrome | Positive | 1.3E-06 | Henneman et al. 2018 | 29601581 |
| cg26572901 | Brain RT - Obesity | down syndrome | Negative | 2.3E-05 | Henneman et al. 2018 | 29601581 |
| cg13360224 | Brain RT - HTG | myalgic encephalomyelitis/chronic fatigue syndrome | Negative | 8.7E-05 | Trivedi et al. 2018 | 30036399 |
| cg13552692 | Abdomen RT - HTG | aging | Negative | 2.1E-13 | Pérez et al. 2019 | 30626398 |
| cg21922478 | Abdomen RT - HCL | aging | Negative | 7.8E-13 | Pérez et al. 2019 | 30626398 |
| cg06963130 | Abdomen RT - HCL | aging | Negative | 3.0E-12 | Pérez et al. 2019 | 30626398 |
| cg12715065 | Brain RT - Obesity | Crohn's disease (CD) | Positive | 1.5E-05 | Somineni et al. 2019 | 30779925 |
| cg09853238 | Abdomen RT - HTG | systemic lupus erythematosus (SLE) | Negative | 6.9E-82 | Imgenberg-Kreuz et al. 2019 | 31428085 |
| cg09853238 | Abdomen RT - HTG | primary Sjögren's Syndrome (pSS) | Negative | 1.5E-20 | Imgenberg-Kreuz et al. 2019 | 31428085 |
| cg22351187 | Corticosteroids | SETD1B-related syndrome | Negative | 6.7E-03 | Krzyzewska et al. 2019 | 31685013 |
| cg13552692 | Abdomen RT - HTG | aging | Negative | 9.7E-319 | McCartney et al. 2019 | 31892350 |
| cg21163477 | Brain RT - Obesity | Gulf War Illness | Positive | 7.4E-06 | Trivedi et al. 2019 | 30920300 |
